# Subjective and Clinically Assessed Hearing Loss; A Cross-Sectional Register-Based Study on a Swedish Population Aged 18 through 50 Years

**DOI:** 10.1371/journal.pone.0123290

**Published:** 2015-04-13

**Authors:** Pernilla Videhult Pierre, Ann-Christin Johnson, Anders Fridberger

**Affiliations:** 1 Division of Audiology, Department of Clinical Science, Intervention, and Technology, Karolinska Institutet, Stockholm, Sweden; 2 Department of Surgical Sciences, Uppsala University, Uppsala, Sweden; 3 Division of Cell Biology, Department of Clinical and Experimental Medicine, Linköping University, Linköping, Sweden; University of Modena and Reggio Emilia, ITALY

## Abstract

**Objectives:**

Questionnaire studies suggest that hearing is declining among young adults. However, few studies have examined the reliability of hearing questionnaires among young adult subjects. This study examined the associations between pure tone audiometrically assessed (PTA) hearing loss and questionnaire responses in young to middle aged adults.

**Materials and Methods:**

A cross-sectional study using questionnaire and screening PTA (500 through 6000 Hz) data from 15322 Swedish subjects (62% women) aged 18 through 50 years. PTA hearing loss was defined as a hearing threshold above 20 dB in both ears at one or more frequencies. Data were analysed with chi-square tests, nonlinear regression, binary logistic regression, and the generalized estimating equation (GEE) approach.

**Results:**

The prevalence of PTA hearing loss was 6.0% in men and 2.9% in women (*p* < 0.001). Slight hearing impairment was reported by 18.5% of the men and 14.8% of the women (*p* < 0.001), whereas 0.5% of men and women reported very impaired hearing. Using multivariate GEE modelling, the odds ratio of PTA hearing loss was 30.4 (95% CI, 12.7-72.9) in men and 36.5 (17.2-77.3) in women reporting very impaired hearing. The corresponding figures in those reporting slightly impaired hearing were 7.06 (5.25-9.49) in men and 8.99 (6.38-12.7) in women. These values depended on the sound stimulus frequency (*p* = 0.001). The area under the ROC curve was 0.904 (0.892-0.915) in men and 0.886 (0.872-0.900) in women.

**Conclusions:**

Subjective hearing impairment predicted clinically assessed hearing loss, suggesting that there is cause for concern as regards the future development of hearing in young to middle-aged people.

## Introduction

Recent large-scale questionnaire studies have reported an increasing prevalence of subjective hearing impairment in young and middle-aged people [1]. Questionnaire data on hearing problems are however often difficult to interpret, because responses may be affected by many factors, including sensory cell function [2], general health [3], symptom severity [4], culture [5], expectations [6], and labor market participation [4]. It is unclear whether subjective hearing impairment in young adults is correlated to hearing threshold elevations measured with pure tone audiometry, the current gold standard for hearing assessment, as few studies examined the reliability of questionnaires in this age group.

The available data on hearing loss prevalence are also conflicting. Several studies found a successive increase in the prevalence of pure tone audiometrically assessed (PTA) hearing loss during the past 30 years [7, 8] and worldwide, there is growing concern that hearing loss is increasing due to the extensive leisure time noise exposure in modern society, in particular through personal listening devices [9, 10]. Other studies showed the opposite trend. The PTA hearing of older adults was found to be better than previous generations in an American study [11]. Another study concluded that the PTA hearing of Americans aged 25 through 64 years was better or equally good in 1999 through 2004 than 40 years earlier [12]. This positive development may be due to improved economic and social welfare, including better medical care for children with ear infections [13] and reduced occupational noise exposure [14].

In this cross-sectional study, we aimed to determine the relation between PTA and subjective hearing impairment in young and middle aged subjects. We found that subjective hearing impairment predicted PTA hearing loss, provided that age, sex, tinnitus, and sound stimulus frequency were included in the models. Thus, should the increase in self-reported hearing problems continue [1], there is cause for concern about the future hearing of young to middle-aged people.

## Materials and Methods

### Sample

A cross-sectional study was conducted, using data from LifeGene, a Swedish national resource for research on the relationships among heredity, environment, and lifestyle. LifeGene, which included its first participant in 2009, collects data via a comprehensive web-based questionnaire and also performs extensive physiological testing along with blood and urine sampling. A random sample of Swedish inhabitants aged 18 through 50 years is invited to participate, but it is also possible to enroll without an invitation, through LifeGene’s web site. Further details can be found elsewhere [15].

Included in the present investigation were all subjects that answered the question “How is your hearing?” in any of the years 2009 through 2012, had their hearing measured with screening audiometry, and were between 18 and 50 years of age at the time of the PTA assessment (n = 5809 men and 9513 women), which were 84% of all subjects that had enrolled in LifeGene.

### Audiometry

Frequency-specific screening PTA measurements were performed by a special-trained nurse at one of LifeGene’s test centers, using SA 202 audiometers (Entomed AB, Malmö, Sweden) and sound-isolating headphones (Sennheiser HDA 200, Sennheiser electronic GmbH & Co KG, Wedemark, Germany), according to procedures established by the International Standards Organization (ISO 8253–1). In short, air conduction hearing thresholds were determined by presenting pure tones at 0 dB or 10 dB, at the ordered frequencies 1000, 2000, 3000, 4000, 6000, and 500 Hz. If no response was obtained, the stimulus level was increased in 5-dB steps, until the subject heard the tone or the maximum level of the audiometer, 110 dB, was reached. Masking of the opposite ear was not performed. In the present study, a hearing threshold below 10 dB and above 110 dB was assigned the value 10 dB and 111 dB, respectively. A pure tone hearing threshold above 20 dB in both ears at one or several frequencies was defined as PTA hearing loss.

### Questionnaire

Multifaceted data on phenotypes and exposures is gathered through LifeGene’s questionnaire, which includes several queries related to hearing. In the present study, the following items were used (the original formulations in Swedish are found in S1 Table):

- Q1. ”How is your hearing?”“Good”;”Slightly impaired”;”Very impaired”.
- Q2. ”Is it difficult for you to hear when talking with one person in a quiet room?”“No, not at all”;”Sometimes a bit difficult”;”Yes, very difficult”;”Do not know/Do not want to answer”.
- Q3. ”Is it difficult for you to hear when talking with several persons at the same time?”“No, not at all”;”Sometimes a bit difficult”;”Yes, very difficult”;”Do not know/Do not want to answer”.
- Q4. ”Do you have a constant ringing or some other disturbing sound in your ears (tinnitus)?”“No”;”Yes, sometimes, but the sound does not disturb me”;”All the time, the sound is very disturbing”;”Do not know/Do not want to answer”.

All included subjects had answered Q1, whereas there were 27% missing answers on each of Q2 and Q3. Only one subject had not answered Q4.

LifeGene’s questionnaire also contains one query on educational level,”What is the highest educational level you have achieved or are currently studying for?” There are five possible answers:”Compulsory school”;”Upper secondary school”;”University”;”Other”;”Do not know/Do not want to answer”. In the present study, educational data were used for descriptive statistics and were missing in 0.5% of subjects.

Information on residence was based on the postal code reported by the participant when answering LifeGene’s questionnaire. If this information was lacking, the postal code of subjects actively invited to participate in LifeGene was obtained from a computerized register. Considering the localities of the numbers and the classification systems used by Statistics Sweden [16], the following residential categories were constructed:”Metropolitan area of Stockholm” (numbers 10 through 19),”Metropolitan area of Malmö or Gothenburg” (numbers 20 through 28 and 40 through 51, respectively),”Region of Umeå” (numbers 90 through 92), and”Elsewhere” (all other numbers). Resident data were used for descriptive statistics and were missing in 0.1% of the men and 0.2% of the women.

### Statistics

Statistical calculations were performed with IBM SPSS Statistics version 21 using the chi-square test, nonlinear regression, binary logistic regression, or the generalized estimating equation (GEE) approach, as appropriate.

The results of binary logistic regression are presented as odds ratios (ORs) followed by p-values within parentheses. Whenever age was a statistically significant predictor of the dependent variable, age adjustment was performed with age as a continuous variable. The adequacy of the models was evaluated with goodness-of-fit statistics based on the Hosmer-Lemeshow decile-of-risk test [17], and a model was rejected if *p* < 0.05.

GEEs was used to investigate the associations between PTA and subjective hearing. The GEE approach allows for analysis of clustered data [18], which in the present investigation arose because hearing thresholds are measured at several different frequencies in each subject. The results are presented as ORs with p-values and 95% confidence intervals (CIs). The quasi likelihood under independence model criterion (QIC) value was used to establish the appropriate working correlation matrix. The discriminatory performance of the model was evaluated with the area under the receiver operating characteristic curve (AUC_ROC_) [18], which was calculated with the trapezoidal approach. The receiver operating characteristic (ROC) curve was constructed using each subject’s frequency-specific predicted probability. Men and women were analyzed separately. Due to the many missing answers on Q2 and Q3, association analysis with PTA hearing was performed with Q1 and Q4 only.

When performing calculations, the response categories of the hearing questions were in some instances reduced in order to increase the statistical power. In these cases, the tinnitus category”Yes, sometimes, but the sound does not disturb me” of Q4 was fused with”No”. All subjects that had answered”Do not know/Do not want to answer” or not answered at all on Q2, Q3, and Q4 were assigned to the same category as those reporting no problems on that specific question. This seemed as a reasonable strategy as almost all of them (99.8% for Q2, 99.9% for Q3, and 78.2% for Q4) had reported good hearing on question Q1.

The data on which our study is based can be found in S2 Table. Information on the variables and the response codes can be found in S3 Table.

### Ethics Statement

The study was approved by the Regional Ethics Board of Stockholm, Sweden (protocol no 2011/1827-31/1). Data were obtained from the Swedish national research project LifeGene. To participate in LifeGene, writted informed consent must be provided.

## Results

### Subjects

**Fig 1:**
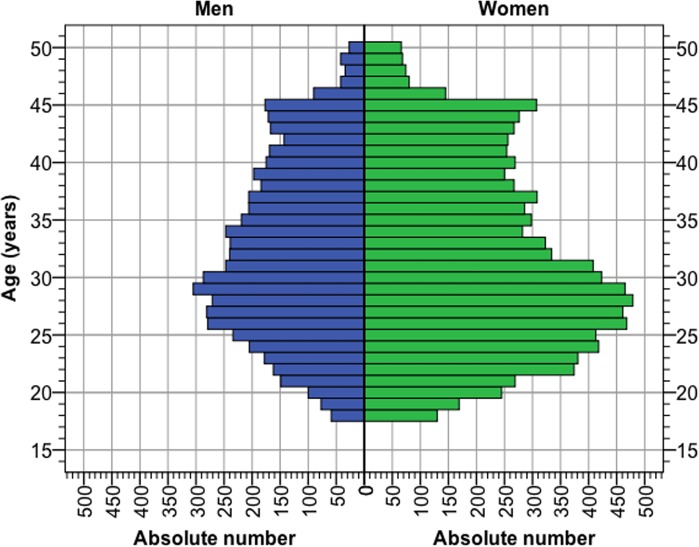
Age distribution of the study population stratified by sex.

The median age of the study population was 32 years in men and 31 years in women. The age distribution stratified by sex is shown in Fig 1, where it can be seen that a majority of study participants were less than 40 years of age.

76% of the men and 78% of the women lived in metropolitan areas. These figures are considerably larger than the fraction of metropolitan inhabitants in the Swedish population at large (42% of all male and 43% of all female Swedish inhabitants aged 18 through 50 years lived in metropolitan areas by the end of 2012; [19]).

The highest self-reported educational level was university in 64% of the men and 70% of the women and upper secondary school in 27% of the men and 21% of the women. Again, these figures are higher than those found in the general population, where 33% of men and 43% of women had a university education, whereas 50% of Swedish men and 43% of women had an upper secondary school education at the end of 2012 [19]. Hence, the population examined here is young, urban, and well educated.

### Prevalence of PTA hearing loss

The prevalence of PTA hearing loss, i.e. a hearing threshold above 20 dB in both ears at one or several frequencies, was 6.0% in men and 2.9% in women (*p* < 0.001; chi-square test). Its dependence on age could be described with Eq 1 for men and Eq 2 for women (R^2^ = 0.885 in men and 0.764 in women; weighted least square):

$$Prevalence_{men}=17.1-1.45\times Age+3.23\times 10^{-2}\times Age^{2}$$ (1)

$$Prevalence_{women}=6.31-5.06\times 10^{-1}\times Age+1.18\times 10^{-2}\times Age^{2}$$ (2)

**Fig 2:**
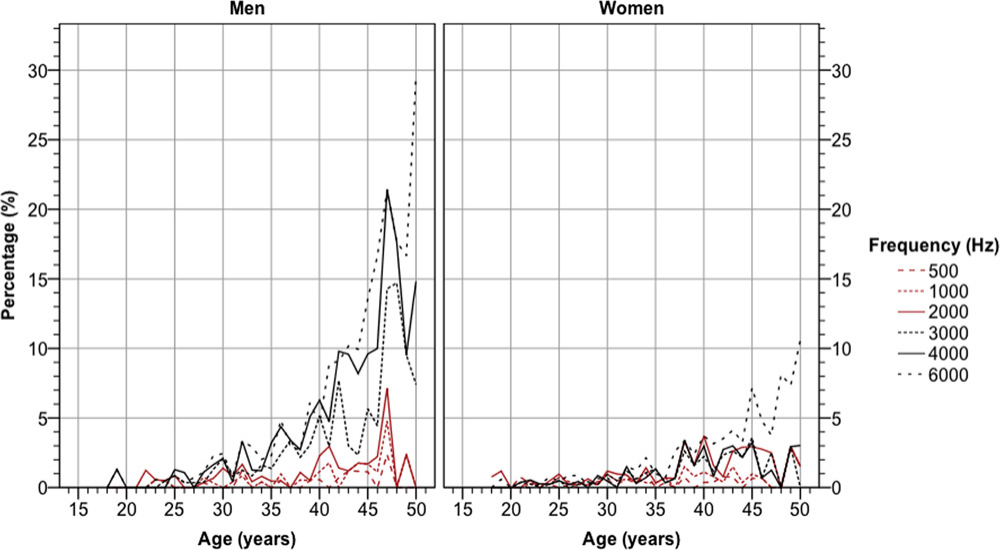
Prevalence of a pure tone hearing threshold above 20 dB in both ears versus age, stratified by frequency and sex.

These data show that sex and age are major predictors for hearing impairment in this population. Hearing impairment was more common at the highest frequencies (Fig 2). At the three highest frequencies, the prevalence of a hearing threshold above 20 dB in both ears was significantly higher in men than women; the age-adjusted OR for men to women was 2.23 (*p* < 0.001; binary logistic regression) at 3000 Hz, 3.44 (*p* < 0.001) at 4000 Hz, and 2.35 (*p* < 0.001) at 6000 Hz. At all other frequencies, the difference between men and women was non-significant.

### Prevalence of Subjective Hearing Impairment

**Fig 3:**
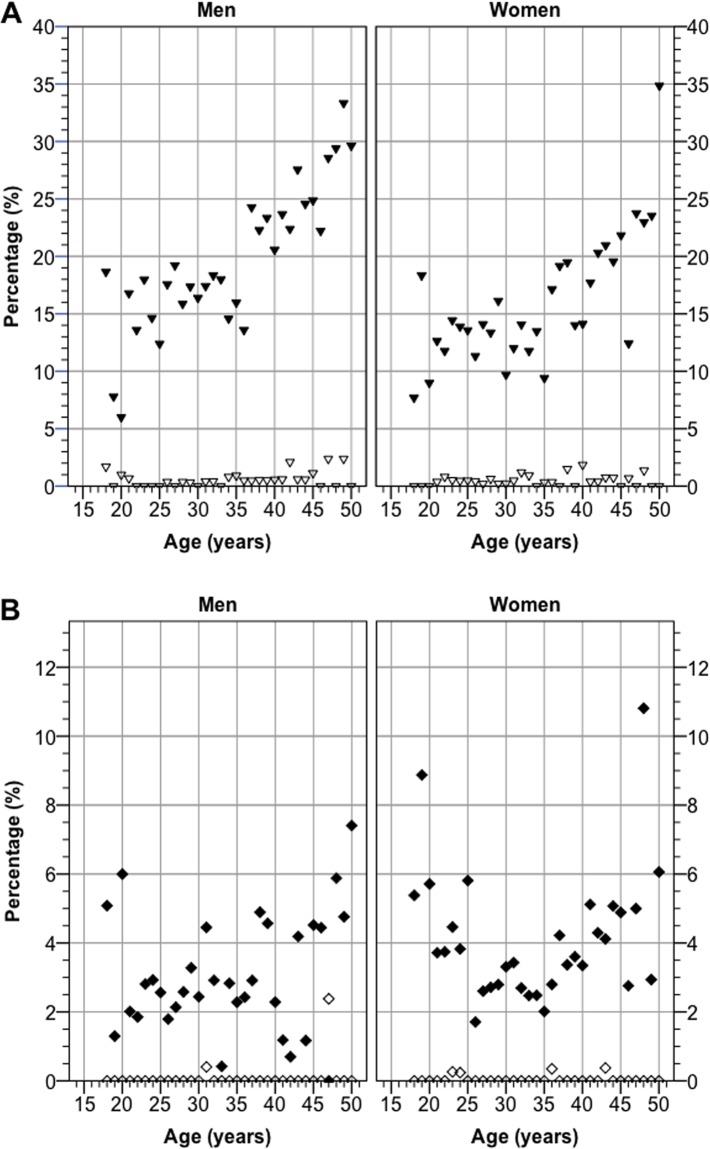
Prevalence of different self-assessed hearing problems versus age, stratified by sex. A, Percentage of subjects answering ”Slightly impaired” (closed triangles) or ”Very impaired” (open triangles) on the question ”How is your hearing?” (Q1). B, Percentage of subjects answering ”Sometimes a bit difficult” (closed squares) or ”Yes, very difficult” (open squares) on the question ”Is it difficult for you to hear when talking with one person in a quiet room?” (Q2). Missing answers and answers of ”Do not know/Do not want to answer” on Q2 were classified as absence of hearing difficulties.

Table 1 gives the responses on questions regarding different subjective hearing problems. Fig 3A and 3B show the age distribution of some of these results. Having slightly impaired hearing (Q1) was significantly more common among men than women (*p* < 0.001; chi-square) as was sometimes having tinnitus (*p* < 0.001; chi-square), whereas having slight difficulties to hear when talking with one person in a quiet room (Q2) was most common among women (*p* = 0.019; chi-square). All other sex differences were non-significant.

**Table 1:** Self-reported hearing.

**Question** ^a^	**Men**	**Women**
	**(n = 5809)**	**(n = 9513)**
**Q1. How is your hearing?**		
**Good.**	4705 (81.0)	8059 (84.7)
**Slightly impaired.**	1077 (18.5)	1408 (14.8)
**Very impaired.**	27 (0.5)	46 (0.5)
**Q2. Is it difficult for you to hear when talking with one person in a quiet room?**		
**No, not at all.**	4043 (69.6)	6581 (69.2)
**Sometimes a bit difficult.**	162 (2.8)	355 (3.7)
**Yes, very difficult.**	2 (0.0)	4 (0.0)
**Do not know/Do not want to answer.**	9 (0.2)	24 (0.3)
**Missing answer**	1593 (27.4)	2549 (26.8)
**Q3. Is it difficult for you to hear when talking with several persons at the same time?**		
**No, not at all.**	2797 (48.1)	4519 (47.5)
**Sometimes a bit difficult.**	1304 (22.4)	2204 (23.2)
**Yes, very difficult.**	104 (1.8)	220 (2.3)
**Do not know/Do not want to answer.**	11 (0.2)	21 (0.2)
**Missing answer**	1593 (27.4)	2549 (26.8)
**Q4. Do you have a constant ringing or some other disturbing sound in your ears (tinnitus)?**		
**No.**	4227 (72.8)	7510 (78.9)
**Yes, sometimes, but the sound does not disturb me.**	1427 (24.6)	1784 (18.8)
**Yes, all the time, the sound is very disturbing.**	124 (2.1)	163 (1.7)
**Do not know/Do not want to answer.**	31 (0.5)	55 (0.6)
**Missing answer**	0 (0.0)	1 (0.0)

The study population answered a web-based questionnaire on living conditions, habits, and health. The responses on questions on different aspects of hearing stratified by sex are shown. Absolute numbers are given followed by percentage within sex.

^a^The questions and answers have been translated from Swedish to English. The Swedish formulations are given in S1 Table.

### Association between PTA and subjective hearing loss

The relationship between PTA and subjective hearing was evaluated with the GEE approach. The results of the final model are presented in Table 2 as ORs of having a hearing threshold above 20 dB in both ears when using frequency, subjective hearing (Q1), tinnitus (Q4), and age as independent variables. PTA and subjective hearing were significantly associated in both sexes (*p* < 0.001 in both men and women).

**Table 2:** Odds ratios (ORs) of having PTA hearing loss^a^.

	Men				Women			
Independent variable			95% CI				95% CI	
	OR	P-value	Lower	Upper	OR	P-value	Lower	Upper
**Subjective hearing**								
Very impaired	30.4	<0.001	12.7	72.9	36.5	<0.001	17.2	77.3
Slightly impaired	7.06	<0.001	5.25	9.49	8.99	<0.001	6.38	12.7
Good	1				1			
**Frequency (Hz)**								
500	0.062	<0.001	0.025	0.154	0.036	<0.001	0.009	0.148
1000	0.037	<0.001	0.012	0.118	0.108	<0.001	0.046	0.251
2000	0.112	<0.001	0.056	0.222	0.543	0.008	0.347	0.850
3000	0.313	<0.001	0.204	0.479	0.415	<0.001	0.265	0.652
4000	0.607	0.001	0.447	0.823	0.434	<0.001	0.286	0.658
6000	1				1			
**Age (years)**	1.13	<0.001	1.11	1.15	1.09	<0.001	1.07	1.10
**Interaction of subjective hearing and frequency (Hz)**								
Very impaired and 500	4.54	0.022	1.24	16.6	14.2	0.002	2.65	76.6
Very impaired and 1000	12.2	<0.001	3.11	47.6	6.83	0.002	2.08	22.4
Very impaired and 2000	3.23	0.032	1.10	9.48	1.59	0.302	0.660	3.83
Very impaired and 3000	3.16	0.007	1.38	7.25	2.77	0.002	1.45	5.26
Very impaired and 4000	2.82	0.002	1.47	5.40	1.99	0.007	1.20	3.29
Very impaired and 6000	1				1			
Slightly impaired and 500	0.751	0.625	0.237	2.38	6.60	0.012	1.50	28.9
Slightly impaired and 1000	3.21	0.067	0.922	11.2	3.11	0.016	1.23	7.86
Slightly impaired and 2000	2.03	0.074	0.933	4.41	1.13	0.645	0.670	1.91
Slightly impaired and 3000	1.72	0.029	1.06	2.81	1.23	0.444	0.728	2.06
Slightly impaired and 4000	1.48	0.031	1.04	2.11	1.32	0.255	0.819	2.12
Slightly impaired and 6000	1				1			
Good and 500	1				1			
Good and 1000	1				1			
Good and 2000	1				1			
Good and 3000	1				1			
Good and 4000	1				1			
Good and 6000	1				1			
**Tinnitus** ^**b**^								
Severe	2.05	0.003	1.27	3.31	2.10	0.003	1.28	3.42
Not severe or unknown	1				1			

Association analysis was performed with the generalized estimating equation (GEE) approach. Working correlation matrix structure: unstructured; quasi likelihood under independence model criterion (QIC): 4481 for men and 4624 for women.

^a^PTA hearing loss was defined as a pure tone hearing threshold above 20 dB in both ears at one or several of the frequencies 500, 1000, 2000, 3000, 4000, and 6000 Hz.

^b^All subjects not answering ”All the time, the sound is very disturbing” on the tinnitus question ”Do you have a constant ringing or some other disturbing sound in your ears (tinnitus)” were assigned to the category ‘Not severe or unknown’, including the single missing answer.

**Fig 4:**
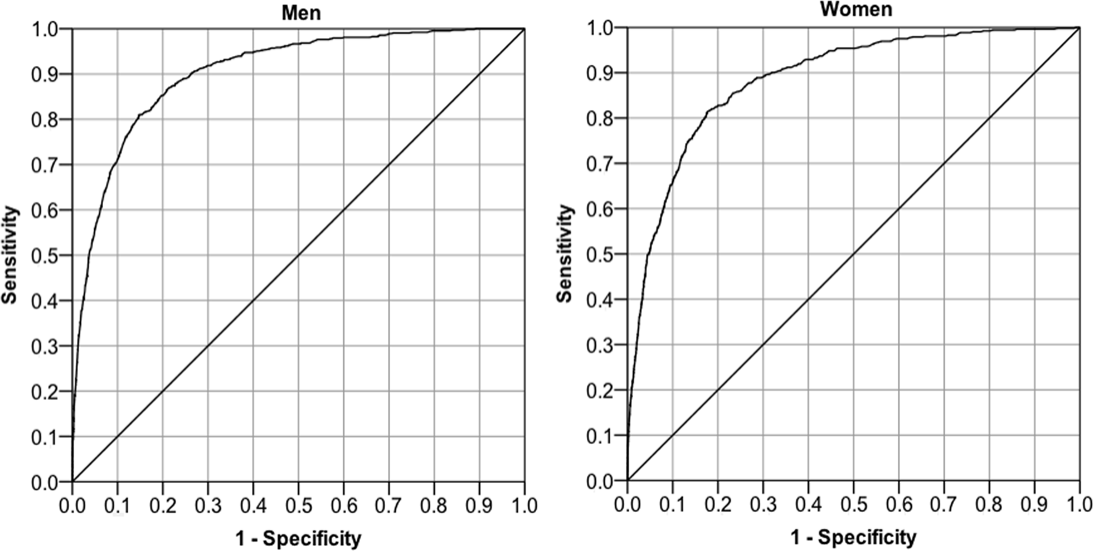
Receiver operating characteristic (ROC) curves of the generalized estimating equation model presented in Table 2.

There was a significant interaction between subjective hearing and frequency (*p* = 0.001 in men, *p* < 0.001 in women), generally contributing to an increased association between PTA and subjective hearing at lower frequencies. The ROC curves given in Fig 4 illustrate the discriminatory performance of the model in men and women. The AUC_ROC_ was 0.904 (95% CI, 0.892–0.915) in men and 0.886 (0.872–0.900) in women.

## Discussion

In this well-educated, urban, and young to middle-aged adult Swedish population, the prevalence of screened PTA hearing loss, i.e. a hearing threshold above 20 dB in both ears at one or several of the frequencies 500, 1000, 2000, 3000, 4000, and 6000 Hz, was 6.0% in men and 2.9% in women and was highly dependent on age. We found that subjective hearing problems predicted PTA hearing loss after adjusting for age, stimulus frequency, and the presence of tinnitus.

Most studies comparing self-reported and PTA hearing have been performed on populations with a high prevalence of PTA hearing loss, such as elderly populations (e.g. [20–22]. In agreement with the results found in the present investigation, a general finding is that self-report is a valuable tool for quick screening for hearing loss [20–22]. However, studies on younger subjects with a low prevalence of PTA hearing loss, as in the present investigation, often conclude that self-report fails to predict PTA hearing loss [23–25]. There are some main methodological differences that most likely contribute to this discrepancy and which will be discussed below:

1. The criterion used in order to define PTA hearing loss.
2. The questions and response alternatives used for assessing self-reported hearing.
3. The statistical method used for performing association analysis.

The prevalence of PTA hearing loss and its association with self-reported hearing impairment are affected by how PTA hearing loss is defined. In studies on young populations, a frequent criterion of PTA hearing loss is a hearing threshold of more than 20 dB at one or several frequencies in at least one ear [7, 26–28]. This criterion was not found reliable enough to use in the present study due to the risk of measurement errors confounding the results. We instead chose to define PTA hearing loss as having a hearing threshold above 20 dB in both ears at one or several frequencies. Using this criterion, we found that self-reported hearing impairment not only was highly correlated with PTA hearing loss but also could predict PTA hearing loss. In many studies performed on populations older than the present one, PTA hearing loss is defined as having a pure tone average of at least 25 dB at 500, 1000, 2000, and 4000 Hz uni- or bilaterally (e.g. [20–23, 25, 29, 30]). When employing such criteria on the present population, self-reported hearing impairment was highly correlated with PTA hearing loss, but it was not possible to elaborate an association model with a high predictive value due to the low prevalence of PTA hearing loss (data not shown). Another disadvantage with such criteria is that the minimum threshold level used in the present investigation was 10 dB and since many subjects in this young population probably had a much lower hearing threshold, taking the mean value would overestimate the prevalence rates. Still, the age-specific prevalence of a mean hearing threshold of at least 25 dB at 500, 1000, 2000, and 4000 Hz uni- or bilaterally was lower (data not shown) than in other investigations [23, 25, 29, 30].

The prevalence of subjective symptoms may be affected even by minor differences in questionnaire-wording [31]. In the present investigation, subjective hearing impairment was common, with 18.5% of the men and 14.8% of the women reporting slightly impaired hearing (Q1), and about one in four men and women reporting hearing difficulties when talking with several people (Q3). In a recent study on a population representative of the adult Swedish population, the prevalence of subjective hearing impairment was 9.7% in men and 8.3% in women aged 20 through 54 years [1]. Lower age-specific prevalences were also obtained in three other recent studies, on Swedish women [32] and young American adults [24, 28], respectively. Besides questionnaire-wording, a possible explanation for the somewhat discordant results is differences in study populations. The prevalence of self-reported hearing impairment increased with age, but much less than that of PTA hearing loss; subjective hearing problems were common already in young adulthood. This lower age-dependency of subjective than PTA hearing loss has been shown previously [24, 33]. Regarding tinnitus, constant disturbing tinnitus (Q4) was equally common among men (2.1%) and women (1.7%). No age-dependency was found (data not shown), in agreement with a previous study on Swedish urban dwellers [34]. That study found that 8.9% of men and 6.1% of women aged 20 through 49 years [34] had constant or nearly constant tinnitus, and other studies have confirmed these figures [30]. Most likely, the main reason for the higher prevalences is that those investigations include subjects with frequent or constant tinnitus that is not necessarily disturbing [30, 34], which the present study does not.

The statistical method used for performing association analysis clearly influences the outcome. Using GEEs, significant associations between self-reported and PTA hearing were found in the present investigation, and these were generally stronger at lower sound stimulus frequencies. When reviewing the literature, no other studies were found that used GEEs to investigate the relation between PTA and subjective hearing loss. The GEE approach is advantageous as it allows specification of a working correlation matrix that accounts for the within-subject correlation of PTA hearing data [18]. AUC_ROC_ is a summary measure of the discriminatory performance of a model with a dichotomous outcome measure, and in contrast to sensitivity and specificity, it is not dependent on how the predicted outcome values are classified, i.e. the cut-point [18]. An AUC_ROC_ of about 0.90, which was obtained here, can be classified as a very good discriminatory performance of a model [18]. AUC_ROC_ has been used as a performance measure in other studies on PTA and subjective hearing [22, 35, 36]. In comparison, the GEE model of the present investigation performs well, in particular when considering the overall good PTA hearing of the study population. ROC curves have also been used to determine the best criterium for PTA hearing loss in order to predict self-assessed hearing impairment [37]. AUC_ROC_ was not presented in that study, but a variety of other performance measures were, such as the sensitivity and specificity, which were 0.59 and 0.90, respectively, using a bilateral pure tone mean >25 dB at 0.5, 1, 2, and 4 kHz [37]. With the method used in the present investigation, the values of sensitivity and specificity for a given cut-point can easily be obtained by examining the data points of the ROC. For example, a cut-point of 0.0106 in men yielded a sensitivity of 0.90 and specificity of 0.74. In women, a sensitivity of 0.90 was obtained with a cut-point of 0.00459; the obtained specificity was then 0.68.

### Limitations of the study

The population examined here was urban, young, and with high education level, and the subjects were likely more interested in health issues than the average Swedish inhabitant, since many of them joined the LifeGene project without being actively recruited. The diversity of the LifeGene project however decreases the risk of a high representation of subjects interested in hearing issues specifically. Air-conduction screening PTA assessments were used, which were not performed at the same time as the subjective hearing assessments. It cannot be excluded that these factors may have influenced the results.

There were 27% missing answers each on Q2 and Q3. Since almost all of these had reported good hearing on the general question (Q1), they were classified as having no hearing problems with one or several persons, respectively. This may have led to underestimated prevalences of these difficulties.

## Conclusions

In the young to middle-aged, well-educated, and urban population investigated in this study, the overall prevalence of PTA hearing loss was lower than that of subjective hearing impairment. Still, we found that subjective hearing impairment predicted PTA hearing loss, provided that age, sex, tinnitus, and sound stimulus frequency were included in the models. Our results suggest that there is cause for concern about the future development of hearing problems in this population, since hearing problems generally worsen with age. Future studies should explore the longitudinal relation between subjective and PTA hearing and how it is influenced by factors unrelated to the hearing function, such as a reduced tolerance to hearing problems due to higher health expectations or higher demands of communication skills in modern society.

## Supporting Information

S1 Table The population of this Swedish study answered a web-based questionnaire on living conditions, habits, and health. Original Swedish formulations on hearing items are shown.
(PDF)

S2 Table The data on which the study is based. (XLSX)

S3 Table Information on the variables and the response codes used in S2 Table. (PDF)
