# Supplementary material for: Subjective and Clinically Assessed Hearing Loss; A Cross-Sectional Register-Based Study on a Swedish Population Aged 18 through 50 Years
Source: PLoS One. 2015 Apr 13;10(4):e0123290. doi: 10.1371/journal.pone.0123290 (PMC4395427; doi:10.1371/journal.pone.0123290)
Supplement: S1 Table — Original Swedish formulations on hearing items are shown. (PDF) [file pone.0123290.s001.pdf]

Q1. Hur är din hörsel?

- ☐ Bra.
- ☐ Något nedsatt.
- ☐ Mycket nedsatt.

Q2. Har du svårt att höra när du talar med en person i ett tyst rum?

- ☐ Nej, inte alls.
- ☐ Ibland, lite svårt.
- ☐ Ja, mycket svårt.
- ☐ Vet ej/vill ej svara.

Q3. Har du svårt att höra när du talar med flera personer samtidigt?

- ☐ Nej, inte alls.
- ☐ Ibland, lite svårt.
- ☐ Ja, mycket svårt.
- ☐ Vet ej/vill ej svara.

Q4. Ringer det konstant i öronen eller har du något annat besvärande ljud i öronen (tinnitus)?

- ☐ Nej.
- ☐ Hela tiden, ljudet är mycket störande.
- ☐ Ja, ibland, men ljudet stör mig inte.
- ☐ Vet ej/vill ej svara.
