# Supplementary material for: Subjective and Clinically Assessed Hearing Loss; A Cross-Sectional Register-Based Study on a Swedish Population Aged 18 through 50 Years
Source: PLoS One. 2015 Apr 13;10(4):e0123290. doi: 10.1371/journal.pone.0123290 (PMC4395427; doi:10.1371/journal.pone.0123290)
Supplement: S3 Table — (PDF) [file pone.0123290.s003.pdf]

Enkätöversikten

Här svarar du på frågor om dig och ditt liv, för att vi ska kunna ta reda på hur dina val, ditt arv, och din livsmiljö påverkar dig. Klicka på en ikon för att sätta igång.

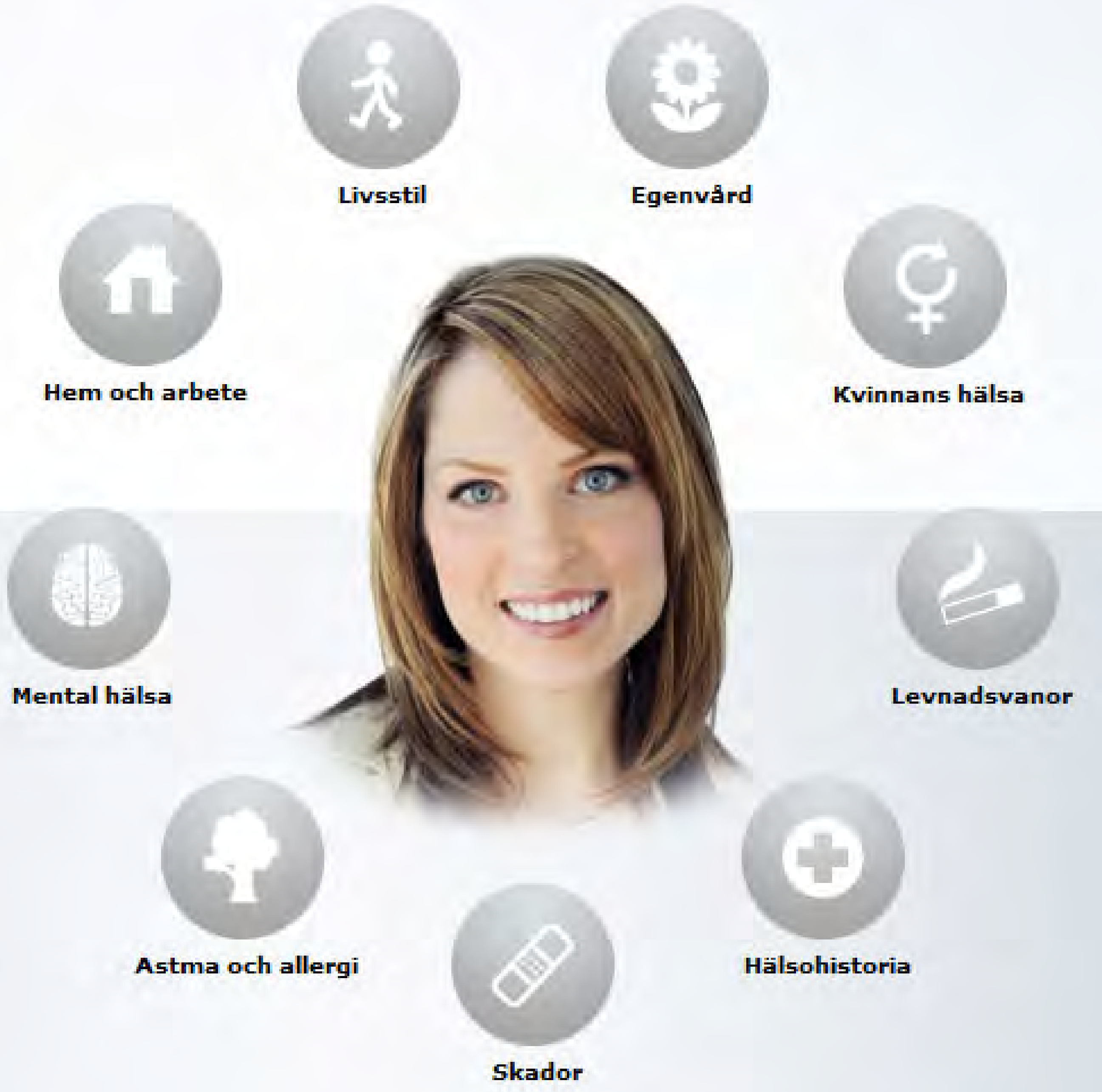

Questionnaire overview

The baseline survey is built up by instruments covering a vast range of the epidemiological research on physical, social and mental well-being by this date, and is designed for study participants in the age range from 0 to 45 at baseline. In total, the survey delivers 4.000 variables out of a bank of 1.300 questions for adults and 1.050 questions for parents and children.

The questionnaire is available to the study participants through a web portal designed as a circular clock-like menu with questionnaire themes on the dial. Eight or nine themes are shown to adults dependent on their sex, and four to nine themes are available to the parents and children. The parents answer for their children of age 0 to 14 and the children answer for themselves from age 11. This means that the survey system collects data from both parents and children in the ages from 11 to 14.

The survey themes are:

- Socio demography
- Lifestyle
- Self care
- Woman’s health (puberty for adolescence)
- Living habits
- Health history
- Injuries
- Asthma and allergies
- Mental health

Socio-demography

The socio economic and demographic questions collect information on socio economic belonging, family situation, and occupation. The socio demographic theme constitutes a general, an occupational and a work related module. The general part collects information about dwelling, living, civil status, family, descent, and education. Health and demographics differs by profession and greatly between those having a job and those who do not have a job. Civil status and family situation is related to mortality and health status, and health/social characteristic stratification is available on e.g. questions on descent. The occupational questions collect information about the present and the past 5 years of occupation, working shift, night work and unemployment. The working environment and the work related questions focus on physical discomforts, chemical exposures, work related psychological and social situations, discrimination, stress, overtime, sick-leave, sleeping problems and the balance between work and home. These questions therefore collect information about social support, psychological requirements and lack of control at work, which all are important factors behind environmental causes of illness, stress, morbidity and mortality.

Lifestyle

The lifestyle theme comprises a quality of life module along with modules on diet, physical activity, sexual behavior, and wireless devices. The first module studies health related quality of life. The quality of life module collects data of general health outcome as well as mental well-being and possible symptoms of stress, pain and fatigue. The EQ5D instrument is used to capture general health outcome in the five dimensions mobility, hygiene, daily activities, pain, and anxiety/depression. Self-rated health state is a well-known indicator of mortality. The quality of life module includes this overall quality of life scale along with the Diener satisfaction with life scale and the Lyubomirsky happiness scale. The stress section uses the Cohen Perceived Stress Scale, which complements the work related questions of stress located in the socio demography theme. This data renders further understanding of e.g. gender specific and socio economic related stress. The LifeGene pain section is based on The Brief Pain Inventory which captures everyday pain. BPI is available in several versions and asks in the different versions about pain in the last 24 hours, in the last week or in the last month. For the purposes of this cohort the month time span is used, which makes it feasible to also capture duration of pain within that time span. The next section in the quality of life module is about fatigue and is based on the Brief Fatigue Inventory instrument, which collects information about fatigue experiences in the last week time span.

The lifestyle theme also includes a module on diet, which is a further development of the standard FFQ (food frequency questionnaire) assessment method. The food items are here listed in the order they are consumed during the day – i.e. food items consumed at breakfast, lunch, dinner, and supper. This makes it more convenient for study participants to answer the questions. The study participants respond on questions on e.g. sugar and fat intake, fruit and fiber intake, dairy and caffeine intake, vitamin D, selenium and calcium intake. A validation study was performed to establish and fine-tune the instrument, where energy and nutrition intake was analyzed and compared with traditional assessment methods and golden standards. This instrument makes it possible to study essential relationship between food intake and e.g. obesity, energy intake, cardiovascular diseases, blood pressure, digestive problems, cholesterol levels, insulin sensitivity, type II diabetes, metabolic disturbances, bone health, weight loss, slowing memory loss, antioxidant capacity and prevention of certain forms of cancer.

Physical activity is known to resist obesity, cardiovascular diseases, high blood pressure, high blood fat, diabetes type II, colon cancer, and depression among others. Overall health outcome is also related to the level of physical activity during leisure time. In the inventory of possible candidates for physical activity assessments, we learned that there was no reasonable questionnaire available for directly asking the study participants what physical activities they perform, e.g. popular Swedish activities like power-walking. The newly developed LifeGene module now therefore directly asks about the most popular physical activities, and in-activities, concerning frequencies and durations of these during working hours, leisure time, transportation, and during sport. These measures are then readily available for e.g. traditional categorization of intensity ranges from in-activity through low and moderate to vigorous activities.

The LifeGene lifestyle theme includes a module about sexual behavior, which captures information related to Human Papilloma Virus, as well as sexual risk behavior and sexually transmitted diseases. A study of 40.000 Swedes was performed previously on the subject and the LifeGene module will provide further variables, e.g. on cervix vaccination, on the matter. One concern with this module was that the drop-off might be clearly distinguished compared to other modules. However, this was shown not to be the case.

The LifeGene survey also covers exposure to mobile phones, DECT phones and wireless computer devices. The duration of exposure, frequencies and body parts exposed is encountered for in this module. The questionnaire is aligned with large studies like UK Biobank.

Self-care

LifeGene has access to Swedish national medical prescription registers. However, they do not cover non-prescribed medication bought over the

counter at pharmacies. The self-care module therefore collects information about the most common non-prescribed substances used in Sweden, e.g. paracetamol, ibuprofen, diklofenak, and acetylsalicylic acid. The Swedish medical product agency now also includes complementary and alternative medical products on their list of approval, e.g. Omega-3, Esberitox, Bio-Biloba, and Gericomplex, which can be found in the self-care module. In 2001 Stockholms läns landsting started a study investigating 1,000 citizens in Stockholm about their experiences of complementary and alternative medicine. The study showed that the number of persons receiving treatment from at CAM practitioner, at one time or more often, more than doubled in the last 20 years. Nearly half of the study participants received such a treatment in year 2001. The self-care module provides data for further investigations by collecting information on frequency and duration on the most common CAM treatments and techniques, e.g. professional massage.

Woman's health

The woman's health theme asks women about their menstruation, contraceptives, pregnancy, giving birth, gynecological surgery, infertility, diseases, cervix vaccine, and incontinence. Older women are also asked about menopause. The menstruation section collects information about year of menarche, regularity in menstruation, years of use of contraceptives, and questions related to pain. The pregnancy / giving birth section asks questions about number of pregnancies, miscarriages, and giving births. The infertility section collects information about time to pregnancy and means to pregnancy. There is also a section on gynecological surgery asking questions about surgery of the cervix, uterus, ovary, sterilization, abortion, pregnancy complications, and other operations. A change in the hormonal system is an important factor in hormonal-related diseases and gene/environment studies. The disease section collects information about myoma, endometriosis, polycystic ovarian syndrome, infertility, and asks a question about vaccination against cervical cancer, the human papilloma virus. The menopause section gathers information about menopause age, symptoms, medication use, and osteoporosis. The incontinence section uses the instrument UDI-6 (Urinary Distress Inventory) and IC (Interstitial Cystitis Symptom and Problem Questionnaire) to collects information about difficulties controlling the urine bladder.

Living habits - tobacco, alcohol and drug use

Tobacco use is known to cause or worsen several kinds of cancer, cardiovascular diseases, lung diseases, and stomach ulcer, among others. The tobacco module gathers information about this asking about smoking

and use of snus among adults. It also collects information on exposure to passive smoking among children and parents. The smoking section captures onset age, package years, present smoking, nicotine dependency (Fagerström Test for Nicotine Dependency scale), serious quit attempts, and peer smoking. The snus section, which captures a kind of moist oral tobacco which is popular in Sweden, is analogous in setup.
Improper use of alcohol may lead to social problems, accidents, alcoholic intoxication, psychoses, gastritis, obesity, and eventually liver cirrhosis. In the first of four sections this module collects information about alcohol consumption on beer, wine, spirits, cider and pops. Weekday and weekend drinking is differentiated in the assessment. The second section is about alcoholic risk behavior. The instrument AUDIT (Alcohol Use Disorder Identification Test) developed by the World Health Organization, is used to capture binge drinking, intoxication, drink amount and frequencies. The third section is there for those who show signs of addiction. This section is consistent with the DSM-IV terms for addiction. The last section in this module captures heredity related drink abuse.
The drug module gathers information about the use of both licit and illicit drugs which are used for the purpose to get high, to feel better, to change mood, enhance performance or to grow muscles. The instrument DUDIT (Drug Use Disorder Identification Test), developed by the World Health Organization, captures information about onset age, present or previous use and how often and how much is used. The remaining questions in this module about addiction fulfill the corresponding DSM-IV terms.

Health history

The health history theme includes questions on dental, infection, diseases, eating disorder, reflux/IBS, headache, and hearing. The national survey HLV, administered by the Swedish National Institute of Public Health in 2004, showed that 11% of the population has poor dental health. Poor dental health is closely related to socio economic belonging and age. Dental health is essential to diet to digest and poor diet habits will affect the dental health as well. The dental module focuses on these issues including questions on last dentist visit, frequency of teeth brushing, use of dental floss and tooth paste, use of braces, dental fillings and periodontal disease. By combining the diet module with this dental module, it is possible to study the diet affects on the dental health as well.
The infection module captures information about tick bites in the last 12 months, fungal infection, pet exposure, and flu like illness. The flu like illness is pursued further in detail concerning how often this has been the case in the last 12 months, home sick-time, and use of medication. This module is correlated to the questionnaires, which were used in the LifeGene add-on study “flu-like illness”, started in September 2009.
The disease module is essentially a list of more than 60 of the most common Swedish diseases. The areas covered are heart and circulation,

pulmonary disease, skin, the endocrine system, urology, musculoskeletal, neurology, child diseases and psychiatry. The module also includes follow-on questions for diagnoses. Some of the diseases also have more detailed follow-on questions, e.g. questions about the location of a blood clot, kind of thyroid disease, kind of diabetes and treatment, type of epilepsy, symptoms/treatments of osteoarthritis, and anal incontinence. The instrument Cleveland Clinic Incontinence Score (CCIS) is used to capture anal incontinence.

The eating disorder module gathers information about Body Mass Index, anorexia, bulimia, binging, and self-cleaning. The instrument has previously been used in STAGE and it has been structured in LifeGene in close collaboration with the author. The instrument fulfills the DSM-IV criteria for each of the eating disorder conditions. The BMI value is also used for other purposes, like detecting obesity, which is a significant risk factor for poor general health, fatigue, pain, illness, and mortality.

The health history theme also includes a module on reflux and Irritable Bowel Syndrome. The IBS questions gather information about discomfort frequency, duration, onset age, and pain location and related discomfort experiences. The reflux section, about heart burn and reflux, asks questions about onset age, frequency, duration, night awakenings and use of medication. This instrument fulfills the Rome II criteria for these functional gastrointestinal disorders.

Sleep is essential to physical and mental recovery. Sleeping length and sleeping quality is known to be affected by stress, BMI, consumption of alcohol and shift working. Too little or too much sleep is also related to mortality, heart disease and diabetes. The sleep module covers these issues and captures information about sleeping length/problems, by starting off with a general question of sleep quality related to having children or a snoring partner. The module continues with questions about how much sleep is needed each night, if the person is a morning or evening type, actual bed and rise time on evenings before weekdays and before weekend days. There are also questions on snoring and apnea, use of means to sleep better, e.g. medication. Sleep apnea is occurring among men and women at all age groups. The sleeping problem section looks into several kinds of sleeping problems and it also covers the effects disturbed sleep has on day time activities. It is possible to combine the data about sleeping problems with data on perceived stress by combining sleep data with data in the quality of life module and the socio demography theme.

The headache module collects information about daily and recurrent headaches. The module follows up on symptoms and severity of tension headache, migraine, and cluster headaches. Data is also collected on the effects of every day quality, possible diagnoses, and use of medication. The instrument follows the International Headache Society recommendations.

The hearing module is based on the Swedish H70 questionnaire and collects information about estimated hearing capability, listening to loud

music, tinnitus, dizziness, use of hearing aid, and the problems with communication related to impaired hearing.

Injuries

The injury module collects incidence information on injuries being inflicted to the study participant. It also gathers information about long term deficiencies related to these injuries. This instrument is based on the Injury Mortality Diagnosis Matrix and asks questions about the cause of injury, what body part was injured and what kind of injury was inflicted, e.g. a superficial leg injury, a wound, dislocation, fracture, blood vessel, burn, frostbite or another kind of injury. There are extra questions for those who suffered an injury to the head, i.e. questions about experiences of confusion, loss of consciousness, amnesia, irritability, or headache. The injury module also includes a question if the injury was related to risk behavior. The module also asks questions about eventual long term effect from an injury, i.e. problems with seeing, hearing, memory, self-care, and problems with communication.

Asthma and allergy

The asthma and allergy theme includes modules on general exposures, asthma, Chronic Obstructive Pulmonary Disease, allergy and chronic sinusitis. The general exposures module asks questions about vacuum cleaning and floor washing in the home environment. Information is also collected on how often the mattress cover is washed, problems with window condensation (bad ventilation), stains in the home caused by moisture, mold or mildew, and use of gas stove.
The asthma module collects information about wheezing and in which circumstances wheezing occurs, e.g. during physical activity, having a cold etc. There are also questions on shortness of breath. People with asthma will receive detailed questions about onset age, diagnose, severity and use of medication. The COPD section is available for people who smokes and also for people over age 35. It collects information about coughing and typical bringing up phlegm. People with these symptoms will also get questions about the respiratory severity, e.g. problems walking in stairs.
The allergy and chronic sinusitis module collects information about nasal allergies, food allergies, other allergies, medication and chronic rhino sinusitis. The nasal allergy section asks questions about nasal allergy symptoms, which includes hay fever as well as non-allergic rhinitis. People with nasal allergies will receive further questions about the continuity of these problems and typical itchy eyes symptoms. The food allergy section asks questions about allergies with egg, fish/shellfish, peanuts, nuts (e.g. almond), pip or stone fruit (e.g. apple), grain, milk, and other kinds of food allergies. The food allergic section also collects information about the

allergy symptoms, e.g. itch, swollen lips, anaphylactic shock etc. In the following section the instrument gathers data and diagnoses on pollen, pet, mite, bee/wasp and contact allergies. People with any of these allergies receive questions about use of medication, nasal spray and antihistamine tablet like Nasonex, Rhinicort, Loratadin and Clarityn. The allergy module is finalized with questions on chronic rhino sinusitis. The study participant receives questions about having a stuffed nose, nasal discharge, facial pressure and reduction of smell, and whether a doctor has diagnosed these symptoms. The asthma and allergy instruments are based on instruments from the Barn Allergi Miljö Stockholm Epidemiologi (BAMSE), the European Community Respiratory Health Survey (ECRHS), and the International Study of Asthma and Allergies in Childhood (ISAAC).

Mental health

Neuropsychiatric conditions hold 35.6% of the total health burden in Sweden, in terms of disability adjusted life years. This area is vast and in order to be able to include these conditions we constructed top screeners. A top screener includes the basic questions from each neuropsychiatric condition, and by answering these questions the system will show follow-on modules triggered by the top screener. This makes it possible for the study participants to complete the mental health theme in less than 20% of the assessment time. This theme mainly uses the CIDI instrument (Composite International Diagnostic Interview), developed by the World Health Organization. The following two tables shows the neuropsychiatric disorders grouped by the most common conditions in Sweden and how the neuropsychiatric disorders are grouped by the disorders assessed in LifeGene.

**Neuropsychiatric condition**	**DALY* %**
Unipolar depressive disorders (major depression)	30.6
Alcohol use disorders	22.1
Alzheimer and other dementias	7.8
Self-inflicted injuries	7.4
Migraine	6.8
Schizophrenia	5.4
Bipolar disorder	5.3
Panic disorder	2.8
Drug use disorders	2.4
Insomnia (primary)	2.2
Obsessive-compulsive disorder	2.0
Epilepsy	1.7
Post-traumatic stress disorder	1.5
Multiple sclerosis	1.0

Parkinson disease 1.0

Sum **100**

* Disability-Adjusted Life Years. A notion defined by the World Health Organization describing the number of years lost due to ill-health, disability or early death. Above DALY shows the relative proportion within the neuropsychiatric area.

Neuropsychiatric assessment

Disorder	Age				Assessment Method		
	4 - 10	11 - 14	15 - 17	18+	Register	Single item	Module
**Psychotic Disorders**							
Schizophrenia	-	-	X	X	X	X	-
Schizoaffective disorder	-	-	X	X	X	X	-
**Mood**							
Bipolar disorder	-	-	X	X	X	X	-
Major depression	P	P & C	X	X	X	X	X
Suicidality / Self harm	P	P & C	X	X	X	X	X
Perinatal depression	-	-	-	PR	X	X	X
Premenstrual syndrome	-	-	F	F	X	X	X
**Anxiety disorders**							
Panic disorder	-	-	X	X	-	X	X
Agoraphobia	-	-	X	X	-	X	X
Generalized anxiety	P	P & C	X	X	-	X	-
OCD	-	-	X	X	-	X	X
PTSD	-	-	X	X	-	X	X
Social phobia	P	P & C	X	X	-	X	X
Specific phobia	-	-	X	X	-	X	X

**Psychoactive Substance Use Disorder**							
Tobacco use	-	C	X	X	-	-	X
Alcohol use	-	C	X	X	X	-	X
Other licit / illicit drugs	-	-	X	X	X	-	X
**Other Disorders**							
Insomnia (primary)	-	-	X	X	-	-	X
Eating disorders	P	P & C	X	X	X	-	X
Traumatic life events	-	-	X	X	-	X	X
Gambling	-	-	X	X	-	X	X
ADHD	P	P & C	X	X	X	X	X
Tics	P	P & C	X	X	-	X	X
**Neurology**							
Headache	P[1]	C	X	X	-	-	X
Epilepsy	-	-	X	X	X	X	-
Multiple sclerosis	-	-	X	X	X	X	-
Alzheimer's / dementias	-	-	-	X	X	X	-
Parkinson disease	-	-	-	X	X	X	-
Hearing loss	P	C[2]	X	X	X	-	X

1 Headache module from age 7

2 Hearing module from age 11

ADHD Attention-deficit hyperactivity disorder

C Children respond for themselves

F Females

Module A set of follow-on questions given to the respondent if the corresponding OCD Obsessive-compulsive disorder

P Parents respond for their children

P & C Parents respond for their children and the children respond for themselves

PR Females who is pregnant or have been pregnant

PTSD Post-traumatic stress disorder

Register Swedish national registers

Screener A few top level questions on the subject

screening questions were answered positively

The perinatal disorder instrument collects information on depression related to pregnancy and giving birth and it is based on the Edinburgh perinatal module. The PSST (Premenstrual Symptoms Screening Tool) instrument captures premenstrual dysphoric disorder and is designed to quantify the DSM-IV categorical criteria into rating scales and degrees of severity. Description on the tobacco, alcohol and drug use disorders is available in the living habits module. The traumatic life event disorder section is based on the LSC-R (Life Stressor Checklist-Revised) instrument. The gambling section collects information about how often people gambles and the amount of money spent in typical Swedish gambling. People with signs of gambling dependency will also get a few questions on that subject. The instrument SOGS-R (South Oak Gambling Screen Revised) is used to collect information about gambling habits and the CPGI (Canadian Problem Gambling Index) instrument collects information about gambling dependency. The mental health theme also collects information about ADHD using the ASRS instrument (Adult ADHD Self-Report Scale), developed by the World Health Organization.
There are also questions about personality for adults in the mental health theme and the instrument Eysenck Personality Questionnaire is used for this purpose. Parents to children in the age 0 to 3 will receive questions about infant and toddler socio-emotional behavior, delivered by the IBQ-SF (Infant Behavior Questionnaire Short Form) and BITSEA (Brief Infant and Toddler Socio Emotional Assessment) instruments. Parents to children in the ages from 4 to 14, and children from age 11, will receive questions about family environment and their development and well-being. The family environment questions are delivered by the FES (Family Environment Scale) instrument and the DAWBA instrument (Development and Well-Being Assessment).

LifeGene

Instrument definition

Adult

Theme, module and section	Instrument	Source
**Socio-demography**	LG	STAGE
General		
Living		
Family / household		
Descent		
Education		
Occupation		
Work history		
Working shift		
Working nights		
Unemployment		
Working environment		
Physiological discomfort / chemical exposures		
Psychological discomfort / social situations		
Stress at work		
Sick-leave		
Sleep, private life		
**Lifestyle**		
General		
General health	EQ-5D	
Life satisfaction and happiness	D&L	
Overall health state	LG	
Stress	PSS-10	
Pain	LG	BPI
Fatigue	LG	BFI
Diet	LG	KB
Meals		
Beverages		
Food / groceries		
Dishes		
Potatoes, pasta, vegetables, and sauce		
Portion sizes		
Fat and salt		
Food supplements		
Physical activity	LG	KB
Activity during occupational hours		
Transport to occupation		
Leisure time activities		
Sport activities		
Sexual behavior	LG	HPV
General		
Sexually transmitted diseases		

Wireless devices	LG	UK Biobank
Mobile telephone		
DECT phone		
Wireless computer devices		
**Self-care**		
Medication OTC	LG	
Complementary and alternative medicine	LG	ML
Products		
Treatments		
Techniques		
**Woman's health**		
Menstruation / contraceptives	LG	STAGE
Pregnancy / giving birth	LG	STAGE
Gynecological surgery	LG	STAGE
Infertility / diseases / vaccine	LG	STAGE
Menopause / osteoporosis	LG	
Incontinence	UDI-6, CCIS	
**Living habits**		
Smoking		
Smoking history	LG	STAGE
Smoking dependency	FTND	
Present smoking and quitting attempts	LG	STAGE
Snus		
Snus use history	LG	STAGE
Snus dependency	FTND	
Present snus use and quitting attempts	LG	STAGE
Peers	LG	STAGE
Alcohol		
Alcohol onset	LG	STAGE
Present alcohol use	LG	STAGE
Alcohol risk behavior	AUDIT	
Alcohol addiction	LG	STAGE
Alcohol and heredity	LG	STAGE
Friends	LG	STAGE
Drug use	LG	DUDIT
Present or previous drug use		
Drug addiction		
**Medical history**		
Dental	LG	STAGE
Oral health		
Fillings and dental disease		
Infections	LG	MM
Common infections		
Sick-leave and medication		
Diseases	LG	STAGE, CCIS
Eating disorder	LG	STAGE, CB
Introductory questions		
Weight and body shape		
Anorexia		
Binging		
Purging		
Reflux, IBS	LG	STAGE

Sleep	LG	STAGE
Sleeping quality and sleeping time		
Sleeping difficulties, snoring, apnea		
Means to sleep better		
Sleep disturbance		
Headache	LG	STAGE
Daily or recurrent headaches		
Headache symptoms		
Quality of life, diagnose, medication		
Hearing	LG	H70c6
Hearing self-estimation		
Use of earphones		
Tinnitus		
Dizziness		
Hearing impairment		
**Injuries**	LG	KM, IMD
Last year injuries		
Injury cause		
Body parts inflicted		
Medical attention due to injury		
Injury long-term effects		
Risk behavior		
**Asthma and allergy**		
General exposures	LG	BAMSE
Floors and bed		
Ventilation		
Mold and use of gas stove		
Asthma	LG	ECRHS, ISAAC
Wheezing or whistling in the chest		
Attack of shortness of breath		
Asthma		
Hospital visit		
Medication		
COPD	LG	ECRHS
Cough and phlegm		
Shortness of breath		
Allergy and chronic sinusitis	LG	ECRHS, ISAAC
Nasal allergies and non-allergic rhinitis		
Food allergies		
Other allergies		
Medication		
Chronic sinusitis		
**Mental health screener and personality questions**		
Personality questions	Eysenck, AHE	
Screening questions		
Depression	LG	STAGE
Perinatal depression	LG	EPDS
Premenstrual symptoms	LG	PSST
Suicidality (incl. follow-on questions)	LG	CIDI
General anxiety disorder	LG	CIDI
Panic disorder	LG	CIDI

Agoraphobia	LG	CIDI
Obsessive compulsive disorder (OCD)	LG	CIDI
Post Traumatic Stress Disorder	LG	CIDI
Social phobia	LG	CIDI
Specific phobia (incl. follow-on)	LG	CIDI
ADHD (incl. follow-on)	LG	ASRS
Traumatic life events	LG	LSC-R
Chronic fatigue syndrome (incl. follow-on)	LG	STAGE
Gambling (incl. follow-on)	LG	SOGS-R, CGPI
Tourette's syndrome (incl. follow-on)	LG	STAGE
**Mental health follow-on questions**		
Depression	LG	STAGE
Perinatal depression	LG	EPDS
Premenstrual symptom	LG	PSST
Panic disorder	LG	CIDI
Agoraphobia	LG	CIDI
Obsessive compulsive disorder	LG	CIDI
Post traumatic stress disorder	LG	CIDI
Social phobia	LG	CIDI

AHE	Swedish Twin study of Aging, Health, and Environment, part 2
ASRS	Adult ADHD Self-Report Scale
BAMSE	Child study at IMM KI
BFI	Brief Fatigue Inventory
BPI	Brief Pain Inventory
CB	Cynthia Bulik, North Carolina University, USA
CCIS	Cleveland Clinic Incontinence Score
CGPI	Canadian Gambling Problem Index
CIDI	Composite International Diagnostic Interview
D&L	Items from the Diener and Lyubomirsky scales
DUDIT	Drug Use Disorder Identification Test
ECRHS	European Community Respiratory Health Survey
EPDS	Edinburgh Perinatal Depression Scale
H70	The Gothenburg population based study, cohort 6
HPV	undersökning om inställningen till ett nytt vaccin, MEB KI
IC	Interstitial Cystitis Symptom and Problem Questionnaire
ISAAC	International Study of Asthma and Allergies in Childhood
IMD	Injury Mortality Diagnosis (part of ICD-10)
KB	Katarina Bälter, MEB KI
KM	Karl Michaelsson, UCR, Uppsala University
LG	LifeGene
LSC-R	Life Stressor Check List – Revised
ML	Mats Lekander, CNS KI
MM	Markus Maeurer, MTC KI
PSS-10	Perceived Stress Scale 10
PSST	Premenstrual Symptoms Screening Tool
SOGS-R	South Oaks Gambling Screen – Revised

LifeGene

STAGE Swedish Twin Gene and Environment study
UDI-6 Urinary Distress Inventory

Child / parent

Theme, module, and section	Age 0-3	4-6	7-10	11-14	15-17	Instrument	Source
**Socio-demography**							
Household structure	x	x	x	x	x	LG	LG
**Lifestyle**							
Daycare and school	x	x	x	x	x	LG	LG
Cleanliness	x	x	x			LG	CA
Sun	x	x	x	x	x	LG	LG
Diet	x	x	x	x	x	LG	KB
Physical activity	x	x	x	x	x	LG	KB
Sexual behavior					x	LG	LG
Wireless devices	x	x	x	x	x	LG	LG
Pets	x	x	x	x	x	LG	ECRHS
**Self-care**							
Medication over the	x	x	x	x	x	LG	LG
counter			x	x	x	LG	ML
Alternative medical							
products,							
treatments, techniques							
**Puberty and woman's health**							
Puberty			xA	x	x	LG	PDS
Woman's health					x	LG	
**Living habits (tobacco, alcohol and drug use)**							
Smoking (passive)	x	x	x	x	x	LG	LG
Snus				x	x	LG	LG
Alcohol				x	x	LG	CP
Drug use							DUDIT
**Medical history**							
Dental		x	x	x	x	LG	LG
Infections	x	x	x	x	x	LG	LG
Diseases	x	x	x	x	x	LG	LG
Health control	x	x	x	x	x	LG	LG
Vaccination	x	x	x	x	x	LG	CA
Sleep	x	x	x	x	x	LG	LG
Pacifier							
Nap							
Sleeping time							
Sleeping problems							
Headache				x	x	LG	LG
Hearing				x	x	LG	LG
**Injuries**	x	x	x	x	x	LG	LG

**Asthma and allergy**							
Asthma	x	x	x	x	x	LG	ECRHS
Allergy	x	x	x	x	x	LG	ECRHS
Eczema	x	x	x	x	x	LG	ECRHS
**Mental health**							
Infant behavior	xB					IBQ	IBQ
Infant toddler socio-emotional questions	xC					BITSEA	BITSEA
Parenting	x	x	x	x	x	APQ	APQ
Family environment	x	x	x	x	x	FES	FES
Screening questions		x	x	x	x	DAWBA	DAWBA
Strengths and difficulties							
Social Aptitudes Scale							
Development							
Separation anxiety							
Social phobia							
Generalized anxiety							
Depression							
Attention / hyper-activity							
Troublesome behavior							
Diet, weight and body shape							
Tics							
Friendship							
Follow-on questions		x	x	x	x	DAWBA	DAWBA
Development							
Strengths and difficulties							
Social Aptitudes Scale							
Development							
Separation anxiety							
Social phobia							
Generalized anxiety							
Depression							
Attention / hyper-activity							
Troublesome behavior							
Diet, weight and body shape							
Tics							
Friendship							

BAMSE	Child study at IMM KI
CA	Catarina Almqvist, MEB KI
CIDI	Composite International Diagnostic Interview
DAWBA	Development and Well-Being Assessment
ECRHS	European Community Respiratory Health Survey
H70	The Gothenburg population based study, cohort 6
IMD	Injury Mortality Diagnosis (part of ICD-10)

ISAAC	International Study of Asthma and Allergies in Childhood
KB	Katarina Bälter, MEB KI
KM	Karl Michaelsson, UCR, Uppsala University
LG	LifeGene
ML	Mats Lekander, CNS KI
MM	Markus Maeurer, MTC KI
PDS	Puberty Development Scale
STAGE	Swedish Twin Gene and Environment study
xA	Puberty for parents at age 10
xB	Infant behavior from age 0 to 12 months
xC	Infant Toddler Socio-Emotional questions from age 1 to 3

Domains distributed to the study participants

Adults

Age 18+: socio-demography, quality of life, wireless devices, self-care, woman's health, smoking, snus usage, alcohol, illicit drugs, infection, diseases, eating disorder, reflux, irritable bowel syndrome, sleep, hearing, injuries, asthma, allergy, mental health screening questions, major depression, perinatal depression, premenstrual syndrome, panic disorder / agoraphobia, obsessive compulsive disorder, post-traumatic stress disorder, social phobia.

Parents

Age 1-3: socio-demography, self-care, smoking, illness, sleep, injuries, asthma, allergy, eczema, infant behavior questionnaire.
Age 4:-9: socio-demography, self-care, smoking, infection, illness, sleep, injuries, asthma, allergy, eczema, development and well-being assessment.
Age 10: socio-demography, self-care, puberty, smoking, infection, illness, sleep, injuries, asthma, allergy, eczema, development and well-being assessment.
Age 11-14: self-care, infections, diseases, sleep, injuries, asthma, allergy, eczema, development and well-being assessment.

Children

Age 11-14: socio-demography, wireless devices, puberty, smoking, snus usage, alcohol, development and well-being assessment.

Age 15-17: self-care, puberty, smoking, snus usage, alcohol, illicit drugs, infection, diseases, sleep, hearing, injuries, asthma, allergy, eczema, mental health screening questions, major depression, premenstrual syndrome, panic disorder / agoraphobia, obsessive compulsive disorder, post-traumatic stress disorder, social phobia.

LifeGene

General variables

[started_time]
UTC time for when the study participant entered the questionnaire.

[reentered_time]
UTC time for when the study participant re-entered the questionnaire.

[finished_time]
UTC time for when the study participant completed the questionnaire

[answered_by]
Whether an adult, parent or child responded to the questionnaire

[sex]
The sex of the study participant

[age]
Age of study participant when entering the questionnaire.

[responseorder]
The order the study participant chose to respond to the domains of questions.

[survey_version]
Version of the survey used to collect data for each study participant.

[region]
First number in zip code.

Reading the metadata

The questionnaire elements, i.e. the questions, are data about the data actually collected by the survey system, i.e. the metadata. On the highest level the metadata is structured in the following epidemiological themes.

**Themes**
Socioeconomic/demographic characteristics
Physical Environment
Perception of Health
Medication
Women's Health
Reproductive History
Life Habits/Behaviours
Individual Disease History
Familial Disease History
Individual History of Injuries
Mental Health

The theme elements are domains, which constitute the second highest level of the metadata. The domain names are actually part of the variable names with a three-letter pre-extension. E.g. "soc" is for socio-demography grouping all variables (questions) on the socio-demographic subject within one unified local "socio-demographic" domain name space. All domain names together define the full name space of the questionnaire.

Domains

Name	Description
soc	Socio-economy/socio-demographics
qua	Quality of life
die	Diet
phy	Physical activity
sex	Sexual habits
wir	Wireless devices
sel	Self-care
wom	Women’s health
smo	Smoking
snu	Snus use
alc	Alcohol

ill	Illicit drugs
den	Dental health
inf	Infections
dis	Diseasesdiseases
eat	Eating disorder
ref	Reflux
ibs	Irritable Bowel Syndrome
sle	Sleep
hea	Headache
her	Hearing
inj	Injuries
exp	Exposures
ast	Asthma
cop	Chronic Obstructive Pulmonary Disease
all	Allergy
per	Personlity
pms	Premenstrual syndrome
dep	Depression
pan	Panic disorder
ago	Agoraphobia
pts	Post Traumatic Stress Disorder
soi	Social Phobia
cle	Cleanliness
sun	Sun bathing
pet	Pets
pub	Puberty
con	Health check (control)
vac	Vaccination
ecz	Eczema
ibq	The instrument Infant Behaviour Questionnaire
bit	The instrument Brief Infant Toddler Socio-Emotional Assessment
apq	The instrument Alabama Parenting Questionnaire
fes	The instrument Family Environmental Scale
daw	The instrument Development And Well-Being Assessment

Questionnaire items and variable names

a) Below questionnaire element shows a question with a single answer option. The corresponding variable in the dataset is "soc_dwelling" having the predefined answer options "1", "2", "3", "4", and "998".

[soc_dwelling]
SC10. In what sort of dwelling do you live?

❍ Villa / independent house [1]
❍ Apartment [2]
❍ Terrace houses / chain houses [3]
❍ Other [4]
❍ Don't know / refuse [998]

b) Below questionnaire element shows a question with a multi answer option also including open text fields. The corresponding variables in the dataset are:

Dataset variable	values
soc_sibling_full	0, 1
soc_sibling_half	0, 1
soc_sibling_oth	0, 1
soc_sibling_no	0, 1
soc_sibling_998	Free text
soc_sibling_full_other	Free text
soc_sibling_halv_other	Free text
soc_sibling_oth_other	Free text

[soc_sibling]

SC60. Do you have siblings?

Yes
❑ Full siblings (number) [full]
_____________ [full_other]

❑ Half siblings (number) [half]
_____________ [half_other]

❑ Other siblings, e.g. step siblings____ [oth_other]

❍ No [no]

❍ Don't know / refuse [998]

c) In below questionnaire element there is a question with grid answer options also having open text fields. The corresponding variables in the dataset are:

Dataset variable	value
soc_wrkpresent_pres_occ	Free text
soc_wrkpresent_pres_plc	Free text
soc_wrkpresent_pres_dur	Free text
soc_wrkpresent_pres_1	0, 1
soc_wrkpresent_pres_2	0, 1

[soc_wrkpresent]

SC115. What is your present occupation and place of work?

	Occupation (e.g. engineer, nurse) [occ]	Place of work (e.g. office, hospital) [plc]	Worked number of years [dur]	Full-time Part-time [int]	
				[1]	[2]
[pres]	______	______	______	☐	☐

Variable extensions

Extension is a central notion in the metadata design used to build up the name space in the LifeGene questionnaire data. Besides the domain pre-extensions described above there are also in- and post-extensions. E.g. "eat_weight_kg" is the name for a question concerning weight measured in kilogram in the eating disorder domain of questions. The most common extensions are:

Extension	Meaning
cm	Centimeter
kg	Kilogram
cl, 33cl, 50cl	Centiliter
frq	Frequency
dur	Duration
tms	Times
nbr	Number
date	Date
yrs, yr	Years
mnts, mnt	Months
wks, wk	Weeks
days, day	Days
hrs	Hours

min	Minutes
sec	Seconds
whc	Which
amnt	Amount
oth, other	Other
sum	sum
isoxxx	ISO-code
icdxxx	ICD-code
dsmxxx	DSM-code

Variable values - answer options

The answer options, i.e. variable values, are free text or predefined values. The most common predefined values for the variables in the questionnaire dataset are:

Precode	Meaning
1	Yes
2	No
1	Male
2	Female
1	Checkbox checked in a multi answer option question
0	Checkbox not checked in a multi answer option question, but shown to the study participant
999	Refuse to answer
998	Don't know / refuse
997	Don't know
996	None of these. Multi answer option questions often also have this answer option so the study participant can choose to say that none of the other answer options applies.
995	Question has been shown to the study participant, which chose not to respond to the question.
null	The variable does not have a value registered, e.g. a questions in the survey which was not answered. "null" is the symbolic expression for a missing value.

Sociodemografi

Innehåll

Innehåll ... 1

Allmänna frågor ... 2

Boende ... 2

Familj / hushåll ... 2

Härkomst ... 4

Utbildning ... 5

Yrke / sysselsättning ... 6

Yrkeshistorik ... 6

Skiftarbete ... 8

Nattarbete ... 9

Arbetslöshet ... 10

Arbetsmiljö ... 12

Fysiologiska besvär / kemisk exponering ... 12

Psykologiska besvär / sociala situationer ... 15

Arbetsrelaterad stress ... 17

Sjukskrivning ... 19

Sömn och privatliv ... 21

Allmänna frågor

Boende

[soc_dwelling]

SC10. Hur bor du?

❍ Villa [1]
❍ Lägenhet [2]
❍ Radhus / kedjehus [3]
❍ Annat [4]
❍ Vet ej / vill ej svara [998]

[soc_dwelling_own]

SC11. Äger du din bostad?

❍ Ja [1]
❍ Nej [2]
❍ Vet ej / vill ej svara [998]

[soc_dwelling_rooms]

SC20. Hur många rum har din bostad (utöver kök)?

❍ (Antal) [nbr] ____________[nbr_other] ❍ Vet ej / vill ej svara [998]

[soc_dwelling_area]

SC30. Hur stor är din bostad räknat i kvadratmeter?

❍ Storlek (m2) [sqm] ____________[sqm_other] ❍ Vet ej / vill ej svara [998]

Familj / hushåll

[soc_livetogether]

SC40. Bor du tillsammans med någon?

Ja
❑ Man/fru/fästman/fästmö/partner/sambo [hus]
❑ Barn (antal) [chi] ____________[chi_other]
❑ Syskon (antal) [sib]____________ [sib_other]
❑ Föräldrar [par]
❑ Vänner (antal) [fri] ____________[fri_other]
❑ Andra (antal) [oth] ____________[oth_other]
❑
❍ Nej [no]
❍ Vet ej / vill ej svara [998]

[soc_civilstatus]

SC50. Vilket är ditt nuvarande civilstånd?

❍ Gift [1]
❍ Sambo [2]
❍ Singel [3]
❍ Separerad / skild [4]
❍ Särbo [5]
❍ Änka / änkling [6]
❍ Vet ej / vill ej svara [998]

[soc_sibling]

SC60. Har du några syskon?

Ja
❑ Helsyskon (antal) [full] ____________[full_other]
❑ Halvsyskon (antal) [half] ____________[half_other]
❑ Andra syskon, t.ex. styvsyskon____[oth_other]
❍ Nej [no]
❍ Vet ej / vill ej svara [998]

[soc_children]

SC61. Har du biologiska barn?

❍ Ja [1]
❍ Nej [2]
❍ Vet ej / vill ej svara [998]

Om “ja”, visa SC62

[soc_children_whc]

SC62. Hur många biologiska barn har du?

❍ (antal) [nbr] ___________[nbr_other] ❍ Vet ej / vill ej svara [998]

Härkomst

[soc_born]

SC63. Är du född i Sverige?

❍ Ja [1] ❍ Vet ej / vill ej svara [998]
❍ Nej (vänligen ange ditt födelseland) [2] _____[2_other]

Om SC63 är ”nej”, visa SC64

[soc_tosweden]

SC64. När flyttade du till Sverige?

❍ Mindre än 1 år sedan [1] ❍ Vet ej / vill ej svara [998]
❍ 1 till 5 år sedan [2]
❍ 6 till 10 år sedan [3]
❍ 11 till 20 år sedan [4]
❍ Mer än 20 år sedan [5]

[soc_born_mother]

SC65. Är din biologiska mamma född i Sverige?

❍ Ja [1] ❍ Vet ej / vill ej svara [998]

❍ Nej [2]

Om SC65 är “nej”, visa SC66

[soc_born_mother_country]

SC66. I vilket land är din biologiska mamma född?

❍ “Nationslista enligt ISO-3166-2”

❍ Vet ej / vill ej svara [998]

[soc_born_father]

SC67. Är din biologiska pappa född i Sverige?

❍ Ja [1]   ❍ Vet ej / vill ej svara [998]

❍ Nej [2]

Om SC67 är "nej", visa SC68

[soc_born_father_country]

SC68. I vilket land är din biologiska pappa född?

❍ "Nationslista enligt ISO-3166-2"

❍ Vet ej / vill ej svara [998]

Utbildning

[soc_education]

SC70. Vilken är den högsta utbildningsnivå du har uppnått eller studerar på för närvarande?

❍ Nioårig grundskola [1]
❍ Gymnasium [2]
❍ Universitet [3]
❍ Annan [4]
❍ Vet ej / vill ej svara [998]

Yrke / sysselsättning

Yrkeshistorik

[soc_gainfemployed]

SC80. Har du någonsin förvärvsarbetat deltid eller heltid?

Ja
❍ 0 till 2 år [1]
❍ 3 till 5 år [2]
❍ 6 till 10 år [3]
❍ 11 till 20 år [4]
❍ Mer än 20 år [5]
❍
❍ Nej [no]
❍ Vet ej / vill ej svara [998]

❖ **Z. LÄMNA MODULEN OM SC80 ÄR ”NEJ”**

Om “Ja”, visa SC90

[soc_employer]

SC90. Hur många arbetsgivare har du arbetat för under denna tid?

❍ 1 [1]
❍ 2 [2]
❍ 3 till 5 [3]
❍ 6 till 10 [4]
❍ Mer än 10 [5]
❍ Vet ej / vill ej svara [998]

[soc_presentsit]

SC100. Vilket av följande alternativ beskriver din nuvarande situation bäst?

❍ Anställd [1]
❍ Arbetslös [2]
❍ Driver eget företag / arbetar som delägare i företag [3]
❍ Ålderspensionär [4]
❍ Aktivitets- eller sjukersättning (förtidspension) p.g.a. sjukdom / funktionshinder [5]
❍ Sjukskriven (sedan 2 månader eller längre) [6]
❍ Föräldraledig (sedan 2 månader eller längre) [7]
❍ Studerande [8]
❍ Tjänstledig [9]
❍ Hemmafru/ -man [10]
❍ Annat [11]
❍ Vet ej / vill ej svara [998]

Om SC100 inte är "anställd" eller "driver eget företag" eller "arbetar som delägare i företag", visa SC110

[soc_emp5year]

SC110. Har du förvärvsarbetat eller drivit eget företag under de senaste 5 åren?

❍ Ja [1]   ❍ Vet ej / vill ej svara [998]
❍ Nej [2]

❖ **A. OM [SC100 ÄR "ANSTÄLLD" ELLER "DRIVER EGET FÖRETAG ELLER ARBETAR SOM DELÄGARE I FÖRETAG"] ELLER SC110 ÄR "JA" FORTSÄTT MED NEDANSTÅENDE FRÅGOR**

Om SC100 är "anställd" eller "driver eget företag eller arbetar som delägare i företag", visa SC115

[soc_wrkpresent]

SC115. Vilket är ditt nuvarande yrke och typ av arbetsplats?

Yrke (t.ex. ingenjör, sjuksköterska) [occ]	Arbetsplats (t.ex. kontor, sjukhus) [plc]	Arbetat antal år [dur]	Heltid/ deltid [int]	
______	______	______	☐	☐

Om SC110 är "ja" eller SC115 är "2 år eller mindre" eller "deltid", visa SC120

[soc_wrk5year]

SC120. Vilket huvudsakliga yrke och typ av arbetsplats har du haft de senaste 5 åren?

Yrke (t.ex. ingenjör, sjuksköterska) [occ]	Arbetsplats (t.ex. kontor, sjukhus) [plc]	Arbetat antal år [dur]	Heltid/ deltid [int]	
______	______	______	☐	☐

❖ **A. VILLKORET AVSLUTAS**

Skiftarbete

[soc_shiftwork]

SC130. Har du någonsin arbetat skift?

❍ Ja, jag gör det för närvarande [1]
❍ Ja, jag har gjort det tidigare [2]
❍ Nej [3]
❍ Vet ej / vill ej svara [998]

Om SC130 är "ja..." visa SC140

[soc_shiftwork_dur]

SC140. I hur många år {har du arbetat, arbetade du} skift?

❍ Mindre än 1 år [1]
❍ 1 till 2 år [2]
❍ 3 till 5 år [3]
❍ 6 till 10 år [4]
❍ Mer än 10 år [5]
❍ Vet ej / vill ej svara [998]

Nattarbete

[soc_wrknight]

SC150. Har du någonsin arbetat natt (dvs. arbete mellan kl. 24.00 – 05.00)?

❍ Ja, jag gör det för närvarande [1]
❍ Ja, jag har gjort det tidigare [2]
❍ Nej [3]
❍ Vet ej / vill ej svara [998]

Om SC150 är "ja" visa SC160 till SC172

[soc_wrknight_dur]

SC160. I hur många år {har du arbetat, arbetade du} natt?

❍ Mindre än 1 år [1]
❍ 1 till 2 år [2]
❍ 3 till 5 år [3]
❍ 6 till 10 år [4]
❍ 11 till 20 år [5]
❍ Mer än 20 år [6]
❍ Vet ej / vill ej svara [998]

[soc_wrknight_frq]

SC170. Hur många gånger per månad {arbetar, arbetade} du natt?

❍ 1 natt eller mindre [1]
❍ 2 till 3 nätter [2]
❍ 4 till 6 nätter [3]
❍ 7 till 10 nätter [4]
❍ Mer än 10 nätter [5]
❍ Vet ej / vill ej svara [998]

[soc_wrknight_sleep_day]

SC171. Hur {är, var} din "dagsömn" vanligtvis efter ett nattskift?

	1	2	3	4	5	
Mycket dålig	☐	☐	☐	☐	☐	Mycket bra

[soc_wrknight_manage_1]

SC172. Hur {klarar, klarade} du att arbeta natt?

	1	2	3	4	5	
Ingen svårighet	☐	☐	☐	☐	☐	Mycket stora svårigheter

Om SC150 är "ja, tidigare", visa SC175 och SC177

[soc_wrknight_stop]

SC175. Vilket år slutade du arbeta natt?

❍ (Årtal, fyra siffror) [yr]____________ [yr_other] ❍ Vet ej / vill ej svara [998]

[soc_wrknight_stop_reason]

SC177. Varför slutade du att arbeta natt?

❍ Det var för tuff arbetstid [1]
❍ Av annat skäl [2]
❍ Vet ej / vill ej svara [998]

Arbetslöshet

[soc_unempl_bef]

SC180. Har du någonsin varit arbetslös?

❍ Ja [1] ❍ Vet ej / vill ej svara [998]
❍ Nej [2]

Om “ja” visa SC190, SC200 och SC210

[soc_unempl_sev]

SC190. Har du varit arbetslös flera gånger?

❍ Ja [1] ❍ Vet ej / vill ej svara [998]
❍ Nej [2]

[soc_unempl_start]

SC200. Vilket år blev du arbetslös {, senaste gången}?

❍ (Årtal, fyra siffror) [yr]____________ [yr_other] ❍ Vet ej / vill ej svara

[soc_unempl_dur]

SC210. Hur länge var du arbetslös {, senast}?

❍ (Antal år) [yrs] _________[yrs_other] ❍ (antal månader) [mnt] ____________[mnt_other]
❍ Vet ej / vill ej svara [998]

Arbetsmiljö

Fysiologiska besvär / kemisk exponering

[soc_wrkexper1]

SC220. Har du under de senaste 5 åren under en längre tid haft besvär beroende på något av följande på jobbet?

Ja
❑ Arbete med dator [dis]
❑ Obekväm arbetsställning [wrk]
❑ Tungt kroppsarbete [man]
❑ Värme, kyla, drag [hea]
❑ Buller [lou]
❑ Kemikalier [che]
❑ Vibrationer [vib]
❑
❍ Nej [no]
❍ Vet ej / vill ej svara [998]

❖ **B. FÖR VARJE MARKERAT SVAR I SC220 VISA NEDANSTÅENDE FRÅGOR**

[soc_wrkexper2_yr]

SC240. Hur länge har du besvärats av {markerad fråga} på arbetet:

❍ Mindre än 1 år [1]
❍ 1 till 2 år [2]
❍ 3 till 5 år [3]
❍ 6 till 10 år [4]
❍ Mer än 10 år [5]
❍ Vet ej / vill ej svara [998]

[soc_wrkexper2y]

SC250. Hur ofta har du besvärats av {markerad fråga} på arbetet:

❍ Ofta [1]
❍ Ibland [2]
❍ Sällan [3]
❍ Vet ej / vill ej svara [998]

[soc_wrkexperhard1x]

SC260. Har {markerad fråga} försvårat ditt arbete?

❍ Ja [1] ❍ Vet ej / vill ej svara [998]
❍ Nej [2]

Om "buller" markerats, visa också SC270

[soc_loudnoise]

SC270. Använder du hörselskydd mot bullret?

❍ Alltid [1] ❍ Sällan [3]
❍ Ibland [2] ❍ Aldrig [4]
❍ Vet ej / vill ej svara [998]

❖ **B. VILLKORET AVSLUTAS**

[soc_wrknoise]

Störs av buller på arbetet.

SC122. Störs du av buller på din arbetsplats för närvarande?

❍ Inte alls [1]
❍ Något [2]
❍ Ganska mycket [3]
❍ Mycket [4]
❍ Oerhört mycket [5]
❍ Vet ej / vill ej svara [998]

[soc_wrknoise_loud]

Höga bullernivåer på arbete hindrar samtal 1/4 av tiden.

SC121. Är du utsatt eller har du varit utsatt för höga bullernivåer i arbetet, så att du inte kan samtala i normal samtalston under minst 1/4 av arbetstiden?

❍ Ja [1]   ❍ Vet ej / vill ej svara [998]
❍ Nej [2]

[soc_wrknoise_raisev]

Höja rösten p.g.a. hög ljudnivå på arbetet.

SC123. Hur ofta utsätts du i ditt arbete för så höga ljudnivåer att du måste höja rösten för att kunna prata med andra människor?

❍ Aldrig / nästan aldrig [1]
❍ Omkring 25% av tiden [2]
❍ Omkring 50% av tiden [3]
❍ Omkring 75% av tiden [4]
❍ Alltid / nästan alltid [5]
❍ Vet ej / vill ej svara [998]

Om SC123 är ”omkring…” eller ”alltid…”, visa SC124

[soc_wrknoise_shout]

Höja rösten kraftigt p.g.a. hög ljudnivå på arbetet.

SC124. Hur ofta exponeras du för så höga ljudnivåer att du måste höja rösten kraftigt och skrika för att kunna prata med andra människor?

❍ Aldrig / nästan aldrig [1]
❍ Omkring 25% av tiden [2]
❍ Omkring 50% av tiden [3]
❍ Omkring 75% av tiden [4]
❍ Alltid / nästan alltid [5]
❍ Vet ej / vill ej svara [998]

[soc_wrknoise_protection]

Hörselskydd på arbetet.

SC125. Hur ofta använder du hörselskydd på ditt arbete?

❍ Aldrig [1]
❍ Ibland [2]
❍ Ofta [3]

❍ Alltid [4]
❍ Vet ej / vill ej svara [998]

Psykologiska besvär / sociala situationer

[soc_wrkexper2x]

SC280. Har du utsatts för något av följande på arbetet?

Ja
❑ Mobbning [bul]
❑ Diskriminering [dis]
❑ Trakasserier [har]
❑ Hot eller våld [thr]
❑
❍ Nej [no]
❍ Vet ej / vill ej svara [998]

❖ **C. FÖR VARJE MARKERAD FRÅGA VISA FÖLJANDE FRÅGOR**

[soc_wrkexper3_yr]

SC290. Hur länge har du utsatts för {markerat svar} på arbetet?

❍ Mindre än 1 år [1]
❍ 1 till 2 år [2]
❍ 3 till 5 år [3]
❍ 6 till 10 år [4]
❍ Mer än 10 år [5]
❍ Vet ej / vill ej svara [998]

[soc_wrkexper3]

SC300. Hur ofta har du utsatts för {markerat svar} på arbetet?

❍ Varje dag [1]
❍ Varje vecka [2]
❍ Varje månad [3]
❍ Mindre ofta än varje månad [4]
❍ Vet ej / vill ej svara [998]

[soc_wrkexperhard2]

SC310. Har {markerat svar} försvårat ditt arbete?

❍ Ja [1]
❍ Nej [2]
❍ Vet ej / vill ej svara [998]

- **C. VILLKORET AVSLUTAS**
- **D. OM SC100 ÄR "ANSTÄLLD" ELLER "DRIVER EGET FÖRETAG…", VISA NEDAN FRÅGOR**

[soc_12mntsexper]

SC320. Har du erfarit något av följande problem under de senaste 12 månaderna?

Ja
❑ Olust att gå till arbetet [une]
❑ Oro för att inte klara av arbetet [wor]
❑ Osäkerhet på grund av omorganisation [reo]
❑ Osäkerhet på grund av varsel eller hot om stängning [not]
❑
❍ Nej [none]
❍ Vet ej / vill ej svara [998]

Om svaret "olust att gå till arbetet" är markerat i SC320, visa SC330

[soc_wrkexperhard_unease]

SC330. Har dina olustkänslor gjort det svårt för dig att arbeta?

❍ Ja [1]
❍ Nej [2]
❍ Vet ej / vill ej svara [998]

Om svaret "oro för att inte klara av arbetet" är markerad i SC320, visa SC340

[soc_wrkexperhard_worry]

SC340. Har din oro över att inte klara av jobbet gjort det svårt för dig att arbeta?

❍ Ja [1] ❍ Vet ej / vill ej svara [998]
❍ Nej [2]

Arbetsrelaterad stress

[soc_wrkstress1]

SC350. Frågor om stress på arbetet:

	Sällan eller aldrig [3]	Ibland [2]	För det mesta [1]	Vet ej / vill ej svara [998]
Är du tvungen att arbeta mycket fort eller intensivt? [fast]	☐	☐	☐	☐
Finns det ofta motstridiga krav på ditt arbete? [conf]	☐	☐	☐	☐
Måste du göra samma sak om och om igen? [same]	☐	☐	☐	☐
Kräver ditt arbete en alltför stor ansträngning? [effo]	☐	☐	☐	☐

[soc_wrkstress1_1]

SC360.

	För det mesta [1]	Ibland [2]	Sällan eller aldrig [3]	Vet ej / vill ej svara [998]

	För det mesta [1]	Ibland [2]	Sällan eller aldrig [3]	Vet ej / vill ej svara [998]
Kräver ditt arbete stort ansvarstagande? [resp]	☐	☐	☐	☐
Har du möjlighet att välja själv hur du utför ditt arbete? [chow]	☐	☐	☐	☐
Har du möjlighet att välja dina arbetsuppgifter själv? [choh]	☐	☐	☐	☐
Har du möjlighet att lära dig nya saker i ditt arbete? [lear]	☐	☐	☐	☐

[soc_wrkstress2]

SC370.

	För det mesta [1]	Ibland [2]	Sällan eller aldrig [3]	Vet ej / vill ej svara [998]
Jag tycker om mitt arbete [enjw]	☐	☐	☐	☐
Det är bra solidaritet på arbetet [soli]	☐	☐	☐	☐
Jag trivs med mina arbetskamrater [enjm]	☐	☐	☐	☐
Jag kommer väl överens med mina överordnade [supe]	☐	☐	☐	☐

[soc_cngjobs]

SC380.

	Nej [2]	Ja [1]	Vet ej / vill ej svara [998]
Funderar du på att byta jobb?	☐	☐	☐

[soc_wrkovertime]

SC390. Har du arbetat övertid under de senaste 12 månaderna?

Ja
❑ Tidiga mornar [mor]
❑ Under lunch eller raster [lun]
❑ Kvällar [eve]
❑ På helgerna [wee]
❑ Under semester [sem]
❑
❍ Nej [no]
❍ Vet ej / vill ej svara [998]

Om inte “nej”, visa SC400

[soc2_wrkovertime_hrs]

SC400. Hur många timmar övertid har du i genomsnitt arbetat per vecka?

❍ Mindre än 1timme [1]
❍ 1 till 5 timmar [2]
❍ 6 till 10 timmar [3]
❍ 11 till 20 timmar [4]
❍ 21 till 30 timmar [5]
❍ Mer än 30 timmar [6]
❍ Vet ej / vill ej svara [998]

Sjukskrivning

[soc_12mnts_sickshort]

SC413. Hur många gånger har du varit sjukskriven kortare än 15 dagar i följd under de senaste 12 månaderna?

❍ Ingen gång [4]
❍ 1 gång [3]
❍ 2 till 5 gånger [2]
❍ Mer än 5 gånger [1]
❍ Vet ej / vill ej svara [998]

[soc_12mnts_sicklong]

SC416. Hur många gånger har du varit sjukskriven 15 dagar i följd eller längre under de senaste 12 månaderna?

❍ Ingen gang [4] ❍ Mer än 5 gånger [1]
❍ 1 gång [3] ❍ Vet ej / vill ej svara [998]
❍ 2 till 5 gånger [2]

Om (SC413 eller SC416) är varken “ingen gång” eller “vet ej / vill ej svara”, visa SC420 och SC430

[soc_12mnts_sicktime]

SC420. Hur lång tid har du varit sjukskriven {totalt,}?

❍ (Dagar) [days] ______________[days_other]
❍ (Veckor) [wks] ____________[wks_other]
❍ (Månader) [mnts] ____________[mnts_other]
❍ Vet ej / vill ej svara [998]

[soc_12mnts_sick_why]

SC430. Av vilken anledning var du sjukskriven {, under den längsta perioden}?

❍ (Orsak) [cau] ____________[cau_other] ❍ Vet ej / vill ej svara [998]

[soc2_12mnts_wrkhealthrisk]

SC460. Hur många gånger under de senaste 12 månaderna gick du till arbetet fastän du borde ha stannat hemma på grund av sjukdom?

❍ Aldrig [5]
❍ 1 gång [4]
❍ 2 till 5 gånger [3]
❍ 6 till 9 gånger [2]
❍ 10 gånger eller mer [1]
❍ Vet ej / vill ej svara [998]

[soc_away1wk_lostwrk]

SC480. Om du är sjuk i en vecka, hur mycket av det arbete du missat måste du ta igen när du kommer tillbaka till arbetet?

❍ Inget [3] ❍ Allt [1]
❍ Ungefär hälften [2] ❍ Vet ej / vill ej svara [998]

Sömn och privatliv

[soc_12mnts_sleepprob]

SC490. Har du haft sömnsvårigheter på grund av arbetet under de senaste 12 månaderna?

Ja
❍ Några nätter per månad [4]
❍ En natt per vecka [3]
❍ Några nätter per vecka [2]
❍ Varje eller nästan varje natt [1]
❍
❍ Nej [5]
❍ Vet ej / vill ej svara [998]

[soc_wrkrelpriv]

SC500. Om du tänker på relationen mellan ditt arbetsliv och ditt privatliv:

	Sällan eller aldrig [3]	Ibland [2]	Ofta [1]	Vet ej / vill ej svara [998]
Påverkar kraven på arbetet ditt privatliv negativt? [work]	☐	☐	☐	☐
Påverkar ditt privatliv ditt arbete på ett negativt sätt? [priv]	☐	☐	☐	☐
Har du problem med att få tiden att räcka till för både arbete och privatliv? [time]	☐	☐	☐	☐

❖ **D. VILLKORET AVSLUTAS**

Livsstil

Innehåll

Innehåll 1
Allmänna frågor 3
Allmän hälsa 3
Livstillfredställelse och lycka 4
Allmän livskvalitet 6
Stress 6
Smärta 7
Trötthet 11
Matvanor 14
Måltider 14
Drycker 15
Livsmedel 17
Rätter 23
Potatis, pasta, ris, grönsaker och sås 27
Portionsstorlekar 30
Fett och salt 32
Matvanor 33
Kosttillskott 33
Fysisk aktivitet 36
Sysselsättningsaktivitet 36

Transport till sysselsättning 37
Fritidsaktiviteter 38
Sportaktiviteter 41
Sexualvanor 47
Allmänt 47
Sexuellt överförbara sjukdomar 48
Mobil och trådlöst 50
Mobiltelefon 50
DECT telefon 51
Trådlös datoranslutning 53

Allmänna frågor

Allmän hälsa

Markera vilket påstående som bäst beskriver ditt hälsotillstånd i dag.

[qua_eq5_1]

QL10. Rörlighet

❍ Jag går utan svårigheter [1]
❍ Jag kan gå men med viss svårighet [2]
❍ Jag är sängliggande [3]
❍ Vet ej / vill ej svara [998]

[qua_eq5_2]

QL20. Hygien

❍ Jag behöver ingen hjälp med min dagliga hygien, mat eller påklädning [1]
❍ Jag har vissa problem att tvätta eller klä mig själv [2]
❍ Jag kan inte tvätta eller klä mig själv [3]
❍ Vet ej / vill ej svara [998]

[qua_eq5_3]

QL30. Huvudsakliga aktiviteter (t.ex. arbete, studier, hushållssysslor, familje- eller fritidsaktiviteter)

❍ Jag klarar av mina huvudsakliga aktiviteter [1]
❍ Jag har vissa problem med att klara av mina huvudsakliga aktiviteter [2]
❍ Jag klarar inte av mina huvudsakliga aktiviteter [3]
❍ Vet ej / vill ej svara [998]

[qua_eq5_4]

QL40. Smärta / besvär

❍ Jag har varken smärtor eller besvär [1]
❍ Jag har måttliga smärtor eller besvär [2]
❍ Jag har svåra smärtor eller besvär [3]
❍ Vet ej / vill ej svara [998]

[qua_eq5_5]

QL50. Oro / nedstämdhet

❍ Jag är inte orolig eller nedstämd [1]
❍ Jag är orolig eller nedstämd i viss utsträckning [2]
❍ Jag är i högsta grad orolig eller nedstämd [3]

❍ Vet ej / vill ej svara [998]

[qua_eq5_vas_hea]

QL60. Vi vill att du på denna skala markerar hur bra eller dåligt ditt nuvarande hälsotillstånd är, som du själv bedömer det. Gör detta genom att klicka på den del av skalan som markerar hur bra eller dåligt ditt nuvarande hälsotillstånd är.

Ditt nuvarande hälsotillstånd

	0	1	2	3	4	5	6	7	8	9	10	
Sämsta tänkbara hälsotillstånd	☐	☐	☐	☐	☐	☐	☐	☐	☐	☐	☐	Bästa tänkbara hälsotillstånd

Livstillfredställelse och lycka

[qua2_satisfied_1]

QL170. Jag är nöjd med mitt liv.

	1	2	3	4	5	6	7	
Stämmer inte alls	☐	☐	☐	☐	☐	☐	☐	Stämmer helt och hållet

[qua2_gotimport_1]

QL180. Hittills har jag fått de viktiga saker som jag vill ha i livet.

	1	2	3	4	5	6	7	

	1	2	3	4	5	6	7	
Stämmer inte alls	☐	☐	☐	☐	☐	☐	☐	Stämmer helt och hållet

[qua2_nochange_1]

QL190. Om jag kunde leva om mitt liv skulle jag knappast ändra på något.

	1	2	3	4	5	6	7	
Stämmer inte alls	☐	☐	☐	☐	☐	☐	☐	Stämmer helt och hållet

[qua2_happy_1]

QL200. I allmänhet anser jag att jag är en lycklig människa.

	1	2	3	4	5	6	7	
Stämmer inte alls	☐	☐	☐	☐	☐	☐	☐	Stämmer helt och hållet

[qua2_peers_1]

QL210. Jag anser att jag är lyckligare än de flesta i min ålder.

	1	2	3	4	5	6	7	
Stämmer inte alls	☐	☐	☐	☐	☐	☐	☐	Stämmer helt och hållet

[qua2_regardless_1]

QL220. Jag trivs med livet vad som än händer och får ut det mesta av allt.

	1	2	3	4	5	6	7	
Stämmer inte alls	☐	☐	☐	☐	☐	☐	☐	Stämmer helt och hållet

[qua_socialactive_1]

QL225. Jag har ett lika aktivt socialt liv som de flesta i min ålder.

	1	2	3	4	5	6	7	
Stämmer inte alls	☐	☐	☐	☐	☐	☐	☐	Stämmer helt och hållet

Allmän livskvalitet

[qua2_overall_1]

QL230. Hur skulle du beskriva din allmänna livskvalitet så som du har känt dig den senaste månaden?

	0	1	2	3	4	5	6	7	8	9	10	
Sämsta tänkbara	☐	☐	☐	☐	☐	☐	☐	☐	☐	☐	☐	Bästa tänkbara

Stress

[qua_stress]

QL232. Följande frågor handlar om känslor och tankar under den senaste månaden.

Hur ofta har du:

	Aldrig [5]	Sällan [4]	Ibland [3]	Ganska ofta [2]	Mycket ofta [1]	Vet ej / vill ej svara [998]
Blivit upprörd över något	☐	☐	☐	☐	☐	☐

	Aldrig [5]	Sällan [4]	Ibland [3]	Ganska ofta [2]	Mycket ofta [1]	Vet ej / vill ej svara [998]
som skett helt oväntat? [1]						
Känt att du inte haft kontroll över de viktiga faktorerna i ditt liv? [2]	☐	☐	☐	☐	☐	☐
Känt dig nervös och stressad? [3]	☐	☐	☐	☐	☐	☐
Känt dig säker på din förmåga att hantera dina personliga problem? [4]	☐	☐	☐	☐	☐	☐
Tyckt att saker och ting har utvecklat sig som du velat? [5]	☐	☐	☐	☐	☐	☐
Känt att du inte kunde hantera allt som måste göras? [6]	☐	☐	☐	☐	☐	☐
Känt att du haft kontroll över irriterande moment i ditt liv? [7]	☐	☐	☐	☐	☐	☐
Känt att du har haft kontroll över saker och ting? [8]	☐	☐	☐	☐	☐	☐
Blivit arg över saker som har hänt och som låg utanför din kontroll? [9]	☐	☐	☐	☐	☐	☐
Tagit itu med dagliga förtret på ett tillfredställande sätt? [10]	☐	☐	☐	☐	☐	☐

Smärta

[qua_bpi1]

QL235. De flesta människor har någon gång i livet känt smärta (såsom lite huvudvärk, värk från en stukad led eller tandvärk). Har du under den senaste månaden känt någon annan smärta än dessa vanliga typer?

❍ Ja [1] ❍ Vet ej / vill ej svara [998]
❍ Nej [2]

❖ **C. VISA NEDAN FRÅGOR OM SVARET PÅ QL235 ÄR "JA".**

[qua_bpi1b]

QL236. På vilket eller vilka ställen känner du smärta? (Markera alla aktuella)

❑ Huvud [head] ❍ Vet ej / vill ej svara [998]
❑ Nacke [bacn]
❑ Axlar [shou]
❑ Övre rygg [uppb]
❑ Nedre rygg [lowb]
❑ Bröst [ches]
❑ Mage [stom]
❑ Armbågar [elbo]
❑ Händer eller handleder [hand]
❑ Höft [hip]
❑ Knän [knee]
❑ Fötter eller fotleder [feet]
❑ På annat ställe (ange) [oth] _____ [oth_other]

[qua_bpi1c]

QL237. Var gör det mest ont?

❍ Huvud [head] ❍ Vet ej / vill ej svara [998]
❍ Nacke [bacn]
❍ Axlar [shou]
❍ Övre rygg [uppb]
❍ Nedre rygg [lowb]
❍ Bröst [ches]
❍ Mage [stom]
❍ Armbågar [elbo]
❍ Händer eller handleder [hand]
❍ Höft [hip]
❍ Knän [knee]
❍ Fötter eller fotleder [feet]

❍ På det andra stället [oth]

[qua_bpi2]

QL238. Hur lång tid har du haft smärta under den senaste månaden?

❍ Mindre än en vecka [1] ❍ Vet ej / vill ej svara [998]
❍ 1 vecka [2]
❍ 2 veckor [3]
❍ 3 veckor eller längre [4]

[qua_bpi3_1]

QL240. Gradera den värsta smärta du haft under den senaste månaden.

	0	1	2	3	4	5	6	7	8	9	10	
Ingen smärta	☐	☐	☐	☐	☐	☐	☐	☐	☐	☐	☐	Värsta tänkbara smärta

[qua_bpi4_1]

QL250. Gradera den minsta smärta du haft under den senaste månaden.

	0	1	2	3	4	5	6	7	8	9	10	
Ingen smärta	☐	☐	☐	☐	☐	☐	☐	☐	☐	☐	☐	Värsta tänkbara smärta

[qua_bpi5_1]

QL260. Gradera den genomsnittliga smärta du har.

	0	1	2	3	4	5	6	7	8	9	10	
Ingen smärta	☐	☐	☐	☐	☐	☐	☐	☐	☐	☐	☐	Värsta tänkbara smärta

[qua_bpi6_1]

QL265. Gradera den smärta du har just nu.

	0	1	2	3	4	5	6	7	8	9	10	
Ingen smärta	☐	☐	☐	☐	☐	☐	☐	☐	☐	☐	☐	Värsta tänkbara smärta

[qua_bpi7]

QL270. Får du behandlingar eller tar du mediciner för din smärta?

❍ Ja [1] ❍ Vet ej / vill ej svara [998]
❍ Nej [2]

Visa QL280, om QL270 är besvarad "ja"

[qua_bpi8_1]

QL280. Hur mycket har behandlingen eller medicineringen lindrat smärtan under den senaste månaden?

	0	1	2	3	4	5	6	7	8	9	10	
Ingen lindring	☐	☐	☐	☐	☐	☐	☐	☐	☐	☐	☐	Fullständig lindring

❖ **D. VISA NEDAN FRÅGOR OM SVARET PÅ QL240 ÄR "4" ELLER HÖGRE.**

[qua_bpi9]

QL290. Gradera hur din smärta inverkat på följande under den senaste månaden:

	Inte alls 0	1	2	3	4	5	6	7	8	9	Fullständigt 10	Vet ej / vill ej svara [998]
Vardagliga aktiviteter [7]	☐	☐	☐	☐	☐	☐	☐	☐	☐	☐	☐	☐
Sinnesstämning [1]	☐	☐	☐	☐	☐	☐	☐	☐	☐	☐	☐	☐
Gångförmåga [3]	☐	☐	☐	☐	☐	☐	☐	☐	☐	☐	☐	☐
Normalt arbete (inkluderar arbete både i och utanför hemmet) [5]	☐	☐	☐	☐	☐	☐	☐	☐	☐	☐	☐	☐
Relationer till andra människor [2]	☐	☐	☐	☐	☐	☐	☐	☐	☐	☐	☐	☐
Sömn [4]	☐	☐	☐	☐	☐	☐	☐	☐	☐	☐	☐	☐
Förmåga att njuta av livet [6]	☐	☐	☐	☐	☐	☐	☐	☐	☐	☐	☐	☐

- **C OCH D. VILLKOREN AVSLUTAS.**

Trötthet

[qua_bfi1]

QL360. De flesta människor har någon gång i livet känt sig mycket trötta. Har du under senaste veckan känt dig ovanligt trött?

❍ Ja [1] ❍ Vet ej / vill ej svara [998]
❍ Nej [2]

- **E. VISA NEDAN FRÅGOR OM SVARET PÅ QL360 ÄR "JA".**

[qua_bfi2_1]

QL370. Gradera den trötthet du har just nu.

	0	1	2	3	4	5	6	7	8	9	10	
Ingen trötthet	☐	☐	☐	☐	☐	☐	☐	☐	☐	☐	☐	Värsta tänkbara trötthet

[qua_bfi3_1]

QL380. Gradera den trötthet du vanligtvis har.

	0	1	2	3	4	5	6	7	8	9	10	
Ingen trötthet	☐	☐	☐	☐	☐	☐	☐	☐	☐	☐	☐	Värsta tänkbara trötthet

[qua_bfi4_1]

QL390. Gradera den värsta trötthet du haft under den senaste veckan.

	0	1	2	3	4	5	6	7	8	9	10	
Ingen trötthet	☐	☐	☐	☐	☐	☐	☐	☐	☐	☐	☐	Värsta tänkbara trötthet

- **F. VISA NEDAN FRÅGOR OM SVARET PÅ QL390 ÄR “4” ELLER HÖGRE.**

[qua_bfi5]

QL400. Gradera hur din trötthet har inverkat på följande under den senaste veckan.

	Inte alls 0	1	2	3	4	5	6	7	8	9	Fullständigt 10	Vet ej / vill ej svara [998]
Vardagliga aktiviteter [7]	☐	☐	☐	☐	☐	☐	☐	☐	☐	☐	☐	☐
Sinnesstämning [1]	☐	☐	☐	☐	☐	☐	☐	☐	☐	☐	☐	☐
Gångförmåga [3]	☐	☐	☐	☐	☐	☐	☐	☐	☐	☐	☐	☐
Normalt arbete (inkluderar arbete både i och utanför hemmet) [5]	☐	☐	☐	☐	☐	☐	☐	☐	☐	☐	☐	☐
Relationer till andra människor [2]	☐	☐	☐	☐	☐	☐	☐	☐	☐	☐	☐	☐
Sömn [4]	☐	☐	☐	☐	☐	☐	☐	☐	☐	☐	☐	☐
Förmåga att njuta av livet [6]	☐	☐	☐	☐	☐	☐	☐	☐	☐	☐	☐	☐

❖ **E OCH F. VILLKOREN AVSLUTAS.**

Livsstil

Matvanor

Intro

Vi kommer att fråga dig om vad du ätit och druckit under de senaste månaderna. Svara hur du vanligtvis brukar äta och dricka under både vardagar och helger.

Måltider

[die_meals]

DI02. Hur ofta brukar du äta eller dricka något vid följande måltider?

	Ange hur ofta [frq]
Frukost [brea]	❍ Varje dag [1]<br>❍ Flera gånger per vecka [2]<br>❍ Någon gång per vecka [3]<br>❍ Mer sällan eller aldrig [4]
Lunch [lunc]	❍ Varje dag [1]<br>❍ Flera gånger per vecka [2]<br>❍ Någon gång per vecka [3]<br>❍ Mer sällan eller aldrig [4]
Middag eller kvällsmat [dinn]	❍ Varje dag [1]<br>❍ Flera gånger per vecka [2]<br>❍ Någon gång per vecka [3]<br>❍ Mer sällan eller aldrig [4]

[die_meals_coff_frq]

DI03. Hur ofta brukar du fika eller äta mellanmål?

❍ 4 gånger per dag eller mer [1]
❍ 3 gånger per dag [2]
❍ 1-2 gånger per dag [3]
❍ Några gånger per vecka [4]
❍ Mer sällan eller aldrig [5]
❍ Vet ej / vill ej svara [998]

Drycker

[die2_beverage]

DI04. För varje dryck du dricker minst en gång i månaden, välj i en av rullistorna hur ofta du brukar dricka dessa.

	Gånger per dag [day]	Gånger per vecka [week]
Vatten (från kran eller på flaska), 1 glas [wate]	❍ 1 [1]<br>❍ 2 [2]<br>❍ 3 [3]<br>❍ 4 [4]<br>❍ 5+ [5]	❍ 1-2 [1]<br>❍ 3-4 [2]<br>❍ 5-6 [3]
Kaffe [coff]	❍ 1 [1]<br>❍ 2 [2]<br>❍ 3 [3]<br>❍ 4 [4]<br>❍ 5+ [5]	❍ 1-2 [1]<br>❍ 3-4 [2]<br>❍ 5-6 [3]
Te [tea]	❍ 1 [1]<br>❍ 2 [2]<br>❍ 3 [3]<br>❍ 4 [4]<br>❍ 5+ [5]	❍ 1-2 [1]<br>❍ 3-4 [2]<br>❍ 5-6 [3]
Mjölk, i glas eller tallrik [milk]	❍ 1 [1]<br>❍ 2 [2]<br>❍ 3 [3]<br>❍ 4 [4]<br>❍ 5+ [5]	❍ 1-2 [1]<br>❍ 3-4 [2]<br>❍ 5-6 [3]
Juice [juic]	❍ 1 [1]<br>❍ 2 [2]	❍ 1-2 [1]<br>❍ 3-4 [2]

	Gånger per dag [day]	Gånger per vecka [week]
	❍ 3 [3]<br>❍ 4 [4]<br>❍ 5+ [5]	❍ 5-6 [3]
Läsk, cider, måltidsdryck eller saft [soda]	❍ 1 [1]<br>❍ 2 [2]<br>❍ 3 [3]<br>❍ 4 [4]<br>❍ 5+ [5]	❍ 1-2 [1]<br>❍ 3-4 [2]<br>❍ 5-6 [3]

❖ **A. OM KAFFE ELLER TE I OVAN DI04 BESVARATS ”3-4 GÅNGER PER VECKA” ELLER OFTARE VISA NEDANSTÅENDE FRÅGA**

Du nämnde att du dricker {nyckelfraser om te och kaffe från DI04}.

Om kaffe, visa DI05

[die_beverage_coff_acc]

DI05. Vad brukar du ha i ditt kaffe? (Markera alla aktuella)

❑ Mjölk (t.ex. i cappuccino eller latte) [milk]
❑ Socker eller sirap [suga]
❑ Sötningsmedel [swee]
❍ Inget av dessa [996]
❍ Vet ej / vill ej svara [998]

Om te, visa DI06 och DI07

[die_beverage_tea_kind]

DI06. Vilka av följande tesorter brukar du oftast dricka? (Markera alla aktuella)

❑ Svart te [blac]
❑ Grönt te [gree]
❑ Rött te [red]
❑ Örtte [herb]
❑ Annat te [oth]
❍ Vet ej / vill ej svara [998]

[die_beverage_tea_acc]

DI07. Vad brukar du ha i ditt te? (Markera alla aktuella)

❑ Mjölk (t.ex. i cappuccino eller latte) [milk]
❑ Socker eller sirap [suga]
❑ Sötningsmedel [swee]
❍ Inget av dessa [996]
❍ Vet ej / vill ej svara [998]

❖ **A. VILLKORET AVSLUTAS**

Livsmedel

[die2_food1a]

DI08. För varje livsmedel du äter minst en gång i månaden, välj i en av rullistorna hur ofta du brukar äta dessa.

	Gånger per dag [day]	Gånger per vecka [week]
Vitt bröd (t.ex. formbröd, limpa eller tunnbröd) [brew]	❍ 1 [1]<br>❍ 2 [2]<br>❍ 3 [3]<br>❍ 4 [4]<br>❍ 5+ [5]	❍ 1-2 [1]<br>❍ 3-4 [2]<br>❍ 5-6 [3]
Grovt mjukt bröd (t.ex. rågbulle, fullkornsbröd eller kavring) [bred]	❍ 1 [1]<br>❍ 2 [2]<br>❍ 3 [3]<br>❍ 4 [4]<br>❍ 5+ [5]	❍ 1-2 [1]<br>❍ 3-4 [2]<br>❍ 5-6 [3]
Hårt bröd [brer]	❍ 1 [1]<br>❍ 2 [2]<br>❍ 3 [3]<br>❍ 4 [4]<br>❍ 5+ [5]	❍ 1-2 [1]<br>❍ 3-4 [2]<br>❍ 5-6 [3]

	Gånger per dag [day]	Gånger per vecka [week]
Fil, yoghurt eller drickyoghurt [yogh]	❍ 1 [1]<br>❍ 2 [2]<br>❍ 3 [3]<br>❍ 4 [4]<br>❍ 5+ [5]	❍ 1-2 [1]<br>❍ 3-4 [2]<br>❍ 5-6 [3]
Müsli eller flingor [musl]	❍ 1 [1]<br>❍ 2 [2]<br>❍ 3 [3]<br>❍ 4 [4]<br>❍ 5+ [5]	❍ 1-2 [1]<br>❍ 3-4 [2]<br>❍ 5-6 [3]
Havregrynsgröt [oat]	❍ 1 [1]<br>❍ 2 [2]<br>❍ 3 [3]<br>❍ 4 [4]<br>❍ 5+ [5]	❍ 1-2 [1]<br>❍ 3-4 [2]<br>❍ 5-6 [3]

- **C. OM ”VITT BRÖD”, ”GROVT MJUKT BRÖD” ELLER ”HÅRT BRÖD” I OVAN DI07 BESVARATS ”3-4 GÅNGER PER VECKA” ELLER OFTARE VISA NEDANSTÅENDE FRÅGOR**

[die2_food1a_bread_amount]

DI09. Du nämnde att du äter bröd. Hur många skivor bröd äter du vanligtvis per gång?

❍ 1-2 skivor [1]
❍ 3-4 skivor [2]
❍ 5-6 skivor [3]
❍ 7 skivor eller fler [4]
❍ Vet ej / vill ej svara [998]

[die2_food1a_bread_spre_kind]

DI11. Vilka av följande smörgåsfetter använder du oftast på ditt bröd. (Markera alla aktuella)

❑ Bregott [breg]
❑ Smörgåsmargarin (t.ex. Lätta, Lätt & Lagom eller Becel) [marl]
❑ Kolesterolsänkande margarin (t.ex. Becel proactiv) [cole]
❑ Smör [butt]
❑ Annat smörgåsfett [oth]
❍ Använder ej smörgåsfett [no]
❍ Vet ej /vill ej svara [998]

- **C. VILLKORET AVSLUTAS**
- **D. OM FIL... ELLER FLINGOR... I OVAN DI07 BESVARATS 3-4 GÅNGER PER VECKA ELLER OFTARE VISA NEDANSTÅENDE FRÅGA**

Du nämnde att du äter {nyckelfraser för fil/yoghurt, müsli/flingor från DI07}.

Om fil..., visa DI12

[die2_food1a_yoghurt]

DI12. Vilka av följande sorters fil och yoghurt äter du vanligtvis? (Markera alla aktuella)

❑ Med tillsatt bakteriekultur (t.ex. A-fil, Verum hälsofil, Onaka eller Actimel) [bact]
❑ Naturell [natu]
❑ Med frukt-, bär- eller vaniljsmak [frui]
❑ Annan fil eller yoghurt [oth]
❍ Vet ej / vill ej svara [998]

Om flingor..., visa DI13

[die2_food1a_musli]

DI13. Vilka av följande sorters müsli eller flingor äter du vanligtvis? (Markera alla aktuella)

❑ Cornflakes eller Special K [corn]
❑ Start, Crunchy eller söta flingor (t.ex. Frosties eller Kalaspuffar) [star]
❑ Müsli eller fullkornsflingor (t.ex. All Bran) [musl]
❑ Andra flingor [oth]
❍ Vet ej / vill ej svara [998]

- **D. VILLKORET AVSLUTAS**

[die2_food1b]

DI13. För varje livsmedel du äter minst en gång i månaden, välj i en av rullistorna hur ofta du brukar äta dessa.

	Gånger per dag [day]	Gånger per vecka [week]

	Gånger per dag [day]	Gånger per vecka [week]
Ost [che]	❍ 1 [1]<br>❍ 2 [2]<br>❍ 3 [3]<br>❍ 4 [4]<br>❍ 5+ [5]	❍ 1-2 [1]<br>❍ 3-4 [2]<br>❍ 5-6 [3]
Köttpålägg (t.ex. skinka eller salami) [meat]	❍ 1 [1]<br>❍ 2 [2]<br>❍ 3 [3]<br>❍ 4 [4]<br>❍ 5+ [5]	❍ 1-2 [1]<br>❍ 3-4 [2]<br>❍ 5-6 [3]
Ägg eller omelett [egg]	❍ 1 [1]<br>❍ 2 [2]<br>❍ 3 [3]<br>❍ 4 [4]<br>❍ 5+ [5]	❍ 1-2 [1]<br>❍ 3-4 [2]<br>❍ 5-6 [3]
Linfrön [lins]	❍ 1 [1]<br>❍ 2 [2]<br>❍ 3 [3]<br>❍ 4 [4]<br>❍ 5+ [5]	❍ 1-2 [1]<br>❍ 3-4 [2]<br>❍ 5-6 [3]

- **E. OM ”OST” I OVAN DI13 BESVARATS ”3-4 GÅNGER PER VECKA” ELLER OFTARE VISA NEDAN FRÅGA**

[die2_food1b_cheeze]

DI14. Du nämnde att du äter ost. Hur många skivor ost äter du vanligtvis per gång?

❍ 1-2 skivor [1]
❍ 3-4 skivor [2]
❍ 5-6 skivor [3]
❍ 7-8 skivor [4]
❍ 9-10 skivor [5]
❍ Mer än 10 skivor [6]
❍ Vet ej / vill ej svara [998]

- **E. VILLKORET AVSLUTAS**

[die2_food2]

DI14. För varje livsmedel du äter minst en gång i månaden, välj i en av rullistorna hur ofta du brukar äta dessa.

	Gånger per vecka [week]	Gånger per månad [mnt]
Banan [bana]	❍ 1-2 [1]<br>❍ 3-4 [2]<br>❍ 5-6 [3]<br>❍ 7+ [4]	❍ 1-3 [1]
Äpple eller päron [appl]	❍ 1-2 [1]<br>❍ 3-4 [2]<br>❍ 5-6 [3]<br>❍ 7+ [4]	❍ 1-3 [1]
Apelsin, småcitrus eller grapefrukt [oran]	❍ 1-2 [1]<br>❍ 3-4 [2]<br>❍ 5-6 [3]<br>❍ 7+ [4]	❍ 1-3 [1]
Andra frukter eller bär [othf]	❍ 1-2 [1]<br>❍ 3-4 [2]<br>❍ 5-6 [3]<br>❍ 7+ [4]	❍ 1-3 [1]

[die2_food3]

DI15. För varje livsmedel du äter minst en gång i månaden, välj i en av rullistorna hur ofta du brukar äta dessa.

	Gånger per vecka [week]	Gånger per månad [mnt]
Kex, skorpor eller kakor [bisc]	❍ 1-2 [1]<br>❍ 3-4 [2]<br>❍ 5-6 [3]<br>❍ 7+ [4]	❍ 1-3 [1]
Bulle, muffin eller mjuk kaka [muff]	❍ 1-2 [1]<br>❍ 3-4 [2]<br>❍ 5-6 [3]<br>❍ 7+ [4]	❍ 1-3 [1]
Choklad [choc]	❍ 1-2 [1]<br>❍ 3-4 [2]<br>❍ 5-6 [3]<br>❍ 7+ [4]	❍ 1-3 [1]
Godis (ej choklad) [cand]	❍ 1-2 [1]<br>❍ 3-4 [2]<br>❍ 5-6 [3]<br>❍ 7+ [4]	❍ 1-3 [1]

	Gånger per vecka [week]	Gånger per månad [mnt]
Glass, sorbet eller parfait [icec]	❍ 1-2 [1]<br>❍ 3-4 [2]<br>❍ 5-6 [3]<br>❍ 7+ [4]	❍ 1-3 [1]
Nötter, mandlar eller frön (ej linfrön) [nuts]	❍ 1-2 [1]<br>❍ 3-4 [2]<br>❍ 5-6 [3]<br>❍ 7+ [4]	❍ 1-3 [1]
Chips eller ostbågar [chip]	❍ 1-2 [1]<br>❍ 3-4 [2]<br>❍ 5-6 [3]<br>❍ 7+ [4]	❍ 1-3 [1]

❖ **G. OM CHOKLAD ELLER GODIS I OVAN DI15 BESVARATS 1-2 GÅNGER PER VECKA ELLER OFTARE VISA NEDANSTÅENDE FRÅGOR**

Om choklad, visa DI16 och DI17

[die2_food3_choc_kind]

DI17. Vilka av följande chokladsorter äter du vanligtvis? (Markera alla aktuella)

❑ Snickers, Daim, Japp och liknande [crea]
❑ Mjölkchoklad [milk]
❑ Mörk choklad [dark]
❑ Vit choklad [whit]
❍ Vet ej / vill ej svara [998]

[die2_food3_choc_amount]

DI16. Du nämnde att du äter choklad. Hur mycket choklad äter du vanligtvis per gång?

1 stor chokladkaka motsvarar 200 g.

1 snickers eller daim motsvarar 50 g.

1 pralin motsvarar 10 g.

❍ Mindre än 25 g [1]
❍ 25-49 g [2]
❍ 50-99 g [3]
❍ 100-199 g [4]
❍ 200g eller mer [5]
❍ Vet ej / vill ej svara [998]

Om godis, visa DI18

[die2_food3_candy]

DI18. Du nämnde att du äter godis. Hur mycket godis äter du vanligtvis per gång? Räkna inte med choklad.

En godispåse, t.ex. bilar eller gott&blandat, motsvarar 150 g.

1 tablettask motsvarar 25 g.

❍ Mindre än 50 g [1]
❍ 50-99 g [2]
❍ 100-199 g [3]
❍ 200-299 g [4]
❍ 300 g eller mer [5]
❍ Vet ej / vill ej svara [998]

❖ **G. VILLKORET AVSLUTAS**

Rätter

[die2_meal1]

DI19. Vi frågar nu om lagade måltider. För varje kötträtt du äter minst en gång i månaden, välj i en av rullistorna hur ofta du brukar äta dessa.

	Gånger per vecka [week]	Gånger per månad [mnt]
Hamburgare, kebab eller Tex-mex med köttfärs (t.ex. tacos) [hamb]	❍ 1-2 [1]<br>❍ 3-4 [2]<br>❍ 5-6 [3]<br>❍ 7+ [4]	❍ 1-3 [1]
Köttfärsrätter (t.ex. köttfärssås, lasagne, köttbullar eller köttfärsbiff) [minc]	❍ 1-2 [1]<br>❍ 3-4 [2]<br>❍ 5-6 [3]	❍ 1-3 [1]

	Gånger per vecka [week]	Gånger per månad [mnt]
	❍ 7+ [4]	
Kyckling eller annan fågel (t.ex. stekt, kokt, friterad eller i gryta) [chic]	❍ 1-2 [1]<br>❍ 3-4 [2]<br>❍ 5-6 [3]<br>❍ 7+ [4]	❍ 1-3 [1]
Korv (t.ex. stekt, kokt, gratinerad eller i gryta) [saus]	❍ 1-2 [1]<br>❍ 3-4 [2]<br>❍ 5-6 [3]<br>❍ 7+ [4]	❍ 1-3 [1]
Fläsk- eller nötkött (t.ex. biff, filé eller i gryta) [beef]	❍ 1-2 [1]<br>❍ 3-4 [2]<br>❍ 5-6 [3]<br>❍ 7+ [4]	❍ 1-3 [1]
Lammkött eller viltkött [lamb]	❍ 1-2 [1]<br>❍ 3-4 [2]<br>❍ 5-6 [3]<br>❍ 7+ [4]	❍ 1-3 [1]
Blodpudding eller blodkorv [blac]	❍ 1-2 [1]<br>❍ 3-4 [2]<br>❍ 5-6 [3]<br>❍ 7+ [4]	❍ 1-3 [1]

[die2_meal2]

DI20. För varje maträtt du äter minst en gång i månaden, välj i en av rullistorna hur ofta du brukar äta dessa.

	Gånger per vecka [week]	Gånger per månad [mnt]
Vit fisk (t.ex. torsk, sej, fiskpinnar eller fiskbullar) [fish]	❍ 1-2 [1]<br>❍ 3-4 [2]<br>❍ 5-6 [3]<br>❍ 7+ [4]	❍ 1-3 [1]
Lax, sushi, strömming, sill eller makrill [salm]	❍ 1-2 [1]<br>❍ 3-4 [2]<br>❍ 5-6 [3]<br>❍ 7+ [4]	❍ 1-3 [1]

	Gånger per vecka [week]	Gånger per månad [mnt]
Tonfisk [tuna]	❍ 1-2 [1]<br>❍ 3-4 [2]<br>❍ 5-6 [3]<br>❍ 7+ [4]	❍ 1-3 [1]

Vegetariska rätter (t.ex. linsgryta, bönbiff, sojakorv eller quorn) [vego]	❍ 1-2 [1]<br>❍ 3-4 [2]<br>❍ 5-6 [3]<br>❍ 7+ [4]	❍ 1-3 [1]
Salladsrätter [sall]	❍ 1-2 [1]<br>❍ 3-4 [2]<br>❍ 5-6 [3]<br>❍ 7+ [4]	❍ 1-3 [1]
Fylld baguette, smörgås, wrap m.m. [bagu]	❍ 1-2 [1]<br>❍ 3-4 [2]<br>❍ 5-6 [3]<br>❍ 7+ [4]	❍ 1-3 [1]
Soppa [soup]	❍ 1-2 [1]<br>❍ 3-4 [2]<br>❍ 5-6 [3]<br>❍ 7+ [4]	❍ 1-3 [1]
Pizza, paj eller pirog [pizz]	❍ 1-2 [1]<br>❍ 3-4 [2]<br>❍ 5-6 [3]<br>❍ 7+ [4]	❍ 1-3 [1]
Pannkakor, plättar, ugnspannkaka eller våfflor [pann]	❍ 1-2 [1]<br>❍ 3-4 [2]<br>❍ 5-6 [3]<br>❍ 7+ [4]	❍ 1-3 [1]

- ❖ **I. OM ”ÖVRIGA VEGETARISKA RÄTTER…” I OVAN DI20 BESVARATS ”3-4 GÅNGER PER VECKA” ELLER ”SALLADSRÄTTER” I OVAN DI20 BESVARATS ”1-2 GÅNGER PER VECKA” VISA NEDANSTÅENDE FRÅGOR**

Du nämnde att du äter {nyckelfraser för vegetariska rätter och salladsrätter}.

Om ”vegetariska rätter…”, visa DI21

[die2_meal2_vego]

DI21. Vilka av följande vegetariska rätter äter du vanligtvis? (Markera alla aktuella)

❑ Bön-, lins- eller kikärtsrätter (t.ex. linsgryta, bönbiff eller falafel) [been]
❑ Sojakött, sojakorv eller tofu [soya]
❑ Quornfärs eller quorngryta [quor]
❑ Rotfruktsrätter med t.ex. rödbetor, palsternacka eller kålrot [root]
❍ Vet ej / vill ej svara [998]

Om salladsrätter, visa DI22

[die2_meal2_sallad_ingr]

DI22. Vilka av följande ingredienser är det vanligtvis i den sallad du äter? (Markera alla aktuella)

❑ Pasta, couscous, bulgur eller quinoa [past]
❑ Ost (t.ex. hårdost, fetaost, mozzarella eller keso) [chee]
❑ Skinka, korv, salami eller rostbiff [ham]
❑ Kyckling eller kalkon [chic]
❑ Lax, tonfisk eller skaldjur [salm]
❑ Bönor, linser eller ärter [bean]
❑ Andra ingredienser [oth]
❍ Vet ej / vill ej svara [998]

❖ **I. VILLKORET AVSLUTAS**

❖ **K. OM ”FYLLD BAGUETTE, SMÖRGÅS, WRAP MM” ELLER”SOPPA” I OVAN DI20 BESVARATS ”1-2 GÅNGER PER VECKA” VISA NEDANSTÅENDE FRÅGA**

Du nämnde att du äter {nyckelfraser för baguette och soppa}.

Om ”fylld baguette...”

[die2_meal2_baguette]

DI23. Vilka av följande ingredienser är det vanligtvis i den baguette-, smörgås- eller wrap-fyllning du äter? (Markera alla aktuella)

❑ Ost (t.ex. hårdost, fetaost, mozzarella eller keso) [chee]
❑ Skinka, korv, salami, köttbullar eller rostbiff [ham]
❑ Kyckling eller kalkon [chic]
❑ Lax, tonfisk eller skaldjur [salm]
❑ Grönsaker [vege]
❑ Röror (t.ex. skagenröra, tonfiskröra eller rödbetssallad) [skag]
❑ Andra ingredienser [oth]
❍ Vet ej / vill ej svara [998]

Om "soppa"

[die2_meal2_soup]

DI24. Vilka av följande soppor äter du vanligtvis? (Markera alla aktuella)

❑ Färdiga släta soppor (t.ex. Kelda, Blå Band eller Campbells) [read]
❑ Fisk- eller skaldjurssoppa [fish]
❑ Kött- eller kycklingsoppa [meat]
❑ Ärtsoppa [pea]
❑ Grönsakssoppa [vege]
❑ Annan soppa [oth]
❍ Vet ej / vill ej svara [998]

❖ **K. VILLKORET AVSLUTAS**

Potatis, pasta, ris, grönsaker och sås

[die2_food4]

DI25. För varje livsmedel du äter minst en gång i månaden, välj i en av rullistorna hur ofta du brukar äta dessa.

	Gånger per vecka [week]	Gånger per månad [mnt]
Kokt potatis, potatismos eller bakad potatis [pota]	❍ 1-2 [1]<br>❍ 3-4 [2]<br>❍ 5-6 [3]<br>❍ 7+ [4]	❍ 1-3 [1]
Pommes frites, stekt potatis eller klyftpotatis, pyttipanna eller potatisgratäng [pomm]	❍ 1-2 [1]<br>❍ 3-4 [2]<br>❍ 5-6 [3]<br>❍ 7+ [4]	❍ 1-3 [1]
Pasta eller nudlar [past]	❍ 1-2 [1]<br>❍ 3-4 [2]<br>❍ 5-6 [3]<br>❍ 7+ [4]	❍ 1-3 [1]
Ris [ricw]	❍ 1-2 [1]<br>❍ 3-4 [2]<br>❍ 5-6 [3]<br>❍ 7+ [4]	❍ 1-3 [1]
Couscous, bulgur eller quinoa [cous]	❍ 1-2 [1]<br>❍ 3-4 [2]<br>❍ 5-6 [3]<br>❍ 7+ [4]	❍ 1-3 [1]

[die2_vege]

DI26. För varje grönsak du äter minst en gång i månaden, välj i en av rullistorna hur ofta du brukar äta dessa.

	Gånger per vecka [week]	Gånger per månad [mnt]
Grönsaksblandning (t.ex. wok) [mixv]	❍ 1-2 [1]<br>❍ 3-4 [2]<br>❍ 5-6 [3]<br>❍ 7+ [4]	❍ 1-3 [1]
Tomat, gurka eller salladsblad [toma]	❍ 1-2 [1]<br>❍ 3-4 [2]<br>❍ 5-6 [3]	❍ 1-3 [1]

	Gånger per vecka [week]	Gånger per månad [mnt]
	❍ 7+ [4]	
Spenat eller rucola [spin]	❍ 1-2 [1]<br>❍ 3-4 [2]<br>❍ 5-6 [3]<br>❍ 7+ [4]	❍ 1-3 [1]
Lök, purjolök eller vitlök [onio]	❍ 1-2 [1]<br>❍ 3-4 [2]<br>❍ 5-6 [3]<br>❍ 7+ [4]	❍ 1-3 [1]
Morötter [carr]	❍ 1-2 [1]<br>❍ 3-4 [2]<br>❍ 5-6 [3]<br>❍ 7+ [4]	❍ 1-3 [1]
Avokado [avoc]	❍ 1-2 [1]<br>❍ 3-4 [2]<br>❍ 5-6 [3]<br>❍ 7+ [4]	❍ 1-3 [1]
Broccoli, brysselkål eller salladskål [brus]	❍ 1-2 [1]<br>❍ 3-4 [2]<br>❍ 5-6 [3]<br>❍ 7+ [4]	❍ 1-3 [1]
Oliver [oliv]	❍ 1-2 [1]<br>❍ 3-4 [2]<br>❍ 5-6 [3]<br>❍ 7+ [4]	❍ 1-3 [1]

[die2_sauc]

DI27. För varje sås eller dressing du äter minst en gång i månaden, välj i en av rullistorna hur ofta du brukar äta dessa.

	Gånger per vecka [week]	Gånger per månad [mnt]
Ketchup, tomatsås, chilisås eller tomatsalsa [toma]	❍ 1-2 [1]<br>❍ 3-4 [2]<br>❍ 5-6 [3]<br>❍ 7+ [4]	❍ 1-3 [1]
Vinägrett (olja och vinäger) [vina]	❍ 1-2 [1]<br>❍ 3-4 [2]<br>❍ 5-6 [3]	❍ 1-3 [1]

	Gånger per vecka [week]	Gånger per månad [mnt]
	❍ 7+ [4]	
Gräddsås, crème fraiche eller gräddfilssås [crem]	❍ 1-2 [1]<br>❍ 3-4 [2]<br>❍ 5-6 [3]<br>❍ 7+ [4]	❍ 1-3 [1]

Portionsstorlekar

[die_port_pota_1]

DI28. Hur stor portion äter du vanligtvis av följande? (Markera portionsstorlekar för de livsmedel som gäller dig)

	[1]	[2]	[3]	[4]	[5]
Potatis, ris, pasta m.m.	☐	☐	☐	☐	☐

[die_port_prot_1]

	[1]	[2]	[3]	[4]	[5]
Kött, fisk eller vegetariskt alternativ.	☐	☐	☐	☐	☐

[die_port_vege_1]

	[1]	[2]	[3]	[4]	[5]
Grönsaker (råa eller tillagade).	☐	☐	☐	☐	☐

Fett och salt

[die_misc_fat]

DI29. Vilka av följande matfetter använder du vanligtvis när du lagar mat eller bakar? (Markera alla aktuella)

❑ Smör [butt]
❑ Mat- och bakmargarin (t.ex. Milda) [marg]
❑ Flytande margarin (t.ex. Milda eller Becel) [flyt]
❑ Flytande blandning av smörolja och rapsolja [mixt]
❑ Rapsolja [raps]
❑ Olivolja [oliv]
❑ Matolja (t.ex. majsolja eller solrosolja) [oil]
❑ Annat matfett [oth]
❍ Jag använder inte matfett [none]
❍ Vet ej / vill ej svara [998]

[die_misc_salt]

DI30. Vilket eller vilka av följande påståenden stämmer in på dina matvanor? (Markera alla aktuella)

❑ Jag använder oftast salt när jag lagar mat [cook]
❑ Jag saltar oftast på maten på tallriken [food]
❍ Inget av dessa [996]
❍ Vet ej / vill ej svara [998]

[die_misc_light_frq]

DI31. Brukar du äta eller dricka sockerfria livsmedel eller livsmedel med låg fetthalt, dvs. lightprodukter?

❍ Ja, ofta [1]
❍ Ja, ibland [2]
❍ Sällan eller aldrig [3]
❍ Vet ej / vill ej svara [998]

Om DI31 är ”Ja,…”, visa DI32

[die_misc_light_kind]

DI32. Vilka av följande lightprodukter brukar du välja? (Markera alla aktuella)

❑ Läsk, måltidsdryck eller saft [soda]
❑ Sylt, glass, godis eller kakor [jam]
❑ Ost [chee]
❑ Mjölk, fil eller yoghurt [milk]
❑ Övriga mjölkprodukter (t.ex. grädde eller crème fraiche) [crea]
❑ Såser och dressingar [sauc]
❑ Charkprodukter (t.ex. korv, salami eller leverpastej) [prep]
❑ Andra produkter [oth]
❍ Vet ej / vill ej svara [998]

Matvanor

[die2_habits]

DI33. Vilket eller vilka av följande påståenden passar bäst in på dina matvanor? (Markera alla aktuella)

❑ Jag äter på snabbmatsrestaurang, gatukök eller pizzeria (inkl. ”take away”) minst 4 ggr/v [fast]
❑ Jag äter lunch eller middag på restaurang (inkl. ”take away”) minst 4 ggr/v [rest]
❍ Ingen av dessa [996]
❍ Vet ej / vill ej svara [998]

Om DI33 är ”jag äter lunch...”, visa DI34

[die2_restaurant]

DI34. Äter du av salladsbuffén på de restauranger där det serveras?

❍ Ja, varje vecka [1]
❍ Ja, någon gång i månaden [2]
❍ Sällan eller aldrig [3]
❍ Vet ej / vill ej svara [998]

Kosttillskott

[die2_suppl_use]

DI39. Brukar du äta vitaminer, mineraler eller andra tillskott?

❍ Ja, regelbundet [1]
❍ Ja, ibland [2]
❍ Nej [3]
❍ Vet ej / vill ej svara [998]

Om DI39 är ”Ja,…”, visa DI40

[die2_supple_vit]

DI40. För de kosttillskott du ätit under de senaste månaderna, välj i rullistan hur ofta du brukar äta dessa.

	Under de senaste månaderna [frq]
Multivitaminer eller mineraler [mult]	❍ Varje dag [1]<br>❍ Några gånger i veckan [2]<br>❍ Några gånger i månaden [3]<br>❍ I perioder [4]<br>❍ Mer sällan eller aldrig [5]
A-vitamin [vita]	❍ Varje dag [1]<br>❍ Några gånger i veckan [2]<br>❍ Några gånger i månaden [3]<br>❍ I perioder [4]<br>❍ Mer sällan eller aldrig [5]
B-vitaminer [vitb]	❍ Varje dag [1]<br>❍ Några gånger i veckan [2]<br>❍ Några gånger i månaden [3]<br>❍ I perioder [4]<br>❍ Mer sällan eller aldrig [5]
C-vitamin [vitc]	❍ Varje dag [1]<br>❍ Några gånger i veckan [2]<br>❍ Några gånger i månaden [3]<br>❍ I perioder [4]<br>❍ Mer sällan eller aldrig [5]
D-vitamin [vitd]	❍ Varje dag [1]<br>❍ Några gånger i veckan [2]<br>❍ Några gånger i månaden [3]<br>❍ I perioder [4]<br>❍ Mer sällan eller aldrig [5]
E-vitamin [vite]	❍ Varje dag [1]<br>❍ Några gånger i veckan [2]<br>❍ Några gånger i månaden [3]<br>❍ I perioder [4]<br>❍ Mer sällan eller aldrig [5]
Folsyra [foli]	❍ Varje dag [1]<br>❍ Några gånger i veckan [2]<br>❍ Några gånger i månaden [3]<br>❍ I perioder [4]<br>❍ Mer sällan eller aldrig [5]
Järn [iron]	❍ Varje dag [1]<br>❍ Några gånger i veckan [2]<br>❍ Några gånger i månaden [3]<br>❍ I perioder [4]<br>❍ Mer sällan eller aldrig [5]

Kalcium [calc]	❍ Varje dag [1]<br>❍ Några gånger i veckan [2]<br>❍ Några gånger i månaden [3]<br>❍ I perioder [4]<br>❍ Mer sällan eller aldrig [5]

Om DI39 är "Ja, regelbundet", visa DI41

[die_suppl_oth]

DI41. Vilka av följande tillskott har du ätit regelbundet under de senaste månaderna? (Markera alla aktuella)

❑ Betakaroten [beta]
❑ Vitamin B-komplex [vitb]
❑ Selen [sele]
❑ Zink [zink]
❑ Magnesium [magn]
❑ Q10 [q10]
❑ Antioxidanter (t.ex. Bio-Antioxidant eller Antioxidant Plus) [anti]
❑ Fytoöstrogener (t.ex. Menosoy eller Femiform) [phyt]
❑ Andra tillskott [oth]
❍ Inget av dessa [996]
❍ Vet ej / vill ej svara [998]

Fysisk aktivitet

- **OM ÅLDER < 18, ANVÄND MODULEN FYSISK AKTIVITET I TEMAT LIVSSTIL FÖR BARN**

Följande frågor avser din fysiska aktivitet under de senaste månaderna.

Sysselsättningsaktivitet

[phy_occact_1]

QL470. Vilken aktivitetsnivå har du vanligtvis i din dagliga sysselsättning (jobb, studier eller motsvarande)?

Sitter mest [1]	[2]	Står och går mest [3]	[4]	Tungt kroppsarbete [5]
☐	☐	☐	☐	☐

[phy3_occact_dur]

Ungefär hur många timmar per vecka utför du vanligtvis din dagliga sysselsättning (jobb, studier eller motsvarande)?

❍ 5 timmar eller mindre [1]
❍ 6 - 10 timmar [2]
❍ 11 – 20 timmar [3]
❍ 21 – 30 timmar [4]
❍ 31 – 40 timmar [5]
❍ 41 – 50 timmar [6]
❍ Mer än 50 timmar [7]
❍ Vet ej / vill ej svara [998]

Transport till sysselsättning

[phy3_occtran]

Hur tar du dig vanligtvis till och från jobb, studier eller annan daglig sysselsättning?

❑ Promenerar [wal]
❑ Cyklar [bic]
❑ Åker motorcykel, moped eller skoter [mc]
❑ Åker bil eller taxi [car]
❑ Åker buss, tåg, tunnelbana eller båt [bus]
❑ På annat sätt [oth]
❍ Gäller inte mig [996]
❍ Vet ej / vill ej svara [998]

- **A. FÖR VARJE SVAR MARKERAT I OVAN FRÅGA, VISA MOTSVARANDE NEDAN FRÅGA**

[phy3_occtran]

Hur ofta tar du dig till din dagliga sysselsättning på följande sätt och hur lång tid tar det?

	Antal dagar i veckan [frq]	Tid enkel väg per dag [dur]
Promenerar [wal]	❍ 1 [1]<br>❍ 2 [2]<br>❍ 3 [3]<br>❍ 4 [4]<br>❍ 5 [5]<br>❍ 6 [6]<br>❍ 7 [7]	❍ Mindre än 15 minuter [1]<br>❍ 15 - 29 minuter [2]<br>❍ 30 - 44 minuter [3]<br>❍ 45 - 59 minuter [4]<br>❍ 1 - 2 timmar [5]<br>❍ Mer än 2 timmar [6]
Cyklar [bic]	❍ 1 [1]<br>❍ 2 [2]<br>❍ 3 [3]<br>❍ 4 [4]<br>❍ 5 [5]<br>❍ 6 [6]<br>❍ 7 [7]	❍ Mindre än 15 minuter [1]<br>❍ 15 - 29 minuter [2]<br>❍ 30 - 44 minuter [3]<br>❍ 45 - 59 minuter [4]<br>❍ 1 - 2 timmar [5]<br>❍ Mer än 2 timmar [6]
Åker motorcykel, moped eller skoter [mc]	❍ 1 [1]<br>❍ 2 [2]<br>❍ 3 [3]<br>❍ 4 [4]<br>❍ 5 [5]<br>❍ 6 [6]<br>❍ 7 [7]	❍ Mindre än 15 minuter [1]<br>❍ 15 - 29 minuter [2]<br>❍ 30 - 44 minuter [3]<br>❍ 45 - 59 minuter [4]<br>❍ 1 - 2 timmar [5]<br>❍ Mer än 2 timmar [6]
Åker bil eller taxi [car]	❍ 1 [1]	❍ Mindre än 15 minuter [1]

	Antal dagar i veckan [frq]	Tid enkel väg per dag [dur]
	❍ 2 [2]<br>❍ 3 [3]<br>❍ 4 [4]<br>❍ 5 [5]<br>❍ 6 [6]<br>❍ 7 [7]	❍ 15 - 29 minuter [2]<br>❍ 30 - 44 minuter [3]<br>❍ 45 - 59 minuter [4]<br>❍ 1 - 2 timmar [5]<br>❍ Mer än 2 timmar [6]
Åker buss, tåg, tunnelbana eller båt [bus]	❍ 1 [1]<br>❍ 2 [2]<br>❍ 3 [3]<br>❍ 4 [4]<br>❍ 5 [5]<br>❍ 6 [6]<br>❍ 7 [7]	❍ Mindre än 15 minuter [1]<br>❍ 15 - 29 minuter [2]<br>❍ 30 - 44 minuter [3]<br>❍ 45 - 59 minuter [4]<br>❍ 1 - 2 timmar [5]<br>❍ Mer än 2 timmar [6]
På det andra sättet [oth]	❍ 1 [1]<br>❍ 2 [2]<br>❍ 3 [3]<br>❍ 4 [4]<br>❍ 5 [5]<br>❍ 6 [6]<br>❍ 7 [7]	❍ Mindre än 15 minuter [1]<br>❍ 15 - 29 minuter [2]<br>❍ 30 - 44 minuter [3]<br>❍ 45 - 59 minuter [4]<br>❍ 1 - 2 timmar [5]<br>❍ Mer än 2 timmar [6]

❖ **A. VILLKORET AVSLUTAS**

Fritidsaktiviteter

[phy_leiact_activity_1]

Vilken aktivitetsnivå har du vanligtvis på fritiden?

Sitter mest [1]	[2]	Promenerar 30 minuter per dag [3]	[4]	Ansträngande aktivitet 60 minuter per dag [5]
☐	☐	☐	☐	☐

[phy3_leiact]

Vilka aktiviteter ägnar du dig åt minst en gång i veckan under din fritid?

❑ Sitter och tittar på TV, DVD m.m. [tv]
❑ Sitter vid datorn, läser e-brev, spelar datorspel, playstation, Xbox m.m. [com]
❑ Sitter och läser, skriver, syr m.m. [rea]
❑ Spelar musikinstrument eller fysiskt aktiva dator- och TV-spel (t.ex. Wii) [phy]
❑ Hushållsarbetar, städar, tvättar, tar hand om barn, trädgårdsarbetar m.m. [hom]
❑ Handlar eller uträttar ärenden [sho]
❑ Går ut och dansar (t.ex. disco eller dansband) [dan]
❑ Promenerar (ej som transport till daglig sysselsättning), stavgång, rastar hunden [wal]
❑ Cyklar (ej som transport till daglig sysselsättning) [bic]
❍ Inget av dessa [996]
❍ Vet ej / vill ej svara [998]

❖ **B. FÖR VARJE SVAR SOM MARKERATS I OVAN FRÅGA, VISA MOTSVARANDE I NEDAN FRÅGA**

[phy3_leiact1]

Hur ofta ägnar du dig åt följande fritidsaktiviteter och hur lång tid gör du det per dag?

	Antal dagar i veckan [frq]	Total tid per dag [dur]
Sitter och tittar på TV, DVD m.m. [tv]	❍ 1 [1]<br>❍ 2 [2]<br>❍ 3 [3]<br>❍ 4 [4]<br>❍ 5 [5]<br>❍ 6 [6]<br>❍ 7 [7]	❍ Mindre än 30 minuter [1]<br>❍ 30 - 59 minuter [2]<br>❍ 1 - 2 timmar [3]<br>❍ 3 - 4 timmar [4]<br>❍ 5 - 8 timmar [5]<br>❍ Mer än 8 timmar [6]
Sitter vid datorn, läser e-brev, spelar datorspel, playstation, Xbox m.m. [com]	❍ 1 [1]<br>❍ 2 [2]<br>❍ 3 [3]<br>❍ 4 [4]<br>❍ 5 [5]<br>❍ 6 [6]<br>❍ 7 [7]	❍ Mindre än 30 minuter [1]<br>❍ 30 - 59 minuter [2]<br>❍ 1 - 2 timmar [3]<br>❍ 3 - 4 timmar [4]<br>❍ 5 - 8 timmar [5]<br>❍ Mer än 8 timmar [6]
Sitter och läser, skriver, syr m.m. [rea]	❍ 1 [1]<br>❍ 2 [2]<br>❍ 3 [3]<br>❍ 4 [4]	❍ Mindre än 30 minuter [1]<br>❍ 30 - 59 minuter [2]

	Antal dagar i veckan [frq]	Total tid per dag [dur]
	❍ 5 [5]<br>❍ 6 [6]<br>❍ 7 [7]	❍ 1 - 2 timmar [3]<br>❍ 3 - 4 timmar [4]<br>❍ 5 - 8 timmar [5]<br>❍ Mer än 8 timmar [6]
Spelar musikinstrument eller spelar fysiskt aktiva dator- och TV-spel (t.ex. Wii) [phy]	❍ 1 [1]<br>❍ 2 [2]<br>❍ 3 [3]<br>❍ 4 [4]<br>❍ 5 [5]<br>❍ 6 [6]<br>❍ 7 [7]	❍ Mindre än 30 minuter [1]<br>❍ 30 - 59 minuter [2]<br>❍ 1 - 2 timmar [3]<br>❍ 3 - 4 timmar [4]<br>❍ 5 - 8 timmar [5]<br>❍ Mer än 8 timmar [6]
Hushållsarbetar, städar, tvättar, tar hand om barn, trädgårdsarbetar m.m. [hom]	❍ 1 [1]<br>❍ 2 [2]<br>❍ 3 [3]<br>❍ 4 [4]<br>❍ 5 [5]<br>❍ 6 [6]<br>❍ 7 [7]	❍ Mindre än 30 minuter [1]<br>❍ 30 - 59 minuter [2]<br>❍ 1 - 2 timmar [3]<br>❍ 3 - 4 timmar [4]<br>❍ 5 - 8 timmar [5]<br>❍ Mer än 8 timmar [6]
Handlar eller uträttar ärenden [sho]	❍ 1 [1]<br>❍ 2 [2]<br>❍ 3 [3]<br>❍ 4 [4]<br>❍ 5 [5]<br>❍ 6 [6]<br>❍ 7 [7]	❍ Mindre än 30 minuter [1]<br>❍ 30 - 59 minuter [2]<br>❍ 1 - 2 timmar [3]<br>❍ 3 - 4 timmar [4]<br>❍ 5 - 8 timmar [5]<br>❍ Mer än 8 timmar [6]

[phy3_leiact2]

	Antal dagar i veckan [frq]	Total tid per dag [dur]
Går ut och dansar (t.ex. disco eller dansband) [dan]	❍ 1 [1]<br>❍ 2 [2]<br>❍ 3 [3]<br>❍ 4 [4]<br>❍ 5 [5]<br>❍ 6 [6]<br>❍ 7 [7]	❍ Mindre än 30 minuter [1]<br>❍ 30 - 59 minuter [2]<br>❍ 1 - 2 timmar [3]<br>❍ 3 - 4 timmar [4]<br>❍ 5 - 8 timmar [5]<br>❍ Mer än 8 timmar [6]
Promenerar (ej som transport till daglig	❍ 1 [1]	❍ Mindre än 30

	Antal dagar i veckan [frq]	Total tid per dag [dur]
sysselsättning), stavgång eller rastar hunden [wal]	❍ 2 [2]<br>❍ 3 [3]<br>❍ 4 [4]<br>❍ 5 [5]<br>❍ 6 [6]<br>❍ 7 [7]	minuter [1]<br>❍ 30 - 59 minuter [2]<br>❍ 1 - 2 timmar [3]<br>❍ 3 - 4 timmar [4]<br>❍ 5 - 8 timmar [5]<br>❍ Mer än 8 timmar [6]
Cyklar (ej som transport till daglig sysselsättning) [bic]	❍ 1 [1]<br>❍ 2 [2]<br>❍ 3 [3]<br>❍ 4 [4]<br>❍ 5 [5]<br>❍ 6 [6]<br>❍ 7 [7]	❍ Mindre än 30 minuter [1]<br>❍ 30 - 59 minuter [2]<br>❍ 1 - 2 timmar [3]<br>❍ 3 - 4 timmar [4]<br>❍ 5 - 8 timmar [5]<br>❍ Mer än 8 timmar [6]

❖ **B. VILLKORET AVSLUTAS**

Sportaktiviteter

[phy2_sport]

Tränar eller sportar du regelbundet?

❍ Ja [1] ❍ Vet ej / vill ej svara [998]
❍ Nej [2]

Om ”ja”, visa nedan frågor

[phy3_sports]

Vilka träningsformer eller sporter ägnar du dig vanligtvis åt? Om inte just din aktivitet finns med i listan, välj en liknande aktivitet.

❑ Gympapass eller aerobics [aer]
❑ Styrketräning {ålder >= 15: med vikter} [gym]
❑ Jogging, löpning eller orienteering [jog]
❑ Friidrott (t.ex. höjdhopp, längdhopp eller tresteg) [ath]
❑ {ålder >= 11: Spinning eller cykling i tuff terräng, ålder < 11: Cykling i tuff terräng} [spi]
❑ Simning [swi]
❑ Bollspel i lag (t.ex. fotboll, basket, volleyboll eller innebandy) [bal]
❑ Golf [gol]
❑ {ålder >= 18: Danskurs eller tävlingsdans, ålder >= 15: Dans (t.ex. pardans, balett, jazz eller street), ålder < 15: Dans (t.ex. balett, jazz, street)} [dan]
❑ Ridning [hor]
❑ Skridskoåkning, hockey eller bandy [hoc]
❑ Skidåkning utför eller längd [ski]
❑ Kampsport (t.ex. judo eller karate) [mar]
❑ Boxning eller brottning [box]
❑ Tennis, badminton eller bordtennis [ten]
❑ Squash [squ]
❑ Segling, surfing, kanot eller rodd [sai]
❑ Motorsport (t.ex. motorcross) [mot]
❑ Klättring [cli]
❑ Yoga, pilates eller Tai chi [yog]
❑ Annan träningsform eller sport(ange vilken) [oth]____________ [oth_other]
❑
❍ Ingen av dessa [996]
❍ Vet ej / vill ej svara [998]

- **C. FÖR VARJE SVAR PÅ OVAN FRÅGA, VISA MOTSVARANDE I NEDAN FRÅGA**

[phy3_sports]

Hur ofta ägnar du dig åt följande sporter och hur lång tid gör du det per gång?

	Antal gånger [frq]	Tid per gång [dur]
Gympapass eller aerobics [aer]	❍ 1 - 3 gånger per månad [1]<br>❍ 1 gång per vecka [2]<br>❍ 2 - 3 gånger per vecka [3]<br>❍ 4 - 5 gånger per vecka [4]<br>❍ 6 - 7 gånger per vecka [5]	❍ Mindre än 30 minuter [1]<br>❍ 30 - 59 minuter [2]<br>❍ 60 - 119 minuter [3]<br>❍ 2 - 4 timmar [4]<br>❍ Mer än 4 timmar [5]
Styrketräning {ålder >=15: med vikter} [gym]	❍ 1 - 3 gånger per månad [1]<br>❍ 1 gång per vecka [2]<br>❍ 2 - 3 gånger per vecka [3]<br>❍ 4 - 5 gånger per vecka [4]	❍ Mindre än 30 minuter [1]<br>❍ 30 - 59 minuter [2]<br>❍ 60 - 119 minuter [3]<br>❍ 2 - 4 timmar [4]<br>❍ Mer än 4 timmar [5]

	Antal gånger [frq]	Tid per gång [dur]
	❍ 6 - 7 gånger per vecka [5]	
Jogging, löpning eller orienteering [jog]	❍ 1 - 3 gånger per månad [1]<br>❍ 1 gång per vecka [2]<br>❍ 2 - 3 gånger per vecka [3]<br>❍ 4 - 5 gånger per vecka [4]<br>❍ 6 - 7 gånger per vecka [5]	❍ Mindre än 30 minuter [1]<br>❍ 30 - 59 minuter [2]<br>❍ 60 - 119 minuter [3]<br>❍ 2 - 4 timmar [4]<br>❍ Mer än 4 timmar [5]
Friidrott (t.ex. höjdhopp, längdhopp eller tresteg) [ath]	❍ 1 - 3 gånger per månad [1]<br>❍ 1 gång per vecka [2]<br>❍ 2 - 3 gånger per vecka [3]<br>❍ 4 - 5 gånger per vecka [4]<br>❍ 6 - 7 gånger per vecka [5]	❍ Mindre än 30 minuter [1]<br>❍ 30 - 59 minuter [2]<br>❍ 60 - 119 minuter [3]<br>❍ 2 - 4 timmar [4]<br>❍ Mer än 4 timmar [5]
{ålder >= 11: Spinning eller cykling i tuff terräng, ålder < 11: Cykling i tuff terräng} [spi]	❍ 1 - 3 gånger per månad [1]<br>❍ 1 gång per vecka [2]<br>❍ 2 - 3 gånger per vecka [3]<br>❍ 4 - 5 gånger per vecka [4]<br>❍ 6 - 7 gånger per vecka [5]	❍ Mindre än 30 minuter [1]<br>❍ 30 - 59 minuter [2]<br>❍ 60 - 119 minuter [3]<br>❍ 2 - 4 timmar [4]<br>❍ Mer än 4 timmar [5]
Simning [swi]	❍ 1 - 3 gånger per månad [1]<br>❍ 1 gång per vecka [2]<br>❍ 2 - 3 gånger per vecka [3]<br>❍ 4 - 5 gånger per vecka [4]<br>❍ 6 - 7 gånger per vecka [5]	❍ Mindre än 30 minuter [1]<br>❍ 30 - 59 minuter [2]<br>❍ 60 - 119 minuter [3]<br>❍ 2 - 4 timmar [4]<br>❍ Mer än 4 timmar [5]
Bollspel i lag (t.ex. fotboll, basket, volleyboll eller innebandy) [bal]	❍ 1 - 3 gånger per månad [1]<br>❍ 1 gång per vecka [2]<br>❍ 2 - 3 gånger per vecka [3]<br>❍ 4 - 5 gånger per vecka [4]<br>❍ 6 - 7 gånger per vecka [5]	❍ Mindre än 30 minuter [1]<br>❍ 30 - 59 minuter [2]<br>❍ 60 - 119 minuter [3]<br>❍ 2 - 4 timmar [4]<br>❍ Mer än 4 timmar [5]
Golf [gol]	❍ 1 - 3 gånger per månad [1]<br>❍ 1 gång per vecka [2]<br>❍ 2 - 3 gånger per	❍ Mindre än 30 minuter [1]<br>❍ 30 - 59 minuter [2]<br>❍ 60 - 119 minuter [3]

	Antal gånger [frq]	Tid per gång [dur]
	vecka [3]<br>❍ 4 - 5 gånger per vecka [4]<br>❍ 6 - 7 gånger per vecka [5]	❍ 2 - 4 timmar [4]<br>❍ Mer än 4 timmar [5]
{ålder >= 18: Danskurs eller tävlingsdans, ålder >= 15: Dans (t.ex. pardans, balett, jazz eller street), ålder < 15: Dans (t.ex. balett, jazz, street)} [dan]	❍ 1 - 3 gånger per månad [1]<br>❍ 1 gång per vecka [2]<br>❍ 2 - 3 gånger per vecka [3]<br>❍ 4 - 5 gånger per vecka [4]<br>❍ 6 - 7 gånger per vecka [5]	❍ Mindre än 30 minuter [1]<br>❍ 30 - 59 minuter [2]<br>❍ 60 - 119 minuter [3]<br>❍ 2 - 4 timmar [4]<br>❍ Mer än 4 timmar [5]
Ridning [hor]	❍ 1 - 3 gånger per månad [1]<br>❍ 1 gång per vecka [2]<br>❍ 2 - 3 gånger per vecka [3]<br>❍ 4 - 5 gånger per vecka [4]<br>❍ 6 - 7 gånger per vecka [5]	❍ Mindre än 30 minuter [1]<br>❍ 30 - 59 minuter [2]<br>❍ 60 - 119 minuter [3]<br>❍ 2 - 4 timmar [4]<br>❍ Mer än 4 timmar [5]
Skridskoåkning, hockey eller bandy [hoc]	❍ 1 - 3 gånger per månad [1]<br>❍ 1 gång per vecka [2]<br>❍ 2 - 3 gånger per vecka [3]<br>❍ 4 - 5 gånger per vecka [4]<br>❍ 6 - 7 gånger per vecka [5]	❍ Mindre än 30 minuter [1]<br>❍ 30 - 59 minuter [2]<br>❍ 60 - 119 minuter [3]<br>❍ 2 - 4 timmar [4]<br>❍ Mer än 4 timmar [5]
Skidåkning utför eller längd [ski]	❍ 1 - 3 gånger per månad [1]<br>❍ 1 gång per vecka [2]<br>❍ 2 - 3 gånger per vecka [3]<br>❍ 4 - 5 gånger per vecka [4]<br>❍ 6 - 7 gånger per vecka [5]	❍ Mindre än 30 minuter [1]<br>❍ 30 - 59 minuter [2]<br>❍ 60 - 119 minuter [3]<br>❍ 2 - 4 timmar [4]<br>❍ Mer än 4 timmar [5]
Kampsport (t.ex. judo eller karate) [mar]	❍ 1 - 3 gånger per månad [1]<br>❍ 1 gång per vecka [2]<br>❍ 2 - 3 gånger per vecka [3]<br>❍ 4 - 5 gånger per vecka [4]<br>❍ 6 - 7 gånger per vecka [5]	❍ Mindre än 30 minuter [1]<br>❍ 30 - 59 minuter [2]<br>❍ 60 - 119 minuter [3]<br>❍ 2 - 4 timmar [4]<br>❍ Mer än 4 timmar [5]
Boxning eller brottning [box]	❍ 1 - 3 gånger per	❍ Mindre än 30

	Antal gånger [frq]	Tid per gång [dur]
	månad [1]<br>❍ 1 gång per vecka [2]<br>❍ 2 - 3 gånger per vecka [3]<br>❍ 4 - 5 gånger per vecka [4]<br>❍ 6 - 7 gånger per vecka [5]	minuter [1]<br>❍ 30 - 59 minuter [2]<br>❍ 60 - 119 minuter [3]<br>❍ 2 - 4 timmar [4]<br>❍ Mer än 4 timmar [5]
Tennis, badminton eller bordtennis [ten]	❍ 1 - 3 gånger per månad [1]<br>❍ 1 gång per vecka [2]<br>❍ 2 - 3 gånger per vecka [3]<br>❍ 4 - 5 gånger per vecka [4]<br>❍ 6 - 7 gånger per vecka [5]	❍ Mindre än 30 minuter [1]<br>❍ 30 - 59 minuter [2]<br>❍ 60 - 119 minuter [3]<br>❍ 2 - 4 timmar [4]<br>❍ Mer än 4 timmar [5]
Squash [squ]	❍ 1 - 3 gånger per månad [1]<br>❍ 1 gång per vecka [2]<br>❍ 2 - 3 gånger per vecka [3]<br>❍ 4 - 5 gånger per vecka [4]<br>❍ 6 - 7 gånger per vecka [5]	❍ Mindre än 30 minuter [1]<br>❍ 30 - 59 minuter [2]<br>❍ 60 - 119 minuter [3]<br>❍ 2 - 4 timmar [4]<br>❍ Mer än 4 timmar [5]
Segling, surfing, kanot eller rodd [sai]	❍ 1 - 3 gånger per månad [1]<br>❍ 1 gång per vecka [2]<br>❍ 2 - 3 gånger per vecka [3]<br>❍ 4 - 5 gånger per vecka [4]<br>❍ 6 - 7 gånger per vecka [5]	❍ Mindre än 30 minuter [1]<br>❍ 30 - 59 minuter [2]<br>❍ 60 - 119 minuter [3]<br>❍ 2 - 4 timmar [4]<br>❍ Mer än 4 timmar [5]
Motorsport (t.ex. motorcross) [mot]	❍ 1 - 3 gånger per månad [1]<br>❍ 1 gång per vecka [2]<br>❍ 2 - 3 gånger per vecka [3]<br>❍ 4 - 5 gånger per vecka [4]<br>❍ 6 - 7 gånger per vecka [5]	❍ Mindre än 30 minuter [1]<br>❍ 30 - 59 minuter [2]<br>❍ 60 - 119 minuter [3]<br>❍ 2 - 4 timmar [4]<br>❍ Mer än 4 timmar [5]
Klättring [cli]	❍ 1 - 3 gånger per månad [1]<br>❍ 1 gång per vecka [2]<br>❍ 2 - 3 gånger per vecka [3]<br>❍ 4 - 5 gånger per vecka [4]	❍ Mindre än 30 minuter [1]<br>❍ 30 - 59 minuter [2]<br>❍ 60 - 119 minuter [3]<br>❍ 2 - 4 timmar [4]<br>❍ Mer än 4 timmar [5]

	Antal gånger [frq]	Tid per gång [dur]
	❍ 6 - 7 gånger per vecka [5]	
Yoga, pilates eller Tai chi [yog]	❍ 1 - 3 gånger per månad [1]<br>❍ 1 gång per vecka [2]<br>❍ 2 - 3 gånger per vecka [3]<br>❍ 4 - 5 gånger per vecka [4]<br>❍ 6 - 7 gånger per vecka [5]	❍ Mindre än 30 minuter [1]<br>❍ 30 - 59 minuter [2]<br>❍ 60 - 119 minuter [3]<br>❍ 2 - 4 timmar [4]<br>❍ Mer än 4 timmar [5]
Din andra träningsform eller sport (**systemet visar angiven sport**) [oth]	❍ 1 - 3 gånger per månad [1]<br>❍ 1 gång per vecka [2]<br>❍ 2 - 3 gånger per vecka [3]<br>❍ 4 - 5 gånger per vecka [4]<br>❍ 6 - 7 gånger per vecka [5]	❍ Mindre än 30 minuter [1]<br>❍ 30 - 59 minuter [2]<br>❍ 60 - 119 minuter [3]<br>❍ 2 - 4 timmar [4]<br>❍ Mer än 4 timmar [5]

- **C. VILLKORET AVSLUTAS**
- **D. OM EN ELLER FRÅGOR INOM VILLKORET C BESVARATS ”2 – 3 gånger per vecka” ELLER OFTARE VISA NEDAN FRÅGA**

[phy3_prof]

Tävlar du regelbundet inom någon sport?

❍ Ja [1]   ❍ Vet ej / vill ej svara [998]
❍ Nej [2]

Sexualvanor

Allmänt

[sex_hadsex]

SE10. Med vilka av följande har du någon gång haft någon typ av sexuellt umgänge? (Markera alla aktuella)

❑ Man [man] ❍ Jag har aldrig haft någon typ av sex [nev]
❑ Kvinna [wom] ❍ Vet ej / vill ej svara [998]

❖ **A. OM SE10 ÄR "MAN" ELLER "KVINNA" VISA NEDAN FRÅGOR**

[sex_type]

SE20. Vilka av följande typer av sexuellt umgänge har du någon gång haft? (Markera alla aktuella)

❑ Vaginalsex [vag] ❑ Annan typ av sex [oth]
❑ Oralsex [ora] ❍ Vet ej / vill ej svara [998]
❑ Analsex [ana]

❖ **B. AVSLUTA MODUL OM SE20 BESVARAS "VET EJ / VILL EJ SVARA" OCH ÅLDER < 18**

[sex_firsttime_age]

SE30. Hur gammal var du när du hade sexuellt umgänge för första gången?

❍ (År gammal) [yrs] ____________[yrs_other] ❍ Vet ej / vill ej svara [998]

[sex_partners]

SE40. Hur många sexpartners har du haft under det senaste året?

❑ Fasta förbindelser (ange antal) [ste] ❍ Jag har inte haft sex under det

_______[ste_other]
❑ Tillfälliga förbindelser (ange antal) [one]_________ [one_other]

senaste året [not]
❍ Vet ej / vill ej svara [998]

[sex_condom]

SE50. Har du någon gång använt kondom vid sexuellt umgänge?

❍ Ja [1]
❍ Nej [2]
❍ Vet ej / vill ej svara [998]

Om SE50 är "ja", visa SE60 och SE70

[sex_condom_steady]

SE60. Hur ofta använder du kondom när du har sexuellt umgänge i fasta förbindelser?

❍ Varje gång [1]
❍ Nästan varje gång [2]
❍ Ofta [3]
❍ Ibland [4]
❍ Aldrig [5]
❍ Gäller inte mig [994]
❍ Vet ej / vill ej svara [998]

[sex_condom_onenight]

SE70. Hur ofta använder du kondom när du har sexuellt umgänge i tillfälliga förbindelser?

❍ Varje gang [1]
❍ Nästan varje gang [2]
❍ Ofta [3]
❍ Ibland [4]
❍ Aldrig [5]
❍ Gäller inte mig [994]
❍ Vet ej / vill ej svara [998]

Sexuellt överförbara sjukdomar

[sex_risk]

SE80. Hur stor risk tror du att du har för att råka ut för sexuellt överförbara sjukdomar?

❍ Ingen risk [4]
❍ Liten risk [3]
❍ Måttlig risk [2]
❍ Stor risk [1]
❍ Vet ej / vill ej svara [998]

[sex_disease]

SE90. Har en läkare eller annan vårdpersonal någon gång informerat dig om att du har en sexuellt överförbar sjukdom?

❍ Ja [1] ❍ Vet ej / vill ej svara [998]
❍ Nej [2]

Om SE90 är "ja", visa SE100

[sex_disease_type]

SE100. Har du någon gång blivit informerad om att du har någon av följande sexuellt överförbara sjukdomar? (Markera alla aktuella)

Ja
❑ Klamydia [chl]
❑ Herpes [her]
❑ Gonnoré [gon]
❑ Kondylom [con]
❑ Trichomonas [tri]
❑ Annan sjukdom [oth]
❍ Nej [no]
❍ Vet ej / vill ej svara [998]

❖ **A. VILLKORET AVSLUTAS**

Mobil och trådlöst

Mobiltelefon

[wir_dur]

MO640. I hur många år har du använt mobiltelefon minst en gång i veckan?

❍ Mindre än 2 år [1]
❍ 2 till 4 år [2]
❍ 5 till 8 år [3]
❍ 9 till 12 år [4]
❍ 13 till 16 år [5]
❍ 17 till 20 år [6]
❍ Mer än 20 år [7]
❍ Jag har aldrig använt mobiltelefon minst en gång i veckan [nev]
❍ Vet ej / vill ej svara [998]

❖ **A. OM SVARET PÅ MO640 INTE ÄR "JAG HAR ALDRIG ANVÄNT…" VISA NEDANSTÅENDE FRÅGOR**

[wir_week_frq]

MO650. Hur lång tid per vecka använder du din mobiltelefon för att ringa med för närvarande?

❍ Mindre än 5 minuter [1]
❍ 5 till 29 minuter [2]
❍ 30 till 59 minuter [3]
❍ 1 till 3 timmar [4]
❍ 4 till 6 timmar [5]
❍ 7 till 9 timmar [6]
❍ 10 timmar eller mer [7]
❍ Vet ej / vill ej svara [998]

[wir_2yrdiff]

MO660. I vilken utsträckning använder du din mobiltelefon jämfört med för två år sedan?

❍ Oftare nuförtiden [1]
❍ I samma utsträckning [2]
❍ Mindre nuförtiden [3]
❍ Vet ej / vill ej svara [998]

[wir_hndsfree]

MO670. Hur ofta använder du s.k. "hands-free" (öronsnäcka) eller högtalare när du ringer?

❍ För det mesta [1]
❍ Ungefär halva tiden [2]
❍ Aldrig eller nästan aldrig [3]
❍ Vet ej / vill ej svara [998]

Om svaret är "för det mesta" eller "ungefär halva tiden", visa MO680

[wir_hndsfree_phloc]

MO680. När du ringer med s.k. "hands-free" (öronsnäcka) var har du vanligen mobiltelefonen?

❍ I handen [1]
❍ I eller nära byxfickan fram [2]
❍ I eller nära bakfickan på byxan [3]
❍ I bröstfickan eller hängande runt halsen [4]
❍ På annat ställe [5]
❍ Vet ej / vill ej svara [998]

[wir_whcside]

MO690. Om du inte använder s.k. "hands-free" (öronsnäcka) när du ringer, på vilken sida av huvudet brukar du hålla mobiltelefonen?

❍ Höger sida [1]
❍ Vänster sida [2]
❍ Lika mycket på höger och vänster sida [3]
❍ Vet ej / vill ej svara [998]

[wir_handed]

MO691. Är du höger- eller vänsterhänt?

❍ Högerhänt [1]
❍ Vänsterhänt [2]
❍ Använder båda händer lika bra (ambidexter) [3]
❍ Vet ej / vill ej svara [998]

❖ **A. VILLKORET AVSLUTAS**

DECT telefon

[wir_dect]

MO700. Använder du bärbar sladdlös telefon som är ansluten till telefonnätet (DECT) minst en gång i veckan?

❍ Ja [1] ❍ Vet ej / vill ej svara [998]
❍ Nej [2]

If MO700 is “no”, show MO701

[wir_dect_prev]

MO701. Har du tidigare använt bärbar sladdlös telefon som är ansluten till telefonnätet (DECT) minst en gång i veckan?

❍ Ja [1] ❍ Vet ej / vill ej svara [998]
❍ Nej [2]

❖ **B. OM MO700 ÄR “JA” ELLER MO701 ÄR “JA” VISA NEDAN FRÅGOR**

[wir_dect_dur]

MO702. {Sedan hur länge har du använt, Hur länge använde du} sladdlös telefon?

❍ Mindre än 2 år [1]
❍ 2 till 4 år [2]
❍ 5 till 8 år [3]
❍ 9 till 12 år [4]
❍ Mer än 12 år [5]
❍ Vet ej / vill ej svara [998]

[wir_dect_week_frq]

MO704. Hur lång tid per vecka {använder du, använde du} sladdlös telefon?

❍ Mindre än 5 minuter [1]
❍ 5 till 29 minuter [2]
❍ 30 till 59 minuter [3]
❍ 1 till 3 timmar [4]
❍ 4 till 6 timmar [5]
❍ Mer än 6 timmar [6]
❍ Vet ej / vill ej svara [998]

❖ **B. VILLKORET AVSLUTAS**

Trådlös datoranslutning

[wir_comp]

MO706. Använder du någon av följande trådlösa anslutningar för anslutning till internet på datorer / bärbara datorer i {arbetet, skolan} eller på fritiden?

Ja ❑
❑ WLAN [wlan]
❑ 3G [3g]
❍ Nej [no]
❍ Vet ej / vill ej svara [998]

- ❖ **C. OM SVARET FÖR "WLAN" ELLER "3G" ÄR MARKERADE I MO706 VISA NEDANSTÅENDE FRÅGOR**

[wir_comp_dur]

MO710. I hur många år har du använt trådlös anslutning i {arbetet, skolan} eller på fritiden?

❍ Mindre än 2 år [1]
❍ 2 till 4 år [2]
❍ 5 till 8 år [3]
❍ 9 till 12 år [4]
❍ 13 till 16 år [5]
❍ 17 till 20 år [6]
❍ Mer än 20 år [7]
❍ Vet ej / vill ej svara [998]

[wir_comp_week_frq]

MO720. Hur många timmar per dag använder du trådlös anslutning i {arbetet, skolan} eller på fritiden?

❍ Mindre än 30 minuter [1]
❍ 30 till 59 minuter [2]
❍ 1 till 3 timmar [3]
❍ 4 till 6 timmar [4]
❍ 7 till 9 timmar [5]
❍ 10 till 13 timmar [6]
❍ Mer än 13 timmar [7]
❍ Vet ej / vill ej svara [998]

- ❖ **C. VILLKORET AVSLUTAS**

Egenvård

Innehåll

Innehåll 1

Receptfria mediciner 2

Komplementär- och alternativmedicin 5

Produkter 5

Behandlingar 5

Övningstekniker 7

Receptfria mediciner

[sel2_nonpresc]

ME10. Har {ditt barn, du} tagit någon av följande mediciner under den senaste månaden? (Markera alla aktuella)

❑ Alvedon, Panodil, Reliv (paracetamol) [alv]
❑ Ipren, Ibumetin (ibuprofen) [ibu]
❑ {ålder > 15: Voltaren, Diklofenak (diklofenak)} [vol]
❑ {ålder >= 12: Treo, Magnecyl, Albyl-minor (acetylsalicylsyra)} [tre]
❑ {ålder >= 12: Järntillskott} [iro]
❑ {>= 15: Nicotinell, Nicorette} [nic]
❑ Nässpray eller näsdroppar vid nästäppa (t.ex. Otrivin, Nezerril, Nasin) [dec]
❑ Hudlotion, salva (t.ex. Fenuril, Essex, Propyless) [lot]
❑ Kortisonkräm (t.ex. Hydrokortison, Mildison, Hyderm, Uniderm) [cor]
❑ Läkemedel vid förstoppning (t.ex. {om ålder >2, visa Inolaxol, om ålder 2-12, visa Movicol junior} Lactulos) [bow]
❑ Medicin som lindrar vid uppsvälld mage (t.ex. Dimetikon, Minifom) [blo]
❑
❍ Ingen av dessa [996]
❍ Vet ej / vill ej svara [998]

- **A. FÖR VARJE MEDICIN I ME10, VISA MOTSVARANDE I NEDAN FRÅGA**

[sel2_nonpresc]

ME15. Hur ofta har {ditt barn, du} tagit följande under den senaste månaden?

	Antal gånger [frq]
Alvedon, Panodil, Reliv (paracetamol) [alv]	❍ Ungefär en gång i månaden [1]<br>❍ Flera gånger i månaden [2]<br>❍ Ungefär en gång i veckan [3]<br>❍ Flera gånger i veckan [4]<br>❍ Ungefär en gång om dagen [5]<br>❍ Flera gånger per dag [6]
Ipren, Ibumetin (ibuprofen) [ibu]	❍ Ungefär en gång i månaden [1]<br>❍ Flera gånger i månaden [2]<br>❍ Ungefär en gång i veckan [3]<br>❍ Flera gånger i veckan [4]<br>❍ Ungefär en gång om dagen [5]<br>❍ Flera gånger per dag [6]
{ålder > 15: Voltaren, Diklofenak	❍ Ungefär en gång i månaden [1]<br>❍ Flera gånger i månaden [2]

	Antal gånger [frq]
(diklofenak)} [vol]	❍ Ungefär en gång i veckan [3]<br>❍ Flera gånger i veckan [4]<br>❍ Ungefär en gång om dagen [5]<br>❍ Flera gånger per dag [6]
{ålder >= 12: Treo, Magnecyl, Albyl-minor (acetylsalicylsyra)} [tre]	❍ Ungefär en gång i månaden [1]<br>❍ Flera gånger i månaden [2]<br>❍ Ungefär en gång i veckan [3]<br>❍ Flera gånger i veckan [4]<br>❍ Ungefär en gång om dagen [5]<br>❍ Flera gånger per dag [6]
{ålder >= 12: Järntillskott} [iro]	❍ Ungefär en gång i månaden [1]<br>❍ Flera gånger i månaden [2]<br>❍ Ungefär en gång i veckan [3]<br>❍ Flera gånger i veckan [4]<br>❍ Ungefär en gång om dagen [5]<br>❍ Flera gånger per dag [6]
{ålder >= 15: Nicotinell, Nicorette} [nic]	❍ Ungefär en gång i månaden [1]<br>❍ Flera gånger i månaden [2]<br>❍ Ungefär en gång i veckan [3]<br>❍ Flera gånger i veckan [4]<br>❍ Ungefär en gång om dagen [5]<br>❍ Flera gånger per dag [6]
Nässpray eller näsdroppar vid nästäppa (t.ex. Otrivin, Nezeril, Nasin) [dec]	❍ Ungefär en gång i månaden [1]<br>❍ Flera gånger i månaden [2]<br>❍ Ungefär en gång i veckan [3]<br>❍ Flera gånger i veckan [4]<br>❍ Ungefär en gång om dagen [5]<br>❍ Flera gånger per dag [6]
Hudlotion, salva (t.ex. Fenuril, Essex, Propyless) [lot]	❍ Ungefär en gång i månaden [1]<br>❍ Flera gånger i månaden [2]<br>❍ Ungefär en gång i veckan [3]<br>❍ Flera gånger i veckan [4]<br>❍ Ungefär en gång om dagen [5]<br>❍ Flera gånger per dag [6]
Kortisonkräm (t.ex. Hydrokortison, Mildison, Hyderm, Uniderm) [cor]	❍ Ungefär en gång i månaden [1]<br>❍ Flera gånger i månaden [2]<br>❍ Ungefär en gång i veckan [3]<br>❍ Flera gånger i veckan [4]<br>❍ Ungefär en gång om dagen [5]<br>❍ Flera gånger per dag [6]
Läkemedel vid förstoppning (t.ex.{om ålder >2, visa Inolaxol, om ålder 2-12, visa Movicol junior} Lactulos) [bow]	❍ Ungefär en gång i månaden [1]<br>❍ Flera gånger i månaden [2]<br>❍ Ungefär en gång i veckan [3]<br>❍ Flera gånger i veckan [4]<br>❍ Ungefär en gång om dagen [5]<br>❍ Flera gånger per dag [6]
Medicin som lindrar vid uppsvälld mage (t.ex. Dimetikon, Minifom) [blo]	❍ Ungefär en gång i månaden [1]<br>❍ Flera gånger i månaden [2]<br>❍ Ungefär en gång i veckan [3]<br>❍ Flera gånger i veckan [4]

	Antal gånger [frq]
	❍ Ungefär en gång om dagen [5]<br>❍ Flera gånger per dag [6]

❖ **A. VILLKORET AVSLUTAS**

Komplementär- och alternativmedicin

Produkter, behandlingar, övningstekniker

Produkter

[sel2_prod]

KA10. Har {ditt barn, du} använt någon av följande produkter någon gång i veckan under de senaste 12 månaderna? (Markera alla aktuella)

❑ Omega-3 ACO Omega 3, Omega Max, Friggs Eskimo 3, Pikasol, etc. [ome3]
❑ {ålder < 5: AD eller D- droppar} [addr]
❑ Multivitaminer med eller utan mineraler [mult]
❑ {ålder < 5: Magdroppar probiotiska droppar} [bell]
❑ Ginkgo BilobaBio-Biloba, Ginkomax, Gink-Yo, Proginko, Seredrin, etc. [gink]
❑ Echinacea Echinagard, Echinaforce, Esberitox, etc. [echi]
❑ Ginseng Gericomplex, Ginsana, etc. [gins]
❑ Kan Jang [kanj]
❑ Chi San [chis]
❑ Rosenrot [rosa]
❑ Valeriana Valeriana forte, Valeriena, etc. [vale]
❑ Johannesört Esbericum, Movina, Neurokan, etc. [esbe]
❑ Laktobaciller [lakt]
❑ Vitlöksprodukter Kwai, Kyolic, etc. [kwai]
❑
❍ Ingen av dessa [996]
❍ Vet ej / vill ej svara [998]

Behandlingar

❖ **B. OM ÅLDER >= 15, VISA NEDAN FRÅGOR**

[sel2_treat]

KA20. Har {ditt barn, du} fått någon av nedanstående behandlingar under de senaste 12 månaderna? (Markera alla aktuella)

❑ Sjukgymnastik [phy]
❑ Vattengymnastik [aqu]
❑
❑ Antroposofisk medicin [ant]
❑ Healing, Kristallterapi [hea]
❑

❑ Professionell massage [mas]
❑ Kiropraktisk behandling [chi]
❑ Naprapati [nap]
❑ Akupunktur [acu]
❑ Zonterapi, Reflexologi, Rosenmetoden [zon]
❑ Homeopati, Kinesiologi [hom]

❍ Ingen av dessa [996]
❍ Vet ej / vill ej svara [998]

❖ C. FÖR VARJE BEHANDLING I KA20, VISA MOTSVARANDE I NEDAN FRÅGA

[sel_treat]

KA25. Hur ofta har {ditt barn, du} fått följande behandling under de senaste 12 månaderna?

	Antal gånger [frq]
Sjukgymnastik [phy]	❍ Några gånger [1]<br>❍ Några gånger i månaden [2]<br>❍ Några gånger i veckan [3]
Vattengymnastik [aqu]	❍ Några gånger [1]<br>❍ Några gånger i månaden [2]<br>❍ Några gånger i veckan [3]
Professionell massage [mas]	❍ Några gånger [1]<br>❍ Några gånger i månaden [2]<br>❍ Några gånger i veckan [3]
Kiropraktisk behandling [chi]	❍ Några gånger [1]<br>❍ Några gånger i månaden [2]<br>❍ Några gånger i veckan [3]
Naprapati [nap]	❍ Några gånger [1]<br>❍ Några gånger i månaden [2]<br>❍ Några gånger i veckan [3]
Akupunktur [acu]	❍ Några gånger [1]<br>❍ Några gånger i månaden [2]<br>❍ Några gånger i veckan [3]
Zonterapi, Reflexologi, Rosenmetoden [zon]	❍ Några gånger [1]<br>❍ Några gånger i månaden [2]<br>❍ Några gånger i veckan [3]
Homeopati, Kinesiologi [hom]	❍ Några gånger [1]<br>❍ Några gånger i månaden [2]<br>❍ Några gånger i veckan [3]
Antroposofisk medicin [ant]	❍ Några gånger [1]<br>❍ Några gånger i månaden [2]<br>❍ Några gånger i veckan [3]
Healing, Kristallterapi [hea]	❍ Några gånger [1]<br>❍ Några gånger i månaden [2]<br>❍ Några gånger i veckan [3]

❖ **C. VILLKORET AVSLUTAS**

Övningstekniker

[sel2_tech]

KA30. Utövar {ditt barn, du} någon av följande övningstekniker regelbundet? (Markera alla aktuella)

❑ Yoga [yog]
❑ Tai Chi [tai]
❑ Qi gong [qig]
❑ Meditation [med]
❑ Avslappningsövningar [rel]
❑
❍ Ingen av dessa [996]
❍ Vet ej / vill ej svara [998]

❖ **D. FÖR VARJE ÖVNINGSTEKNIK I KA30, VISA MOTSVARANDE I NEDAN FRÅGA**

[sel_tech]

KA35. Hur ofta utövar {ditt barn, du} följande och sedan hur länge har {han/hon, du} gjort det?

	Antal gånger [frq]	Sedan antal år [dur]
Yoga [yog]	❍ Dagligen [1]<br>❍ Några gånger per vecka [2]<br>❍ Några gånger i månaden [3]<br>❍ Några gånger per år [4]	❍ Mindre än 1 år [1]<br>❍ 1 till 5 år [2]<br>❍ 6 till 10 år [3]<br>❍ Mer än 10 år [4]
Tai Chi [tai]	❍ Dagligen [1]<br>❍ Några gånger per vecka [2]<br>❍ Några gånger i månaden [3]<br>❍ Några gånger per år [4]	❍ Mindre än 1 år [1]<br>❍ 1 till 5 år [2]<br>❍ 6 till 10 år [3]<br>❍ Mer än 10 år [4]
Qi gong [qig]	❍ Dagligen [1]<br>❍ Några gånger per vecka [2]<br>❍ Några gånger i månaden [3]<br>❍ Några gånger per år [4]	❍ Mindre än 1 år [1]<br>❍ 1 till 5 år [2]<br>❍ 6 till 10 år [3]<br>❍ Mer än 10 år [4]

	Antal gånger [frq]	Sedan antal år [dur]
Meditation [med]	❍ Dagligen [1]<br>❍ Några gånger per vecka [2]<br>❍ Några gånger i månaden [3]<br>❍ Några gånger per år [4]	❍ Mindre än 1 år [1]<br>❍ 1 till 5 år [2]<br>❍ 6 till 10 år [3]<br>❍ Mer än 10 år [4]
Avslappningsövningar [rel]	❍ Dagligen [1]<br>❍ Några gånger per vecka [2]<br>❍ Några gånger i månaden [3]<br>❍ Några gånger per år [4]	❍ Mindre än 1 år [1]<br>❍ 1 till 5 år [2]<br>❍ 6 till 10 år [3]<br>❍ Mer än 10 år [4]

❖ **B OCH D. VILLKOREN AVSLUTAS**

Kvinnans hälsa

Innehåll

Innehåll 1

Menstruation / preventivmedel 2

Graviditet / födsel 6

Gynekologisk kirurgi 9

Infertilitet / sjukdomar / vaccin 11

Klimakteriet / osteoporos 14

Inkontinens 19

Menstruation / preventivmedel

[wom_mensfirst]

MN10. Hur gammal var du när du fick mens första gången?

❍ (Ålder) [yrs] ____________[yrs_other]
❍ Har inte fått mens [no]
❍ Vet ej / vill ej svara [998]

❖ **A. OM SVARET PÅ MN10 ÄR "ÅLDER" VISA NEDANSTÅENDE FRÅGOR**

[wom_lstyr_period]

MN20. Har du haft mens under det senaste året?

❍ Ja [1]
❍ Nej [2]
❍ Vet ej / vill ej svara [998]

Om "nej", visa MN30

[wom_lstyr_reason]

MN30. Vad är orsaken till att du inte har haft mens?

❍ Graviditet eller amning [1]
❍ Klimakteriet [2]
❍ Mediciner [3]
❍ Preventivmedel [4]
❍ Gynekologisk operation [5]
❍ Intensiv träning [6]
❍ Anorexi / ätstörning [7]
❍ Annan [8]
❍ Vet ej / vill ej svara [998]

Om inte "klimakteriet", visa MN40 till MN100

[wom_cycle]

MN40. Hur lång är din menstruationscykel, {vanligtvis}?

❍ 22 dagar eller kortare [1]
❍ 23 till 26 dagar [2]
❍ 27 till 30 dagar [3]
❍ 31 till 34 dagar [4]
❍ 35 till 38 dagar [5]
❍ Den är oregelbunden [7]
❍ Vet ej / vill ej svara [998]

❍ 39 dagar eller längre [6]

[wom_contr]

MN50. Har du någonsin använt något av följande preventivmedel?

Ja
❑ Minipiller [min]
❑ Kombinationspiller (vanliga p-piller) [com]
❑ P-spruta [pin]
❑ Hormonspiral [coi]
❑ P-stav [pim]
❑ Annat (vänligen ange) [oth] _____ [oth_other]
❍ Nej [no]
❍ Vet ej / vill ej svara [998]

Om MN50 är "ja", visa motsvarande nedan frågor MN51 till MN56

[wom_contr_mini]

MN51. Hur lång tid sammantaget har du tagit minipiller?

❍ (Antal år) [yrs] ____________[yrs_other]
❍ Mindre än 1 år [1]
❍ Vet ej / vill ej svara [998]

[wom_contr_comb]

MN52. Hur lång tid sammantaget har du tagit kombinationspiller (vanliga p-piller)?

❍ (Antal år) [yrs] ____________[yrs_other]
❍ Mindre än 1 år [1]
❍ Vet ej / vill ej svara [998]

[wom_contr_pinj]

MN53. Hur lång tid sammantaget har du tagit p-spruta?

❍ (Antal år) [yrs] ____________[yrs_other]
❍ Mindre än 1 år [1]
❍ Vet ej / vill ej svara [998]

[wom_contr_coil]

MN54. Hur lång tid sammantaget har du tagit hormonspiral?

❍ (Antal år) [yrs] ____________[yrs_other]
❍ Mindre än 1 år [1]
❍ Vet ej / vill ej svara [998]

[wom_contr_pimp]

MN55. Hur lång tid sammantaget har du tagit p-stav?

❍ (Antal år) [yrs] ____________[yrs_other]   ❍ Vet ej / vill ej svara [998]
❍ Mindre än 1 år [1]

[wom_contr_othe]

MN56. Hur lång tid sammantaget har du tagit {annat}?

❍ (Antal år) [yrs] ____________[yrs_other]   ❍ Vet ej / vill ej svara [998]
❍ Mindre än 1 år [1]

[wom_abnhair]

MN60. Tycker du att du har haft onormal hårväxt på olika kroppsdelar, t.ex. på överläppen, hakan, magen eller på låren?

❍ Ja [1]   ❍ Nej [2]
❍ Vet ej / vill ej svara [998]

Om MN30 inte är ”graviditet...”, visa MN70 till MN100

[wom_menspain]

MN70. Lider du av allvarliga menssmärtor?

❍ Ja [1]   ❍ Nej [2]
❍ Vet ej / vill ej svara [998]

Om “ja”, visa MN80

[wom_menspain_act]

MN80. Gör du regelbundet något av följande saker på grund av smärtan?

Ja
❑ Jag tar ledigt från jobbet [off]
❑ Jag tar värktabletter [pai]
❑ Jag tar p-piller [hor]
❑
❍ Nej [no]
❍ Vet ej / vill ej svara [998]

[wom_intercpain]

MN90. Känner du smärta vid samlag?

❍ Ja [1]   ❍ Nej [2]
❍ Vet ej / vill ej svara [998]

[wom_pelpain]

MN100. Lider du av smärtor i bäckenet under tiden mellan två menscykler?

❍ Ja [1]   ❍ Nej [2]
❍ Vet ej / vill ej svara [998]

Graviditet / födsel

Om MN30 inte är "graviditet…", visa MN110

[wom_pregn]

MN110. Har du någonsin varit gravid?

❍ Ja [1] ❍ Nej [2]
❍ Vet ej / vill ej svara [998]

❖ **B. OM SVARET PÅ MN110 ÄR "JA" ELLER MN30 ÄR "GRAVIDITET…" VISA NEDANSTÅENDE FRÅGOR**

[wom_pregn_now]

MN120. Är du gravid för närvarande?

❍ Ja [1] ❍ Nej [2]
❍ Vet ej / vill ej svara [998]

Om MN120 är "ja", visa MN125 och MN127

[wom_pregn_time]

MN125. Hur lång tid tog det för dig att bli gravid {, (under din senaste graviditet)}?

❍ 1 till 3 månader [1] ❍ Vet ej / vill ej svara [998]
❍ 4 till 12 månader [2]
❍ Mer än 1 år (ange antal år) [3] ______[3_other]
❍ Jag försökte inte bli gravid [4]

Om MN125 är "mer än 1 år" eller kortare tid, visa MN126

[wom_pregn_times]

MN126. Hur många gånger per månad försökte du och din partner att bli gravid?

❍ 1 till 4 gånger [1]
❍ 5 till 8 gånger [2]
❍ 9 till 12 gånger [3]
❍ Mer än 12 gånger [4]
❍ Jag blev gravid på annat sätt [5]
❍ Vet ej / vill ej svara [998]

[wom_pregn_how]

MN127. På vilket sätt blev du gravid?

❍ På naturligt sätt [1]
❍ Insemination med spermier [7]
❍ Provrörsbefruktning - IVF (In Vitro Fertilisering) [3]
❍ Provrörsbefruktning - ICSI [4]
❍ Provrörsbefruktning med äggdonation [8]
❍ Med hjälp av enbart hormonbehandling (stimulerad ägglossning) [5]
❍ Annat preparat eller behandling (vänligen ange) [6]_______[6_other]
❍ Vet ej / vill ej svara [998]

Om MN127 är ”insemination”, ”…IVF”, ”ICSI” eller ”…äggdonation”, visa MN128

[wom_pregn_how_sperm]

MN128. Användes spermier från partner eller donator?

❍ Från partner [1]

❍ Från donator [2]

❍ Vet ej / vill ej svara [998]

Om MN127 är ”provrörsbefruktning IVF” eller ”provrörsbefruktning ICSI”, visa MN129

[wom_pregn_how_egg]

MN129. Användes ägg från dig eller donator?

❍ Från dig själv [1]

❍ Från donator [2]

❍ Vet ej / vill ej svara [998]

[wom_pregn_nbr]

MN130. Hur många gånger har du varit gravid?

❍ (Antal gånger) [tms] ____________[tms_other] ❍ Vet ej / vill ej svara [998]

[wom_miscarr]

MN140. Har du någon gång fått missfall?

❍ Nej [no]
❍ Ja (Antal gånger) [tms] ____[tms_other]
❍ Vet ej / vill ej svara [998]

Om MN30 är ”graviditet…” eller MN110 är ”ja” samt att om MN30 inte är ”klimakteriet”, visa MN150

[wom_birth]

MN150. Har du fött barn?

❍ Ja [1] ❍ Nej [2]
❍ Vet ej / vill ej svara [998]

Om “ja”, visa MN160

[wom_birth_nbr]

MN160. Hur många gånger har du fött barn?

❍ (Antal gånger) [tms] ____________[tms_other] ❍ Vet ej / vill ej svara [998]

- **A OCH B. VILLKOREN AVSLUTAS**

Gynekologisk kirurgi

[wom_op_whc]

MN170. Har du någonsin genomgått någon typ av gynekologisk kirurgi (förutom kejsarsnitt)?

Ja
❑ Opererat livmoderhalsen [cerv]
❑ Opererat bort livmodern [uter]
❑ Opererat bort äggstock [ovar]
❑ Sterilisering [ster]
❑ Kirurgisk abort [abor]
❑ Kemisk abort [chem]
❑ Komplikationer under graviditet [comp]
❑ Annan operation [oth]
❑
❍ Nej [no]
❍ Vet ej / vill ej svara [998]

❖ **C. FÖR VARJE MARKERAT SVAR I MN17 VISA RESPEKTIVE FRÅGOR NEDAN**

Om "opererat livmoderhalsen", visa MN180

[wom_op_cerv_age]

MN180. Hur gammal var du (första gången) du opererade livmoderhalsen?

❍ (Ålder) [yrs]____________ [yrs_other] ❍ Vet ej / vill ej svara [998]

Om "opererat bort livmodern", visa MN190

[wom_op_uter_age]

MN190. Hur gammal var du när du opererade bort livmodern?

❍ (Ålder) [yrs]____________ [yrs_other] ❍ Vet ej / vill ej svara [998]

Om "opererat bort äggstockarna", visa MN200

[wom_op_ovar_age]

MN200. Hur gammal var du när du opererade bort äggstocken eller äggstockarna?
❍ (Ålder) [yrs]____________ [yrs_other] ❍ Vet ej / vill ej svara [998]

Om "sterilisering", visa MN210

[wom_op_ster_age]

MN210. Hur gammal var du när steriliseringen genomfördes?
❍ (Ålder) [yrs]____________ [yrs_other] ❍ Vet ej / vill ej svara [998]

Om "kirurgisk abort", visa MN220

[wom_op_abor_age]

MN220. Hur gammal var du (första gången) när den kirurgiska aborten genomfördes?
❍ (Ålder) [yrs]____________ [yrs_other] ❍ Vet ej / vill ej svara [998]

Om "kemisk abort", visa MN220

[wom_op_chem_age]

MN220. Hur gammal var du (första gången) när den kemiska aborten genomfördes?
❍ (Ålder) [yrs]____________ [yrs_other] ❍ Vet ej / vill ej svara [998]

Om "komplikationer under graviditet", visa MN230

[wom_op_comp_age]

MN230. Hur gammal var du (första gången) du opererades på grund av komplikationer under graviditet?

❍ (Ålder) [yrs]____________ [yrs_other] ❍ Vet ej / vill ej svara [998]

Om "annan operation", visa MN240

[wom_op_oth_age]

MN240. Hur gammal var du när du genomgick den andra operationen?

❍ (Ålder) [yrs]____________ [yrs_other] ❍ Vet ej / vill ej svara [998]

❖ **C. VILLKORET AVSLUTAS**

Infertilitet / sjukdomar / vaccin

Om MN127 inte är "...provrörsbefruktning" och ålder >= 18 och MN30 vare sig är ”graviditet...” eller ”klimakteriet”, visa MN270

[wom_infertcheck]

MN270. Har du någon gång undersökts eller behandlats för infertilitet?

❍ Ja [1] ❍ Nej [2]
❍ Vet ej / vill ej svara [998]

[wom_myoma_diag]

MN245. Har du fått diagnosen myom?

❍ Ja [1] ❍ Nej [2]
❍ Vet ej / vill ej svara [998]

Om Mn245 är "ja", visa MN247

[wom_myoma_treat]

MN247. Har du fått behandling för myom?

❍ Ja, kirurgisk [1]
❍ Ja, annan behandling [2]
❍ Nej [3]
❍ Vet ej / vill ej svara [998]

[wom_diagendomet]

MN250. Har du fått diagnosen endometrios?

❍ Ja [1]
❍ Nej [2]
❍ Vet ej / vill ej svara [998]

[wom_pco]

MN260. Har du fått diagnosen polycystiskt ovariesyndrom (PCO/PCOS)?

❍ Ja [1]
❍ Nej [2]
❍ Vet ej / vill ej svara [998]

[wom_cervix]

MN450. Har du fått vaccin mot livmoderhalscancer?

Ja
❍ Gardasil [gra]
❍ Cervarix [cer]
❍ Kommer inte ihåg namnet [don]
❍
❍ Nej [no]
❍ Vet ej / vill ej svara [998]

Om MN450 är "gardasil", "cervarix" or "Kommer inte ihåg namnet", visa MN455

[wom_cervix_age]

MN455. Hur gammal var du när du fick vaccinet för livmoderhalscancer?

❍ (År gammal) [yrs]_____________ [yrs_other] ❍ Vet ej / vill ej svara [998]
❍ Kommer inte ihåg [don]

Klimakteriet / osteoporos

❖ **D. OM ÅLDER > 40 OCH EJ GRAVID**

Om MN30 inte är "klimakteriet" och inte "graviditet…", visa MN280

[wom_menopause]

MN280. Har du kommit in i klimakteriet?

❍ Ja [1] ❍ Nej [2]
❍ Vet ej / vill ej svara [998]

❖ **E. OM SVARET PÅ MN280 ÄR "JA" ELLER MN30 ÄR "JA" VISA NEDANSTÅENDE FRÅGOR**

[wom_menopause_age]

MN290. Hur gammal var du när klimakteriesymtomen började?

❍ (Ålder) [yrs]____________ [yrs_other] ❍ Vet ej / vill ej svara [998]

[wom_menopause_symp]

MN300. Vilka av följande klimakteriesymtom hade / har du?

❑ Vallningar [flu]
❑ Torra eller sköra slemhinnor [muc]
❑ Humörsvängningar [moo]
❑ Hjärtrusning eller hjärtklappning [rus]
❑ Sömnsvårigheter [sle]
❑ Depression [dep]
❑
❍ Inget av dessa [996]
❍ Vet ej / vill ej svara [998]

[wom2_menopause_treat1]

MN310. Har du fått hormonbehandling p.g.a. dina klimakteriebesvär?

Ja
❑ Behandling med enbart östrogen [est]
❑ Östrogen i kombination med progesteron [epc]
❍ Nej [no]
❍ Vet ej / vill ej svara [998]

❑ Östrogen i kombination med gestaden [com]
❑ Behandling med enbart gestaden [ges]
❑ Annan behandling (vänligen ange) [oth]_____
[oth_other]

❖ **F. FÖR VARJE MARKERAT SVAR I MN310 VISA RESPEKTIVE FRÅGOR NEDAN**

Om "...enbart östrogen", visa MN320

[wom_menopause_estr_dur]

MN320. Hur länge har du fått behandling med enbart östrogen?

❍ (Antal år) [yrs]____________ [yrs_other] ❍ Vet ej / vill ej svara [998]
❍ Mindre än 1 år [1]

Om "östrogen i kombination med gestaden", visa MN330

[wom_menopause_comb_dur]

MN330. Hur länge har du fått östrogen i kombination med gestaden?

❍ (Antal år) [yrs]____________ [yrs_other] ❍ Vet ej / vill ej svara [998]
❍ Mindre än 1 år [1]

Om "...gestaden", visa MN340

[wom_menopause_gest_dur]

MN340. Hur länge har du fått behandling med enbart gestaden?

❍ (Antal år) [yrs]____________ [yrs_other] ❍ Vet ej / vill ej svara [998]
❍ Mindre än 1 år [1]

Om “annan behandling”, visa MN350

[wom_menopause_othe_dur]

MN350. Hur länge har du fått {annan behandling}?

❍ (Antal år) [yrs]____________ [yrs_other]   ❍ Vet ej / vill ej svara [998]
❍ Mindre än 1 år [1]

❖ **F. VILLKORET AVSLUTAS**

[wom_local]

MN360. Har du använt hormonmedicin för torra eller sköra slemhinnor?

Ja ❑
❑ Ovesterin [ove]
❑ Vagifem [vag]
❑ Annan (ange) [oth] ____ [oth_other]
❍ Nej [no]
❍ Vet ej / vill ej svara [998]

❖ **G. FÖR VARJE MARKERAD FRÅGA I MN360 VISA RESPEKTIVE FRÅGOR NEDAN**

Om “Ovesterin”, visa MN370

[wom_loc_dur1]

MN370. Hur länge har du använt Ovesterin?

❍ (Antal år) [yrs]____________ [yrs_other]   ❍ Vet ej / vill ej svara [998]
❍ Mindre än 1 år [1]

Om “Vagifem”, visa MN380

[wom_loc_dur2]

MN380. Hur länge har du använt Vagifem?

❍ (Antal år) [yrs]____________ [yrs_other] ❍ Vet ej / vill ej svara [998]
❍ Mindre än 1 år [1]

Om "annan", visa MN385

[wom_loc_dur3]

MN385. Hur länge har du använt {annan}?

❍ (Antal år) [yrs]____________ [yrs_other] ❍ Vet ej / vill ej svara [998]
❍ Mindre än 1 år [1]

- **E OCH G. VILLKORET AVSLUTAS**

[wom_osteo]

MN460. Har du fått diagnosen osteoporos/benskörhet?

❍ Ja [1] ❍ Nej [2]
❍ Vet ej / vill ej svara [998]

[wom_osteo_risk]

MN470.

	Ja [1]	Nej [2]	Vet ej / vill ej svara [998]
Är det någon som har osteoporos i din familj? [ost]	☐	☐	☐
{om mens: Har din mens upphört?} [men]	☐	☐	☐
Har du brutit höften, en ryggkota eller handled vid ett lätt fall? [fra]	☐	☐	☐

	Ja [1]	Nej [2]	Vet ej / vill ej svara [998]
Har du tagit kortisontabletter eller sprutor? [cor]	☐	☐	☐

❖ **D. VILLKORET AVSLUTAS**

Inkontinens

[wom_bladder_x]

MN390. Har du svårigheter med att kontrollera urinblåsan?

❍ Ja [1] ❍ Nej [2]
❍ Vet ej / vill ej svara [998]

❖ **H. MN390 OM SVARET ÄR "JA" VISA NEDANSTÅENDE FRÅGOR**

❖ **I. FÖR VARJE FRÅGA, OM A ÄR "JA" VISA B**

[wom_bladder1]

MN391. Upplever du att du behöver urinera/kissa ofta?

**A.** [1]		**B.** [2]
Ja [1]	Nej [2]	Hur mycket besvärar det dig?
☐	☐	❍ Inte alls [4]<br>❍ Bara lite [3]<br>❍ Något [2]<br>❍ Mycket [1]

[wom_bladder2]

MN392. Upplever du urinläckage vid urinträngning?

[1]		[2]
Ja [1]	Nej [2]	Hur mycket besvärar det dig?
☐	☐	❍ Inte alls [4]<br>❍ Bara lite [3]<br>❍ Något [2]<br>❍ Mycket [1]

[wom_bladder3]

MN393. Upplever du urinläckage i samband med fysisk aktivitet?

[1]		[2]
Ja [1]	Nej [2]	Hur mycket besvärar det dig?
☐	☐	❍ Inte alls [4]<br>❍ Bara lite [3]<br>❍ Något [2]<br>❍ Mycket [1]

[wom_bladder4]

MN394. Upplever du små mängder av urinläckage (droppar)?

[1]		[2]
Ja [1]	Nej [2]	Hur mycket besvärar det dig?
☐	☐	❍ Inte alls [4]<br>❍ Bara lite [3]<br>❍ Något [2]<br>❍ Mycket [1]

[wom_bladder5]

MN395. Upplever du svårighet att tömma blåsan?

[1]		[2]
Ja [1]	Nej [2]	Hur mycket besvärar det dig?
☐	☐	❍ Inte alls [4]<br>❍ Bara lite [3]<br>❍ Något [2]<br>❍ Mycket [1]

[wom_bladder6]

MN396. Upplever du värk eller obehag i nedre delen av buken eller underlivet?

[1]		[2]
Ja [1]	Nej [2]	Hur mycket besvärar det dig?
☐	☐	❍ Inte alls [4]<br>❍ Bara lite [3]

	[1]		[2]
			❍ Något [2]<br>❍ Mycket [1]

Hur ofta under den senaste månaden:

[wom_bladder7]

MN397. Har du känt ett starkt behov av att kissa med liten eller ingen förvarning?

❍ Inte alls [6]
❍ Mindre än 1 av 5 gånger [5]
❍ Mindre än halva tiden [4]
❍ Ungefär halva tiden [3]
❍ Mer än halva tiden [2]
❍ Nästan hela tiden [1]
❍ Vet ej / vill ej svara [998]

[wom_bladder8]

MN398. Har du behövt kissa inom mindre än 2 timmar efter det att du tömt urinblåsan?

❍ Inte alls [6]
❍ Mindre än 1 av 5 gånger [5]
❍ Mindre än halva tiden [4]
❍ Ungefär halva tiden [3]
❍ Mer än halva tiden [2]
❍ Nästan hela tiden [1]
❍ Vet ej / vill ej svara [998]

[wom_bladder9]

MN398.1. Har du behövt gå upp och kissa på natten?

❍ Inte alls [6]
❍ En gång [5]
❍ 2 gånger [4]
❍ 3 gånger [3]
❍ 4 gånger [2]
❍ 5 gånger eller fler [1]
❍ Vet ej / vill ej svara [998]

[wom_bladder10]

MN399. Har du upplevt obehag eller brännande smärta från urinblåsan?

❍ Inte alls [6]
❍ En gang [5]
❍ Några ganger [4]
❍ Ganska ofta [3]
❍ Nästan alltid [2]
❍ Vanligtvis [1]
❍ Vet ej / vill ej svara [998]

[wom_bladder11]

MN400. Har du upplevt att obehaget eller smärtan från urinblåsan upphört direkt efter att du tömt blåsan?

❍ Inte alls [6]
❍ Mindre än 1 av 5 gånger [5]
❍ Mindre än halva tiden [4]
❍ Ungefär halva tiden [3]
❍ Mer än halva tiden [2]
❍ Nästan hela tiden [1]
❍ Vet ej / vill ej svara [998]

❖ **H OCH I. VILLKOREN AVSLUTAS**

Levnadsvanor

Innehåll

Innehåll 1
Rökvanor 2
Rökningshistorik 2
Rökberoende 3
Nuvarande rökning och försök att sluta 4
Snus 6
Snushistorik 6
Snusberoende 7
Nuvarande snusning och försök att sluta 8
Vänner 9
Alkohol 11
Alkoholdebut 11
Nuvarande alkoholkonsumtion 11
Alkoholberoende 15
Alkoholmissbruk 17
Alkohol och hereditet 19
Vänner 20
Droganvändning 21
Nuvarande eller tidigare droganvändning 21
Drogmissbruk 23

Rökvanor

Rökningshistorik

[smo_onewhole]

SM10. Har du någonsin rökt en hel cigarett?

❍ Ja [1] ❍ Vet ej / vill ej svara [998]
❍ Nej [2]

❖ **A. LÄMNA MODULEN OM SM10 INTE ÄR "JA"**

[smo_onewhole_age]

SM20. Hur gammal var du första gången du rökte en hel cigarett?

❍ (Ålder) [yrs] ____________ [yrs_other] ❍ Vet ej / vill ej svara [998]

[smo_life]

SM30. Har du rökt mer än 100 cigaretter under hela din livstid?

❍ Ja [1] ❍ Vet ej / vill ej svara [998]
❍ Nej [2]

❖ **B. LÄMNA MODULEN OM SM30 INTE ÄR "JA"**

[smo_most]

SM40. När du rökte som mest, hur många cigaretter rökte du då?

❍ Mindre än 1 cigarett per månad [3]
❍ Mindre än 1 cigarett per dag men mer än en per månad [2]
❍ Minst 1 cigarett per dag [1]
❍ Vet ej / vill ej svara [998]

❖ **C. OM SM40 ÄR "MINST 1 CIGARETT PER DAG" VISA NEDAN FRÅGOR**

[smo_most_age]

SM50. Hur gammal var du när du började röka minst en cigarett per dag?

❍ (Ålder) [yrs] ____________ [yrs_other] ❍ Vet ej / vill ej svara [998]

[smo_years]

SM60. I hur många år under din livstid har du rökt minst en cigarett per dag?

❍ Mindre än 1 år [1]
❍ 1 till 5 år [2]
❍ 6 till 10 år [3]
❍ 11 till 15 år [4]
❍ 16 till 20 år [5]
❍ 21 till 25 år [6]
❍ 26 till 30 år [7]
❍ Mer än 30 år [8]
❍ Vet ej / vill ej svara [998]

[smo_amount]

SM70. Hur många cigaretter rökte du per dag under denna tid?

❍ (Antal) [nbr]____________ [nbr_other] ❍ Vet ej / vill ej svara [998]

Rökberoende

[smo_most_aftwake]

SM80. När du rökte som mest, hur snart efter det att du vaknat rökte du din första cigarett?

❍ Inom 5 minuter [1]
❍ 6 till 30 minuter [2]
❍ 31 till 60 minuter [3]
❍ Efter 60 minuter [4]
❍ Vet ej / vill ej svara [998]

[smo_most_moremorn]

SM90. När du rökte som mest, rökte du mer under de första timmarna efter det att du vaknat än under resten av dagen?

❍ Ja [1]
❍ Nej [2]
❍ Vet ej / vill ej svara [998]

[smo_most_hategveup]

SM100. När du rökte som mest, vilken cigarett ogillade du mest att avstå ifrån?

❍ Första cigaretten på morgonen [1] ❍ Annan [2]
❍ Vet ej / vill ej svara [998]

[smo_most_refrain]

SM110. När du rökte som mest, tyckte du att det var svårt att avstå från att röka på platser där det var förbjudet (skola, kyrka, bibliotek, bio etc.)?

❍ Ja [1] ❍ Vet ej / vill ej svara [998]
❍ Nej [2]

[smo_most_whnill]

SM120. När du rökte som mest, rökte du även om du var så sjuk att du låg i sängen större delen av dagen?

❍ Ja [1] ❍ Vet ej / vill ej svara [998]
❍ Nej [2]

Nuvarande rökning och försök att sluta

[smo_present]

SM130. Röker du nu?

❍ Ja [1] ❍ Vet ej / vill ej svara [998]
❍ Nej [2]

Om SM130 är "nej", visa SM135

Om SM130 är "ja", visa SM140

[smo_quitage]

SM135. Hur gammal var du när du slutade röka?

❍ (Ålder) [yrs] ____________ [yrs_other] ❍ Vet ej / vill ej svara [998]

[smo_presamount]

SM140. Hur många cigaretter per dag röker du nu?

❍ (Antal) [nbr]____________ [nbr_other] ❍ Vet ej / vill ej svara [998]

[smo_quittimes]

SM150. Hur många allvarliga försök att sluta röka har du gjort under hela din livstid?

❍ (Gånger) [tms]____________ [tms_other]

❍ Jag har inte försökt sluta [no]

❍ Vet ej / vill ej svara [998]

Om SM150 är ”antal gånger”, visa SM160

[smo_stayofftime]

SM160. Hur länge har du lyckats sluta röka som längst?

❍ Ungefär en dag [1]

❍ Ungefär en vecka [2]

❍ Ungefär en månad [3]

❍ Ungefär ett halvår [4]

❍ Ungefär ett år [5]

❍ Längre än ett år [6]

❍ Vet ej / vill ej svara [998]

❖ **C. VILKORET AVSLUTAS**

Snus

Snushistorik

[snu _tried]

SN10. Har du någonsin provat snus?

❍ Ja [1] ❍ Vet ej / vill ej svara [998]
❍ Nej [2]

❖ **D. LÄMNA MODULEN OM SN10 INTE ÄR "JA"**

[snu_age]

SN20. Hur gammal var du när du provade snus för första gången?

❍ (Ålder) [yrs] ____________ [yrs_other] ❍ Vet ej / vill ej svara [998]

[snu_life]

SN30. Har du använt mer än 5 dosor snus under din livstid?

❍ Ja [1] ❍ Vet ej / vill ej svara [998]
❍ Nej [2]

❖ **E. LÄMNA MODULEN OM SN30 INTE ÄR "JA"**

[snu_most]

SN40. När du snusade som mest, hur länge räckte en dosa?

❍ En månad eller mer [3]
❍ En vecka eller mer, men mindre än en månad [2]
❍ Mindre än en vecka [1]
❍ Vet ej / vill ej svara [998]

❖ **F. LÄMNA MODULEN OM SN40 RÄCKTE LÄNGRE ÄN "MINDRE ÄN EN VECKA"**

[snu_most_age]

SN50. Hur gammal var du när du började använda minst en dosa snus per vecka?

❍ (Ålder) [yrs] ____________ [yrs_other] ❍ Vet ej / vill ej svara [998]

[snu_years]

SN60. I hur många år i ditt liv använde du minst en dosa snus per vecka?

❍ Mindre än 1 år [1]
❍ 1 till 5 år [2]
❍ 6 till 10 år [3]
❍ 11 till 15 år [4]
❍ 16 till 20 år [5]
❍ 21 till 25 år [6]
❍ 26 till 30 år [7]
❍ Mer än 30 år [8]
❍ Vet ej / vill ej svara [998]

[snu_amount]

SN70. Hur många dagar räckte en dosa snus under denna tid?

❍ Mindre än 1 dag [1]
❍ 1 dag [2]
❍ 2 till 3 dagar [3]
❍ 4 till 7 dagar [4]
❍ Vet ej / vill ej svara [998]

Snusberoende

[snu_most_aftwake]

SN80. När du snusade som mest, hur snart efter det att du vaknat tog du din första portion snus?

❍ Inom 5 minuter [1]
❍ 6 till 30 minuter [2]
❍ 31 till 60 minuter [3]
❍ Efter 60 minuter [4]
❍ Vet ej / vill ej svara [998]

[snu_most_hategveup]

SN90. När du snusade som mest, vilken portion snus ogilla du mest att avstå från?

❍ Första snusen på morgonen [1] ❍ Annan [2]

❍ Vet ej / vill ej svara [998]

[snu_most_mouthtime]

SN100. När du snusade som mest i hur många minuter i genomsnitt behöll du en ny portion snus i munnen?

❍ Mindre än 10 minuter [1]
❍ 10 till 19 minuter [2]
❍ 20 till 29 minuter [3]
❍ 30 minuter eller längre [4]
❍ Vet ej / vill ej svara [998]

[snu_most1]

SN120. När du snusade som mest:

	Ja [1]	Nej [2]	Vet ej / vill ej svara [998]
Kände du en stark längtan efter snus när du varit utan det i 2 timmar? [1]	☐	☐	☐
Snusade du mer på morgonen än under resten av dagen? [2]	☐	☐	☐
Var det svårt för dig att inte snusa i olämpliga situationer? [3]	☐	☐	☐
Snusade du även då du var så sjuk att du låg i sängen större delen av dagen? [4]	☐	☐	☐

Nuvarande snusning och försök att sluta

[snu_present]

SN130. Snusar du nu?

❍ Ja [1]
❍ Nej [2]
❍ Vet ej / vill ej svara [998]

Om SN130 är "nej", visa SN135

Om SN130 är "ja", visa SN140

[snu_quitage]

SN135. Hur gammal var du när du slutade snusa?

❍ (Ålder) [yrs] ____________ [yrs_other] ❍ Vet ej / vill ej svara [998]

[snu_presamount]

SN140. Hur många dagar räcker en dosa snus?

❍ 1 dag [1] ❍ 4 till 7 dagar [3]
❍ 2 till 3 dagar [2] ❍ Vet ej / vill ej svara [998]

[snu_quittimes]

SN150. Hur många allvarliga försök har du gjort att sluta snusa under hela din livstid?

❍ (Antal gånger) [tms]____________ [tms_other] ❍ Jag har inte försökt sluta [no]
❍ Vet ej / vill ej svara [998]

Om SN150 är ”antal gånger”, visa SN160

[snu_stayofftime]

SN160. Hur länge har du lyckats sluta snusa som längst?

❍ Ungefär en dag [1] ❍ Ungefär ett halvår [4]
❍ Ungefär en vecka [2] ❍ Ungefär ett år [5]
❍ Ungefär en månad [3] ❍ Längre än ett år [6]
❍ Vet ej / vill ej svara [998]

Vänner

Visa SM170, om svaret på SC30 inte är "nej" och inte "vet ej/vill ej svara" och ålder >= 18

[smo_livesmoke]

SM170. Hur många av de som du bor tillsammans med röker?

❍ Ingen [1] ❍ 2 personer [3]
❍ 1 person [2] ❍ Mer än 2 personer [4]
❍ Vet ej / vill ej svara [998]

[smo_friendsmoke]

SM180. Hur många av dina tre bästa vänner som du inte bor tillsammans med röker?

❍ Ingen [1] ❍ Två [3]
❍ En [2] ❍ Alla tre [4]
❍ Vet ej / vill ej svara [998]

Visa SN170, om svaret på SC30 inte är "nej" och inte "vet ej/vill ej svara" och ålder >= 18

[snu_livesnus]

SN170. Hur många av de personer du bor tillsammans med snusar?

❍ Ingen [1] ❍ 2 personer [3]
❍ 1 person [2] ❍ Mer än 2 personer [4]
❍ Vet ej / vill ej svara [998]

[snu_friendsnus]

SN180. Hur många av dina tre bästa vänner som du inte bor tillsammans med snusar?

❍ Ingen [1] ❍ Två [3]
❍ En [2] ❍ Alla tre [4]
❍ Vet ej / vill ej svara [998]

Alkohol

Alkoholdebut

[alc_fulldrink]

AL10. Har du någonsin druckit alkohol?

❍ Ja [1]   ❍ Vet ej / vill ej svara [998]
❍ Nej [2]

❖ **G. LÄMNA MODULEN OM AL10 INTE ÄR "JA"**

[alc_fulldrink_age]

AL20. Hur gammal var du första gången du drack alkohol?

❍ (Ålder) [yrs] ____________ [yrs_other]   ❍ Vet ej / vill ej svara [998]

Nuvarande alkoholkonsumtion

[alc_drink_frq]

AL30. Om du tänker på de senaste 12 månaderna, hur ofta har du druckit alkohol?

❍ Aldrig [6]
❍ En gång i månaden eller mindre ofta [5]
❍ 2 till 3 gånger i månaden [4]
❍ 1 gång i veckan [3]
❍ 2 till 3 gånger i veckan [2]
❍ 4 gånger i veckan eller oftare [1]
❍ Vet ej / vill ej svara [998]

❖ **H. LÄMNA MODULEN OM AL10 ÄR "ALDRIG".**

- **OM AL30 ÄR "2 TILL 3 GÅNGER I MÅNADEN" ELLER MINDRE OFTA, ANVÄND FORMULERINGEN "VARJE MÅNAD" I NEDANSTÅENDE FRÅGOR, ANNARS ANVÄNDS FORMULERINGEN "VARJE VECKA"**

[alc_drink_wh]

AL40. Dricker du någon av följande alkoholhaltiga drycker {varje månad, varje vecka}? (Markera alla aktuella)

Ja
❑ Folköl (3,5 %) [foll]
❑ Mellanöl (4,5 %) [mell]
❑ Starköl (5 - 7%) [stro]
❑ Extra stark öl (> 7%) [very]
❑ Cider (5 %) [cide]
❑ Vin [wine]
❑ Sprit [spir]
❑ Alkoläsk [pops]
❑
❍ Nej [no]
❍ Vet ej / vill ej svara [998]

- **I. LÄMNA MODULEN OM AL10 ÄR "NEJ"**

- **J. FÖR VARJE MARKERAT SVAR I AL40 VISA MOTSVARANDE SVARSALTERNATIV NEDAN**

[alc_drink_beer]

AL50. Hur många flaskor eller burkar av följande alkoholhaltiga drycker dricker du en vanlig {månad, vecka}?

	33 cl [33cl]	50 cl [50cl]
Folköl [folk]	_______	_______
Mellanöl [mell]	_______	_______
Starköl [stro]	_______	_______
Extra stark öl [very]	_______	_______
Cider [cide]	_______	_______
Alkoläsk [pops]	_______	_______

- **J. VILLKORET AVSLUTAS**

Om "vin" är markerat i AL40, visa AL60

[alc_drink_wine_wh]

AL60. Dricker du mer än en flaska vin (75 cl) per {månad, vecka}?

❍ Ja [1] ❍ Vet ej / vill ej svara [998]
❍ Nej [2]

Om AL60 är "nej", visa AL70 och AL90

[alc_drink_wine_glasses]

AL70. Hur många glas vin dricker du per {månad, vecka}?

❍ 1 [1] ❍ 4 [4]
❍ 2 [2] ❍ 5 eller fler [5]
❍ 3 [3] ❍ Vet ej / vill ej svara [998]

Om AL60 är "ja", visa AL80 och AL90

[alc_drink_wine_bottles]

AL80. Hur många flaskor vin dricker du per {månad, vecka}?

❍ 2 flaskor [1] ❍ 5 flaskor eller fler [3]
❍ 3 till 4 flaskor [2] ❍ Vet ej / vill ej svara [998]

[alc_drink_wine_mix]

AL90. Vilken mängd rött (inklusive rosévin) respektive vitt vin (inklusive mousserande vin) dricker du?

❍ 100% rödvin [1] ❍ 25% rödvin och 75% vitt vin [4]
❍ 75% rödvin och 25% vitt vin [2] ❍ 100 % vitt vin [5]
❍ 50% rödvin och 50% vitt vin [3] ❍ Vet ej / vill ej svara [998]

Om "sprit" är markerat i AL40, visa AL100 och AL110

[alc_drink_spir]

AL100. Hur mycket sprit innehåller din drink i genomsnitt?

❍ 4 cl (1 liten snaps) [1]
❍ 6 cl (1 stor snaps) [2]
❍ 8 cl [3]
❍ 10 cl [4]
❍ 12 cl [5]
❍ Vet ej / vill ej svara [998]

[alc_drink_spir_drink]

AL110. Hur många sådana drinkar dricker du under en normal {månad, vecka}?

❍ (Antal) [nbr]___________ [nbr_other] ❍ Vet ej / vill ej svara [998]

❖ **K. FÖR VARJE MARKERAT SVAR I AL40, VISA MOTSVARANDE SVARSALTERNATIV NEDAN**

[alc_drink]

AL115. Hur mycket av dessa drycker dricker du på helgerna (Fredag till Söndag) {under en normal vecka, under en normal månad}?

	Din total {per månad, per vecka} **"datorn fyller i"** [sum]	Ange total per helg [weekend]
Folköl [folk]	_______	_______
Mellanöl [mell]	_______	_______
Starköl [stro]	_______	_______
Extra stark öl [very]	_______	_______
Cider [cide]	_______	_______
Vin [wine]	_______	_______
Sprit [spir]	_______	_______
Alkoläsk [pops]	_______	_______

❖ **K. VILLKORET AVSLUTAS**

Alkoholberoende

[alc_12mnts_frqocc]

AL120. Hur ofta har du druckit {män: 5, kvinnor: 4} eller fler "standarddrinkar" vid samma tillfälle under de senaste 12 månaderna?

❍ Aldrig [5]
❍ Mindre än en gång i månaden [4]
❍ Varje månad [3]
❍ Varje vecka [2]
❍ Dagligen eller nästan dagligen [1]
❍ Vet ej / vill ej svara [998]

❖ **BILD PÅ "STANDARDDRINKAR"**

❖ **L. RÄKNA FRAM ANTALET "STANDARDDRINKAR" FRÅN AL40 ALKOHOLHALTIGA DRYCKER. LÄMNA MODULEN OM [VARJE VECKA <14 STANDARDDRINKAR FÖR MÄN OCH <9 STANDARDDRINKAR FÖR KVINNOR] ELLER AL120 ÄR "VARJE MÅNAD" ELLER OFTARE**

[alc_audit]

AL130. Hur ofta under det senaste året:

	Aldrig [5]	Mindre än en gång i månaden [4]	Varje månad [3]	Varje vecka [2]	Dagligen eller nästan dagligen [1]	Vet ej / vill ej svara [998]
Har du inte kunnat sluta dricka sedan du börjat? [4]	☐	☐	☐	☐	☐	☐
Har du låtit bli att göra något som du borde för att du	☐	☐	☐	☐	☐	☐

	Aldrig [5]	Mindre än en gång i månaden [4]	Varje månad [3]	Varje vecka [2]	Dagligen eller nästan dagligen [1]	Vet ej / vill ej svara [998]
drack? [5]						
Har du behövt en "drink" på morgonen efter mycket drickande dagen innan? [6]	☐	☐	☐	☐	☐	☐
Har du haft skuldkänslor eller samvetskval på grund av ditt drickande? [7]	☐	☐	☐	☐	☐	☐
Har du inte kommit ihåg vad som hände kvällen innan på grund av ditt drickande? [8]	☐	☐	☐	☐	☐	☐

[alc_audit_9]

AL135. Har en släkting, vän, doktor eller någon annan sjukvårdspersonal oroat sig över ditt drickande eller antytt att du borde minska ned på det?

❍ Nej [3]
❍ Ja, men inte under det senaste året [2]
❍ Ja, under det senaste året [1]
❍ Vet ej / vill ej svara [998]

[alc_audit_10]

AL137. Har du eller någon annan blivit skadad på grund av ditt drickande?

❍ Nej [3]
❍ Ja, men inte under det senaste året [2]
❍ Ja, under det senaste året [1]
❍ Vet ej / vill ej svara [998]

Alkoholmissbruk

[alc_addicted]

AL140. Följande frågor handlar om ditt drickande under de senaste 12 månaderna:

	Ja [1]	Nej [2]	Vet ej / vill ej svara [998]
Behövde du dricka en större mängd alkohol för att få någon effekt? [tole]	☐	☐	☐
Har du försökt sluta eller skära ned på ditt drickande men inte lyckats? [con1]	☐	☐	☐
Fanns det perioder då du drack så mycket eller då det gick åt så mycket tid att återhämta dig så att du hade litet tid för något annat? [reco]	☐	☐	☐
Fanns det en period när du kraftigt drog ned på viktiga aktiviteter på grund av ditt drickande – t.ex. att sporta, arbeta eller träffa släkt och vänner? [acti]	☐	☐	☐
Fanns det tillfällen då du visste att du hade allvarliga fysiska eller känslomässiga problem som orsakades av ditt drickande och ändå fortsatte att dricka? [con2]	☐	☐	☐

[alc_socint]

AL150. Under de senaste 12 månaderna, fanns det en period då ditt drickande eller din baksmälla upprepade gånger hindrade dig från att ta ansvar på arbetet, i skolan eller i hemmet?

❍ Ja [1] ❍ Vet ej / vill ej svara [998]
❍ Nej [2]

Om “ja”, visa AL170

[alc_socint_cont]

AL170. Fortsatte du att dricka fastän ditt drickande orsakade dessa probem?

❍ Ja [1] ❍ Vet ej / vill ej svara [998]
❍ Nej [2]

[alc_argum]

AL180. Under de senaste 12 månaderna, fanns det en period då ditt drickande orsakade upprepade bråk med familj, vänner, grannar eller arbetskamrater?

❍ Ja [1] ❍ Vet ej / vill ej svara [998]
❍ Nej [2]

Om "ja", visa AL190

[alc_argum_cont]

AL190. Fortsatte du att dricka fastän ditt drickande orsakade bråk med dessa människor?

❍ Ja [1] ❍ Vet ej / vill ej svara [998]
❍ Nej [2]

[alc_hurtrisk]

AL200. Under de senaste 12 månaderna har du upprepade gånger varit påverkad av alkohol i situationer då det fanns risk för att du eller någon annan kunde bli skadad – t. ex. att du körde bil eller använde en maskin?

❍ Ja [1] ❍ Vet ej / vill ej svara [998]
❍ Nej [2]

[alc_treat]

AL210. Har du någonsin sökt behandling eller hjälp för ditt drickande?

❍ Ja [1] ❍ Vet ej / vill ej svara [998]
❍ Nej [2]

Om AL210 är "ja", visa AL220

[alc_treatget]

AL220. Var sökte du hjälp? (Markera alla aktuella)

❑ Läkare [doc]
❑ Kurator eller annan sjukvårdspersonal [couns]
❑ Präst eller pastor [prie]
❑ Anonyma Alkoholister [self]
❑ Annan självhjälpsgrupp [alctre]
❑ Annan [oth]

❍ Vet ej / vill ej svara [998]

Alkohol och hereditet

[alc_heredity]

AL230. Är det någon av dina nära släktingar som har eller har haft alkoholproblem?

	Nej [2]	Ja [1]	Gäller inte mig [994]	Vet ej / vill ej svara [998]
Far [1]	☐	☐	☐	☐
Mor [2]	☐	☐	☐	☐
Bröder eller systrar [3]	☐	☐	☐	☐
Halvbröder eller halvsystrar [4]	☐	☐	☐	☐
Farfar [5]	☐	☐	☐	☐
Farmor [6]	☐	☐	☐	☐
Morfar [7]	☐	☐	☐	☐
Mormor [8]	☐	☐	☐	☐
Barn [9]	☐	☐	☐	☐

Vänner

[alc_peer]

AL240. Hur många av dina tre bästa vänner dricker alkohol?

❍ Ingen [1]
❍ En [2]
❍ Två [3]
❍ Alla tre [4]
❍ Vet ej / vill ej svara [998]

Droganvändning

Nuvarande eller tidigare droganvändning

[ill_tried]

IL10. Följande frågor handlar om droganvändning utöver medicinskt förskrivna droger.

Har du någonsin använt någon annan drog förutom tobak eller alkohol av någon av följande anledningar?

- för att bli hög
- för att må bättre
- för att ändra sinnesstämning
- för att höja prestationen
- för att bygga muskler

❍ Ja [1] ❍ Nej [2]
❍ Vet ej / vill ej svara [998]

❖ **N. LÄMNA MODULEN OM IL10 ÄR "NEJ"**

[ill_tried_whc]

IL20. Har du någonsin provat någon av följande droger? (Markera alla aktuella)

Droger
❑ Cannabis, marijuana, hash [whc]
❑
❑ Hallucinogena svampar (Psilocybin, Psilocin) [hal]
❑ LSD [lsd]
❑ Ecstasy [ecs]
❑ GHB [ghb]
❑
❑ Amfetamin [amp]
❑ Kokain [coc]
❑
❑ Heroin [her]
❑ Opium [opi]

Mediciner
❑ Codein, Citodon, Treo Comp, Panocod [cod]
❑ Tramadol, Tradolan, Tiparol, Nobligan [tra]
❑
❑ Sobril, Oxascand, Stesolid, Diazepam, Xanor, Alprazolam [sob]
❑ Stilnoct, Zolpidem, Imovan, Zopiklon [sti]
❑
❑ Metylfenidat (Ritalin, Concerta)
❑
❑ Morfin
❑ Subutex, Suboxone

❍ Ingen av dessa
❑
❍ Vet ej / vill ej svara

❑ ❑
❑ Tillväxthormon [gro] ❑ Annan drog eller medicin
❑ Anabola steroider [ana]

- **O. LÄMNA MODULEN OM IL10 ÄR "INGEN AV DESSA"**
- **P. FÖR VARJE MARKERAT SVAR I IL20 VISA NEDANSTÅENDE FRÅGOR**

[ill_tried_once]

IL25. Har du använt {markerad drog} mer än en gång?

❍ Ja [1] ❍ Vet ej / vill ej svara [998]
❍ Nej [2]

[ill_tried_age]

IL30. Hur gammal var du {första gången, när} du provade {markerad drog}?

❍ (Ålder) [yrs]___________ [yrs_other] ❍ Vet ej / vill ej svara [998]

Om IL25 är "ja", visa IL40 och IL50

[ill_pres]

IL40. Använder du {markerad drog} nu?

❍ Ja [1] ❍ Vet ej / vill ej svara [998]
❍ Nej [2]

[ill_frq]

IL50. Hur ofta {använder, använde} du {markerad drog} när du {använder, använde} det som mest?

❍ 1 gång i månaden eller mindre ofta [1]
❍ 2 till 4 gånger i månaden [2]
❍ 2 till 3 gånger i veckan [3]
❍ 4 gånger i veckan eller oftare [4]
❍ Vet ej / vill ej svara [998]

- **P. VILLKORET AVSLUTAS**

❖ **Q. LÄMNA OM IL40 ÄR "NEJ" FÖR ALLA MARKERADE DROGER I IL20**

Om IL20 har två eller fler droger markerade och IL40 är "ja" för båda drogerna, visa IL60

[ill_severalocc]

IL60. Hur ofta använder du mer än en typ av drog vid ett och samma tillfälle?

❍ Aldrig [5]
❍ En gång i månaden eller mindre ofta [4]
❍ 2 till 4 gånger i månaden [3]
❍ 2 till 3 gånger i veckan [2]
❍ 4 gånger i veckan eller oftare [1]
❍ Vet ej / vill ej svara [998]

[ill_amounttypday]

IL70. En typisk dag då du tar droger, hur många gånger om dagen tar du någon drog?

❍ 1 till 2 gånger [1]
❍ 3 till 4 gånger [2]
❍ 5 till 6 gånger [3]
❍ 7 gånger eller fler [4]
❍ Vet ej / vill ej svara [998]

Drogmissbruk

[ill_druguse1]

IL80. Hur ofta under det senaste året har du:

	Aldrig [4]	Varje månad [3]	Varje vecka [2]	Dagligen eller nästan dagligen [1]	Vet ej / vill ej svara [998]
Upplevt att din längtan efter droger varit så stark att du inte kunnat stå emot? [fel]	☐	☐	☐	☐	☐
Inte kunnat sluta ta droger när du börjat? [sto]	☐	☐	☐	☐	☐
Tagit droger och sedan låtit	☐	☐	☐	☐	☐

	Aldrig [4]	Varje månad [3]	Varje vecka [2]	Dagligen eller nästan dagligen [1]	Vet ej / vill ej svara [998]
bli att göra något som du borde ha gjort? [neg]					
Behövt ta någon drog på morgonen efter stort drogintag dagen innan? [mor]	☐	☐	☐	☐	☐
Haft skuldkänslor eller dåligt samvete på grund av att du använt droger? [gui]	☐	☐	☐	☐	☐
Varit kraftigt påverkad av droger? [inf]	☐	☐	☐	☐	☐

[ill_worried]

IL90. Har någon oroat sig för att du använder droger eller sagt till dig att du bör sluta med droger?

❍ Nej [3]
❍ Ja, men inte under det senaste året [2]
❍ Ja, under det senaste året [1]
❍ Vet ej / vill ej svara [998]

[ill_injuried]

IL100. Har du eller någon annan blivit skadad på grund av din droganvändning?

❍ Nej [3]
❍ Ja, men inte under det senaste året [2]
❍ Ja, under det senaste året [1]
❍ Vet ej / vill ej svara [998]

Hälsohistoria

Innehåll

Innehåll ... 1
Tandhälsa ... 3
  Munhälsa ... 3
  Fyllningar och tandsjukdom ... 4
Infektioner ... 6
  Vanliga infektioner ... 6
  Sjukledighet och mediciner ... 7
Sjukdomar ... 9
Ätbeteende ... 19
  Inledande frågor ... 19
  Vikt och kroppsform ... 21
  Anorexi ... 22
  Hetsätning ... 25
  Självrening ... 27
Reflux, IBS ... 32
Sömn ... 36
  Sömnkvalitet och sovtid ... 36
  Sömnbesvär, snarkning, sömnapné ... 38
  Sömnhjälpmedel ... 40
  Sömnstörningar ... 40
Huvudvärk ... 42

Daglig och återkommande huvudvärk 42
Huvudvärkssymtom 42
Livskvalitet, diagnos, medicinering 44
Hörsel och tal 46
Självestimering av hörsel 46
Hörlursanvändning 46
Tinnitus 47
Yrsel 47
Hörselnedsättning 48
Tal 49

Tandhälsa

Munhälsa

Visa DE10, om ålder >= 11

[den_last]

DE10. När var du senast hos tandläkaren?

❍ Mindre än 1 år sedan [1] ❍ 3 till 5 år sedan [3] ❍ Jag går aldrig dit [5]
❍ 1 till 2 år sedan [2] ❍ Mer än 5 år sedan [4] ❍ Vet ej / vill ej svara [998]

[den_brush]

DE20. Hur ofta borstar {ditt barn, du} tänderna?

❍ 2 gånger om dagen [1] ❍ Mindre än 1 gång om dagen [3]
❍ 1 gång om dagen [2] ❍ Vet ej / vill ej svara [998]

[den_type]

DE30. Vilken typ av tandborste använder {ditt barn, du} vanligtvis?

❍ Vanlig tandborste [1] ❍ Elektrisk tandborste [2]
❍ Vet ej / vill ej svara [998]

[den_paste]

DE40. Brukar {ditt barn, du} använda tandkräm regelbundet?

❍ Ja [1] ❍ Vet ej / vill ej svara [998]
❍ Nej [2]

[den_floss]

DE50. Brukar {ditt barn, du} använda tandtråd regelbundet?

❍ Ja [1] ❍ Vet ej / vill ej svara [998]
❍ Nej [2]

Visa DE60, om ålder mellan 4 och 14

[den_milk]

DE60. Har {ditt barn, du} tappat {sina, dina} mjölktänder?

❍ Ja, alla mjölktänder [1]
❍ Ja, några mjölktänder [2]
❍ Nej [3]
❍ Vet ej / vill ej svara [998]

Visa DE65, om ålder >= 11

[den_brace]

DE65. Har {ditt barn, du} tandställning?

❍ Ja [1]
❍ Nej [2]
❍ Vet ej / vill ej svara [998]

Fyllningar och tandsjukdom

Visa DE70, om ålder >= 4

[den2_fillings]

DE70. Hur många fyllningar har {ditt barn, du}?

❍ Inga [none]
❍ 1 [1]
❍ 2 [2]
❍ 3 [3]
❍ 4 [4]
❍ 5 [5]
❍ 6 till 7 [6]
❍ 8 till 10 [7]
❍ 11 till 15 [8]
❍ 16 till 20 [9]
❍ Fler än 20 [10]
❍ Vet ej / vill ej svara [998]

Visa DE80, om ålder >= 18

[den_periodon]

DE80. Har din tandläkare sagt att du har en parodontal sjukdom (infektion i tandköttet/tandlossning)?

❍ Ja [1] ❍ Vet ej / vill ej svara [998]
❍ Nej [2]

Infektioner

Vanliga infektioner

[inf_tickbite]

IF10. Har {ditt barn, du} fått något fästingbett under de senaste 12 månaderna?

❍ Ja [1] ❍ Vet ej / vill ej svara [998]
❍ Nej [2]

[inf2_fungal]

IF20. Har {ditt barn, du} haft någon form av svampinfektion (t.ex. under naglar {, i underlivet} eller i munnen) under de senaste 12 månaderna?

❍ Ja (vänligen ange) [1] ____ [1_other] ❍ Vet ej / vill ej svara [998]
❍ Nej [2]

Om enkäten besvaras av vuxen, visa IF30 (barn får frågor om djur i annan modul)

[inf_pets]

IF30. Finns det några husdjur i {ditt barns, ditt} hem?

Ja
❑ Katt [cat]
❑ Hund [dog]
❑ Gnagare [rod]
❑ Fåglar [bir]
❑ Annat (vilket) [oth]____________ [oth_other]
❍
❍ Nej, inga husdjur [no]
❍ Vet ej / vill ej svara [998]

[inf_illness]

IF40. Har {ditt barn, du} under de senaste 12 månaderna blivit hastigt sjuk (dvs. inom några timmar) med feber, halsont, snuva, hosta eller muskelvärk?

❍ Ja [1] ❍ Vet ej / vill ej svara [998]
❍ Nej [2]

Om IF40 är ”ja”, visa IF45

[inf_illness_time]

IF45. Hur många gånger har {ditt barn, du} haft den typen av symtom under de senaste 12 månaderna?

❍ 1 gång [1]
❍ 2 gånger [2]
❍ 3 gånger [3]
❍ Mer än 3 gånger (ange) [4]____ [4_other]
❍ Vet ej / vill ej svara [998]

Sjukledighet och mediciner

[inf2_athome]

IF50. Har {ditt barn, du} varit hemma från {dagis, skola, arbete} eller annan daglig sysselsättning på grund av förkylning, influensa eller maginfluensa under de senaste 12 månaderna?

Ja
❍ 1 gång [1]
❍ 2 gånger [2]
❍ 3 gånger [3]
❍ Mer än 3 gånger (ange) [4]____ [4_other]
❍ Nej [no]
❍ Vet ej / vill ej svara [998]

Om IF50 är ”1 gång” eller mer, visa IF60

[inf2_athome_tottime]

IF60. Hur lång tid {, sammanlagt} var {ditt barn, du} hemma under de senaste 12 månaderna på grund av förkylning, influensa eller maginfluensa?

❍ Mindre än 1 vecka [1]
❍ 1 till 2 veckor [2]
❍ 3 till 4 veckor [3]
❍ Mer än 4 veckor [4]
❍ Vet ej / vill ej svara [998]

[inf2_cure]

IF70. Har {ditt barn fått, du tagit} medicin för förkylning, influensa eller maginfluensa under de senaste 12 månaderna?

Ja
❍ 1 gång [1]
❍ 2 till 3 gånger [2]
❍ 4 till 5 gånger [3]
❍ Fler än 5 gånger [4]
❍ Nej [no]
❍ Vet ej / vill ej svara [998]

Om IF70 är "1 gång" eller fler, visa IF75

[inf2_cure_med]

IF75. Vilken eller vilka mediciner {fick ditt barn, tog du}? (Markera alla aktuella)

❑ Febernedsättande medicin [fev]
❑ Antibiotika [ant]
❑ Medicin mot influensa (t.ex. Tamiflu eller Relenza) [flu]
❑ Annat läkemedel (ange) [oth]_____ [oth_other]
❍ Vet ej / vill ej svara [998]

[inf_vaccine]

IF65. Har {ditt barn, du} fått vaccin mot influensa under detta eller föregående år?

❑ Ja, under de senaste 12 månaderna [1]
❑ Ja, för 1 till 2 år sedan [2]
❍ Nej [3]
❍ Vet ej / vill ej svara [998]

Sjukdomar

[dis_1]

DS10. Vilka av följande sjukdomar har du för närvarande eller har du haft?

Hjärta / kärl
❑ Högt blodtryck [hibl]
❑ Höga blodfetter eller kolesterol [hift]
❑ Kärlkramp (Angina pectoris) [angp]
❑ Hjärtattack (hjärtinfarkt) [hear]
❑ Ojämn hjärtrytm [tach]
❑ Blodpropp [bloo]
Lungsjukdom
❑ Astma [asth]
❑ KOL, kronisk obstruktiv lungsjukdom [copd]
Hud
❑ Munsår (herpes) [lips]
❑ Akne [acne]
❑ Eksem [ecze]
❑ Psoriasis [psor]
Endokrina systemet
❑ Diabetes [diab]
❑ Sköldkörtelsjukdom (t.ex. struma) [thyr]
Mage
❑ Glutenintolerans (Celiaki) [spru]
❑ Laktosintolerans [lac]
❑ Halsbränna, sura uppstötningar [refl]
❑ Magsår [stom]
❑ Magkatarr [gas]
❑ Problem med gallblåsan [gall]
❑ Irritabel tarm (colon irritabile, IBS) [colo]
❑ Crohns sjukdom [chro]
❑ Ulcerös kolit [ulce]
❑ Avföringsinkontinens [ana]
Urologi
❑ Återkommande urinvägs-infektioner [urin]
❑ {om man: Prostatabesvär} [pros]
❑ Njursten [kidn]
❑
❍ Ingen av dessa [996]
❍ Vet ej / vill ej svara [998]
❑
❑

❖ **A. FÖR VARJE MARKERAT SVAR I DS10 VISA MOTSVARANDE FRÅGOR NEDAN**

Om "ojämn hjärtrytm"är markerat, visa DS11

[dis_1_tach_diag]

DS11. Har en läkare diagnostiserat ojämn hjärtrytm?

❍ Ja [1]
❍ Vet ej / vill ej svara [998]
❍ Nej [2]

Om "blodpropp", visa DS12, DS13 och DS14

[dis_1_bloo_where]

DS12. Var i kroppen har (hade) du en blodpropp?

❑ I ett ben [1]
❑ I lungan/lungorna [2]
❑ På ett annat ställe [3]
❍ Vet ej / vill ej svara [998]

[dis_1_bloo_repeated]

DS13. Har du haft flera blodproppar?

❍ Ja [1]
❍ Nej [2]
❍ Vet ej / vill ej svara [998]

[dis_1_waran]

DS14. Får du (fick du) medicinen Waran (blodförtunnande medel) för blodproppen?

❍ Ja [1]
❍ Nej [2]
❍ Vet ej / vill ej svara [998]

[dis_1_thyr_diag]

DS15. Vilken typ av struma eller sköldkörtelsjukdom har (hade) du?

❍ Giftstruma (ökad mängd sköldkörtelhormon, hyperthyroidism) [1]
❍ Låg mängd sköldkörtelhormon (hypothyroidism) [2]
❍ Annan struma [3]
❍ Vet ej / vill ej svara [998]

Om "halsbränna eller sura uppstötningar/reflux", visa DS16 och DS18

[dis_1_refl_diag]

DS16. Har en läkare ställt diagnosen halsbränna eller sura uppstötningar?

❍ Ja [1]
❍ Nej [2]
❍ Vet ej / vill ej svara [998]

[dis_1_pros_diag]

DS17. Har en läkare diagnostiserat dina prostatabesvär?

❍ Ja [1]
❍ Nej [2]
❍ Vet ej / vill ej svara [998]

[dis_1_kidn_diag]

DS18. Har en läkare diagnostiserat dina njurstensbesvär?

❍ Ja [1] ❍ Vet ej / vill ej svara [998]
❍ Nej [2]

[dis_1_gall_diag]

DS19. Har en läkare diagnostiserat dina problem med gallblåsan?

❍ Ja [1] ❍ Vet ej / vill ej svara [998]
❍ Nej [2]

[dis_1_stom_diag]

DS20. Har en läkare ställt diagnosen magsår?

❍ Ja [1] ❍ Vet ej / vill ej svara [998]
❍ Nej [2]

[dis_1_asth_diag]

DS21. Har en läkare ställt diagnosen astma?

❍ Ja [1] ❍ Vet ej / vill ej svara [998]
❍ Nej [2]

[dis_1_copd_diag]

DS22. Har en läkare ställt diagnosen KOL (kroniskt obstruktiv lungsjukdom)?

❍ Ja [1] ❍ Vet ej / vill ej svara [998]
❍ Nej [2]

[dis_1_psor_diag]

DS23. Har en läkare ställt diagnosen psoriasis?

❍ Ja [1] ❍ Vet ej / vill ej svara [998]
❍ Nej [2]

[dis_1_ecze_diag]

DS24. Har en läkare ställt diagnosen eksem?

❍ Ja [1] ❍ Vet ej / vill ej svara [998]
❍ Nej [2]

Om "diabetes", visa DS25 och DS26

[dis_1_diabtype]

DS25. Vilken diabetestyp har (hade) du?

❍ Barndiabetes (typ I) [1] ❍ Vuxendiabetes (typ II) [2] ❍ Vet ej / vill ej svara [998]

[dis_1_diatreat]

DS26. Vilken behandling har du för din diabetes?

❑ Insulinsprutor [ins] ❑ Diet [die]
❑ Tabletter [tab] ❑ Annan [oth]

Om "avföringsinkontinens", visa DS27

[dis_1_ana_problems]

DS27. Hur ofta besväras du av följande problem med avföringsinkontinens?

	Aldrig eller nästan aldrig [4]	Ibland [3]	Vanligtvis [2]	Alltid [1]	Vet ej / vill ej svara [998]
Uppsvälld mage eller gaser [fla]	☐	☐	☐	☐	☐
Läckage av lös avföring [liq]	☐	☐	☐	☐	☐
Läckage av fast avföring [sol]	☐	☐	☐	☐	☐
Behov av att använda blöja vid avföringsinkontinens [pad]	☐	☐	☐	☐	☐
Ändring av livsstil [lif]	☐	☐	☐	☐	☐

❖ **A. VILLKORET AVSLUTAS**

[dis_2]

DS30. Vilka av följande sjukdomar har du för närvarande eller har du haft?

Muskuloskeletal sjukdom
❑ Ischias [scia]
❑ Ständig ryggvärk (kronisk) [back]
❑ Ständig värk i axlar (kronisk) [shou]
❑ Artros (t.ex i höfter, knän eller fingrar) [artr]
❑ Ledgångsreumatism [rheu]
❑ SLE (Lupus) [sle]
❑ Fibromyalgi [fibr]
Neurologi
❑ Läs- och skrivsvårigheter (dyslexi) [dysl]
❑ Svårighet att tala, hitta ord eller förstå tal [dysp]
❑ Migrän [migr]
❑ Hortons sjukdom [hort]
❑ Menières sjukdom [meni]
❑ Epilepsi [epil]
❑ MS (multipel skleros) [ms]
Barn och ungdom
❑ Vattkoppor [chic]
❑ Körtelfeber (mononukleos) [glan]
Psykiatri
❑ Utbrändhet [burn]
❑ Depression [depr]
❑ Bipolär sjukdom [bipo]
❑ Ångestsyndrom [gad]
❑ Panikångest [pani]
❑ Agorafobi [agor]
❑ Social fobi [soci]
❑ Tvångssyndrom [ocd]
❑ Posttraumatiskt stressyndrom [ptsd]
❑ Schizofreni [schi]
❑ Schizoaffektivt syndrome [scha]
❑ Aspergers syndrome [aspe]
❑ Autism [auti]
❑ Tourettes syndrome [tour]
❑ Annan sjukdom [oth] ___[oth_other]
❑
❍ Ingen av dessa [996]
❍ Vet ej / vill ej svara [998]

❖ **B. FÖR VARJE SVAR SOM MARKERATS I DS30 VISA MOTSVARANDE FRÅGOR SOM FINNS NEDAN**

Om "epilepsi" är markerat i DS30, visa DS30.1 , DS31.2 och DS31.3

[dis_2_epil_diag]

DS30.1. Har en läkare ställt diagnosen epilepsi?

❍ Ja [1]
❍ Nej [2]
❍ Vet ej / vill ej svara [998]

[dis_2_epil_name]

DS31.2. Vilken är den medicinska beteckningen på epilepsin?

❍ Grand mal epilepsi [1]
❍ Petit mal [2]
❍ Lokaliserad (fokal) epilepsy [3]
❍ Infantil spasm [4]
❍ Annan [5]
❍ Vet ej / vill ej svara [998]

[dis_2_epil_age]

DS31.3. Hur gammal var du när du fick epilepsi första gången?

❍ (Ålder) [yrs]____________ [yrs_other] ❍ Vet ej / vill ej svara [998]

[dis_2_scia_diag]

DS31. Har en läkare ställt diagnosen ischias?

❍ Ja [1]
❍ Nej [2]
❍ Vet ej / vill ej svara [998]

Om ”artros” markerats, visa DS32.1 och DS32.2

[dis_2_artr_whc]

DS32.1. Vilka av följande stämmer in på dig för mer än 3 veckor under det senaste året? (Markera alla aktuella)

❑ Värk eller smärta i en led [ach]
❑ Svårighet att böja en led (varit stel i en led) [sti]
❑ Tagit smärtstillande medicin för värk eller smärta i en led [med]
❑ Ändrat dagliga aktiviterer eller fritidsaktiviteter p.g.a. värk eller smärta i en led [cha]
❍ Inget av dessa [996]
❍ Vet ej / vill ej svara [998]

[dis_2_artr_diag]

DS32.2. Har en läkare ställt diagnosen artros?

❍ Ja [1]
❍ Nej [2]
❍ Vet ej / vill ej svara [998]

[dis_2_rheu_diag]

DS33. Har en läkare ställt diagnosen ledgångsreumatim?

❍ Ja [1] ❍ Vet ej / vill ej svara [998]
❍ Nej [2]

[dis_2_fibr_diag]

DS34. Har en läkare ställt diagnosen fibromyalgi?

❍ Ja [1] ❍ Vet ej / vill ej svara [998]
❍ Nej [2]

[dis_2_migr_diag]

DS35. Har en läkare ställt diagnosen migrän?

❍ Ja [1] ❍ Vet ej / vill ej svara [998]
❍ Nej [2]

[dis_2_hort_diag]

DS36. Har en läkare ställt diagnosen Hortons sjukdom?

❍ Ja [1] ❍ Vet ej / vill ej svara [998]
❍ Nej [2]

[dis_2_burn_diag]

DS37. Har en läkare ställt diagnosen utbrändhet?

❍ Ja [1] ❍ Vet ej / vill ej svara [998]
❍ Nej [2]

[dis_2_depr_diag]

DS38. Har en läkare ställt diagnosen depression?

❍ Ja [1] ❍ Vet ej / vill ej svara [998]
❍ Nej [2]

[dis_2_gad_diag]

DS39. Har en läkare ställt diagnosen ångestsyndrom?

❍ Ja [1]   ❍ Vet ej / vill ej svara [998]
❍ Nej [2]

[dis_2_pani_diag]

DS40. Har en läkare ställt diagnosen panikångest?

❍ Ja [1]   ❍ Vet ej / vill ej svara [998]
❍ Nej [2]

[dis_2_agor_diag]

DS41. Har en läkare ställt diagnosen agorafobi?

❍ Ja [1]   ❍ Vet ej / vill ej svara [998]
❍ Nej [2]

[dis_2_soci_diag]

DS42. Har en läkare ställt diagnosen social fobi?

❍ Ja [1]   ❍ Vet ej / vill ej svara [998]
❍ Nej [2]

[dis_2_ocd_diag]

DS43. Har en läkare ställt diagnosen tvångssyndrom?

❍ Ja [1]   ❍ Vet ej / vill ej svara [998]
❍ Nej [2]

[dis_2_ptsd_diag]

DS44. Har en läkare ställt diagnosen posttraumatiskt stressyndrom?

❍ Ja [1]   ❍ Vet ej / vill ej svara [998]
❍ Nej [2]

Om DS30 är ”svårighet att tala…”

[dis_2_dysph_diag]

DS45. Hur skulle du beskriva dina språkliga färdigheter att på ditt modersmål:

	Mycket svårt [1]	Ganska svårt [2]	Ungefär medel [3]	Ganska lätt [4]	Mycket lätt [5]	Vet ej / vill ej svara [998]
Upprepa ord eller fraser såsom andra uttalat dem [rep]	☐	☐	☐	☐	☐	☐
Hitta ord eller kunna namnge saker [nam]	☐	☐	☐	☐	☐	☐
Förstå vad andra sager [com]	☐	☐	☐	☐	☐	☐
Uttrycka dig med ett flytande tal [flu]	☐	☐	☐	☐	☐	☐
Uttrycka dig i skrift [wri]	☐	☐	☐	☐	☐	☐

[dis_2_dysph_slur]

DS46. Är ditt tal sluddrigt, otydligt eller svagt?

❍ Ja [1] ❍ Vet ej / vill ej svara [998]
❍ Nej [2]

[dis_2_dysph_theraphy]

DS47. Har du fått talterapi eller någon annan {barn: habilitering, vuxna: rehabilitering} för talsvårigheter?

❍ Ja [1] ❍ Vet ej / vill ej svara [998]
❍ Nej [2]

Om DS30 är ”läs- och skrivsvårigheter…”

[dis_2_dysl_diag]

DS48. Har en logoped ställt diagnosen dyslexi?

❍ Ja [1] ❍ Vet ej / vill ej svara [998]
❍ Nej [2]

❖ **B. VILLKORET AVSLUTAS**

Ätbeteende

Inledande frågor

[eat_length]

ED10. Hur lång är du?

❍ (cm) [cm]____________ [cm_other]   ❍ Vet ej / vill ej svara [998]

[eat_weight]

ED20. Hur mycket väger du nu när du har inomhuskläder på dig?

❍ (kg) [kg]____________ [kg_other]   ❍ Vet ej /vill ej svara [998]

Om ålder > 18, visa ED25

[eat_weight_least]

ED25. Bortsett från när du varit sjuk, vad är det minsta du har vägt sedan du var 18 år?

❍ (kg) [kg]____________ [kg_other]   ❍ Vet ej / vill ej svara [998]

[eat_anorexia]

ED30. Har du någonsin haft anorexi?

❍ Ja [1]   ❍ Nej [3]
❍ Osäker [2]   ❍ Vill ej svara [997]

[eat_bulimia]

ED40. Har du någonsin haft bulimi?

❍ Ja [1]   ❍ Nej [3]
❍ Osäker [2]   ❍ Vill ej svara [997]

Om ED30 är inte "ja" och ED40 är inte "ja", visa ED50

[eat_suspected]

ED50. Har någon någonsin misstänkt att du hade en ätstörning?

❍ Ja [1] ❍ Nej [3]
❍ Osäker [2] ❍ Vill ej svara [997]

[eat_binges]

ED60. Har du någonsin hetsätit, d.v.s. du åt vad de flesta skulle anse ovanligt mycket mat på kort tid och det kändes som att du inte kunde kontrollera ditt ätande?

❍ Ja [1] ❍ Vill ej svara [997]
❍ Osäker [2]
❍ Nej [3]

[eat_purging]

ED70. Vilken/vilka av följande metoder har du någonsin använt dig av för att kontrollera din kroppsform eller vikt?

	Aldrig [3]	Några gånger [2]	Oftare [1]	Vet ej / vill ej svara [998]
Fastat eller inte ätit (i 24 timmar eller mer) [fas]	☐	☐	☐	☐
Använt bantningspiller [die]	☐	☐	☐	☐
Tränat i mer än 2 timmar per dag [exe]	☐	☐	☐	☐
Jag har fått mig själv att kräkas [vom]	☐	☐	☐	☐
Använt laxermedel eller vätskedrivande medicin [lax]	☐	☐	☐	☐

- ❖ **C1 (ANOREXI). RÄKNA FRAM BMI FRÅN ED10 OCH ED25. OM BMI <= 18.5 ELLER (ED30 ÄR "JA" ELLER "OSÄKER") ELLER ED50 ÄR "JA" VISA BÅDE DEN GENERELLA OCH ANOREXI SEKTIONEN**
- ❖ **C2 (HETSÄTNNING). OM ED60 ÄR "JA" VISA BÅDE DEN GENERELLA OCH HETSÄTNINGSSSEKTIONEN**
- ❖ **C3 (SJÄLVRENING). OM SVARET PÅ ED70 ÄR "OFTARE" FÖR ÅTMINSTONE EN FRÅGA VISA DEN GENERELLA SEKTIONEN OCH DE MOTSVARANDE FRÅGORNA I SJÄLVRENINGSSEKTIONEN**

Vikt och kroppsform

Se villkoren C1, C2 och C3

[eat_weight_most]

ED90. Vad har du vägt som mest {för gravida kvinnor: bortäknat från när du var gravid}?

❍ (kg) [kg]____________ [kg_other]   ❍ Vet ej /vill ej svara [998]

[eat2_weight_most_age]

ED100. Hur gammal var du när du vägde som mest?

❍ 14 år eller yngre [1]   ❍ Vet ej / vill ej svara [998]
❍ 15 till 19 år [2]
❍ 20 till 24 år [3]
❍ 25 till 29 år [4]
❍ 30 till 34 år [5]
❍ 35 till 39 år [6]
❍ 40 år eller äldre [7]

[eat_esteembody_1]

ED110. Hur mycket beror din självkänsla på din kroppsform eller vikt?

	1	2	3	4	5	
Inte alls	☐	☐	☐	☐	☐	Helt och hållet

Anorexi

Se villkor C1

[eat_loweight]

ED120. Har du någonsin haft en längre tidsperiod då du vägde mycket mindre än vad andra tyckte att du borde väga?

❍ Ja [1] ❍ Vet ej / vill ej svara [998]
❍ Nej [2]

Om ED120 är "ja", visa ED130

[eat_loweight_eatdis]

ED130. Var detta på grund av en ätstörning?

❍ Ja [1] ❍ Vet ej / vill ej svara [998]
❍ Nej [2]

❖ **D. OM SVARET PÅ ED120 ÄR "JA" VISA FRÅGORNA NEDAN**

[eat2_loweight_age]

ED140. Hur gammal var du då?

❍ 14 år eller yngre [1] ❍ Vet ej / vill ej svara [998]
❍ 15 till 19 år [2]
❍ 20 till 24 år [3]
❍ 25 till 29 år [4]
❍ 30 till 34 år [5]
❍ 35 till 39 år [6]
❍ 40 år eller äldre [7]

[eat_loweight_min]

ED150. Vad vägde du som minst?

❍ (kg) [kg]____________ [kg_other] ❍ Vet ej / vill ej svara [998]

[eat_loweight_length]

ED160. Hur lång var du då?

❍ (cm) [cm]____________ [cm_other] ❍ Vet ej / vill ej svara [998]

[eat_loweight_fear]

ED170. Under den tid du låg på denna låga vikt, hur rädd var du att du skulle gå upp i vikt eller bli tjock?

❍ Inte alls [1] ❍ Väldigt mycket [3]
❍ Något [2] ❍ Vet ej / vill ej svara [998]

[eat_loweight_neg]

ED180. Tänkte du någonsin att din låga vikt kunde påverka din hälsa negativt?

❍ Inte alls [1] ❍ Väldigt mycket [3]
❍ Något [2] ❍ Vet ej / vill ej svara [998]

[eat_loweight_feel]

ED190. När du hade så låg vikt, kände du dig tjock?

❍ Inte alls [1] ❍ Väldigt mycket [3]
❍ Något [2] ❍ Vet ej / vill ej svara [998]

Om kön "kvinna", visa ED200 till ED230

[eat_periods_started]

ED200. Hade din mens redan börjat före denna tidsperiod?

❍ Ja [1] ❍ Vet ej / vill ej svara [998]
❍ Nej [2]

Om svaret på ED200 är “ja”, visa ED210

[eat_periods_stop]

ED210. Upphörde din mens under den tid när du vägde så lite?

❍ Ja [1]   ❍ Vet ej / vill ej svara [998]
❍ Nej [2]

Om svaret på ED130 är “ja”, visa ED140 och ED150

[eat_periods_dur]

ED220. Hur länge upphörde din mens?

❍ (Antal månader) [mnt]____________ [mnt_other]   ❍ Vet ej / vill ej svara [998]
❍ (Antal år) [yrs]____________ [yrs_other]

[eat2_periods_age]

ED230. Hur gammal var du när din mens upphörde?

❍ 14 år eller yngre [1]   ❍ Vet ej / vill ej svara [998]
❍ 15 till 19 år [2]
❍ 20 till 24 år [3]
❍ 25 till 29 år [4]
❍ 30 till 34 år [5]
❍ 35 till 39 år [6]
❍ 40 år eller äldre [7]

❖ **D. VILLKORET AVSLUTAS**

Hetsätning

Se villkor C2

Tidigare nämnde du att du hetsåt, d.v.s. du åt vad de flesta skulle anse som ovanligt mycket mat på kort tid.

[eat_binges_nocntrl]

ED240. När du hetsåt, kände du att du inte kunde kontrollera ditt ätande?

❍ Ja, definitivt [1] ❍ Vet ej /vill ej svara [998]
❍ Ja, något [2]
❍ Nej [3]

[eat_binge]

ED245. När du hetsåt som mest, hur många gånger brukade du hetsäta under en månad?

❍ (Gånger per månad) [tms]____________ [tms_other] ❍ Vet ej /vill ej svara [998]

[eat_binges_period]

ED250. Hur länge varade dina perioder då du hetsåt?

❍ Mindre än en månad [1] ❍ 6 till 11 månader [4]
❍ 1 till 2 månader [2] ❍ 1 år eller längre [5]
❍ 3 till 5 månader [3] ❍ Vet ej /vill ej svara [998]

[eat2_binges_first_age]

ED255. Hur gammal var du när du hade din första episod av hetsätning?

❍ 14 år eller yngre [1] ❍ Vet ej / vill ej svara [998]
❍ 15 till 19 år [2]
❍ 20 till 24 år [3]
❍ 25 till 29 år [4]
❍ 30 till 34 år [5]
❍ 35 till 39 år [6]
❍ 40 år eller äldre [7]

[eat2_binges_last_age]

ED260. Hur gammal var du när du hade din sista episod av hetsätning?

❍ Jag hetsäter fortfarande [now] ❍ Vet ej / vill ej svara [998]

❍ 14 år eller yngre [1]
❍ 15 till 19 år [2]
❍ 20 till 24 år [3]
❍ 25 till 29 år [4]
❍ 30 till 34 år [5]
❍ 35 till 39 år [6]
❍ 40 år eller äldre [7]

[eat_binges1]

ED280. När du hetsåt:

	Ja [1]	Nej [2]	Vet ej / vill ej svara [998]
Åt mycket fortare än vanligt? [rap]	☐	☐	☐
Åt tills du blev proppmätt? [unc]	☐	☐	☐
Åt mycket mat fastän du inte kände dig fysiskt hungrig? [lar]	☐	☐	☐
Åt ensam därför att du var generad över hur mycket du åt? [alo]	☐	☐	☐
Kände dig äcklad över dig själv, deprimerad, eller mycket skyldig efter hetsätningen? [dis]	☐	☐	☐

[eat_binges_upset]

ED285. Hur orolig eller ledsen kände du dig över din hetsätning?

❍ Inte alls [3] ❍ Väldigt mycket [1]
❍ Något [2] ❍ Vet ej / vill ej svara [998]

Om villkoret C1 gäller, visa ED287

[eat_binges_anorex]

ED287. Inträffade dessa episoder av hetsätning under den tidsperiod då du vägde mycket mindre än vad andra tyckte att du borde väga?

❍ Ja [1] ❍ Vet ej / vill ej svara [998]
❍ Nej [2]

Självrening

Se villkor C3

Om “fastat eller inte ätit…”, visa ED290, ED291 och ED292

[eat2_fast_age]

ED290. Hur gammal var du när du fastade första gången?

❍ 14 år eller yngre [1] ❍ Vet ej / vill ej svara [998]
❍ 15 till 19 år [2]
❍ 20 till 24 år [3]
❍ 25 till 29 år [4]
❍ 30 till 34 år [5]
❍ 35 till 39 år [6]
❍ 40 år eller äldre [7]

[eat_fast_per]

ED291. Under den tiden då du fastade, hur långa var de perioder då du fastade?

❍ Kortare än 1 månad [1] ❍ 6 till 11 månader [3]
❍ 1 till 5 månader [2] ❍ Längre än 1 år [4]
❍ Vet ej / vill ej svara [998]

[eat_fast_frq]

ED292. Hur ofta brukade du fasta under dessa perioder?

❍ Mindre än en gång i veckan [1] ❍ Varje dag / nästan varje dag [3]
❍ En gång i veckan [2] ❍ Vet ej / vill ej svara [998]

Om “använde bantningspiller…”, visa ED293, ED294 och ED295

[eat2_dietpill_age]

ED293. Hur gammal var du när du använde bantningspiller för första gången?

❍ 14 år eller yngre [1] ❍ Vet ej / vill ej svara [998]
❍ 15 till 19 år [2]
❍ 20 till 24 år [3]
❍ 25 till 29 år [4]
❍ 30 till 34 år [5]
❍ 35 till 39 år [6]

❍ 40 år eller äldre [7]

[eat_dietpill_per]

ED294. Under den tiden då du använde bantningspiller hur långa var de perioder då du tog bantningspiller?

❍ Kortare än 1 månad [1]
❍ 1 till 5 månader [2]
❍ 6 till 11 månader [3]
❍ 1 år eller längre [4]
❍ Vet ej / vill ej svara [998]

[eat_dietpill_frq]

ED295. Hur ofta brukade du använda bantningspiller under dessa perioder?

❍ Mindre än en gång i veckan [1]
❍ En gång i veckan [2]
❍ Åtminstone två gånger i veckan [3]
❍ Vet ej / vill ej svara [998]

Om "tränade mer än 2 timmar…", visa ED296, ED297 och ED298

[eat2_exermre_age]

ED296. Hur gammal var du första gången du tränade mer än 2 timmar per dag?

❍ 14 år eller yngre [1]
❍ 15 till 19 år [2]
❍ 20 till 24 år [3]
❍ 25 till 29 år [4]
❍ 30 till 34 år [5]
❍ 35 till 39 år [6]
❍ 40 år eller äldre [7]
❍ Vet ej / vill ej svara [998]

[eat_exermre_per]

ED297. Under den tiden då du tränade mer än 2 timmar per dag hur långa var perioderna?

❍ Kortare än 1 månad [1]
❍ 1 till 5 månader [2]
❍ 6 till 11 månader [3]
❍ 1 år eller längre [4]
❍ Vet ej / vill ej svara [998]

Om "fick dig själv att kräkas", visa ED299, ED300 och ED301

[eat2_vomit_age]

ED299. Hur gammal var du när du fick dig själv att kräkas första gången?

❍ 14 år eller yngre [1] ❍ Vet ej / vill ej svara [998]
❍ 15 till 19 år [2]
❍ 20 till 24 år [3]
❍ 25 till 29 år [4]
❍ 30 till 34 år [5]
❍ 35 till 39 år [6]
❍ 40 år eller äldre [7]

[eat_vomit_per]

ED300. När du hade perioder då du fick dig själv att kräkas hur länge varade de?

❍ Kortare än 1 månad [1] ❍ 6 till 11 månader [3]
❍ 1 till 5 månader [2] ❍ 1 år eller längre [4]
❍ Vet ej / vill ej svara [998]

[eat_vomit_frq]

ED301. Hur ofta brukade du få dig själv att kräkas under dessa perioder?

❍ Mindre än en gång i veckan [1] ❍ Åtminstone två gånger i veckan [3]
❍ En gång i veckan [2] ❍ Vet ej / vill ej svara [998]

Om "använde laxermedel eller vätskedrivande medicin…", visa ED302

[eat_laxordiur]

ED302. Tidigare svarade du att du använde laxermedel eller vätskedrivande medicin för att gå ner i vikt. Vad använde du? (Markera alla aktuella)

❑ Laxermedel [laxa] ❑ Vätskedrivande medicin [dur]
❍ Vet ej / vill ej svara [998]

Om svaret "laxermedel" markerats i ED302, visa ED303, ED304 och ED305

[eat2_laxa_age]

ED303. Hur gammal var du när du använde laxermedel för första gången?

❍ 14 år eller yngre [1] ❍ Vet ej / vill ej svara [998]
❍ 15 till 19 år [2]
❍ 20 till 24 år [3]
❍ 25 till 29 år [4]
❍ 30 till 34 år [5]
❍ 35 till 39 år [6]
❍ 40 år eller äldre [7]

[eat_laxa_per]

ED304. När du hade perioder då du använde laxermedel, hur länge varade de?

❍ Kortare än 1 månad [1] ❍ 6 till 11 månader [3]
❍ 1 till 5 månader [2] ❍ 1 år eller längre [4]
❍ Vet ej / vill ej svara [998]

[eat_laxa_frq]

ED305. Hur ofta brukade du använda laxermedel under dessa perioder?

❍ Mindre än en gång i veckan [1] ❍ Åtminstone två gånger i veckan [3]
❍ En gång i veckan [2] ❍ Vet ej / vill ej svara [998]

Om svaret "vätskedrivande medicin" är markerat i ED302, visa ED306, ED307 och ED308

[eat2_diur_age]

ED306. Hur gammal var du när du använde vätskedrivande medicin för första gången?

❍ 14 år eller yngre [1] ❍ Vet ej / vill ej svara [998]
❍ 15 till 19 år [2]
❍ 20 till 24 år [3]
❍ 25 till 29 år [4]
❍ 30 till 34 år [5]
❍ 35 till 39 år [6]
❍ 40 år eller äldre [7]

[eat_diur_per]

ED307. När du hade perioder då du använde vätskedrivande medicin hur länge varade de?

❍ Kortare än 1 månad [1]
❍ 1 till 5 månader [2]
❍ 6 till 11 månader [3]
❍ 1 år eller längre [4]
❍ Vet ej / vill ej svara [998]

[eat_diur_frq]

ED308. Hur ofta brukade du använda vätskedrivande medicin under dessa perioder?

❍ Mindre än en gång i veckan [1]
❍ En gång i veckan [2]
❍ Åtminstone två gånger i veckan [3]
❍ Vet ej / vill ej svara [998]

Om villkoret C2 gäller, visa ED310

[eat_binge_purging]

ED310. Vilken/vilka av följande använde du dig av under din hetsätningsperiod? (Markera alla aktuella)

❑ Jag fick mig själv att kräkas [vom]
❑ Laxermedel [lax]
❑ Vätskedrivande medicin [diu]
❑ Bantningspiller [wei]
❑ Tränade i mer än 2 timmar per dag [exe]
❑ Fastade (åt ej alls) [fas]
❍ Inget av dessa [996]
❍ Vet ej / vill ej svara [998]

❖ **C1, C2 OCH C3. VILLKOREN AVSLUTAS**

Reflux, IBS

- **F. OM SVARET ÄR "HALSBRÄNNA ELLER SURA UPPSTÖTNINGAR" I DS10, VISA NEDANSTÅENDE FRÅGOR**

[ref_lastyear]

RE10. Har du haft halsbränna eller sura uppstötningar under det senaste året?

❍ Ja [1] ❍ Vet ej / vill ej svara [998]
❍ Nej [2]

Om RE10 är "nej", visa RE12

[ref_stopped]

RE12. Vid vilken ålder hade du detta / dessa problem senast?

❍ (Ålder) [yrs]____________ [yrs_other] ❍ Vet ej / vill ej svara [998]

Om RE12 är "ja", visa RE15 till RE50

[ref_behstern]

RE15. Vilket/vilka symtom besväras du av?

❑ Halsbränna [heart] ❑ Sura uppstötningar [reflux]
❍ Vet ej / vill ej svara [998]

[ref_behstern_age]

RE20. Hur gammal var du första gången du besvärades av detta / dessa problem?

❍ (Ålder) [yrs]____________ [yrs_other] ❍ Vet ej / vill ej svara [998]

[ref_behstern_frq]

RE30. Hur ofta har du smärta eller besvär på grund av {halsbränna, sura uppstötningar}?

❍ Mindre än en gång i månaden [4]
❍ Varje månad [3]
❍ Varje vecka [3]
❍ Varje dag [2]
❍ Vet ej / vill ej svara [998]

[ref_behstern_wakenight]

RE40. Brukar du vakna på natten på grund av {halsbränna, sura uppstötningar}?

❍ Ja [1]
❍ Nej [2]
❍ Vet ej / vill ej svara [998]

[ref_12mnt_med]

RE50. Har du under det senaste året tagit någon medicin på grund av {din halsbränna, dina sura uppstötningar}?

❍ Ja, dagligen [1]
❍ Ja, vid behov [2]
❍ Nej [3]
❍ Vet ej / vill ej svara [998]

Om RE50 är "ja...", visa RE60

[ref_12mnt_med_whc]

RE60. Vilken/vilka av följande mediciner använder du? (Markera alla aktuella)

❑ Novalucol, Novaluzid, Link, Rennie [nov]
❑ Andapsin, Sukralfat, Gaviscon [and]
❑ Artonil, Ranitidin, Inside, Zantac, Pepcid, Pepcidin [art]
❑ Omeprazol, Losec MUPS, Nexium, Lansoprazol, Lanso, Pantoloc [ome]
❑ Annan [oth]
❍ Vet ej / vill ej svara [998]

[ibs_stomrecur]

RE70. Har du haft återkommande magbesvär under det senaste året? {förutom halsbränna eller sura uppstötningar}

❍ Ja [1]
❍ Nej [2]
❍ Vet ej / vill ej svara [998]

❖ **G. OM RE70 ÄR "JA" VISA NEDANSTÅENDE FRÅGOR**

[ibs_stomrecur_lasts]

RE80. Har besvären varat i åtminstone 3 dagar per månad? (ej nödvändigtvis 3 dagar i följd)

❍ Ja [1] ❍ Vet ej / vill ej svara [998]
❍ Nej [2]

Om "ja", visa RE90

[ibs_stomrecur_age]

RE90. För hur länge sedan började dina besvär?

❍ 1 till 5 år sedan [1]
❍ 6 till 10 år sedan [2]
❍ 11 till 20 år [3]
❍ Mer än 20 år sedan [4]
❍ Vet ej / vill ej svara [998]

[ibs_stomrecur_discomf]

RE100. När du har magbesvär, brukar du känna något av följande:

❑ Ökad mättnadskänsla efter maten [sat]
❑ Uppkörd/uppblåst mage [blo]
❑ Förstoppning [con]
❑ Lös eller vattnig avföring [loo]
❑ En känsla av att tarmtömingen inte är avslutad [inc]
❑ Att tarmtömningen lindrar besvären [all]
❑
❍ Inget av dessa [996]
❍ Vet ej / vill ej svara [998]

[ibs_stomrecur_where]

RE110. Var känner du smärtan?

❍ I övre delen av magen [upp]
❍ I nedre delen av magen [low]
❍ I både övre och nedre delen av magen [bot]
❍ Vet ej / vill ej svara [998]

[ibs_stomrecur_pain]

RE120. Vad händer med smärtan i olika situationer:

❑ Den lindras när jag äter [rel]
❑ Den sätter igång när jag äter [tir]
❑ Den sätter igång när jag äter mejeriprodukter (laktosintolerans) [lac]
❑ Den väcker mig på natten [wak]
❑
❍ Inget av dessa [996]
❍ Vet ej / vill ej svara [998]

❖ **F OCH G. VILLKORET AVSLUTAS**

Sömn

Sömnkvalitet och sovtid

[sle_dist]

SP10. Brukar din sömn störas när du sover, t.ex. av barn, oljud eller {en partner, någon} som snarkar?

❍ Ja [1] ❍ Vet ej / vill ej svara [998]
❍ Nej [2]

[sle_general_gen]

SP20. Hur sover du vanligen?

	1	2	3	4	5	
Mycket dåligt	☐	☐	☐	☐	☐	Mycket bra

[sle_need]

SP30. Hur många timmars sömn behöver du per dygn?

❍ 5 timmar eller mindre [1] ❍ 8 timmar [4]
❍ 6 timmar [2] ❍ 9 timmar eller mer [5]
❍ 7 timmar [3] ❍ Vet ej / vill ej svara [998]

[sle_morneven_type_sle]

SP40. Är du en ”morgonmänniska” eller en ”kvällsmänniska”?

	Utpräglad morgonmänniska [1]	[2]	Varken eller [3]	[4]	Utpräglad kvällsmänniska [5]	
	☐	☐	☐	☐	☐	

[sle_tosleep]

SP50. Hur lång tid tar det för dig att somna (efter att du släckt lampan)?

❍ Mindre än 5 minuter [1] ❍ 30 till 59 minuter [4]

❍ 5 till 14 minuter [2] ❍ 60 minuter eller mer [5]
❍ 15 till 29 minuter [3] ❍ Vet ej / vill ej svara [998]

[sle_work_rise]

SP60. Hur dags brukar du stiga upp på en arbetsdag?

Klockan	och (minut)
❍ 4 eller tidigare [1]	❍ 00 [1]
❍ 5 [2]	❍ 15 [2]
❍ 6 [3]	❍ 30 [3]
❍ 7 [4]	❍ 45 [4]
❍ 8 [5]	
❍ 9 [6]	
❍ 10 eller senare [7]	

[sle_work_bed]

SP70. Hur dags brukar du gå och lägga dig (släcka lyset) kvällen före en arbetsdag?

Klockan	och (minut)
❍ 20 eller tidigare [1]	❍ 00 [1]
❍ 21 [2]	❍ 15 [2]
❍ 22 [3]	❍ 30 [3]
❍ 23 [4]	❍ 45 [4]
❍ 24 [5]	
❍ 1 [6]	
❍ 2 eller senare [7]	

[sle_nowork_rise]

SP80. Hur dags brukar du stiga upp på “lediga dagar”?

Klockan och (minut)

❍ 6 eller tidigare [1] ❍ 00 [1]

❍ 7 [2] ❍ 15 [2]

❍ 8 [3] ❍ 30 [3]

❍ 9 [4] ❍ 45 [4]

❍ 10 [5]

❍ 11 [6]

❍ 12 eller senare [7]

[sle_nowork_bed]

SP90. Hur dags brukar du gå och lägga dig (släcka lyset) kvällen före "lediga dagar"?

Klockan och (minut)

❍ 21 eller tidigare [1] ❍ 00 [1]

❍ 22 [2] ❍ 15 [2]

❍ 23 [3] ❍ 30 [3]

❍ 24 [4] ❍ 45 [4]

❍ 1 [5]

❍ 2 [6]

❍ 3 eller senare [7]

Sömnbesvär, snarkning, sömnapné

[sle_sleep_qual]

SP100.

	Aldrig eller sällan [4]	1 till 3 gånger i månaden [3]	1 till 3 gånger i veckan [2]	4 gånger eller mer i veckan [1]	Vet ej / vill ej svara [998]
Har du svårt att somna? [hard]	☐	☐	☐	☐	☐
Är din sömn orolig eller störd? [worr]	☐	☐	☐	☐	☐
Vaknar du flera gånger och har svårighet att somna om? [seve]	☐	☐	☐	☐	☐
Vaknar du för tidigt? [earl]	☐	☐	☐	☐	☐
Känner du dig inte utsövd när du vaknar? [notr]	☐	☐	☐	☐	☐
Hur ofta får du för lite sömn? [shor]	☐	☐	☐	☐	☐
Känner du dig rastlös i benen när du lägger dig eller när du sover? [legs]	☐	☐	☐	☐	☐
Snarkar du högt (enligt dig själv eller andra)? [snor]	☐	☐	☐	☐	☐
Har du andningsuppehåll eller svårt att andas under sömnen (enligt dig själv eller andra)? [brea]	☐	☐	☐	☐	☐

Om "har du andningsuppehåll..." i SP100 är besvarad "1 till 3 gånger..." eller oftare, visa SP101

[sle_sleep_qua_breat_diag]

SP101. Har en läkare någon gång ställt diagnosen sömnapné?

❍ Ja [1]   ❍ Vet ej / vill ej svara [998]

❍ Nej [2]

Sömnhjälpmedel

[sle_means]

SP103. Använder du någon medicin eller något hjälpmedel för att sova bättre?

❍ Ja, regelbundet [1]
❍ Ja, i perioder [2]
❍ Nej [3]
❍ Vet ej / vill ej svara [998]

Om SP103 är "ja...", visa SP106

[sle_means_type]

SP106. Vad gör du för att sova bättre? (Markera alla aktuella)

❑ Använder medicin eller medel (t.ex. sömnmedicin eller lugnande) [med]
❑ Använder hjälpmedel (t.ex. för snarkning) [mea]
❑ Följer råd eller tips [adv]
❑ Annat (vänligen ange) [oth]_____ [oth_other]
❑ ❍ Vet ej / vill ej svara [998]

Om SP106 inte är "vet ej...", visa SP109

[sle_means_frq]

SP109. Hur ofta använder du detta / dessa för att sova bättrre?

❍ 4 eller fler gånger i veckan [1]
❍ 1 till 3 gånger i veckan [2]
❍ 1 till 3 gånger i månaden [3]
❍ Mindre ofta [4]
❍ Vet ej / vill ej svara [998]

Sömnstörningar

[sle_probawake]

SP110. Har du svårt att hålla dig vaken under dagen?

❍ Sällan eller aldrig [3] ❍ Vet ej / vill ej svara [998]
❍ Ibland [2]
❍ Ofta [1]

[sle_afternoontired]

SP113. Är du trött på eftermiddagen?

❍ Sällan eller aldrig [3] ❍ Vet ej / vill ej svara [998]
❍ Ibland [2]
❍ Ofta [1]

[sle_winddown]

SP115. Har du svårt att varva ner på kvällen efter en stressig dag?

❍ Sällan eller aldrig [3] ❍ Vet ej / vill ej svara [998]
❍ Ibland [2]
❍ Ofta [1]

[sle_affectshrtsleep_day]

SP120. Hur påverkas du nästa dag om du har haft en kort eller störd sömn?

	1	2	3	4	5	
Jag kan lätt göra det jag brukar	☐	☐	☐	☐	☐	Nästa dag är svår att klara av

[sle_distlife_pro]

SP130. I vilken grad är sömnstörningar ett problem i ditt liv?

	1	2	3	4	5	
Inget problem alls	☐	☐	☐	☐	☐	Ett stort problem

Huvudvärk

Daglig och återkommande huvudvärk

[hea_recurrent]

HA10. Har du någonsin haft perioder med återkommande huvudvärk, ofta med intervaller utan huvudvärk emellan?

❍ Ja [1] ❍ Vet ej / vill ej svara [998]
❍ Nej [2]

[hea_daily]

HA15. Har du någonsin haft daglig eller nästan daglig huvudvärk i åtminstone ett par månader som inte orsakades av en vanlig förkylning, feber, baksmälla eller någon annan medicinsk orsak så vitt du vet?

❍ Ja [1] ❍ Vet ej / vill ej svara [998]
❍ Nej [2]

- **H. LÄMNA MODULEN OM HA10 ELLER HA15 ÄR "NEJ"**

[hea_monthly]

HA20. Har du någonsin haft återkommande huvudvärk en gång i månaden eller oftare?

❍ Ja [1] ❍ Vet ej / vill ej svara [998]
❍ Nej [2]

Huvudvärkssymtom

[hea_pain_gen]

HA25. Hur skulle du beskriva din vanliga huvudvärk?

	1	2	3	4	5	
Mild	☐	☐	☐	☐	☐	Svår

[hea_grade]

HA30. Upplever du något av följande när du har huvudvärk?

	Ja [1]	Nej [2]	Vet ej / vill ej svara [998]
Smärtan är antingen på höger eller vänster sida av huvudet [uni]	☐	☐	☐
Smärtan är på både höger och vänster sida av huvudet [both]	☐	☐	☐
Smärtan pulserar eller dunkar [puls]	☐	☐	☐
Smärtan känns som ett tryck på eller runt huvudet [pres]	☐	☐	☐
Illamående eller kräkning [naus]	☐	☐	☐
Ökad känslighet för ljud [sound]	☐	☐	☐
Ökad känslighet för ljus [light]	☐	☐	☐
Fysisk aktivitet förvärrar huvudvärken [act]	☐	☐	☐

- **I. OM “MIGRÄN” I DS10 ÄR “JA”; ELLER ÅTMINSTONE TVÅ AV ALTERNATIVEN “SMÄRTA PÅ ENDERA SIDAN”, “PULSERAR”, “FÖRVÄRRAS AV FYSISK AKTIVITET” I HA30 ÄR “JA” OCH HA20 ÄR VÄRRE ÄN “MILD” OCH [“STÖRRE KÄNSLIGHET FÖR LJUD OCH LJUS” I DS10 ÄR “JA” ELLER “ILLAMÅENDE ELLER KRÄKNING” I DS10 ÄR “JA”]; VISA NEDANSTÅENDE FRÅGOR**

[hea_vision]

HA40. Brukar du ha kortvariga synstörningar precis innan huvudvärken börjar? (Synstörningen kan bestå av ljusa prickar, sick-sack mönster eller problem med att läsa.)

❍ Ja [1] ❍ Vet ej / vill ej svara [998]
❍ Nej [2]

[hea_feelcoming]

HA50. Kan du känna när huvudvärken är på väg?

❍ Ja [1] ❍ Vet ej / vill ej svara [998]
❍ Nej [2]

❖ **I. VILLKORET AVSLUTAS**

Livskvalitet, diagnos, medicinering

[hea_dailyact]

HA60. Påverkar huvudvärken dina normala dagliga aktiviteter?

❍ Ja [1] ❍ Vet ej / vill ej svara [998]
❍ Nej [2]

[hea_doc]

HA70. Har du konsulterat läkare för din huvudvärk?

❍ Ja [1] ❍ Vet ej / vill ej svara [998]
❍ Nej [2]

Om HA70 är "ja", visa HA75

[hea_doc_age]

HA75. Hur gammal var du första gången när du pratade med din läkare om huvudvärken?

❍ (Ålder) [yrs]____________ [yrs_other] ❍ Vet ej / vill ej svara [998]

[hea_allev]

HA80. Lindrar vanliga värktabletter din huvudvärk?

❍ Ja [1] ❍ Vet ej / vill ej svara [998]
❍ Nej [2]

Hörsel och tal

Självestimering av hörsel

[her_hear]

HE10. Hur är din hörsel?

❍ Bra [1] ❍ Mycket nedsatt [3]
❍ Något nedsatt [2] ❍ Vet ej / vill ej svara [998]

Hörlursanvändning

[her_headphone]

HE20. Använder du ofta hörlurar (t.ex. när du lyssnar på mp3-spelare eller radio eller när du spelar dataspel)?

❍ Ja [1] ❍ Vet ej / vill ej svara [998]
❍ Nej [2]

- **J. OM HE20 ÄR "JA" VISA FRÅGORNA NEDAN**

[her_headphone_vol]

HE30. Hur hög volym har du i hörlurarna?

❍ Låg [1] ❍ Hög [3]
❍ Medel [2] ❍ Vet ej / vill ej svara [998]

[her_headphone_frq]

HE40. Hur ofta använder du hörlurar?

❍ Dagligen [1] ❍ Varje månad [3]
❍ Varje vecka [2] ❍ Vet ej / vill ej svara [998]

[her_headphone_yrs]

HE50. Hur många år har du använt hörlurar?

❍ Mindre än 1 år [1]
❍ 1 till 5 år [2]
❍ 6 till 10 år [3]
❍ 11 till 15 år [4]
. 16 till 20 år [5]
. Mer än 20 år [6]
❍ Vet ej / vill ej svara [998]

❖ **J. VILLKORET AVSLUTAT**

Tinnitus

[her_tinnitus]

HE60. Ringer det konstant i öronen eller har du något annat besvärande ljud i öronen (tinnitus)?

Ja
❍ Ibland, men ljudet stör mig inte [1]
❍ Hela tiden, ljudet är mycket störande [2]
❍
❍ Nej [3]
❍ Vet ej / vill ej svara [998]

Yrsel

❖ **K. OM ÅLDER >= 15, VISA NEDAN FRÅGOR**

[her_dizz]

HE70. Har du haft yrselattacker under det senaste året?

❍ Ja [1]
❍ Vet ej / vill ej svara [998]
❍ Nej [2]

[her_disease]

HE80. Har du haft återkommande öroninfektioner i ditt liv?

❍ Ja [1]
❍ Vet ej / vill ej svara [998]
❍ Nej [2]

Om HE70 är "ja", visa HE82, HE84 och HE86

[her_dizz_bed]

HE82. Upplever du stark yrsel när du går till sängs?

❍ Ja [1] ❍ Vet ej / vill ej svara [998]
❍ Nej [2]

[her_dizz_tinn]

HE84. Har du någonsin upplevt hörselnedsättning och tinnitus i samband med en yrselattack?

❍ Ja [1] ❍ Vet ej / vill ej svara [998]
❍ Nej [2]

[her_dizz_drop]

HE86. Har du någonsin fallit ihop när du haft kraftig yrsel?

❍ Ja [1] ❍ Vet ej / vill ej svara [998]
❍ Nej [2]

- **L. OM HE10 ÄR "NÅGOT NEDSATT" ELLER "MYCKET NEDSATT" VISA NEDANSTÅENDE FRÅGOR**
- **M. OM HE10 ÄR "NORMAL" VISA HE100 a OCH b**

Hörselnedsättning

Om HE10 är "mycket nedsatt", visa HE90

[her_hearaid]

HE90. Använder du hörapparat?

❍ Ja [1] ❍ Vet ej / vill ej svara [998]
❍ Nej [2]

[her_difficulties]

HE100.

	Nej, inte alls [3]	Ibland, lite svårt [2]	Ja, mycket svårt [1]	Vet ej / vill ej svara [998]
a) Har du svårt att höra när du talar med en person i ett tyst rum? [one]	☐	☐	☐	☐
b) Har du svårt att höra när du talar med flera personer samtidigt? [sev]	☐	☐	☐	☐
c) Har du svårt att höra när du talar med någon i stadstrafik? [tra]	☐	☐	☐	☐
d) Har du svårt att höra varifrån olika ljud kommer, t.ex. billjud i trafiken? [dir]	☐	☐	☐	☐
e) Har du problem med hörseln och därför undviker att träffa folk? [soc]	☐	☐	☐	☐

❖ **K, L OCH M. VILLKOREN AVSLUTAS**

Tal

[her_speakprob]

HE101. Har du någonsin haft en period i ditt liv med följande talproblem? (Markera alla aktuella)

Ja

❑ Stamning [stu] ❍ Vet ej / vill

❑ Talat ovanligt snabbt [qui] ej svara [998]
❑ Hes eller svag röst utan att varit förkyld eller haft luftvägsinfektion [hoa]
❍ Nej [no]

Om HE101 är "stamning", visa HE105 och HE106

[her_speakprob_stut_12mnt]

HE105. Har du haft problem med stamning under de senaste 12 månaderna?

❍ Ja [1] ❍ Vet ej / vill ej svara [998]
❍ Nej [2]

[her_speakprob_stut_frq]

HE106. Hur ofta {brukade du stamma tidigare, brukar du stamma}?

❍ I ditt dagliga tal [1] ❍ Vet ej / vill ej svara [998]
❍ Vid speciella tillfällen [2]

Om HE100 är "talat ovanligt snabbt", visa HE107 och HE108

[her_speakprobquic_12mnt]

HE107. Har du haft problem med ha talat ovanligt snabbt under de senaste 12 månaderna?

❍ Ja [1] ❍ Vet ej / vill ej svara [998]
❍ Nej [2]

[her_speakprob_quic_frq]

HE108. Hur ofta {brukade du tala ovanligt snabbt tidigare, brukar du tala ovanligt snabbt}?

❍ I ditt dagliga tal [1] ❍ Vet ej / vill ej svara [998]
❍ Vid speciella tillfällen [2]

Om HE100 är "hes eller svag röst...", visa HE121 och HE122

[her_speakprob_hoar_12mnt]

HE121. Har du haft problem, under de senate 12 månaderna, med hes eller svag röst utan att varit förkyld eller haft någon luftvägsinfektion?

❍ Ja [1] ❍ Vet ej / vill ej svara [998]
❍ Nej [2]

[her_speakprob_hoar_frq]

HE122. Hur ofta {hade du tidigare, har du} problem med hes eller svag röst?

❍ Någon eller några gånger per år [1] ❍ Vet ej / vill ej svara [998]
❍ Månadsvis [2]
❍ Veckovis [3]
❍ Dagligen eller nästan dagligen [4]

Skador

Innehåll

Innehåll 1

Senaste års skadetillfällen 2

Skadeorsak 3

Skadade kroppsdelar 4

Vård sökt för skada 7

Långtidseffekter av tidigare skada 8

Riskbeteende.. 9

Senaste års skadetillfällen

[inj_1]

IN1. Har {du, ditt barn} under de senaste 12 månaderna råkat ut för en skada som hindrat {dig, honom/henne} från att utföra {dina, sina} vanliga aktiviteter under en dag eller mer?

Exempel på skadetillfällen:

- En olyckshändelse (t.ex. ett fall, blivit träffad av något föremål, slagit sig mot något, en trafikolycka)

- En skadlig händelse (t.ex. ett bett, blivit attackerad av annan person, ett kvävningstillbud, ett drunkningstillbud)

- Andra farliga omständigheter (t.ex. svalt giftigt ämne, andats in giftiga ångor, blivit utsatt för höga ljud, fått en elektrisk stöt)

❍ Nej [3]

❍ Ja, en gång [1]

❍ Ja, flera gånger [2]

❍ Vet ej / vill ej svara [998]

Om IN1 är "ja, flera gånger", visa IN1.5

[inj_1_5]

IN1.5. Hur många gånger blev {du, ditt barn} skadad under de senaste 12 månaderna?

❍ Två gånger [2]

❍ Tre gånger [3]

❍ Fyra gånger [4]

❍ Fem gånger [5]

❍ Sex gånger [6]

❍ Sju gånger eller mer [7]

❍ Vet ej / vill ej svara [998]

❖ **B. FÖR VARJE SKADETILLFÄLLE I IN1 VISA NEDANSTÅENDE FRÅGOR**

Skadeorsak

[inj_1_6]

IN1.6. Varför inträffade skadan {den n:te gången}? (Beskriv med egna ord)

____

[inj_1_7]

IN1.7. Hur utlöstes skadan?

❍ Slumpmässigt olycksfall [1]

❍ Någon försökte avsiktligt skada mig [2]

❍ Försökte skada mig själv [3]

❍ På annat sätt [4]

❍ Vet ej / vill ej svara [998]

[inj_1_8]

IN1.8. Hur orsakades skadan?

❑ Fallskada [1]
❑ Våldshandling [2]
❑ Skärande våld [3]
❑ Förgiftning [4]
❑ Kvävning [5]
❑ Drunkningstillbud [6]
❑ Av en maskin [7]
❑ Bilolycka [8]
❑ Cykelolycka [9]
❑ Gångtrafikantolycka [10]
❑ Annan typ av
❑ Överansträngning [13]
❑ Hett föremål [14]
❑ Eld [15]
❑ Pistol / gevär [6]
❑ På annat sätt [17]
❍ Vet ej / vill ej svara

transportolycka [11]   [998]
❑ Miljöolycka [12]

Skadade kroppsdelar

[inj_9]

IN9. Vilken eller vilka delar av kroppen blev skadade {den n:te gången}? (Markera alla aktuella)

☐ Armar eller ben [ext]

☐ Huvud (t.ex. skalle, ögon eller näsa) [hea]

☐ Hals / nacke [nec]

☐ Rygg / ryggrad [bac]

☐ Bröstkorg [tho]

☐ Mage / buk [abd]

☐ Bäcken [pel]

❍ Vet ej / vill ej svara [998]

Om IN9 är “armar eller ben”, visa IN9.1 nedan

[inj_9_ext_kind]

IN9.1. Vilken slags arm- / benskada drabbades {du, ditt barn} av {den n:te gången}?

❑ Ytlig skada [sup]
❑ Sår (annat än brännskada) [wou]
❑ Ur led, vrickning, stukning [dis]
❑ Benbrott (fraktur) [fra]
❑ Kärl-/nervskada [blo]
❑ Brännskada [bur]
❑ Köldskada / förfrysning [fro]
❑ Annan skada [oth]
❍
❍ Vet ej / vill ej svara [998]

Om IN9 är “huvud”, visa IN9.1 till IN9.6 nedan

[inj_9_hea_type]

IN9.1. Vilken eller vilka delar av huvud blev skadade {den n:te gången}? (Markera alla aktuella)

☐ Skalle [scu] ☐ Mun eller tänder [tee]

☐ Hjärna [bra] ☐ Annan del av huvudet (vänligen ange) [oth] ___[oth_other]

☐ Ögon [eye]

☐ Öron [ear] ❍ Vet ej / vill ej svara [998]

☐ Näsa [nos]

För varje markerad skada i IN9.1, visa IN9.2

[inj_9_hea_kind]

IN9.2. Vilken slags {skada} drabbades {du, ditt barn} av {den n:te gången}?

❑ Ytlig skada [sup]
❑ Sår (annat än brännskada) [wou]
❑ Krosskada / fraktur [fra]
❑ Hjärnskada [bra]
❑ Nervskada (domningar, försvagning eller förlamning) [ner]
❑ Brännskada [bur]
❑ Köldskada / förfrysning [fro]
❑ Annan skada [oth]
❍
❍ Vet ej / vill ej svara [998]

[inj_9_hea1]

IN9.3. Kan huvudskadan som {du, ditt barn} drabbades av {den n:te gången} kopplas till:

	Ja [1]	Nej [2]	Vet ej / vill ej svara [998]
En period av desorientering, förvirring eller nedsatt medvetandegrad [conf]	☐	☐	☐
Medvetslöshet upp till 30 minuter [cons]	☐	☐	☐
Minnesrubbningar eller minnesförlust i samband med skadan [memo]	☐	☐	☐
Kramper eller krampattacker [seiz]	☐	☐	☐

	Ja [1]	Nej [2]	Vet ej / vill ej svara [998]
Lättretlighet, apati eller illamående [irri]			
Huvudvärk, yrsel, utmattningskänsla eller koncentrationssvårigheter kort efter skadan [head]			

Om IN9 är "hals/nacke", visa IN9.1 nedan

[inj_9_neck]
IN9.1. Vilken slags hals- / nackskada drabbades {du, ditt barn} av {den n:te gången}?

❑ Ytlig skada [sup]
❑ Sår (annat än brännskada) [wou]
❑ Sträckning, dislokation (ur led) [dis]
❑ Benbrott (fraktur) [fra]
❑ Nervskada (domningar, försvagning eller förlamning) [ner]
❑ Brännskada [bur]
❑ Köldskada / förfrysning [fro]
❑ Annan skada [oth]
❍
❍ Vet ej / vill ej svara [998]

Om IN9 är "rygg/ryggrad", visa IN9.1 nedan

[inj_9_back]
IN9.1. Vilken slags rygg- / ryggradsskada drabbades {du, ditt barn} av {den n:te gången}?

❑ Ytlig skada [sup]
❑ Sår (annat än brännskada) [wou]
❑ Sträckning, dislokation (ur led) [dis]
❑ Benbrott (fraktur) [fra]
❑ Nervskada (domningar, försvagning eller förlamning) [ner]
❑ Brännskada [bur]
❑ Köldskada / förfrysning [fro]
❑ Annan skada [oth]
❍
❍ Vet ej / vill ej svara [998]

Om IN9 är "bröstkorg", visa IN9.1 nedan

[inj_9_thorax]

IN9.1. Vilken slags bröstkorgsskada drabbades {du, ditt barn} av {den n:te gången}?

❑ Ytlig skada [sup]
❑ Sår (annat än brännskada) [wou]
❑ Sträckning, dislokation (ur led) [dis]
❑ Revbensfraktur (ej ryggradsskada) [fra]
❑ Skador på inre organ [int]
❑ Brännskada [bur]
❑ Köldskada / förfrysning [fro]
❑ Annan skada [oth]
❍
❍ Vet ej / vill ej svara [998]

Om IN9 är "buk", visa IN9.1 nedan

[inj_9_abdomen]

IN9.1. Vilken slags bukskada drabbades {du, ditt barn} av {den n:te gången}?

❑ Ytlig skada [sup]
❑ Sår (annat än brännskada) [wou]
❑ Skador på inre organ [int]
❑ Brännskada [bur]
❑ Köldskada / förfrysning [fro]
❑ Annan skada [oth]
❍
❍ Vet ej / vill ej svara [998]

Om IN9 är "bäcken", visa IN9.1 nedan

[inj_9_pelvis]

IN9.1. Vilken slags bäckenskada drabbades {du, ditt barn} av {den n:te gången}?

❑ Ytlig skada [sup]
❑ Sår (annat än brännskada) [wou]
❑ Skador på inre organ [int]
❑ Benbrott / fraktur [fra]
❑ Brännskada [bur]
❑ Köldskada / förfrysning [fro]
❑ Annan skada [oth]
❍
❍ Vet ej / vill ej svara [998]

Vård sökt för skada

[inj_10]

IN10. Sökte du dig till någon sjukvårdsinrättning {, för ditt barn} på grund av {skadan, någon skada}?

❍ Ja [1]   ❍ Vet ej / vill ej svara [998]

❍ Nej [2]

Om IN10 är "ja", visa IN11

[inj_11]

IN11. Behövde {du, ditt barn} vårdas minst en natt på sjukhus på grund av skadan?

❍ Ja [1]   ❍ Vet ej / vill ej svara [998]

❍ Nej [2]

❖ **B OCH C. VILLKORET AVSLUTAS**

Långtidseffekter av tidigare skada

Om ålder >= 11, visa IN12

[inj_12]

IN12. Har {du, ditt barn} på grund av någon skada i {ditt, sitt} liv:

	Nej [2]	Ja [1]	Vet ej / vill ej svara [998]
Svårt att se, även med glasögon? [see]	☐	☐	☐
Svårt att höra, även med hörapparat? [hea]	☐	☐	☐
Problem med minnet eller med koncentrationen? [rem]	☐	☐	☐
Svårt att sköta {dig, sig} själv, t.ex. att tvätta eller klä {dig, sig}? [car]	☐	☐	☐

	Nej [2]	Ja [1]	Vet ej / vill ej svara [998]
Svårt att kommunicera på {ditt, sitt} modersmål? [com]	☐	☐	☐

Om någon fråga i IN12 är "ja", visa IN17

[inj_17]

IN17. Hur länge har {du, ditt barn} haft funktionsnedsättning till följd av skadan?

❍ Mindre än 1månad [1] ❍ Mer än 6 månader [3]

❍ 1 till 6 månader [2] ❍ Vet ej / vill ej svara [998]

Riskbeteende

Om IN1 är "ja" ålder >= 11, visa IN12

[inj_18]

IN18. Anser du att {du, ditt barn} har en större risk än genomsnittet att bli skadad under de kommande 5 åren?

❍ Ja [1] ❍ Vet ej / vill ej svara [998]

❍ Nej [2]

Om IN18 är "ja", visa IN19

[inj_19]

IN19. Vad tror du är den huvudsakliga orsaken till den förhöjda risken?

❍ {Ditt, Ditt barns} beteendemönster eller {din,} personlighet [1]

❍ Vistelse i farlig miljö (trafik, maskiner, jordbruk etc.) [2]

❍ Högrisksport eller riskfylld fritidssysselsättning [3]

❍ Annan orsak [4]

❍ Vet ej / vill ej svara [998]

Astma och allergi

Innehåll

Innehåll 1
Allmänna frågor 3
Golv och säng 3
Ventilation 4
Mögel och användande av gasspis 4
Astma 6
Väsande eller pipande andning 6
Andnödsattack 7
Astma 7
Sjukhusvistelse 10
Medicinering 10
KOL (kronisk obstruktiv lungsjukdom) 12
Hosta och slem 12
Andnöd 13
Allergi och kronisk bihåleinflammation 15
Allergisk och icke-allergisk snuva 15
Födoämnesallergi 17
Andra allergier 19
Medicinering 21
Kronisk bihåleinflammation 21

Allmänna frågor

Golv och säng

[exp_vacuum_frq]

IA10. Hur ofta dammsugs golven i vardagsrum och sovrum?

❍ 2 gånger i veckan eller mer [1]
❍ 1 gång i veckan [2]
❍ 2 till 3 gånger i månaden [3]
❍ 1 gång i månaden [4]
❍ 2 eller 3 gånger i halvåret [5]
❍ Mindre ofta [6]
❍ Vet ej / vill ej svara [998]

[exp_floorwash_frq]

IA20. Hur ofta torkas golven i vardagsrum och sovrum?

❍ 2 gånger i veckan eller mer [1]
❍ 1 gång i veckan [2]
❍ 2 till 3 gånger i månaden [3]
❍ 1 gång i månaden [4]
❍ 2 eller 3 gånger i halvåret [5]
❍ Mindre ofta [6]
❍ Vet ej / vill ej svara [998]

[exp_mattress]

IA30. I hur många år har du använt din nuvarande säng?

❍ (År) [yrs]____________ [yrs_other] ❍ Vet ej / vill ej svara [998]

[exp_mattresscover]

IA40. Har du skyddsöverdrag eller madrasskydd på sängen?

❍ Ja [1]
❍ Nej [2]
❍ Vet ej / vill ej svara [998]

Om "ja", visa IA50

[exp_mattresscover_2ndmnt]

IA50. Brukar skyddsöverdraget eller madrasskyddet tvättas oftare än varannan månad?

❍ Ja [1] ❍ Vet ej / vill ej svara [998]
❍ Nej [2]

Ventilation

[exp_3glasswindow]

IA55. Är det 3-glasfönster i din bostad?

❍ Ja [1] ❍ Vet ej / vill ej svara [998]
❍ Nej [2]

[exp_windowopen]

IA57. Har du vanligtvis öppet fönster eller balkongdörren öppen?

Ja
❍ Varje dag eller natt [1]
❍ Varje vecka [2]
❍ På sommaren [3]
❍ Nej [4]
❍ Vet ej / vill ej svara [998]

[exp_cond_winter]

IA60. Finns det kondens på insidan av vardagsrums- eller sovrumsfönstren på vintern?

❍ Ja, det händer [1] ❍ Nej [2]
❍ Vet ej / vill ej svara [998]

Om "ja", visa IA70

[exp_cond_novmarch_frq]

IA70. Hur ofta förekommer kondens på vintern (november-mars)?

❍ Varje månad [3] ❍ Varje dag [1]
❍ Varje vecka [2] ❍ Vet ej / vill ej svara [998]

Mögel och användande av gasspis

[exp_moisestain]

IA80. Finns det eller har det funnits fläckar som orsakats av fukt i ditt hem?

❍ Ja [1] ❍ Vet ej / vill ej svara [998]
❍ Nej [2]

[exp_mold]

IA90. Finns det eller har det funnits lukt av mögel eller mjöldagg i ditt hem?

❍ Ja [1] ❍ Vet ej / vill ej svara [998]
❍ Nej [2]

[exp_gasstove]

IA100. Finns det en gasspis i ditt hem?

❍ Ja [1] ❍ Vet ej / vill ej svara [998]
❍ Nej [2]

Om "ja", visa IA110

[exp_gasstove_daily]

IA110. Används gasspisen dagligen?

❍ Ja [1] ❍ Vet ej / vill ej svara [998]
❍ Nej [2]

Astma

Väsande eller pipande andning

Om ålder < 15, visa IA130

[ast_wheez]

IA130. Har {ditt barn, du} någonsin haft pipande eller väsande andning?

❍ Ja [1] ❍ Vet ej / vill ej svara [998]
❍ Nej [2]

Om ålder >= 15 eller IA130 är "ja", visa IA140

[ast_12mnt_wheez]

IA140. Har {du, ditt barn} haft pip eller har det väst i bröstet vid något tillfälle under de senaste {6, 12} månaderna?

❍ Ja [1] ❍ Vet ej / vill ej svara [998]
❍ Nej [2]

Om ålder över 1 och IA130 eller IA140 är "ja", visa IA145 och IA146

[ast_whz_present]

IA145. Vid vilka tillfällen har {du, ditt barn} pipande eller väsande andning?

❑ Vid förkylningar [col]
❑ {ålder < 6: Under eller efter lek eller gråt, Under eller efter fysisk träning} [exe]
❑ Vid pollen- eller djurexponering [pol]
❑ Vid andra tillfällen [oth]
❍ Vet ej / vill ej svara [998]

[ast_whz_frq]

IA146. Hur många gånger har {ditt barn, du} haft pipande eller väsande andning under de senaste 12 månaderna?

❍ 1 till 3 gånger [1] ❍ Vet ej / Vill ej svara [998]
❍ 4 till 12 gånger [2]
❍ Mer än 12 gånger [3]

Andnödsattack

Om ålder >= 4, visa IA150 och IA160

[ast_12mnt_stren]

IA150. Har {du, ditt barn} någon gång under de senaste 12 månaderna haft någon andnödsattack som kommit efter ansträngning?

❍ Ja [1] ❍ Vet ej / vill ej svara [998]
❍ Nej [2]

[ast_12mnt_wake]

IA160. Har {du, ditt barn} vaknat av andnödsattack vid något tillfälle under de senaste 12 månaderna?

❍ Ja [1] ❍ Vet ej / vill ej svara [998]
❍ Nej [2]

Astma

Om ålder >= 1, visa IA165

[ast_asthma]

IA165. Har {du, ditt barn} astma?

❍ Ja [1] ❍ Vet ej / vill ej svara [998]
❍ Nej [2]

Om IA165 är ”ja”, visa IA167

[ast_asthma_diag]

IA167. Har en läkare ställt diagnosen astma?

❍ Ja [1]   ❍ Vet ej / vill ej svara [998]
❍ Nej [2]

Om IA167 är ”ja”, visa IA168

[ast_asthma_diag_age]

IA168. Vid vilken ålder fick {du, ditt barn} diagnosen astma?

❍ (År gammal) [yrs]_____ [yrs_other]   ❍ Vet ej / vill ej svara [998]

Om IA168 är ”vet ej / vill ej svara”, visa IA169

[ast_asthma_diag_age_estim]

IA169. Vid vilken ålder uppskattar du att {ditt barn, du} fick diagnosen astma?

❍ 0-6 år [1]
❍ 7-12 år [2]
❍ 13-18 år [3]
❍ Över 18 år [4]
❍ Vet ej / vill ej svara [998]

- **B. LÄMNA MODULEN OM ”ASTMA” EJ ÄR MARKERAD I C.DS10, C.DS15 ELLER DS10 ELLER IA165 ÄR ”NEJ”**

[ast_first_age]

IA170. Hur gammal var {ditt barn, du} när {han/hon hade sitt, du hade ditt} första astmaanfall?

❍ (Ålder) [yrs]____________ [yrs_other] ❍ Vet ej / vill ej svara [998]

Om IA170 är "vet ej / vill ej svara", visa IA171

[ast_first_age_estim]

IA171. Vid vilken ålder uppskattar du att {ditt barn hade sitt, du hade ditt} första astmaanfall?

❍ 0-6 år [1]
❍ 7-12 år [2]
❍ 13-18 år [3]
❍ Över 18 år [4]
❍ Vet ej / vill ej svara [998]

Om ålder mellan 2 och 15, visa IA175

[ast_12mnt_speachlim]

IA175. Under de senaste 12 månaderna, har den pipande eller väsande andningen begränsat {talet, ditt tal} till bara ett eller två ord i taget mellan andetagen?

❍ Ja [1] ❍ Vet ej / vill ej svara [998]
❍ Nej [2]

[ast_breathtimes]

IA180. Hur ofta har {du, ditt barn} haft andningssvårigheter på grund av astma under de senaste 12 månaderna?

❍ Hela tiden [1]
❍ Ungefär en gång per dag [2]
❍ Mer än en gång i veckan, men mindre än en gång per dag [3]
❍ Mindre än en gång i veckan[4]
❍ Inte alls [5]
❍ Vet ej / vill ej svara [998]

[ast_waketimes]

IA185. Hur många gånger har {du, ditt barn} blivit väckt av {din, sin} astma under de senaste 12 månaderna?

❍ Varje natt eller nästan varje natt [1]
❍ Mer än en gång i veckan, men inte de flesta nätter [2]
❍ Mer än två gånger per månad, men högst en gång i veckan [3]
❍ Mindre än två gånger per månad [4]
❍ Inte alls [5]
❍ Vet ej / vill ej svara [998]

[ast_interfered]

IA190. Hur mycket har {du, ditt barn} störts av {din, sin} astma i {dina, sina} dagliga aktiviteter under de senaste 12 månaderna?

❍ Inte alls [4]
❍ Lite [3]
❍ Något [2]
❍ Mycket [1]
❍ Vet ej / vill ej svara [998]

Sjukhusvistelse

[ast_emergency]

IA195. Har {du, ditt barn} varit på en akutmottagning på grund av astma under de senaste 12 månaderna?

❍ Ja [1]
❍ Nej [2]
❍ Vet ej / vill ej svara [998]

Om IA195 är "ja", visa IA197

[ast_hospital]

IA197. Har {du, ditt barn} blivit inlagd på sjukhus för astmabesvär under de senaste 12 månaderna?

❍ Ja [1]
❍ Nej [2]
❍ Vet ej / vill ej svara [998]

Medicinering

[ast_med]

IA200. Tar {du, ditt barn} för närvarande någon astmamedicin (inklusive inhalatorer, sprejer eller tabletter)?

❍ Ja [1] ❍ Vet ej / vill ej svara [998]
❍ Nej [2]

Om IA200 är "ja", visa IA202 och IA204

[ast_med1]

IA202. Har {du, ditt barn} tagit snabbverkande luftrörsvidgande medicin, till exempel Bricanyl, Ventoline, Buventol eller Airomir, på grund av astmabesvär mer än två gånger den senaste veckan?

❍ Ja [1] ❍ Vet ej / vill ej svara [998]
❍ Nej [2]

[ast_med2]

IA204. Har {du, ditt barn} under de senaste 12 månaderna använt följande mediciner?

	Ja, regelbundet [1]	Ja, i perioder [2]	Nej [3]	Vet ej / vill ej svara [998]
Kortison för inandning - Pulmicort, Flutide, Becotide, Asmanex, Beclomet, etc. [cor]	☐	☐	☐	☐
Långverkande luftrörsvidgande - Serevent, Oxis, etc. [lon]	☐	☐	☐	☐
En kombination av långverkande luftrörsvidgande och kortison - Seretide, Symbicort, etc. [com]	☐	☐	☐	☐
Singulair (tabletter) [sin]	☐	☐	☐	☐
Kortisontabletter – Prednisolon, Betapred [ste]				

KOL (kronisk obstruktiv lungsjukdom)

Hosta och slem

- **D. OM ÅLDER ÄR >= 35 ELLER SVARET PÅ DS10 (KOL) ÄR MARKERAD VISA NEDANSTÅENDE FRÅGOR**

[cop_cou_3mnt]

IA210. Brukar du hosta nästan varje dag under åtminstone tre månader per år?

❍ Ja [1] ❍ Vet ej / vill ej svara [998]
❍ Nej [2]

Om "ja", visa IA220

[cop_cou_3mnt_yr]

IA220. I hur många år har du haft denna hosta?

❍ Mindre än 2 år [1] ❍ Mer än 5 år [3]
❍ 2 till 5 år [2] ❍ Vet ej / vill ej svara [998]

[cop_phl_3mnt]

IA230. När du inte är förkyld, brukar du få upp slem från bröstet nästan varje dag under åminstone tre månader per år?

❍ Ja [1] ❍ Vet ej / vill ej svara [998]
❍ Nej [2]

Om "ja", visa IA240

[cop_phl_yr]

IA240. I hur många år har du haft dessa slemproblem?

❍ Mindre än 2 år [1] ❍ Mer än 5 år [3]
❍ 2 till 5 år [2] ❍ Vet ej / vill ej svara [998]

Andnöd

❖ **E. OM SVARET PÅ IA210 ELLER IA230 ÄR "JA" VISA NEDANSTÅENDE FRÅGOR**

[cop_hill]

IA250. Besväras du av andnöd när du går och har bråttom eller när du går uppför en mindre backe?

❍ Ja [1] ❍ Vet ej / vill ej svara [998]
❍ Nej [2]

Om "nej", visa IA260

[cop_walkslow]

IA260. Blir du andfådd när du går med andra personer i din egen ålder på plan mark?

❍ Ja [1] ❍ Vet ej / vill ej svara [998]
❍ Nej [2]

Om "nej", visa IA270

[cop_100m]

IA270. Måste du stanna för att andas när du går cirka 100 meter på plan mark?

❍ Ja [1] ❍ Vet ej / vill ej svara [998]
❍ Nej [2]

Om “nej”, visa IA280

[cop_dress]

IA280. Blir du andfådd av att klä av eller på dig?

❍ Ja [1] ❍ Vet ej / vill ej svara [998]
❍ Nej [2]

❖ **D OCH E. VILLKOREN AVSLUTAS**

Allergi och kronisk bihåleinflammation

Allergisk och icke-allergisk snuva

[all_nasal]

IA290. Har {ditt barn, du} hösnuva eller någon annan allergisk snuva?

❍ Ja [1] ❍ Vet ej / vill ej svara [998]
❍ Nej [2]

Om IA290 är "nej", visa IA291

[all_non]

IA291. Har {ditt barn, du} haft problem med nästäppa, snuva eller nysattacker under de senaste 12 månaderna utan att {han/hon, du} haft en förkylning eller allergi?

❍ Ja [1] ❍ Vet ej / vill ej svara [998]
❍ Nej [2]

Om IA291 är "ja", visa IA292

[all_non_diag]

IA292. Har en läkare ställt diagnosen icke-allergisk rinit?

❍ Ja [1] ❍ Vet ej / vill ej svara [998]
❍ Nej [2]

Om IA292 är "ja", visa IA292.1

[all_non_diag_age]

IA292.1. Vid vilken ålder fick {ditt barn, du} diagnosen icke-allergisk rinit?

❍ (År gammal) [yrs]_____ [yrs_other] ❍ Vet ej / vill ej svara [998]

❖ **F. OM IA290 ÄR "JA" VISA NEDANSTÅENDE FRÅGOR**

[all_nasal]

IA293. Har {ditt barn, du} haft problem med hösnuva eller allergisk snuva under de senaste 12 månaderna?

❍ Ja [1] ❍ Vet ej / vill ej svara [998]
❍ Nej [2]

Om IA293 är "ja", visa IA294

[all_nasal_4days]

IA294. Har {ditt barn, du} under de senaste 12 månaderna haft problem med hösnuva eller allergisk snuva under mer än 4 dagar under en enskild vecka?

❍ Ja [1] ❍ Vet ej / vill ej svara [998]
❍ Nej [2]

Om IA294 är "ja", visa IA295

[all_nasal_4days_cont]

IA295. Hände detta under mer än 4 veckor i sträck?

❍ Ja [1] ❍ Vet ej / vill ej svara [998]
❍ Nej [2]

[all_itchwat_eyes]

IA296. Har {ditt barn, du} klåda eller irritation i ögonen samtidigt med {sina, dina} näsbesvär?

❍ Ja [1] ❍ Vet ej / vill ej svara [998]
❍ Nej [2]

[all_nasal_diag]

IA297. Har en läkare ställt diagnosen hösnuva eller allergisk snuva?

❍ Ja [1] ❍ Vet ej / vill ej svara [998]
❍ Nej [2]

Om IA297 är "ja", visa IA298

[all_nasal_diag_age]

IA298. Vid vilken ålder fick {ditt barn, du} diagnosen hösnuva eller allergisk snuva?

❍ (År gammal) [yrs]_____ [yrs_other] ❍ Vet ej / vill ej svara [998]

- **F. VILLKORET AVSLUTAT**

Födoämnesallergi

[all_food_ill]

IA300. Har {ditt barn, du} någonsin blivit sjuk eller reagerat på något annat sätt på något speciellt födoämne?

❍ Ja [1] ❍ Vet ej / vill ej svara [998]
❍ Nej [2]

Om "ja", visa IA310

[all_food_cont]

IA310. Blir {ditt barn, du} nästan alltid sjuk på samma sätt eller får samma typ av besvär när {han/hon, du} äter detta födoämne?

❍ Ja [1] ❍ Vet ej / vill ej svara [998]
❍ Nej [2]

❖ **G. OM SVARET PÅ FRÅGORNA IA300 OCH IA310 ÄR "JA" VISA NEDANSTÅENDE FRÅGOR**

[all_food_kind]

IA320. Vilket eller vilka födoämnen får {ditt barn, du} dessa besvär av? (Markera alla aktuella)

❑ Ägg [egg]
❑ Fisk, skaldjur [fis]
❑ Jordnötter [pea]
❑ Nötter (t.ex. hasselnöt, mandel) [nut]
❑ Kärn- och stenfrukter (t.ex. äpple, päron, persika, kiwi) [pip]
❑ Spannmål (t.ex. vete, havre, råg, korn) [gra]
❑ Mjölk [mil]
❑ Annat [oth]

❍ Vet ej / vill ej svara [998]

[all_food_symp]

IA330. Vilken reaktion får {ditt barn, du}? (Markera alla aktuella)

❑ Klåda eller svullnad i mun eller svalg [ras]
❑ Svullnad på läppar, i ansikte eller öron [dia]
❑ Besvär från ögon eller näsa [nos]
❑ Astma eller andra andningsbesvär [ast]
❑ Magkramp eller kräkningar [bre]
❑ Kliande utslag eller rodnad över större delen av kroppen [who]
❑ Allergichock (anafylaktisk chock) [cho]
❍ Vet ej / vill ej svara [998]

❑ Annan[oth]

Om IA330 är "allergichock…", visa IA335

[all_food_anaphylactic]

IA335. Har en läkare konstaterat anafylaktisk chock (allergichock)?

❍ Ja [1]
❍ Nej [2]

❍ Vet ej / vill ej svara [998]

❖ **G. VILLKORET AVSLUTAS**

Andra allergier

[all_whc]

IA340. Upplever du att {ditt barn, du} har någon av följande allergier?

Ja
❑ Pollenallergi [poll]
❑ Pälsdjursallergi [pet]
❑ Kvalsterallergi [mite]
❑ Bi- eller getingallergi [bee]
❑ Kontaktallergi [cont]
❑
❍ Nej [no]
❍ Vet ej/ vill ej svara [998]

❖ **H. OM NÅGON ALLERGI ÄR MARKERAD I IA340 VISA NEDAN FRÅGOR**

Om "pollenallergi", visa IA350 och IA360

[all_whc_pollen_type]

IA350. Vilken sorts pollenallergi har {ditt barn, du}?

❑ Träd [tre] ❑ Gräs [gra]
❍ Vet ej / vill ej svara [998]

[all_whc_pollen]

IA360. Har en läkare ställt diagnosen pollenallergi?

❍ Ja [1] ❍ Vet ej / vill ej svara [998]
❍ Nej [2]

Om "pälsdjursallergi", visa IA370

[all_whc_pets]

IA370. Har en läkare ställt diagnosen pälsdjursallergi?

❍ Ja [1] ❍ Vet ej / vill ej svara [998]
❍ Nej [2]

Om "kvalsterallergi", visa IA375

[all_whc_mites]

IA375. Har en läkare ställt diagnosen kvalsterallergi?

❍ Ja [1] ❍ Vet ej / vill ej svara [998]
❍ Nej [2]

Om "bi eller geting allergi", visa IA380

[all_whc_bees]

IA380. Har en läkare ställt diagnosen bi- eller getingallergi?

❍ Ja [1] ❍ Vet ej / vill ej svara [998]
❍ Nej [2]

Om "kontaktallergi", visa IA390

[all_whc_contact]

IA390. Har en läkare ställt diagnosen kontaktallergi?

❍ Ja [1] ❍ Vet ej / vill ej svara [998]
❍ Nej [2]

❖ **H. VILLKORET AVSLUTAS**

Medicinering

❖ **I. OM IA290 ÄR "JA…", IA310 ELLER IA340 ÄR "JA" VISA NEDAN FRÅGOR**

[all_spray]

IA392. Har {ditt barn, du} under det senaste året använt nässpray som innehåller kortison - till exempel Nasonex, Flutide Nasal, Rhinocort eller Budesonide Nasal – för allergisk snuva?

❍ Ja, regelbundet [1]
❍ Ja, i perioder [2]
❍ Nej [3]
❍ Vet ej / vill ej svara [998]

[all_antihist]

IA394. Har {ditt barn, du} under det senaste året använt antihistamin-tabletter - till exempel Loratadin, Clarityn, Aerius, Cetirizin, Kestin eller Telfast - för {sin, din} allergiska snuva?

❍ Ja, regelbundet [1]
❍ Ja, i perioder [2]
❍ Nej [3]
❍ Vet ej / vill ej svara [998]

Kronisk bihåleinflammation

[all_rhinit]

IA400. Har {ditt barn, du} i mer än 12 veckor under de senaste 12 månaderna haft besvär av något av följande?

Ja
❑ Täppt näsa [stu]
❑ Snuva (snor eller slem) [dis]
❑ Värk eller tryck runt pannan, näsan och ögonen [fac]
❑ Försämrat eller inget luktsinne [red]
❑
❍ Nej [no]
❍ Vet ej / vill ej svara [998]

Om två eller fler svar är "markerade" i IA400, visa IA410

[all_chronrhinit_diag]

IA410. Har en läkare ställt diagnosen kronisk bihåleinflammation (rinosinuit)?

❍ Ja [1] ❍ Vet ej / vill ej svara [998]
❍ Nej [2]

❖ **I. VILLKORET AVSLUTAS**

Mental hälsa – skreening och personlighetsfrågor

Innehåll

Innehåll 1
Personlighetsfrågor 2
Skreeningfrågor 4
Depression 4
Perinatal depression 5
Premenstruell dysforisk störning 6
Suicidalitet (inkl. följdfrågor) 7
Generaliserat ångestsyndrom 11
Paniksyndrom / panikångest 12
Agorafobi 13
Tvångssyndrom (OCD) 14
Posttraumatiskt stressyndrom 15
Social fobi 16
Specifika fobier (inkl. följdfrågor) 16
ADHD (inkl. följdfrågor) 17
Traumatiska livshändelser 20
Kroniskt trötthetssyndrom (inkl. följdfrågor) 22
Spelvanor (inkl. följdfrågor) 26
Tourette’s syndrom (inkl. följdfrågor) 28

Personlighetsfrågor

Den följande sektionen handlar om skillnader i attityder, intressen och känslor. Det finns inget rätt eller fel svar till frågorna. Dina svar beskriver hur du tänker, känner och agerar i olika situationer. Vi ber dig att tänka på hur du tänker, känner och agerar i allmänhet. Tänk inte för länge på någon fråga.

[per_1]

	Ja [1]	Nej [2]	Vet ej / vill ej svara [998]
Vill du ha mycket liv och rörelse omkring dig? [1]	☐	☐	☐
Är du orolig och känner att det är något du vill ha, utan att veta vad? [2]	☐	☐	☐
Har du ett svar till hands när folk talar till dig? [3]	☐	☐	☐
Är du glad eller ledsen utan särskild anledning? [4]	☐	☐	☐
Håller du dig gärna i bakgrunden när du är tillsammans med andra? [5]	☐	☐	☐
Anser du dig vara glad och bekymmerslös? [6]	☐	☐	☐
Beslutar du dig för sent? [7]	☐	☐	☐
Känner du dig trött och håglös utan särskild anledning? [8]	☐	☐	☐
Har du ett livligt sätt? [9]	☐	☐	☐

[per_2]

Kan du snabbt klargöra i ord vad du tänker? [1]	☐	☐	☐
Är du försjunken i tankar? [2]	☐	☐	☐
Har du något emot att sälja saker eller att be folk om pengar för något välgörande ändamål? [3]	☐	☐	☐
Är du överdrivet känslig i vissa sammanhang? [4]	☐	☐	☐

	Ja [1]	Nej [2]	Vet ej / vill ej svara [998]
Är du någonsin så rastlös att du inte kan sitta still? [5]	☐	☐	☐
Håller du saker och ting för dig själv utom tillsammans med goda vänner? [6]	☐	☐	☐
Har du nervösa besvär? [7]	☐	☐	☐
Tycker du om att skämta och berätta roliga historier för dina vänner? [8]	☐	☐	☐
Brukar du bekymra dig alltför länge efter en pinsam upplevelse? [9]	☐	☐	☐
Anser du dig vara en nervös person? [10]<br><br>[per_3]	☐	☐	☐
Går du och oroar dig över saker och ting? [1]	☐	☐	☐
Känner du dig uttråkad på allt? [2]	☐	☐	☐
Bekymrar du dig över saker som du inte borde ha sagt eller gjort? [3]	☐	☐	☐
Har du lätt för att bli sårad och ta åt dig? [4]	☐	☐	☐
Anser du dig vara spänd eller nervös? [5]	☐	☐	☐
Blir du lätt sårad när folk påpekar fel på dig eller på det du gör? [6]	☐	☐	☐
Plågas du av skuldkänslor? [7]	☐	☐	☐
Är du lättirriterad? [8]	☐	☐	☐

[per_4]

	Stämmer inte alls [5]	Stämmer inte så bra [4]	Stämmer varken eller [3]	Stämmer ganska bra [2]	Stämmer precis [1]
Jag gillar att lösa problem och gåtor [1]	☐	☐	☐	☐	☐
Det är lätt för mig att ha medkänsla för andra [2]	☐	☐	☐	☐	☐
Jag har en stor intellektuell nyfikenhet [3]	☐	☐	☐	☐	☐
Jag tycker det är intressant att börja med nya hobbier [4]	☐	☐	☐	☐	☐
Jag gillar att fundera kring teorier eller filosofiska idéer [5]	☐	☐	☐	☐	☐
Jag provar ofta ny och utländsk mat [6]	☐	☐	☐	☐	☐

Skreeningfrågor

Depression

[scr_sc21]

SC21. Har du någon gång i livet haft en period som varat i 2 veckor eller längre då du kände dig ledsen, tom eller deprimerad mest hela dagen?

❍ Ja [1] ❍ Vet ej / vill ej svara [998]

❍ Nej [2]

[scr_sc23]

SC23. Har du någonsin haft en period som varat i 2 veckor eller längre då du tappade intresset för sådant som du brukar tycka om som t.ex. arbete, hobbier och personliga relationer?

❍ Ja [1] ❍ Vet ej / vill ej svara [998]

❍ Nej [2]

- **A. OM SC21 ELLER SC23 ÄR "JA" AKTIVERA FÖLJDMODULEN FÖR DEPRESSION**

Perinatal depression

- **A. LÄMNA OM MN150 ÄR "NEJ" (DVS. EJ FÖTT BARN)**

[scr_pn1]

PN10. Har du känt dig ledsen, olycklig, eller mycket orolig under någon av dina graviditeter? Då avses en period på minst 2 veckor när du inte var dig själv och som definitivt var värre än livets normala svängningar.

❍ Ja [1] ❍ Vet ej / vill ej svara [998]

❍ Nej [2]

[scr_pn2]

PN20. Inom sex månader efter någon av dina förlossningar, hände det att du kände dig ledsen, olycklig, eller mycket orolig? Då menar vi en period på minst 2 veckor när du inte var dig själv och som definitivt var värre än livets normala svängningar.

❍ Ja [1]　❍ Vet ej / vill ej svara [998]

❍ Nej [2]

- **B. OM PN10 OCH PN20 ÄR "JA", AKTIVERA FÖLJDMODULEN FÖR PERINATAL DEPRESSION**

Premenstruell dysforisk störning

- **A. AVSLUTA OM KÖN ÄR "MAN" ELLER ÅLDER <15**

Om MN20 inte är ”nej” (ej haft mens), visa PMS10 och PMS15

[scr2_pms_1]

PMS10. Har du, under de flesta menstruationscykler under senaste året, haft humörförändringar och/eller kroppsliga symptom under veckan före menstruationen, dvs. det man brukar kalla PMS?

❍ Ja [1]　❍ Vet ej / vill ej svara [998]

❍ Nej [2]

[scr2_pms_1_5]

PMS15. Har du under det senaste året tagit medicin för PMS?

❍ Ja [1]　❍ Vet ej / vill ej svara [998]

❍ Nej [2]

Om PMS10 är "ja", visa PMS20

[scr2_pms_2]

PMS20. Har dina PMS-besvär varit så allvarliga att de påverkat dina relationer med andra, eller din förmåga att utföra arbete eller andra aktiviteter?

❍ Ja [1] ❍ Vet ej / vill ej svara [998]

❍ Nej [2]

Om PMS20 är "ja", visa PMS30

[scr2_pms_3]

PMS30. Är du helt säker på att besvären är begränsade till den premenstruella perioden, dvs. att du alltid är helt besvärsfri cirka en vecka efter att menstruationen har startat?

❍ Ja [1] ❍ Vet ej / vill ej svara [998]

❍ Nej [2]

- **B. AKTIVERA MODULEN OM PREMENSTRUELL DYSFORISK STÖRNING OM PMS30 ÄR "JA"**

Suicidalitet (inkl. följdfrågor)

[scr_sd2]

SD2. Har du någonsin allvarligt funderat på att begå självmord?

❍ Nej [3]

❍ Ja, en gång [1]

❍ Ja, flera gånger [2]

❍ Vet ej / vill ej svara [998]

❖ **A. LÄMNA MODULEN OM SD2 ÄR "NEJ"**

[scr2_sd2a]

SD2a. Hur gammal var du {när, första gången} detta hände?

❍ 9 år eller yngre [1] ❍ Vet ej / vill ej svara [998]
❍ 10 till 14 år [2]
❍ 15 till 19 år [3]
❍ 20 till 24 år [4]
❍ 25 till 29 år [5]
❍ 30 till 34 år [6]
❍ 35 till 39 år [7]
❍ 40 år eller äldre [8]

Om SD2 är "ja, flera gånger", visa SD3a

[scr2_sd3a]

SD3a. Hur gammal var du senast detta hände?

❍ 9 år eller yngre [1] ❍ Vet ej / vill ej svara [998]
❍ 10 till 14 år [2]
❍ 15 till 19 år [3]
❍ 20 till 24 år [4]
❍ 25 till 29 år [5]
❍ 30 till 34 år [6]
❍ 35 till 39 år [7]
❍ 40 år eller äldre [8]

[scr_sd4]

SD4. Har du någonsin gjort upp en plan för att begå självmord?

❍ Nej [2]

❍ Ja [1]

❍ Vet ej / vill ej svara [998]

❖ **B. LÄMNA MODULEN OM SD4 ÄR "NEJ"**

[scr_sd6]

SD6. Har du någonsin försökt att begå självmord?

❍ Ja [1]   ❍ Vet ej / vill ej svara [998]

❍ Nej [2]

❖ **C. LÄMNA MODULEN OM SD6 ÄR "NEJ"**

[scr_sd6a]

SD6a. Hur många gånger har du försökt ta livet av dig under din livstid?

❍ (Antal gånger) [tms]____________ [tms_other]

❍ Vet ej / vill ej svara [998]

[scr2_sd8]

SD8. Hur gammal var du {då, första gången}?

❍ 9 år eller yngre [1]   ❍ Vet ej / vill ej svara [998]
❍ 10 till 14 år [2]
❍ 15 till 19 år [3]
❍ 20 till 24 år [4]
❍ 25 till 29 år [5]
❍ 30 till 34 år [6]
❍ 35 till 39 år [7]
❍ 40 år eller äldre [8]

Om "antal gånger" i SD6a är mer än 1, visa a annars b

[scr_sd9]

SD9. {**a**: Tänk på det mest allvarliga av dina försök att begå självmord **b**: Tänk på ditt försök att begå självmord}. Vilket av dessa tre påståenden beskriver bäst vad som hände dig?

❍ Jag gjorde ett allvarligt försök att ta livet av mig och det var bara tur att jag inte lyckades [1]

❍ Jag försökte ta livet av mig, men jag visste att metoden inte var helt säker [2]

❍ Mitt självmordsförsök var ett rop på hjälp. Jag hade inte för avsikt att dö [3]

❍ Vet ej / vill ej svara [998]

[scr_sd11]

SD11. Resulterade det i skada eller förgiftning?

❍ Ja [1] ❍ Vet ej / vill ej svara [998]

❍ Nej [2]

[scr_sd12]

SD12. Krävdes det medicinsk behandling?

❍ Ja [1] ❍ Vet ej / vill ej svara [998]

❍ Nej [2]

Om SD12 är "ja", visa SD13

[scr_sd13]

SD13. Krävdes en övernattning på sjukhus?

❍ Ja [1] ❍ Vet ej / vill ej svara [998]

❍ Nej [2]

[scr_sd14_2]

SD14.2. Vilken metod använde du för att försöka begå självmord? (Markera alla alternativ som är relevanta.)

❑ Pistol [1]

❑ Rakblad, kniv eller annat vasst föremål [2]

❑ Överdos av receptbelagda medicine [3]

❑ Överdos av receptfria medicine [4]

❑ Överdos av annan drog (t.ex. heroin, crack, alkohol) [5]

❑ Hängning, strypning, kvävning [6]

❑ Drunkning [7]

❑ Hopp från hög plats [8]

❑ Bilkrasch [9]

❑ Annan [10]

❍ Vet ej / vill ej svara [998]

Generaliserat ångestsyndrom

[scr_gad]

G1. Har du någonsin haft en period i ditt liv, sex månader eller längre, när du hade betydligt mer ångest eller var betydligt mer orolig än de flesta andra personer med samma problem som du?

❍ Ja [1]   ❍ Vet ej / vill ej svara [998]

❍ Nej [2]

Om G1 är "ja", visa G2

[scr_gad_dep]

G2. Kände du dig också deprimerad under hela denna ångestperiod?

❍ Ja [1] ❍ Vet ej / vill ej svara [998]

❍ Nej [2]

Paniksyndrom / panikångest

[scr_sc20_1]

SC20.1. Har du någonsin i livet haft en oväntad attack av rädsla eller panik då du helt plötsligt kände något av följande:

– du kände dig mycket rädd, orolig eller olustig
– du fick svårt att andas, kände dig yr eller fick hjärtklappning
– du trodde att du skulle förlora kontrollen, dö eller bli galen?

❍ Ja [1] ❍ Vet ej / vill ej svara [998]

❍ Nej [2]

Om SC20.1 är "ja", visa SC20.2 och SC20.3

[scr_sc20_2]

SC20.2. Har en sådan attack av oförklarlig rädsla eller panik hänt flera gånger?

❍ Ja [1] ❍ Vet ej / vill ej svara [998]

❍ Nej [2]

[scr_sc20_3]

SC20.3. Har det alltid funnits en rimlig förklaring till varför känslan av rädsla eller panik uppstod?

❍ Ja [1] ❍ Vet ej / vill ej svara [998]

❍ Nej [2]

❖ **A. OM SC20.1, SC20.2 OCH SC20.3 ÄR "JA" AKTIVERA FÖLJDMODULEN FÖR PANIKSYNDROM/PANIKÅNGEST**

Agorafobi

❖ **A. OM VILLKORET A I SCREENINGMODULEN FÖR PANIKSYNDROM/PANIKÅNGEST ÄR UPPFYLLT VISA NEDANSTÅENDE FRÅGOR**

[scr_sc30_1]

SC30.1. Har det någonsin hänt under din livstid att du blev mycket upprörd eller nervös varje gång du var i folksamlingar, på allmän plats eller på resa?

❍ Ja [1] ❍ Vet ej / vill ej svara [998]

❍ Nej [2]

Om SC30.1 är "ja", visa SC30.2

[scr_sc30_2]

SC30.2. Försökte du undvika dessa situationer närhelst du kunde på grund av din rädsla?

❍ Ja [1] ❍ Vet ej / vill ej svara [998]

❍ Nej [2]

Om SC30.2 är "ja", visa SC30.3

[scr_sc30_3]

SC30.3. Tycker du att din rädsla var mycket starkare än den borde ha varit i dessa situationer?

❍ Ja [1] ❍ Vet ej / vill ej svara [998]

❍ Nej [2]

- **A. VILLKORET AVSLUTAS**
- **B. OM SC30.1, SC30.2 OCH SC30.3 ÄR "JA", AKTIVERA FÖLJDMODULEN FÖR AGORAFOBI**

Tvångssyndrom (OCD)

[scr_oc1]

OC1. Har du någonsin besvärats av ologiska tankar som återkom fastän du försökte att inte tänka på dem? Till exempel: att skada någon fastän du verkligen inte ville det eller att du smittas av bakterier eller blir nedsmutsad. Har du någon gång i livet haft den typen av tankar?

❍ Ja [1] ❍ Vet ej / vill ej svara [998]

❍ Nej [2]

[scr_oc2]

OC2. Har du någonsin känt att det finns något du måste göra upprepade gånger och att du inte kunde låta bli att göra det, som att tvätta händerna om och om

igen, att räkna till en viss siffra, eller att kontrollera om och om igen att du har gjort något på rätt sätt.

❍ Ja [1]   ❍ Vet ej / vill ej svara [998]

❍ Nej [2]

- **A. OM OC1 ELLER OC2 ÄR "JA", AKTIVERA FÖLJDMODULEN FÖR TVÅNGSSYNDROM/OCD**

Posttraumatiskt stressyndrom

[scr_pt1]

PT1. Ibland händer det saker som är mycket upprörande – det kan vara en livshotande situation som t.ex. en katastrof, en mycket allvarlig olycka eller brand; att bli fysiskt eller sexuellt antastad eller våldtagen, inklusive att bli misshandlad som barn, att se en annan person dö eller dödas, eller bli allvarligt skadad, eller att höra talas om något hemskt som har hänt någon närstående. Har något sådant någonsin hänt dig under din livstid?

❍ Ja [1]   ❍ Vet ej / vill ej svara [998]

❍ Nej [2]

[scr_pt2]

PT2. Har du någonsin deltagit i strid, antingen som militär eller som medlem i en icke militär organisation?

❍ Ja [1]   ❍ Vet ej / vill ej svara [998]

❍ Nej [2]

- **A. OM PT1 ELLER PT2 ÄR "JA", AKTIVERA FÖLJDMODULEN FÖR POSTTRAUMATISKT STRESSSYNDROM**

Social fobi

[scr_sc29x]

SC29. Har du någonsin i ditt liv känt dig mycket rädd för människor eller känt dig väldigt blyg för människor, som till exempel att möta nya människor, att vara i en grupp, att gå på fest, gå på date eller att använda en offentlig toalett?

❍ Ja [1] ❍ Vet ej / vill ej svara [998]

❍ Nej [2]

❖ **A. OM SC29 ÄR "JA", AKTIVERA FÖLJDMODULEN FÖR SOCIAL FOBI**

Specifika fobier (inkl. följdfrågor)

[scr_sc27]

SI10. Följande frågor handlar om saker som en del människor är rädda för fastän de vet att det inte finns någon riktig fara.

Är du rädd för följande:

	Nej [1]	Ja [2]	Vet ej / vill ej svara [998]
Insekter, ormar, hundar, eller något annat djur? [1]	☐	☐	☐
Stilla vatten, som i en swimmingpool eller sjö eller dramatiskt väder som t.ex. storm, åska eller blixt? [2]	☐	☐	☐
Instängda utrymmen, såsom i en grotta, tunnel, garderob eller hiss? [3]	☐	☐	☐
Högt belägna platser såsom på tak, balkong, bro eller trappa? [4]	☐	☐	☐

	Nej [1]	Ja [2]	Vet ej / vill ej svara [998]
Att flyga eller för flygplan? [5]	☐	☐	☐

❖ **A. LÄMNA MODULEN OM ALLA FRÅGOR I SI10 ÄR "NEJ"**

[scr_sc27_1]

SI20.

	Nej [1]	Ja [2]	Vet ej / vill ej svara [998]
Hände det någon gång i ditt liv att du blev väldigt upprörd eller nervös närhelst du stod inför denna eller dessa situationer? [1]	☐	☐	☐
Undvek du denna eller dessa situationer närhelst du kunde på grund av din rädsla? [2]	☐	☐	☐
Tycker du att din rädsla var mycket starkare än den borde ha varit? [3]	☐	☐	☐

ADHD (inkl. följdfrågor)

[scr_adhd1]

AD1-6. Hur ofta händer det att du

	Aldrig eller sällan [1]	Ibland [2]	Ofta [3]	Vet ej / vill ej svara [998]
Har du problem att komma ihåg bokade	☐	☐	☐	☐

	Aldrig eller sällan [1]	Ibland [2]	Ofta [3]	Vet ej / vill ej svara [998]
möten eller andra åtaganden? [reme]				
Undviker du eller har du svårt att komma igång med en uppgift som kräver mycket tankeverksamhet? [dela]	☐	☐	☐	☐
Har du svårt att få saker i ordning när du ska utföra en uppgift som kräver en viss organisation? [orde]	☐	☐	☐	☐
Har du svårt att avsluta de sista detaljerna i ett projekt när de utmanande delarna är klara? [wrap]	☐	☐	☐	☐
Rör du dina händer eller fötter oroligt när du måste sitta still länge? [fidg]	☐	☐	☐	☐
Känner du dig mycket aktiv och måste göra saker, som om du drevs av en motor? [acti]	☐	☐	☐	☐

❖ **A. LÄMNA OM MINST TRE AV FRÅGORNA MELLAN AD1 OCH AD6 ÄR MINDRE FREKVENTA ÄN "OFTA"**

[scr_adhd2]

	Aldrig eller nästan aldrig [1]	Ibland [2]	Ofta [3]	Vet ej /vill ej svara [998]
AD7. Hur ofta gör du slarviga misstag när du måste arbeta på ett tråkigt eller svårt projekt? [1]	☐	☐	☐	☐
AD8. Hur ofta har du svårt att behålla koncentrationen när du arbetar med något som är tråkigt eller repetitivt?	☐	☐	☐	☐

	Aldrig eller nästan aldrig [1]	Ibland [2]	Ofta [3]	Vet ej /vill ej svara [998]
[2]				
AD9. Hur ofta har du svårt att koncentrera dig på vad människor säger till dig fastän de pratar direkt till dig? [3]	☐	☐	☐	☐
AD10. Hur ofta förlägger du saker eller har svårt att hitta saker hemma eller på arbetet? [4]	☐	☐	☐	☐
AD11. Hur ofta blir du distraherad av aktiviteter eller ljud runt omkring dig? [5]	☐	☐	☐	☐
AD12. Hur ofta brukar du lämna din plats under möten eller i andra situationer där du förväntas sitta still? [6]	☐	☐	☐	☐

[scr_adhd3]

	Aldrig eller nästan aldrig [1]	Ibland [2]	Ofta [3]	Vet ej/ vill ej svara [998]
AD13. Hur ofta känner du dig rastlös eller orolig? [1]	☐	☐	☐	☐
AD14. Hur ofta har du svårt att varva ner och koppla av när du har tid för dig själv? [2]	☐	☐	☐	☐
AD15. Hur ofta finner du att du pratar för mycket när du är i sociala situationer? [3]	☐	☐	☐	☐
AD16. När du samtalar med någon, hur ofta händer det att du märker att du avslutar meningar åt den du talar med	☐	☐	☐	☐

	Aldrig eller nästan aldrig [1]	Ibland [2]	Ofta [3]	Vet ej/ vill ej svara [998]
innan de kan avsluta dem själva? [4]				
AD17. Hur ofta har du svårt att vänta på din tur i situationer där man förväntas att turas om? [5]	☐	☐	☐	☐
AD18. Hur ofta händer det att du avbryter andra när de är upptagna? [6]	☐	☐	☐	☐

Traumatiska livshändelser

[scr_sl1]

SL1. Har något av följande hänt dig under din livstid? (Markera alla aktuella)

❑ Egen skilsmässa eller separation [1]

❑ Allvarliga ekonomiska problem (t.ex. inga pengar till mat) [2]

❑ Blivit diskriminerad på ett mycket besvärande sätt [3]

❑ Varit utsatt för mobbning [4]

❑

❑ Bevittnat en allvarlig olycka [6]

❑ Varit involverad i en allvarlig olycka [7]

❑

❑ Varit med om en naturkatastrof (t.ex. tsunami, orkan, skogsbrand) [8]

❑ Åkt in i fängelse för ett brott [9]

❑ Nära familjemedlem som åkt in i fängelse [10]

❑

❍ Inget av dessa [12]

❍ Vet ej / vill ej svara [998]

[scr_sl2]

SL2. Har något av följande hänt dig under din livstid? (Markera alla aktuella)

❑ En nära vän som dött [1]

❑ Blivit adopterad eller placerad i

❑

❑ Allvarlig fysisk eller psykisk sjukdom [3]

❑ En nära familjemedlem med allvarlig fysisk eller psykisk sjukdom [14]

❑ En nära familjemedlem som dött (förutom barn) [4]

❑ Ett barn som dött [5]

❑

familjehem / fosterhem [7]

❑ Före 18 år varit med om en skilsmässa eller separation mellan dina föräldrar [8]

❑ Separerats från ditt barn mot din vilja [9]

❑ Före 18 år blivit vittne till misshandel mellan familjemedlemmar [fam]

❑ Blivit fysiskt försummad (t.ex. inte fått mat) [10]

❑ Blivit känslomässigt misshandlad eller försummad (t.ex. ofta fått skämmas) [11]

❑

❍ Inget av dessa [13]

❍ Vet ej / vill ej svara [998]

[scr_sl3]

SL3. Har du upplevt något av följande? (Markera alla aktuella)

❑ Varit vittne till ett rån eller personrån [robs]

❑ Varit utsatt för ett rån eller personrån [robv]

❑ Blivit förföljd, hotad att bli dödad eller allvarligt skadad [3]

❑ Före 18 år varit utsatt (ej sexuellt) eller misshandlad av någon du kände [5]

❑ Som vuxen varit utsatt (ej sexuellt) eller misshandlad av någon du kände [6]

❑ Besvärats eller trakasserats med sexuella tillmälen [sexb]

❑ Före 18 år blivit utsatt för sexuellt ofredande eller tvingats till sexuella handlingar mot din vilja [sext]

❑ Som vuxen blivit utsatt för sexuellt ofredande eller tvingats till sexuella handlingar [9]

❑ Före 18 år tvingats att ha sex [sexf]

❑ Tvingats att ha sex som vuxen [11]

❑ Haft sex i utbyte mot pengar eller droger [12]

❑

❍ Inget av dessa [14]

❍ Vet ej / vill ej svara [998]

[scr_sl4]

SL4. Har du varit allvarligt upprörd över att någon närstående varit utsatt för någon av dessa stressande livshändelser?

❍ Ja [1] ❍ Vet ej / vill ej svara [998]

❍ Nej [2]

Kroniskt trötthetssyndrom (inkl. följdfrågor)

Om man eller om kvinna och inte gravid (svarat ”ja” på MN150), visa CF10 till CF140

[scr_cf_6mntsabtired]

CF10. Har du känt dig onormalt trött under de senaste sex månaderna?

❍ Ja [1] ❍ Vet ej / vill ej svara [998]

❍ Nej [2]

Om CF10 är "ja", visa CF20

[scr_cf_6mntalltme]

CF20. Kände du denna trötthet under hela sexmånadersperioden?

❍ Ja [1] ❍ Vet ej / vill ej svara [998]

❍ Nej [2]

Om CF20 är "ja", visa CF30

[scr_cf_6mntnrmlife]

CF30. Kände du dig för trött för att leva ett normalt liv under dessa sex månader?

❍ Ja [1]   ❍ Vet ej / vill ej svara [998]

❍ Nej [2]

Om kön är "kvinna" och MN150 är "ja", visa CF40

[scr_cf_pregntired]

CF40. Inträffade den ovanliga tröttheten bara under graviditet?

❍ Ja [1]   ❍ Vet ej / vill ej svara [998]

❍ Nej [2]

- **A. OM CF10, CF20 OCH CF30 ÄR "JA" OCH OM CF40 ÄR "NEJ" FÖR KVINNA SOM SVARAT "JA" PÅ MN150, VISA NEDANSTÅENDE FRÅGOR**

[scr_cf_period_dur]

CF50. Under hur lång tid åt gången har du känt denna onormala trötthet?

❍ (Antal veckor) [wks] ____________[wks_other]

❍ (Antal månader) [mnt] ____________[mnt_other]

❍ (Antal år) [yrs] ____________[yrs_other]

❍ Vet ej / vill ej svara [998]

[scr2_cf_onset]

CF60. Hur gammal var du när problemet började?

❍ 9 år eller yngre [1]   ❍ Vet ej / vill ej svara [998]
❍ 10 till 14 år [2]
❍ 15 till 19 år [3]
❍ 20 till 24 år [4]
❍ 25 till 29 år [5]

❍ 30 till 34 år [6]
❍ 35 till 39 år [7]
❍ 40 till 44 år [8]
❍ 45 till 49 år [9]
❍ 50 år eller äldre [10]

[scr_cf_disease]

CF70. Lider du av någon sjukdom som orsakar denna långvariga trötthet?

❍ Ja [1] ❍ Vet ej / vill ej svara [998]

❍ Nej [2]

Om CF70 är "ja", visa CF80

[scr_cf_diag]

CF80. Har en läkare ställt diagnosen för denna sjukdom?

❍ Ja [1] ❍ Vet ej / vill ej svara [998]

❍ Nej [2]

[scr_cf_work_able]

CF90. Är din arbetsförmåga nedsatt på grund av tröttheten?

❍ Ja, oförmögen att arbeta eller är sjukskriven eller har förtidspension på grund av trötthet eller smärta [1]

❍ Ja, delvis nedsatt arbetsförmåga (%)[2]______ [2_other]

❍ Nej [3]

❍ Vet ej /vill ej svara [998]

[scr_cf_social]

CF100. Medför tröttheten att du har en nedsatt förmåga vad gäller ditt sociala liv, som t.ex. fritidsaktiviteter?

❍ Ja [1] ❍ Vet ej / vill ej svara [998]

❍ Nej [2]

[scr_cf_suffer]

CF110. Under den senaste sexmånadersperioden av onormal trötthet, led du också av följande? (Markera alla aktuella)

❑ Vaknade utan att känna dig riktigt utvilad [1]

❑ Problem med korttidsminnet eller svårigheter med koncentrationen [2]

❑ Mådde illa i 24 timmar eller mer efter ansträngning [3]

❑ Ny typ av huvudvärk [4]

❑ Ont i halsen [5]

❑ Ömhet i lymfkörtlarna i hals eller armhåla [6]

❑ Muskelsmärta [7]

❑ Värk i flera leder utan rodnad eller svullnad [8]

❍ Inget av dessa [996]

❑

❍ Vet ej / vill ej svara [998]

Om CF110 är ”mådde illa i 24 timmar...”, visa CF120

[scr_cf_sick24h_over]

CF120. Under perioden av onormal trötthet efter ansträngning, mådde du illa i mer än 24 timmar?

❍ Ja [1]

❍ Nej [2]

❍ Vet ej / vill ej svara [998]

[scr_cf_relief]

CF140. Om du skulle vila i några dagar, i vilken utsträckning skulle tröttheten lindras?

❍ Helt och hållet [1]

❍ Något [2]

❍ Inte alls [3]

❍ Vet ej / vill ej svara [998]

❖ **A. VILLKORET AVSLUTAS**

Spelvanor (inkl. följdfrågor)

[scr_ga_12mnts_gamble]

GA1. Hur ofta har du spelat under de senaste 12 månaderna?

Då inkluderas att spela på hästar, spela Bingo, spela på Lotto, spela på Postkodlotteriet, spela i sportsliga sammanhang, spela på casino, spela på spelautomater (förutom casino), spela poker för pengar, och att spela på internet.

❍ Aldrig eller några få gånger [4]
❍ Dagligen [1]
❍ Varje månad [3]
❍ Vet ej / vill ej svara [998]
❍ Varje vecka [2]

❖ **A. LÄMNA MODULEN OM GA1 ÄR "ALDRIG ELLER NÅGRA GÅNGER"**

[scr_ga_12mnts]

	Aldrig [4]	Ibland [3]	För det mesta [2]	Nästan alltid [1]	Vet ej / vill ej svara [998]
GA2. Hur ofta har du spelat för mer pengar än du egentligen hade råd att förlora? [betmrelose]	☐	☐	☐	☐	☐

	Aldrig [4]	Ibland [3]	För det mesta [2]	Nästan alltid [1]	Vet ej / vill ej svara [998]
GA3. Hur ofta har du behövt spela för större summor pengar för att uppnå samma spänningskänsla? [betsamefeel]	☐	☐	☐	☐	☐
GA4. Hur ofta har du gått tillbaka en annan dag för att försöka vinna tillbaka de pengar du förlorat? [winback]	☐	☐	☐	☐	☐
GA5. Hur ofta har du lånat pengar eller sålt något för att få pengar att spela med? [borrow]	☐	☐	☐	☐	☐
GA6. Hur ofta har du känt att du kan ha spelproblem? [hveprob]	☐	☐	☐	☐	☐
GA7. Hur ofta har du känt att spelandet har förorsakat något hälsoproblem, inklusive stress eller oro? [csehealth]	☐	☐	☐	☐	☐
GA8. Hur ofta har folk kritiserat ditt spelande eller sagt att du har spelproblem, oavsett om du tyckte att det var sant eller ej? [critiz]	☐	☐	☐	☐	☐
GA9. Hur ofta har ditt spelande orsakat ekonomiska problem för dig eller för ditt hushåll? [finaprob]	☐	☐	☐	☐	☐
GA10. Hur ofta har du känt skuld över ditt spelande eller vad som händer när du spelar? [guilt]	☐	☐	☐	☐	☐

Tourette’s syndrom (inkl. följdfrågor)

[scr_to_motor]

TO10. Har du någonsin haft motoriska tics – d.v.s. upprepade, ofrivilliga rörelser som du inte kan sluta med?

❍ Ja [1]   ❍ Vet ej / vill ej svara [998]

❍ Nej [2]

[scr_to_vocal]

TO20. Har du någonsin haft vokala tics – d.v.s. upprepade, ofrivilliga ljud som du inte kan sluta med?

❍ Ja [1]   ❍ Vet ej / vill ej svara [998]

❍ Nej [2]

- **A. OM TO10 ÄR "JA" VISA TO30 OCH TO35.**
- **B. OM TO20 ÄR "JA" VISA TO40 OCH TO45.**

[scr_to_motortype]

TO30. Vilken eller vilka av följande motoriska tics har du någonsin haft?
(Markera alla aktuella)

❑ Överdrivet blinkande med ögonen [1]

❑ Höja ögonbrynen [2]

❑ Skela med ögonen [3]

❑ Rulla med ögonen, upp, ner eller åt sidan [4]

❑ Ha ryckningar i näsan [5]

❑ Spänna ut näsvingarna [6]

❑ Puta med munnen (som vid en puss) [7]

❑ Gapa stort [8]

❑ Nicka med huvudet [9]

❑ Skrynkla ihop ansiktet [10]

❑ Röra axeln med hakan [11]

❑ Sträcka nacken [12]

❑ Rycka på axlarna [13]

❑ Plötslig rörelse med arm eller ben [14]

❑ Andra motoriska tics [15]

[scr_to_motorbegan]

TO35. Hur gammal var du när dina motoriska tics började första gången?

❍ Jag har haft det sedan födseln [1]

❍ (År gammal) [yrs]____________ [yrs_other]

❍ Vet ej / vill ej svara [998]

[scr_to_vocaltype]

TO40. Vilken eller vilka av följande vokala tics har du någonsin haft? (Markera alla aktuella)

❑ Harkla dig [1]

❑ Överdrivet sniffande [2]

❑ Hosta vanemässigt [3]

❑ Sväljande [4]

❑ Höga pip [5]

❑ Små ljud som 'Åh', 'Äh', 'Öh' [6]

❑ Sugande ljud [7]

❑ Rapar, inte bara när du äter eller dricker [8]

❑ Ett ord som sägs upprepat och utan sammanhang [9]

❑ Svärande, utan avsikt och utan att du är irriterad [10]

❑ Andra vokala tics [11]

[scr_to_vocalbegan]

TO45. Hur gammal var du när dina vokala tics började första gången?

❍ Jag har haft det sedan födseln [1]

❍ (År gammal) [yrs]_____________ [yrs_other]

❍ Vet ej / vill ej svara [998]

Mental hälsa - uppföljningsfrågor

Innehåll

Innehåll 1

Depression 2

Perinatal depression 13

Premenstruella symtom 16

Paniksyndrom / panikångest 20

Agorafobi 24

Tvångssyndrom (OCD) 31

Posttraumatiskt stressyndrom 43

Social fobi 67

Depression

- **A. OM VILLKORET A I SCREENINGMODULEN FÖR DEPRESSION ÄR SANT, VISA NEDANSTÅENDE FRÅGOR**

[dep_d2]

D2. Du nämnde tidigare att du har haft perioder som varade i flera dagar eller längre då du kände dig ledsen, nedstämd eller deprimerad största delen av dagen.

Inträffade perioderna alltid i samband med en speciellt besvärande händelse?

Ja

❑ Efter att ha förlorat någon närstående [clo]

❑ Efter kroppslig skada eller sjukdom [bod]

❑ Efter annan besvärande händelse [oth]

❍ Nej, inte relaterat till en besvärande händelse [no]

❍ Vet ej / vill ej svara [998]

[dep_d3]

D3. För nästkommande frågor, tänk på den tvåveckorsperiod då dessa känslor var som värst. Hur länge varade känslan av att vara ledsen, nedstämd eller deprimerad?

❍ Hela dagen eller större delen av dagen [1]

❍ Mindre ofta [2]

❍ Vet ej / vill ej svara [998]

- **B. OM D3 ÄR “HELA DAGEN ELLER NÄSTAN HELA DAGEN”, VISA NEDANSTÅENDE FRÅGOR**

[dep_d4]

D4. Under de två veckorna, hur ofta kände du dig ledsen, nedstämd eller deprimerad?

❍ Varje dag eller nästan varje dag [1]

❍ Mindre ofta [2]

❍ Vet ej / vill ej svara [998]

Om D4 är “varje dag eller nästan varje dag”, visa D5 och D6

[dep_d5]

D5. Under dessa två veckor, tappade du intresset för det mesta i livet?

❍ Ja [1] ❍ Vet ej / vill ej svara [998]

❍ Nej [2]

- **B. VILLKORET AVSLUTAS**
- **C. OM D3 ÄR “MINDRE OFTA” VISA NEDANSTÅENDE FRÅGOR**

[dep_d8]

D8. Tänk på den tvåveckorsperiod då känslan av att ha helt förlorat intresset för allt var som starkast. Hur länge varade känslan av att ha förlorat intresset för allt?

❍ Hela dagen eller större delen av dagen [1]

❍ Mindre ofta [2]

❍ Vet ej / vill ej svara [998]

Om D8 är "hela dagen eller större delen av dagen", visa D9 och D10

[dep_d9]

D9. Hur ofta under de två veckorna kändes det som om du hade förlorat intresset för allt?

❍ Varje dag eller nästan varje dag [1]

❍ Mindre ofta [2]

❍ Vet ej / vill ej svara [998]

- **C. VILLKORET AVSLUTAS**
- **D. OM D3 ÄR "HELA DAGEN ELLER STÖRRE DELEN AV DAGEN" ELLER D9 ÄR "VARJE DAG ELLER NÄSTAN VARJE DAG", VISA NEDANSTÅENDE FRÅGOR**

[dep_d10]

D10. Kände du dig konstant trött eller utan energi under dessa två veckor?

❍ Ja [1] ❍ Vet ej / vill ej svara [998]

❍ Nej [2]

[dep_d11]

D11. Ökade eller minskade din aptit väldigt mycket under den här tiden?

❍ Aptiten förblev ungefär densamma [1]

❍ Aptiten ökade mycket [2]

❍ Aptiten minskade mycket [3]

❍ Båda – aptiten ökade mycket ibland och aptiten minskade mycket ibland [4]

❍ Vet ej / vill ej svara [998]

[dep_d12]

D12. Förändrades din vikt under den här tiden?

❍ Vikten förblev ungefär densamma [1]

❍ Du gick upp i vikt [2]

❍ Du gick ned i vikt [3]

❍ Du gick både upp och ned i vikt [4]

❍ Vet ej / vill ej svara [998]

Om D12 är "gick upp i vikt" eller "gick ned i vikt", visa D13

[dep_d13]

D13. Ungefär hur många kilo gick du {upp, ned}?

❍ 1 kg [1]

❍ 2 kg [2]

❍ 3 kg [3]

❍ 4 kg [4]

❍ 5 kg eller mer [5]

❍ Vet ej / vill ej svara [998]

[dep_d15]

D15. Sov du mycket mindre än vanligt?

❍ Ja [1] ❍ Vet ej / vill ej svara [998]

❍ Nej [2]

Om D15 är "ja", visa D16 och D17

[dep_d16]

D16. Hade du svårare att somna, att fortsätta sova, eller vaknade du för tidigt?

❍ Ja [1]   ❍ Vet ej / vill ej svara [998]

❍ Nej [2]

Om D16 är "ja", visa D17

[dep_d17]

D17. Hur ofta hade du svårt att somna under dessa två veckor?

❍ Varje natt eller nästan varje natt [1]

❍ Mindre ofta [2]

❍ Vet ej / vill ej svara [998]

[dep_d18]

D18. Sov du mycket mer än vanligt?

❍ Ja [1]   ❍ Vet ej / vill ej svara [998]

❍ Nej [2]

Om D18 är "ja", visa D19

[dep_d19]

D19. Under dessa två veckor, hur ofta sov du mycket mer än vanligt?

❍ Varje natt eller nästan varje natt [1]   ❍ Mindre ofta [2]

❍ Vet ej / vill ej svara [998]

[dep_d20]

D20. Var det mycket svårare för dig än det brukar vara att kunna tänka eller att koncentrera dig?

❍ Ja [1] ❍ Vet ej / vill ej svara [998]

❍ Nej [2]

[dep_d21]

D21. Under den här tiden, var du så nervös eller rastlös att du inte kunde sitta stilla så att andra människor lade märke till det?

❍ Ja [1] ❍ Vet ej / vill ej svara [998]

❍ Nej [2]

[dep_d22]

D22. Under den här tiden, talade du långsammare eller rörde dig långsammare än vanligt så att andra människor lade märke till det?

❍ Ja [1] ❍ Vet ej / vill ej svara [998]

❍ Nej [2]

[dep_d23]

D23. Människor ser ibland ner på sig själva, känner sig dåliga eller värdelösa. Kände du det så under den här tiden?

❍ Ja [1] ❍ Vet ej / vill ej svara [998]

❍ Nej [2]

[dep_d24]

D24.Tänkte du mycket på döden under den här tiden – antingen din egen eller någon annans död eller på döden överhuvudtaget?

❍ Ja [1]   ❍ Vet ej / vill ej svara [998]

❍ Nej [2]

- ❖ **E. OM D5 ELLER D10 ÄR "JA" ELLER D12 INTE ÄR "VET EJ / VILL EJ SVARA" ELLER D17 ÄR "VARJE NATT ELLER NÄSTAN VARJE NATT" ELLER [D20, D21, D22, D23 ELLER D24] ÄR "JA"; VISA NEDANSTÅENDE FRÅGOR**
- ❖ **F. OM D4 ÄR "VARJE DAG ELLER NÄSTAN VARJE DAG", VISA NEDANSTÅENDE FRÅGOR**

[dep_d25]

D25. Du nämnde tidigare att du hade en period på två veckor i sträck då du hade något av följande probem: {var trött och hade ingen energi}{hade problem med aptiten} {hade problem med vikten}{hade sömnproblem}{hade problem med koncentrationen}{var nervös eller rastlös}{talade långsammare eller rörde dig långsammare än vanligt}{hade problem med självförtroendet} {hade tankar om döden}.
I vilken utsträckning hindrade dessa symtom dig i ditt arbete, ditt sociala liv eller dina relationer med andra?

❍ Helt och hållet eller mycket [1]

❍ Något [2]

❍ Litet eller inte alls [3]

❍ Vet ej / vill ej svara [998]

[dep_d26]

D26. I vilken utsträckning var du bedrövad på grund av dessa symtom under den här tiden?

❍ Helt och hållet eller mycket [1]

❍ Något [2]

❍ Litet eller inte alls [3]

❍ Vet ej / vill ej svara [998]

[dep2_d27]

D27. Hur gammal var du första gången då du under två veckor i sträck var ledsen, nedstämd eller deprimerad och samtidigt {var trött och hade ingen energi}{hade problem med aptiten} {hade problem med vikten}{hade sömnproblem}{hade problem med koncentrationen}{var nervös eller rastlös}{talade långsammare eller rörde dig långsammare än vanligt}{hade problem med självförtroendet} {hade tankar om döden}?

❍ 9 år eller yngre [1]
❍ 10 till 14 år [2]
❍ 15 till 19 år [3]
❍ 20 till 24 år [4]
❍ 25 till 29 år [5]
❍ 30 till 34 år [6]
❍ 35 till 39 år [7]
❍ 40 år eller äldre [8]
❍ Vet ej / vill ej svara [998]

[dep2_d28]

D28. Hur gammal var du senaste gången du kände så här?

❍ 9 år eller yngre [1]
❍ 10 till 14 år [2]
❍ 15 till 19 år [3]
❍ 20 till 24 år [4]
❍ 25 till 29 år [5]
❍ 30 till 34 år [6]
❍ 35 till 39 år [7]
❍ 40 år eller äldre [8]
❍ Vet ej / vill ej svara [998]

- **F. VILLKORET AVSLUTAS**
- **G. OM D9 ÄR "VARJE DAG ELLER NÄSTAN VARJE DAG", VISA NEDANSTÅENDE FRÅGOR**

[dep2_d29]

D29. Hur gammal var du första gången du hade en period på två veckor i sträck då du förlorade intresset för det mesta och också andra saker som att du {var

trött och hade ingen energi}{hade problem med aptiten} {hade problem med vikten}{hade sömnproblem}{hade problem med koncentrationen}{var nervös eller rastlös}{talade långsammare eller rörde dig långsammare än vanligt}{hade problem med självförtroendet} {hade tankar om döden}?

❍ 9 år eller yngre [1] ❍ Vet ej / vill ej svara [998]
❍ 10 till 14 år [2]
❍ 15 till 19 år [3]
❍ 20 till 24 år [4]
❍ 25 till 29 år [5]
❍ 30 till 34 år [6]
❍ 35 till 39 år [7]
❍ 40 år eller äldre [8]

[dep2_d30]

D30. Hur gammal var du senaste gången du kände så här?

❍ 9 år eller yngre [1] ❍ Vet ej / vill ej svara [998]
❍ 10 till 14 år [2]
❍ 15 till 19 år [3]
❍ 20 till 24 år [4]
❍ 25 till 29 år [5]
❍ 30 till 34 år [6]
❍ 35 till 39 år [7]
❍ 40 år eller äldre [8]

- **G. VILLKORET AVSLUTAS**
- **H. OM D4 ÄR "VARJE DAG ELLER NÄSTAN VARJE DAG" ELLER D9 ÄR "VARJE DAG ELLER NÄSTAN VARJE DAG", VISA NEDANSTÅENDE FRÅGOR**

[dep2_d31]

D31. Hur många gånger har du känt så här under din livstid?

❍ 1 gång [1]

❍ 2 gånger [2]

❍ 3 till 4 gånger [3]

❍ 5 till 9 gånger [4]

❍ 10 gånger eller mer [5]

❍ Vet ej / vill ej svara [998]

[dep_d32]

D32. Har du någonsin talat med en läkare eller annan professionell person om din depression? (Med professionell person menas läkare, psykolog, rådgivare inom vården, akupunktör eller annan professionell person som arbetar inom en hälsobefrämjande verksamhet t.ex. en andlig rådgivare, örtmedicinare)

❍ Ja [1] ❍ Vet ej / vill ej svara [998]

❍ Nej [2]

Om D32 är "ja", visa D32a

[dep2_d32a]

D32a. Hur gammal var du första gången du talade med en professionell person om din depression?

❍ 9 år eller yngre [1] ❍ Vet ej / vill ej svara [998]
❍ 10 till 14 år [2]
❍ 15 till 19 år [3]
❍ 20 till 24 år [4]
❍ 25 till 29 år [5]
❍ 30 till 34 år [6]
❍ 35 till 39 år [7]
❍ 40 år eller äldre [8]

❖ **A, D, E, G OCH H. VILLKORET AVSLUTAS**

Perinatal depression

❖ **A. OM VILLKORET B I MODULEN SCREENING FÖR PERINATAL DEPRESSION ÄR SANT, VISA NEDANSTÅENDE FRÅGOR**

[dep_pn3]

PN30. Tidigare nämnde du att du känt dig ledsen, olycklig eller mycket orolig under graviditeten eller efter en förlossning. Tänk tillbaka på den värsta perioden under graviditeten eller efter förlossningen. Under den värsta perioden då du känt dig ledsen, olycklig, eller mycket orolig under graviditeten eller efter förlossningen, hur ofta:

	Ofta [1]	Ibland [2]	Sällan [3]	Aldrig [4]	Vet ej / vill ej svara [998]
Kände du att du kunde skratta eller se det roliga i en situation? [1]	☐	☐	☐	☐	☐
Kunde du se fram emot något med hög förväntan? [2]	☐	☐	☐	☐	☐
Klandrade du dig själv när något blev fel? [3]	☐	☐	☐	☐	☐
Var du ängslig eller orolig utan anledning? [4]	☐	☐	☐	☐	☐
Kände du rädsla eller panik utan anledning? [5]	☐	☐	☐	☐	☐
Kände du dig överhopad eller nertyngd? [6]	☐	☐	☐	☐	☐
Var du så olycklig att du hade svårt att sova? [7]	☐	☐	☐	☐	☐
Kände du dig ledsen eller olycklig? [8]	☐	☐	☐	☐	☐

	Ofta [1]	Ibland [2]	Sällan [3]	Aldrig [4]	Vet ej / vill ej svara [998]
Var du så olycklig att du grät? [9]	☐	☐	☐	☐	☐
Kom du på tanken att skada dig själv? [10]	☐	☐	☐	☐	☐

❖ **B. LÄMNA OM ALLA FRÅGORNA I PN30 ÄR "ALDRIG"**

[dep_pn4]

PN40. Under den värsta perioden då du kände dig ledsen, olycklig, eller mycket orolig under en graviditet eller efter en förlossning, orsakade symtomen problem för dig eller störde de ditt dagliga liv?

❍ Ja [1] ❍ Vet ej / vill ej svara [998]

❍ Nej [2]

[dep_pn5]

PN50. Under den värsta perioden, när började dessa symtom?

❍ Under graviditeten [1]

❍ Efter förlossningen [2]

❍ Vet ej / vill ej svara [998]

Om PN50 är "under graviditeten", visa PN51
Om PN50 är "efter förlossningen", visa PN52

[dep_pn5_1]

PN51. Hur långd tid efter att du blev gravid började problemen?

❍ 1 till 3 månader [1]

❍ 4 till 6 månader [2]

❍ 7 till 9 månader [3]

❍ Vet ej / vill ej svara [998]

[dep_pn5_2]

PN52. Hur lång tid efter förlossningen började problemen?

❍ 0 till 4 veckor [1]

❍ 1 till 3 månader [2]

❍ Mer än 3 månader efter förlossningen [3]

❍ Vet ej / vill ej svara [998]

[dep_pn6]

PN60. Under den värsta perioden, hur länge varade symtomen?

❍ Mindre än 2 veckor [1]

❍ 2 till 4 veckor [2]

❍ 1 månad eller längre [3]

❍ Vet ej / vill ej svara [998]

[dep_pn7]

PN70. Talade du någonsin med en läkare eller annan professionell person om att du kände dig ledsen, olycklig eller mycket orolig under graviditeten eller efter förlossningen? Med professionell person menas psykolog, medicinsk eller andlig rådgivare, örtmedicinare, akupunktör, eller annan vårdgivare.

❍ Ja [1]   ❍ Vet ej / vill ej svara [998]

❍ Nej [2]

Om PN70 är "ja", visa PN80

[dep2_pn8]

PN80. Hur gammal var du första gången du talade med en professionell person om din depression?

❍ 9 år eller yngre [1]
❍ 10 till 14 år [2]
❍ 15 till 19 år [3]
❍ 20 till 24 år [4]
❍ 25 till 29 år [5]
❍ 30 till 34 år [6]
❍ 35 till 39 år [7]
❍ 40 år eller äldre [8]
❍ Vet ej / vill ej svara [998]

❖ **A. VILLKORET AVSLUTAS**

Premenstruella symtom

❖ **A. OM VILLKORET B I SCREENINGMODULEN FÖR PREMENSTRUELLA SYMTOM ÄR SANT VISA NEDAN FRÅGOR**

[pms_4]

PMS40. Du nämnde tidigare att du har besvär som börjar före din menstruation och upphör helt och hållet ungefär en vecka efter att menstruationen börjar.

Vilket/vilka av följande symptom brukar du uppleva i den premenstruella perioden?

	Inte alls [1]	Måttligt [2]	Ganska mycket [3]	Mycket [4]
Ilska / irritabilitet [ang]	☐	☐	☐	☐
Oro / ångest / spänningskänsla / svårighet att koppla av [anx]	☐	☐	☐	☐
Ökad benägenhet att gråta / ökad känslighet för kritik [tea]	☐	☐	☐	☐
Nedstämdhet / känsla av hopplöshet [dep]	☐	☐	☐	☐
Minskat intresse för vardagliga ativiteter [act]	☐	☐	☐	☐

	Inte alls [1]	Måttligt [2]	Ganska mycket [3]	Mycket [4]
Koncentrationssvårigheter [con]	☐	☐	☐	☐
Trötthet / orkeslöshet / brist på energi [fat]	☐	☐	☐	☐
Ökad eller förändrad aptit / begär efter viss speciell mat [eat]	☐	☐	☐	☐
Förändrad sömn (ökad eller minskad) [sle]	☐	☐	☐	☐
Känsla av att inte ha kontroll över tillvaron [ove]	☐	☐	☐	☐
Bröstspänning / smärta eller svullnad i brösten [bre]	☐	☐	☐	☐
Svullnadskänsla i kroppen [blo]	☐	☐	☐	☐
Huvudvärk [hea]	☐	☐	☐	☐
Muskel- eller ledvärk [joi]	☐	☐	☐	☐
Viktuppgång [wei]	☐	☐	☐	☐

- **B. FÖR VARJE FRÅGA I PMS40 SOM ÄR BESVARAD ”MÅTTLIGT” ”GANSKA MYCKET”ELLER ”MYCKET” VISA NEDAN MOTSVARANDE FRÅGA**

[pms_5]

PMS50. Vilket/vilka av följande symtom påverkar din livskvalitet, t.ex. vad gäller relationer med andra, ditt sociala liv eller din förmåga till arbete och hemarbete? (Markera alla aktuella)

Ilska / irritabilitet [ang]	☐
Oro / ångest / spänningskänsla / svårighet att koppla av [anx]	☐
Ökad benägenhet att gråta / ökad känslighet för kritik [tea]	☐
Nedstämdhet / känsla av hopplöshet [dep]	☐
Minskat intresse för vardagliga ativiteter [act]	☐

Koncentrationssvårigheter [con]	☐
Trötthet / orkeslöshet / brist på energi [fat]	☐
Ökad eller förändrad aptit / begär efter viss speciell mat [eat]	☐
Förändrad sömn (ökad eller minskad) [sle]	☐
Känsla av att inte ha kontroll över tillvaron [ove]	☐
Bröstspänning / smärta eller svullnad i brösten [bre]	☐
Svullnadskänsla i kroppen [blo]	☐
Huvudvärk [hea]	☐

Muskel- eller ledvärk [joi]	☐
Viktuppgång [wei]	☐

❖ **A OCH B. VILLKOREN AVSLUTAS**

Paniksyndrom / panikångest

- **A. OM VILLKORET A I SCREENINGMODULEN FÖR PANIKÅNGEST ÄR SANT, VISA NEDANSTÅENDE FRÅGOR**

[pan_1a]

PD1. Intro2. Tidigare svarade du att du har haft attacker då du helt plötsligt besväras av flera problem som t.ex. andningssvårigheter, hjärtklappning eller att hjärtat rusat eller att du fick yrsel, och att du var rädd att du skulle dö eller att du höll på att bli galen. Tänk tillbaka på en sådan attack. Vilka av följande problem hade du under attacken?

	Ja [1]	Nej [2]	Vet ej / vill ej svara [998]
Slog hjärtat fort eller rusade? [1]	☐	☐	☐
Fick du andningssvårigheter? [2]	☐	☐	☐
Drabbades du av illamående eller ont i magen? [3]	☐	☐	☐
Kände du dig yr eller svimfärdig? [4]	☐	☐	☐
Svettades du? [5]	☐	☐	☐
Darrade du eller skakade? [6]	☐	☐	☐
Kändes det som om du höll på att kvävas? [7]	☐	☐	☐
[pan_1b] Fick du smärta eller besvär över bröstet? [8]	☐	☐	☐
Var du rädd att du skulle tappa kontrollen eller bli tokig? [9]	☐	☐	☐
Kändes det som om du eller din omgivning verkade overkliga? [10]	☐	☐	☐

	Ja [1]	Nej [2]	Vet ej / vill ej svara [998]
Var du rädd att du skulle dö? [11]	☐	☐	☐
Fick du varma vallningar eller kalla rysningar? [12]	☐	☐	☐
Drabbades du av domningar eller stickningar? [13]	☐	☐	☐

❖ **B. LÄMNA OM MINDRE ÄN 4 FRÅGOR I PD1 ÄR "JA"**

[pan_2_1]

PD2.1 Under dina attacker, brukade problem som {nyckelfraser i PD1} uppstå plötsligt?

❍ Ja [1] ❍ Vet ej / vill ej svara [998]

❍ Nej [2]

Om PD2.1 är "ja", visa PD2.2 och PD4

[pan_2_2]

PD2.2 Under dina attacker, brukade problem som att hjärtat slog fort eller rusade, andningssvårigheter eller rädsla för att förlora kontrollen nå sin kulmen inom 10 minuter efter det att attacken började?

❍ Ja [1] ❍ Vet ej / vill ej svara [998]

❍ Nej [2]

[pan_4]

PD4. Ungefär hur många sådana plötsliga attacker har du haft under hela din livstid?

❍ (Antal attacker) [nbr]____________ [nbr_other]

❍ Vet ej / vill ej svara [998]

[pan2_9]

PD9. Hur gammal var du allra första gången du hade en sådan attack?

❍ 9 år eller yngre [1]
❍ 10 till 14 år [2]
❍ 15 till 19 år [3]
❍ 20 till 24 år [4]
❍ 25 till 29 år [5]
❍ 30 till 34 år [6]
❍ 35 till 39 år [7]
❍ 40 år eller äldre [8]
❍ Vet ej / vill ej svara [998]

[pan2_10d]

PD10d. Hur gammal var du senaste gången du hade en sådan attack?

❍ 9 år eller yngre [1]
❍ 10 till 14 år [2]
❍ 15 till 19 år [3]
❍ 20 till 24 år [4]
❍ 25 till 29 år [5]
❍ 30 till 34 år [6]
❍ 35 till 39 år [7]
❍ 40 år eller äldre [8]
❍ Vet ej / vill ej svara [998]

[pan_13]

PD13. Efter en sådan attack, hände det att du hade någon av följande upplevelser under en månad eller mer:

	Ja [1]	Nej [2]	Vet ej / vill ej svara [998]
Du oroade dig ofta för att du skulle få en attack till eller att du oroade dig för att något hemskt skulle hända på grund av attackerna? [1]	☐	☐	☐
Du ändrade dina dagliga aktiviteter eller undvek vissa situationer för att du var rädd för att få en attack till?	☐	☐	☐

	Ja [1]	Nej [2]	Vet ej / vill ej svara [998]
[2]			

[pan_17]

PD17. Den här sortens attacker kan komma “från ingenstans”. Fick du någonsin en attack som kom helt oväntat “från ingenstans”?

❍ Ja [1] ❍ Vet ej / vill ej svara [998]

❍ Nej [2]

Om PD17 är “ja”, visa PD17a

[pan_17a]

PD17a. Ungefär hur många attacker under din livstid kom helt oväntat ”från ingenstans”?

❍ (Antal attacker) [nbr]____________ [nbr_othere]

❍ Vet ej / vill ej svara [998]

[pan_50]

PD50. Har du någonsin talat med en läkare eller annan professionell person om dina attacker? (Med professionell person menas psykolog, medicinsk eller andlig rådgivare, örtmedicinare, akupunktör, eller annan professionell person.)

❍ Ja [1] ❍ Vet ej / vill ej svara [998]

❍ Nej [2]

❖ **A. VILLKORET AVSLUTAS**

Agorafobi

❖ **A. OM VILLKORET B I SCREENINGMODULEN FÖR AGORAFOBI ÄR SANT, VISA NEDANSTÅENDE FRÅGOR**

[ago_1]

AG1. Följande frågor handlar om att man kan känna en stark rädsla i samband med stora folksamlingar, allmänna platser och att resa. Har du någonsin känt en stark rädsla för att vara i följande situationer?

	Ja [1]	Nej [2]	Vet ej / vill ej svara [998]
AG1a. Att vara ensam hemma? [1]	☐	☐	☐
AG1b. Att vara i stora folksamlingar? [2]	☐	☐	☐
AG1c. Att resa hemifrån? [3]	☐	☐	☐
AG1d. Att resa ensam eller att vara borta ensam från hemmet? [4]	☐	☐	☐
AG1e. Att åka kommunalt? [5]	☐	☐	☐
AG1f. Att köra bil? [6]	☐	☐	☐
AG1g. Att stå i kö på allmän plats? [7]	☐	☐	☐
AG1h. Att vistas i ett varuhus, shoppingcenter eller en livsmedelsaffär? [8]	☐	☐	☐
AG1i. Att vara på biograf, i ett auditorium, en föreläsningssal eller kyrka? [9]	☐	☐	☐
AG1j. Att vara på restaurang eller någon annan allmän plats? [10]	☐	☐	☐
AG1k. Att vara på ett brett, öppet fält eller gata? [11]	☐	☐	☐

❖ **B. LÄMNA OM MINDRE ÄN FYRA FRÅGOR I AG1 ÄR "JA"**

[ago_3i2]

AG3i2. Du svarade att du känner en stor rädsla i flera av de situationer som nämns på listan. Kommer du ihåg vid vilken ålder du första gången kände en stark rädsla i någon sådan situation?

❍ Ja [1] ❍ Vet ej / vill ej svara [998]

❍ Nej [2]

Om AG3i2 är "ja", visa AG3b1

[ago2_3b1]

AG3b1. Hur gammal var du?

❍ 9 år eller yngre [1] ❍ Vet ej / vill ej svara [998]
❍ 10 till 14 år [2]
❍ 15 till 19 år [3]
❍ 20 till 24 år [4]
❍ 25 till 29 år [5]
❍ 30 till 34 år [6]
❍ 35 till 39 år [7]
❍ 40 år eller äldre [8]

[ago_4]

AG4. Människor med sådana rädslor skiljer sig i vad det är de fruktar i situationen. Vilken av följande rädslor upplevde du:

	Ja [1]	Nej [2]	Vet ej / vill ej svara [998]
AG4a. Rädsla att vara ensam eller att vara separerad från nära och kära? [1]	☐	☐	☐
AG4b. Rädsla för att det föreligger en reell fara, t.ex. att du kunde bli rånad eller överfallen? [2]	☐	☐	☐
AG4c. Rädsla för att bli dålig i magen eller få	☐	☐	☐

	Ja [1]	Nej [2]	Vet ej / vill ej svara [998]
diarré? [3]			
AG4d. Rädsla för att få en panikattack? [4]	☐	☐	☐
AG4e. Rädsla för att få en hjärtattack eller något annat akut problem? [5]	☐	☐	☐
AG4f. Rädsla för att bli fysiskt sjuk och inte kunna få hjälp? [6]	☐	☐	☐
AG4g. Rädsla för att det skulle vara svårt eller pinsamt att fly? [7]	☐	☐	☐
AG4h. Rädsla för att något annat hemskt skulle hända? [8]	☐	☐	☐
AG4i. Rädsla för att det inte skulle finnas någon hjälp att få om du behövde det? [9]	☐	☐	☐

[ago_5]

AG5. Har det någonsin varit så att du nästan alltid blev mycket upprörd eller orolig närhelst du stod inför en sådan situation?

❍ Ja [1] ❍ Vet ej / vill ej svara [998]

❍ Nej [2]

[ago_6]

AG6. Har det någonsin varit så att du undvek en sådan situation närhelst du kunde på grund av din rädsla?

❍ Ja [1] ❍ Vet ej / vill ej svara [998]

❍ Nej [2]

[ago_8]

AG8. Var det en speciell situation eller en speciell händelse som orsakade din rädsla för sådana situationer första gången det hände?

❍ Ja [1]   ❍ Vet ej / vill ej svara [998]

❍ Nej [2]

Om AG8 är "ja", visa AG8a

[ago_8a]

AG8a. Resulterade situationen eller händelsen i en panikattack?

❍ Ja [1]   ❍ Vet ej / vill ej svara [998]

❍ Nej [2]

[ago_9]

AG9. Tänk på den tiden i ditt liv när din rädsla (och undvikande) var mest allvarlig och mest frekvent. När du hamnade i eller trodde du skulle hamna i dessa situationer, hade du någonsin någon av följande upplevelser?

	Ja [1]	Nej [2]	Vet ej / vill ej svara [998]
Slog ditt hjärta fort eller rusade? [1]	☐	☐	☐
Svettades du? [2]	☐	☐	☐
Darrade du eller skakade? [3]	☐	☐	☐
Blev du torr i munnen? [4]	☐	☐	☐

[ago_13a]

AG13. Nu en fråga om hur din rädsla (eller undvikande) kan ha påverkat ditt liv. Var det någonsin så att du inte kunde gå hemifrån en hel dag på grund av din rädsla?

❍ Ja [1]   ❍ Vet ej / vill ej svara [998]

❍ Nej [2]

Om AG13 är "ja", visa AG13a

[ago_13a]

AG13a. Vilken är den längsta perioden räknat i dagar, veckor, månader eller år då du inte kunde gå hemifrån?

❍ (Dagar) [dys]____________
[dys_other]

❍ (Veckor) [wks]____________
[wks_other]

❍ (Månader) [mnt]____________
[mnt_other]

❍ (År) [yrs]____________
[yrs_other]

❍ Vet ej / vill ej svara [998]

[ago_14]

AG14. En del människor kan inte gå hemifrån om de inte har någon bekant med sig t.ex. en familjemedlem eller vän. Har det varit så för dig någon gång?

❍ Ja [1]

❍ Nej [2]

❍ Vet ej / vill ej svara [998]

[ago_15]

AG15. I vilken utsträckning har din rädsla för (eller undvikande av) sådana situationer någonsin hindrat dig antingen i ditt arbete, ditt sociala liv eller dina personliga relationer?

❍ Inte alls [5]

❍ Litet [4]

❍ Något [3]

❍ Mycket [2]

❍ Extremt mycket [1]

❍ Vet ej / vill ej svara [998]

Om AG15 är “mycket” eller “extremt mycket”, visa AG15.1

[ago_15_1]

AG15.1. Hur ofta under den tiden var det omöjligt för dig att genomföra dina dagliga aktiviteter och ta hand om dig själv på grund av din rädsla för (eller undvikande av) dessa situationer?

❍ Ofta [1]

❍ Ibland [2]

❍ Inte så ofta [3]

❍ Aldrig [4]

❍ Vet ej / vill ej svara [998]

[ago2_17b]

AG17b. Hur gammal var du senaste gången du antingen var mycket rädd för eller undvek en sådan situation?

❍ 9 år eller yngre [1]
❍ 10 till 14 år [2]
❍ 15 till 19 år [3]
❍ 20 till 24 år [4]
❍ 25 till 29 år [5]
❍ 30 till 34 år [6]
❍ 35 till 39 år [7]
❍ 40 år eller äldre [8]
❍ Vet ej / vill ej svara [998]

[ago_ag24]

AG24. Har du någonsin i ditt liv talat med en läkare eller annan professionell person om din rädsla för (eller undvikande av) dessa situationer? (Med annan professionell person menas psykolog, medicinsk eller andlig rådgivare, örtmedicinare, akupunktör, eller annan vårdgivare.)

❍ Ja [1]

❍ Nej [2]

❍ Vet ej / vill ej svara [998]

Om AG24 är "ja", visa AG24a

[ago2_ag24a]

AG24a. Hur gammal var du första gången (du talade med en professionell person om din rädsla)?

❍ 9 år eller yngre [1]
❍ 10 till 14 år [2]
❍ 15 till 19 år [3]
❍ 20 till 24 år [4]
❍ 25 till 29 år [5]
❍ 30 till 34 år [6]
❍ 35 till 39 år [7]
❍ Vet ej / vill ej svara [998]

❍ 40 år eller äldre [8]

❖ **A. VILLKORET AVSLUTAS**

Tvångssyndrom (OCD)

- **A. OM VILLKORET A I SCREENINGMODULEN FÖR TVÅNGSSYNDROM ÄR SANT, VISA NEDANSTÅENDE FRÅGOR**

[ocd_o1]

O1. En del människor har upprepade obehagliga tankar, bilder, eller impulser som de inte kan bli av med. En del människor har t.ex. för sig att deras händer är smutsiga hur många gånger de än tvättar dem. Har du någonsin haft en period i ditt liv då du besvärades av något av följande:

	Ja [1]	Nej [2]	Vet ej / vill ej svara [998]
O1a. En återkommande, ihållande oro för smuts, bakterier eller smitta? [1]	☐	☐	☐
O1b. En återkommande, ihållande oro för att göra illa någon, eller att vara ansvarig för att saker blir fel? [2]	☐	☐	☐
O1c. En återkommande, ihållande oro om att saker ska vara symmetriska, ligga i rak linje, på exakt rätt sätt eller i rätt ordning, eller ett återkommande behov av att räkna eller att ta i saker? [3]	☐	☐	☐
O1d. En återkommande, ihållande oro över att behöva spara eller behålla saker, även om de har litet ekonomiskt eller sentimentalt värde? [4]	☐	☐	☐
O1e. Någon annan störande tanke som återkom, t.ex. en oro för att göra något hemskt eller omoraliskt, sexuella tankar som du fann störande eller obehagliga, eller någon annan återkommande obehaglig tanke, bild eller impuls? [5]	☐	☐	☐

- **B. OM MINST EN FRÅGA I O1 ÄR "JA", VISA NEDANSTÅENDE FRÅGOR**

[ocd_o3]

O3. Du nämner {nyckelfraser i O1}. Följande frågor fokuserar endast på dessa tankar, inte på något du kan ha gjort när tankarna uppstod. Ibland är sådana obehagliga tankar relaterade till vardagliga problem i områden som ekonomi, arbete, personliga relationer, eller att planera för framtiden. Hur ofta var dina obehagliga tankar koncentrerade på vardagliga problem.

❍ Hela tiden [1]
❍ För det mesta [2]
❍ Ibland [3]
❍ Sällan [4]
❍ Aldrig [5]
❍ Vet ej / vill ej svara [998]

Om O3 inte är "aldrig", visa O3a

[ocd_o3a]

O3a. Hur ofta fokuserade dina obehagliga tankar på annat än vardagliga problem?

❍ Hela tiden [1]
❍ För det mesta [2]
❍ Ibland [3]
❍ Sällan [4]
❍ Aldrig [5]
❍ Vet ej / vill ej svara [998]

[ocd_o5]

O5. I vilken utsträckning gjorde dessa obehagliga tankar att du blev ledsen eller känslomässigt upprörd?

❍ Extremt mycket [1]
❍ Mycket [2]
❍ En del [3]
❍ Litet [4]
❍ Inte alls [5]
❍ Vet ej / vill ej svara [998]

[ocd_o6]

O6. Under den period i ditt liv när du hade dessa obehagliga tankar, hur ofta tyckte du att de verkade överdrivna eller orimliga?

❍ Hela tiden [1] ❍ Sällan [4]

❍ För det mesta [2] ❍ Aldrig [5]

❍ Ibland [3] ❍ Vet ej / vill ej svara [998]

❖ **B1. OM O5 ÄR "EXTREMT MYCKET", "MYCKET" ELLER "EN DEL", VISA NEDANSTÅENDE FRÅGOR**

[ocd_o8a]

O8a. Hur ofta försökte du motstå eller få bort de obehagliga tankarna ur huvudet?

❍ Ofta [1] ❍ Aldrig [4]

❍ Ibland [2] ❍ Vet ej / vill ej svara [998]

❍ Sällan [3]

Om O8a är "sällan" eller "aldrig", visa O8b och O8c

[ocd_o8b]

O8b. Hur ofta försökte du ignorera dessa obehagliga tankar eller tänka på andra saker?

❍ Ofta [1] ❍ Aldrig [4]

❍ Ibland [2] ❍ Vet ej / vill ej svara [998]

❍ Sällan [3]

Om O8b är "sällan" eller "aldrig", visa O8c

[ocd_o8c]

O8c. En del människor reagerar på obehagliga tankar genom att tänka på något eller göra något om och om igen. En person som oroar sig för att lämna dörren olåst kan t.ex. kontrollera låset om och om igen. Eller en person som har en hemsk tanke kan be, räkna, eller repetera ord i huvudet om och om igen. Hur ofta reagerade du på dina obehagliga tankar genom att tänka något eller göra något om och om igen?

❍ Ofta [1]   ❍ Aldrig [4]

❍ Ibland [2]   ❍ Vet ej / vill ej svara [998]

❍ Sällan [3]

- **C. OM NÅGON AV FRÅGORNA I O8a, O8b ELLER O8c ÄR OFTARE ÄN "ALDRIG", VISA NEDANSTÅENDE FRÅGOR**

[ocd_o10]

O10. Hur ofta var dessa tankar så starka att du inte kunde få dem ur huvudet hur mycket du än försökte?

❍ Ofta [1]   ❍ Aldrig [4]

❍ Ibland [2]   ❍ Vet ej / vill ej svara [998]

❍ Sällan [3]

[ocd_o11]

O11. I vilken utsträckning hindrade dessa tankar dig någonsin i ditt arbete, sociala liv eller dina personliga relationer?

❍ Inte alls [5]   ❍ Mycket [2]

❍ Litet [4]   ❍ Extremt mycket [1]

❍ Något [3]   ❍ Vet ej / vill ej svara [998]

[ocd2_o12c]

O12c. Hur gammal var du sista gången du hade någon av dessa obehagliga tankar?

❍ 9 år eller yngre [1] ❍ Vet ej / vill ej svara [998]
❍ 10 till 14 år [2]
❍ 15 till 19 år [3]
❍ 20 till 24 år [4]
❍ 25 till 29 år [5]
❍ 30 till 34 år [6]
❍ 35 till 39 år [7]
❍ 40 år eller äldre [8]

[ocd_o16]

O16. Här följer några frågor om din upplevelse av dessa obehagliga tankar. Hur mycket av din tid upptogs av dessa tankar i genomsnitt?

❍ Mindre än 1 timme om dagen [1] ❍ 8 timmar eller mer om dagen [4]

❍ 1 till 3 timmar om dagen [2] ❍ Vet ej / vill ej svara [998]

❍ Mer än 3 upp till 8 timmar om dagen [3]

[ocd_o19]

O19. Hur mycket ansträngde du dig för att motstå dessa obehagliga tankar eller för att leda bort uppmärksamheten från dem när de uppstod i huvudet?

❍ Inte alls [1] ❍ Mycket [4]

❍ Litet [2] ❍ Extremt mycket [5]

❍ Något [3] ❍ Vet ej / vill ej svara [998]

[ocd_o20]

O20. Hur stor kontroll hade du över dessa obehagliga tankar?

❍ Ingen kontroll [1] ❍ Stor kontroll [4]

❍ Liten kontroll [2] ❍ Fullständig kontroll [5]

❍ Måttlig kontroll [3] ❍ Vet ej / vill ej svara [998]

❖ **B, B1 OCH C. VILLKOREN AVSLUTAS**

[ocd_o21]

O21. En del människor känner sig tvingade att utföra vissa handlingar om och om igen antingen fysiskt eller i huvudet. En del människor kontrollerar t.ex. spisen hemma om och om igen, många gånger om dagen, hur många gånger de än ser att spisen är avstängd. Hade du någonsin en period i ditt liv då du utförde någon av följande handlingar om och om igen:

	Ja [1]	Nej [2]	Vet ej / vill ej svara [998]
Tvättade dig, städade, eller sanerade upprepade gånger? [1]	☐	☐	☐
Kontrollerade saker som lås eller spis uppprepade gånger eller upprepade gånger såg till att inget hände eller skadade dig eller någon annan? [2]	☐	☐	☐
Rättade till, ställde i rad, ordnade saker, räknade, eller tog på saker, eller gjorde saker i en speciell ordning? [3]	☐	☐	☐
Alltid sparade saker, tills du inte kunde kasta saker som du inte längre behövde eller brydde dig om? [4]	☐	☐	☐
Några andra upprepade handlingar som du kände dig tvingad att göra, som att gå igenom ett moraliskt resonemang i huvudet om och om igen, eller att be om förlåtelse om och om igen, eller någon annan fysisk eller mental aktivitet som du kände att du måste göra upprepade gånger? [5]	☐	☐	☐

- ❖ **D. AVSLUTA MODULEN OM ALLA FRÅGORNA I O21 INTE ÄR "JA" OCH ALLA FRÅGORNA I O8 ÄR "ALDRIG" ELLER VET EJ / VILL EJ SVARA**
- ❖ **D1. OM INGEN AV FRÅGORNA I O21 ÄR "JA" OCH MINST EN FRÅGA I O8 ÄR OFTARE ÄN "ALDRIG", VISA EJ NEDANSTÅENDE FRÅGOR**

[ocd_o24]

O24. Följande frågor fokuserar bara på dessa upprepade handlingar och inte på de tankar vi frågade om tidigare. Under den period i ditt liv då du utförde dessa upprepade handlingar, hur ofta verkade någon av dem överdriven eller orimlig för dig?

❍ Hela tiden [1] ❍ Sällan [4]

❍ För det mesta [2] ❍ Aldrig [5]

❍ Ibland [3] ❍ Vet ej / vill ej svara [998]

Om O24 är "aldrig", visa O24a

[ocd_o24a]

O24a. Tyckte du någonsin att dessa handlingar var meningslösa eller onödiga, eller att du överdrev dem?

❍ Ja [1] ❍ Vet ej / vill ej svara [998]

❍ Nej [2]

- ❖ **D2. OM O24a INTE ÄR "JA" ELLER O24 ÄR OFTARE ÄN "ALDRIG", VISA NEDANSTÅENDE FRÅGOR**
- ❖ **D3. OM O24a INTE ÄR "JA" OCH MINST EN FRÅGA I O8 ÄR OFTARE ÄN "ALDRIG", VISA INTE NEDANSTÅENDE FRÅGOR**

[ocd_o26a]

O26a. Hur ofta fick dessa upprepade handlingar dig att känna dig mindre orolig eller ledsen?

❍ Ofta [1] ❍ Aldrig [4]

❍ Ibland [2] ❍ Vet ej / vill ej svara [998]

❍ Sällan [3]

Om O26a är "sällan" eller "aldrig", visa O26b

[ocd_o26b]

O26b. Hur ofta hindrade dessa upprepade handlingar dig från att bli orolig eller ledsen?

❍ Ofta [1] ❍ Aldrig [4]

❍ Ibland [2] ❍ Vet ej / vill ej svara [998]

❍ Sällan [3]

Om O26b är "sällan" eller "aldrig", visa O26c

[ocd_o26c]

O26c. Hur ofta kände du att något hemskt kanske skulle hända om du inte utförde dessa upprepade handlingar?

❍ Ofta [1] ❍ Aldrig [4]

❍ Ibland [2] ❍ Vet ej / vill ej svara [998]

❍ Sällan [3]

❖ **E. OM INGEN AV O26a, O26b OCH O26c ÄR "ALDRIG", VISA O28, O28a OCH O28b**

[ocd_o28]

O28. Hur ofta försökte du stoppa dig själv från att utföra dessa upprepade handlingar?

❍ Ofta [1] ❍ Aldrig [4]

❍ Ibland [2] ❍ Vet ej / vill ej svara [998]

❍ Sällan [3]

[ocd_o28a]

O28a. Hur ofta var tvånget att utföra dessa handlingar någonsin så starkt att du inte kunde motstå dem hur mycket du än försökte?

❍ Ofta [1] ❍ Aldrig [4]

❍ Ibland [2] ❍ Vet ej / vill ej svara [998]

❍ Sällan [3]

[ocd_o28b]

O28b. En del människor känner inte bara en lättnad när de utför dessa handlingar utan de finner dem behagliga eller njutbara. Hur ofta tyckte du att dessa upprepade handlingar var behagliga?

❍ Ofta [1] ❍ Aldrig [4]

❍ Ibland [2] ❍ Vet ej / vill ej svara [998]

❍ Sällan [3]

- **E. VILLKORET AVSLUTAS**
- **F. OM O28a ÄR OFTARE ÄN "ALDRIG", VISA NEDANSTÅENDE FRÅGOR**
- **G. LÄMNA OM FÖREGÅENDE I VILLKORET C ÄR FALSKT**

[ocd_o31]

O31. På en skala från "extremt mycket" till "inte alls", hur mycket har dessa upprepade handlingar gjort dig ledsen eller orsakat känslomässig upprördhet?

❍ Extremt mycket [1] ❍ Litet [4]

❍ Mycket [2] ❍ Inte alls [5]

❍ Något [3] ❍ Vet ej / vill ej svara [998]

[ocd_o32]

O32. Hur mycket har dessa upprepade handlingar hindrat dig i ditt arbete, ditt sociala liv eller personliga relationer?

❍ Inte alls [5] ❍ Mycket [2]

❍ Litet [4] ❍ Extremt mycket [1]

❍ Något [3] ❍ Vet ej / vill ej svara [998]

[ocd2_o33c]

O33c. Hur gammal var du sista gången du utförde några av dessa upprepade handlingar?

❍ 9 år eller yngre [1] ❍ Vet ej / vill ej svara [998]
❍ 10 till 14 år [2]
❍ 15 till 19 år [3]
❍ 20 till 24 år [4]
❍ 25 till 29 år [5]
❍ 30 till 34 år [6]
❍ 35 till 39 år [7]
❍ 40 år eller äldre [8]

[ocd_o37]

O37. Följande frågor gäller din upplevelse av dessa upprepade handlingar. Hur mycket tid per dag ägnade du i genomsnitt åt att utföra dessa handlingar?

❍ Mindre än 1 timme [1] ❍ Mer än 8 timmar [4]

❍ 1till 3 timmar [2] ❍ Vet ej / vill ej svara [998]

❍ Mer än 3 upp till 8 timmar [3]

❖ **D1, D3 OCH F. VILLKORET AVSLUTAS**

[ocd2_o60a]

O60a. Tänk på den första gången i livet då du upplevde de obehagliga tankarna eller de upprepade handlingarna. Hur gammal var du?

❍ 9 år eller yngre [1] ❍ Vet ej / vill ej svara [998]
❍ 10 till 14 år [2]
❍ 15 till 19 år [3]

❍ 20 till 24 år [4]
❍ 25 till 29 år [5]
❍ 30 till 34 år [6]
❍ 35 till 39 år [7]
❍ 40 år eller äldre [8]

Om O60a är "vet ej / vill ej svara", visa O60b

[ocd_o60b]

O60b. Ungefär i vilken ålder var du när du hade en sådan erfarenhet?

❍ Före skolstarten [1]

❍ Före tonåren [2]

❍ Före 20-årsåldern [3]

❍ Hela livet [4]

❍ Vet ej / vill ej svara [998]

[ocd_o69]

O69. Talade du någonsin i livet med en läkare eller annan professionell person om de obehagliga tankarna eller upprepade handlingarna? (Med professionell person menas psykolog, medicinsk eller andlig rådgivare, örtmedicinare, akupunktör eller annan vårdpersonal.)

❍ Ja [1]   ❍ Vet ej / vill ej svara [998]

❍ Nej [2]

Om O69 är "ja", visa O70

[ocd2_o70]

O70. Hur gammal var du första gången (du talade med en läkare eller annan professionell person om de obehagliga tankarna, handlingarna eller de upprepade handlingarna)?

❍ 9 år eller yngre [1]   ❍ Vet ej / vill ej svara [998]
❍ 10 till 14 år [2]

❍ 15 till 19 år [3]
❍ 20 till 24 år [4]
❍ 25 till 29 år [5]
❍ 30 till 34 år [6]
❍ 35 till 39 år [7]
❍ 40 år eller äldre [8]

❖ **A OCH D2. VILLKORET AVSLUTAS**

Posttraumatiskt stressyndrom

- ❖ **A. OM VILLKORET A I SCREENINGMODULEN FÖR POSTTRAUMATISKT STRESSYNDROM ÄR SANT, VISA NEDANSTÅENDE FRÅGOR**

[pts2]

PT3*. Har något av följande hänt dig någon gång under ditt liv?

❑ Bott på ett ställe där det pågått terror bland civilbefolkningen [4a]

❑ Varit en flykting [5a]

❑ Blivit kidnappad eller hållits fången [6a]

❑ Varit inblandad i en livshotande bilolycka [8a]

❑ Varit med om någon annan livshotande olycka, t.ex. en arbetsolycka [9a]

❑ Varit utsatt för en naturkatastrof, t.ex. en tsunami eller en stor översvämning [10a]

❑ Haft en livshotande sjukdom [12a]

❑ Som barn eller vuxen blivit allvarligt misshandlad [13a_15a]

❑ Blivit överfallen, rånad eller hotad med vapen [16a]

❑ Blivit utsatt för sexuellt övergrepp eller ofredande [17a_18a]

❑ Varit utsatt för att någon förföljt dig så att du känt allvarlig fara [19a]

❑ Haft en närstående som oväntat dött [20a]

❑ Haft ett barn med en livshotande sjukdom eller skada [21a]

❑ Som barn blivit vittne till allvarliga slagsmål hemma [22a]

❑ Haft en närstående som haft en extremt traumatisk upplevelse [22_1a]

❑ Sett någon bli svårt skadad, dödad eller oväntat sett en död kropp [23a]

❑

❍ Ingen av dessa [996]

❍ Vet ej / vill ej svara [998]

Om PT är "bott på ett ställe där det pågått terror bland civilbefolkningen", visa PT4b

[pts2_4b]

PT4b. Du nämnde att du bott på ett ställe där det pågått terror bland civilbefolkningen. Har detta hänt dig flera gånger?

❍ Ja [1] ❍ Vet ej / vill ej svara [998]

❍ Nej [2]

[pts3_4c]

PT4c. Hur gammal var du när detta hände {, första gången}?

❍ 9 år eller yngre [1] ❍ Vet ej / vill ej svara [998]
❍ 10 till 14 år [2]
❍ 15 till 19 år [3]
❍ 20 till 24 år [4]
❍ 25 till 29 år [5]
❍ 30 till 34 år [6]
❍ 35 till 39 år [7]
❍ 40 år eller äldre [8]

Om PT4b är "ja", visa PT4d

[pts2_4d]

PT4d. Hur många gånger har detta hänt?

❍ 2 [1]

❍ 3 [2]

❍ 4 [3]

❍ 5 eller fler [4]

Om PT är "varit en flykting"

[pts2_5b]

PT5b. Du nämnde att du varit en flykting – dvs. att du flytt från ditt land till ett främmande land eller en plats för att undkomma fara eller förföljelse. Har detta hänt dig flera gånger?

❍ Ja [1] ❍ Vet ej / vill ej svara [998]

❍ Nej [2]

[pts3_5c]

PT5c. Hur gammal var du när detta hände {, för första gången}?

❍ 9 år eller yngre [1] ❍ Vet ej / vill ej svara [998]
❍ 10 till 14 år [2]
❍ 15 till 19 år [3]
❍ 20 till 24 år [4]
❍ 25 till 29 år [5]
❍ 30 till 34 år [6]
❍ 35 till 39 år [7]
❍ 40 år eller äldre [8]

Om PT5b är "ja", visa PT5d

[pts2_5d]

PT5d. Hur många gånger har detta hänt?

❍ 2 [1]

❍ 3 [2]

❍ 4 [3]

❍ 5 eller fler [4]

Om PT är "blivit kidnappad eller hållits fången", visa PT6b

[pts2_6b]

PT6b. Du nämnde att du blivit kidnappad eller hållits fången. Har detta hänt dig flera gånger?

❍ Ja [1] ❍ Vet ej / vill ej svara [998]

❍ Nej [2]

[pts3_6c]

PT6c. Hur gammal var du när detta hände {, för första gången}?

❍ 9 år eller yngre [1]   ❍ Vet ej / vill ej svara [998]
❍ 10 till 14 år [2]
❍ 15 till 19 år [3]
❍ 20 till 24 år [4]
❍ 25 till 29 år [5]
❍ 30 till 34 år [6]
❍ 35 till 39 år [7]
❍ 40 år eller äldre [8]

Om PT6b är "ja", visa PT6d

[pts2_6d]

PT6d. Hur många gånger har detta hänt?

❍ 2 [1]

❍ 3 [2]

❍ 4 [3]

❍ 5 eller fler [4]

Om PT är "varit inblandad i en livshotande bilolycka", visa PT8b

[pts2_8b]

PT8b. Du nämnde att du varit inblandad i en livshotande bilolycka. Har detta hänt dig flera gånger?

❍ Ja [1]   ❍ Vet ej / vill ej svara [998]

❍ Nej [2]

[pts3_8c]

PT8c. Hur gammal var du när detta hände {, för första gången}?

❍ 9 år eller yngre [1] ❍ Vet ej / vill ej svara [998]
❍ 10 till 14 år [2]
❍ 15 till 19 år [3]
❍ 20 till 24 år [4]
❍ 25 till 29 år [5]
❍ 30 till 34 år [6]
❍ 35 till 39 år [7]
❍ 40 år eller äldre [8]

Om PT8b är "ja", visa PT8d

[pts2_8d]

PT8d. Hur många gånger har detta hänt?

❍ 2 [1]

❍ 3 [2]

❍ 4 [3]

❍ 5 eller fler [4]

Om PT är "varit inblandad i någon annan livshotande olycka, t.ex. en arbetsolycka", visa PT9b

[pts2_9b]

PT9b. Du nämnde att du varit med om någon annan livshotande olycka, t.ex. en arbetsolycka. Har detta hänt dig flera gånger?

❍ Ja [1] ❍ Vet ej / vill ej svara [998]

❍ Nej [2]

[pts3_9c]

PT9c. Hur gammal var du när detta hände {, för första gången}?

❍ 9 år eller yngre [1] ❍ Vet ej / vill ej svara [998]
❍ 10 till 14 år [2]

❍ 15 till 19 år [3]
❍ 20 till 24 år [4]
❍ 25 till 29 år [5]
❍ 30 till 34 år [6]
❍ 35 till 39 år [7]
❍ 40 år eller äldre [8]

Om PT9b är “ja”, visa PT9d

[pts2_9d]

PT9d. Hur många gånger har detta hänt?

❍ 2 [1]

❍ 3 [2]

❍ 4 [3]

❍ 5 eller fler [4]

Om PT är “varit utsatt för en naturkatastrof, t.ex. en tsunami eller en stor översvämning”, visa PT10b

[pts2_10b]

PT10b. Du nämnde att du varit utsatt för en naturkatastrof, t.ex. en tsunami eller en stor översvämning. Har detta hänt dig flera gånger?

❍ Ja [1]   ❍ Vet ej / vill ej svara [998]

❍ Nej [2]

[pts3_10c]

PT10c. Hur gammal var du när detta hände {, för första gången}?

❍ 9 år eller yngre [1]   ❍ Vet ej / vill ej svara [998]
❍ 10 till 14 år [2]
❍ 15 till 19 år [3]
❍ 20 till 24 år [4]
❍ 25 till 29 år [5]

❍ 30 till 34 år [6]
❍ 35 till 39 år [7]
❍ 40 år eller äldre [8]

Om PT10b är "ja", visa PT10d

[pts2_10d]

PT10d. Hur många gånger har detta hänt?

❍ 2 [1]

❍ 3 [2]

❍ 4 [3]

❍ 5 eller fler [4]

Om PT är "haft en livshotande sjukdom", visa PT12b

[pts2_12b]

PT12b. Du nämnde att du haft en livshotande sjukdom. Har detta hänt dig flera gånger?

❍ Ja [1] ❍ Vet ej / vill ej svara [998]

❍ Nej [2]

[pts3_12c]

PT12c. Hur gammal var du när detta hände {, för första gången}?

❍ 9 år eller yngre [1] ❍ Vet ej / vill ej svara [998]
❍ 10 till 14 år [2]
❍ 15 till 19 år [3]
❍ 20 till 24 år [4]
❍ 25 till 29 år [5]
❍ 30 till 34 år [6]
❍ 35 till 39 år [7]
❍ 40 år eller äldre [8]

Om PT12b är "ja", visa PT12d

[pts2_12d]

PT12d. Hur många gånger har detta hänt?

❍ 2 [1]

❍ 3 [2]

❍ 4 [3]

❍ 5 eller fler [4]

Om PT är "som barn eller vuxen blivit svårt misshandlad", visa PT13a

[pts2_13a]

PT13a. Följande frågor handlar om misshandel. Har du blivit allvarligt misshandlad som barn av dina föräldrar eller vårdnadshavare?

❍ Ja [1] ❍ Vet ej / vill ej svara [998]

❍ Nej [2]

Om PT13a är "ja", visa PT13b

[pts2_13b]

PT13b. Har detta hänt dig flera gånger?

❍ Ja [1] ❍ Vet ej / vill ej svara [998]

❍ Nej [2]

[pts3_13c]

PT13c. Hur gammal var du när detta hände {, för första gången}?

❍ 9 år eller yngre [1] ❍ Vet ej / vill ej svara [998]
❍ 10 till 14 år [2]
❍ 15 till 19 år [3]
❍ 20 till 24 år [4]
❍ 25 till 29 år [5]
❍ 30 till 34 år [6]
❍ 35 till 39 år [7]
❍ 40 år eller äldre [8]

Om PT13b är “ja”, visa PT13d

[pts2_13d]

PT13d. Hur många gånger har detta hänt?

❍ 2 [1]

❍ 3 [2]

❍ 4 [3]

❍ 5 eller fler [4]

[pts2_14a]

PT14a. Har du blivit allvarligt misshandlad av en make/maka eller partner?

❍ Ja [1] ❍ Vet ej / vill ej svara [998]

❍ Nej [2]

Om PT14a är “ja”, visa PT14b

[pts2_14b]

PT14b. Har detta hänt dig flera gånger?

❍ Ja [1] ❍ Vet ej / vill ej svara [998]

❍ Nej [2]

[pts3_14c]

PT14c. Hur gammal var du när detta hände {, för första gången}?

❍ 9 år eller yngre [1]   ❍ Vet ej / vill ej svara [998]
❍ 10 till 14 år [2]
❍ 15 till 19 år [3]
❍ 20 till 24 år [4]
❍ 25 till 29 år [5]
❍ 30 till 34 år [6]
❍ 35 till 39 år [7]
❍ 40 år eller äldre [8]

Om PT14b är "ja", visa PT14d

[pts2_14d]

PT14d. Hur många gånger har detta hänt?

❍ 2 [1]

❍ 3 [2]

❍ 4 [3]

❍ 5 eller fler [4]

[pts2_16a]

PT15a. Har du blivit allvarligt misshandlad av någon annan?

❍ Ja [1]   ❍ Vet ej / vill ej svara [998]

❍ Nej [2]

Om PT15a är "ja", visa PT15b

[pts2_15b]

PT15b. Har detta hänt dig flera gånger?

❍ Ja [1] ❍ Vet ej / vill ej svara [998]

❍ Nej [2]

[pts3_15c]

PT15c. Hur gammal var du när detta hände {, för första gången}?

❍ 9 år eller yngre [1] ❍ Vet ej / vill ej svara [998]
❍ 10 till 14 år [2]
❍ 15 till 19 år [3]
❍ 20 till 24 år [4]
❍ 25 till 29 år [5]
❍ 30 till 34 år [6]
❍ 35 till 39 år [7]
❍ 40 år eller äldre [8]

Om PT15b är "ja", visa PT15d

[pts2_15d]

PT15d. Hur många gånger har detta hänt?

❍ 2 [1]

❍ 3 [2]

❍ 4 [3]

❍ 5 eller fler [4]

Om PT är "blivit överfallen, rånad eller hotad med vapen", visa PT16b

[pts2_16b]

PT16b. Du nämnde att du blivit överfallen, rånad eller hotad med vapen. Har detta hänt dig flera gånger?

❍ Ja [1] ❍ Vet ej / vill ej svara [998]

❍ Nej [2]

[pts3_16c]

PT16c. Hur gammal var du när detta hände {, för första gången}?

❍ 9 år eller yngre [1] ❍ Vet ej / vill ej svara [998]
❍ 10 till 14 år [2]
❍ 15 till 19 år [3]
❍ 20 till 24 år [4]
❍ 25 till 29 år [5]
❍ 30 till 34 år [6]
❍ 35 till 39 år [7]
❍ 40 år eller äldre [8]

Om PT16b är "ja", visa PT16d

[pts2_16d]

PT16d. Hur många gånger har detta hänt?

❍ 2 [1]

❍ 3 [2]

❍ 4 [3]

❍ 5 eller fler [4]

Om PT är "blivit utsatt för sexuellt övergrepp eller ofredande", visa PT17a

[pts2_17a]

PT17a. Följande frågorna handlar om sexuella övergrepp. Den första handlar om våldtäkt. Våldtäkt definierar vi som att någon haft samlag med dig mot din vilja eller att någon penetrerat din kropp med ett finger eller föremål mot din vilja eller hotat dig genom att använda våld, eller när du var så ung att du inte förstod vad som hände. Har det någonsin hänt dig?

❍ Ja [1] ❍ Vet ej / vill ej svara [998]

❍ Nej [2]

Om PT17a är "ja", visa PT17b

[pts2_17b]

PT17b. Har detta hänt dig flera gånger?

❍ Ja [1] ❍ Vet ej / vill ej svara [998]

❍ Nej [2]

[pts3_17c]

PT17c. Hur gammal var du när detta hände {, för första gången}?

❍ 9 år eller yngre [1] ❍ Vet ej / vill ej svara [998]
❍ 10 till 14 år [2]
❍ 15 till 19 år [3]
❍ 20 till 24 år [4]
❍ 25 till 29 år [5]
❍ 30 till 34 år [6]
❍ 35 till 39 år [7]
❍ 40 år eller äldre [8]

Om PT17b är "ja", visa PT17d

[pts2_17d]

PT17d. Hur många gånger har detta hänt?

❍ 2 [1]

❍ 3 [2]

❍ 4 [3]

❍ 5 eller fler [4]

[pts2_18a]

PT18a. Förutom våldtäkt, har du någonsin varit utsatt för sexuellt övergrepp eller sexuellt ofredande?

❍ Ja [1] ❍ Vet ej / vill ej svara [998]

❍ Nej [2]

Om PT18a är "ja", visa PT18b

[pts2_18b]

PT18b. Har detta hänt dig flera gånger?

❍ Ja [1] ❍ Vet ej / vill ej svara [998]

❍ Nej [2]

[pts3_18c]

PT18c. Hur gammal var du när detta hände {, för första gången}?

❍ 9 år eller yngre [1] ❍ Vet ej / vill ej svara [998]
❍ 10 till 14 år [2]
❍ 15 till 19 år [3]
❍ 20 till 24 år [4]
❍ 25 till 29 år [5]
❍ 30 till 34 år [6]
❍ 35 till 39 år [7]
❍ 40 år eller äldre [8]

Om PT18b är "ja", visa PT18d

[pts2_18d]

PT18d. Hur många gånger har detta hänt?

❍ 2 [1]

❍ 3 [2]

❍ 4 [3]

❍ 5 eller fler [4]

Om PT är "varit utsatt för förföljelse så att du känt allvarlig fara", visa PT19b

[pts2_19b]

PT19b. Du nämnde att du varit utsatt för att någon förföljt dig och kontrollerat dina aktiviteter på ett sätt som fått dig att känna att du var i allvarlig fara. Har detta hänt dig flera gånger?

❍ Ja [1] ❍ Vet ej / vill ej svara [998]

❍ Nej [2]

[pts3_19c]

PT19c. Hur gammal var du när detta hände {, för första gången}?

❍ 9 år eller yngre [1] ❍ Vet ej / vill ej svara [998]
❍ 10 till 14 år [2]
❍ 15 till 19 år [3]
❍ 20 till 24 år [4]
❍ 25 till 29 år [5]
❍ 30 till 34 år [6]
❍ 35 till 39 år [7]
❍ 40 år eller äldre [8]

Om PT19b är "ja", visa PT19d

[pts2_19d]

PT19d. Hur många gånger har detta hänt?

❍ 2 [1]

❍ 3 [2]

❍ 4 [3]

❍ 5 eller fler [4]

Om PT är "haft någon närstående som oväntat dött", visa PT20b

[pts2_20b]

PT20b. Du nämnde att du haft någon närstående som oväntat dött. Har detta hänt dig flera gånger?

❍ Ja [1] ❍ Vet ej / vill ej svara [998]

❍ Nej [2]

[pts3_20c]

PT20c. Hur gammal var du när detta hände {, för första gången}?

❍ 9 år eller yngre [1] ❍ Vet ej / vill ej svara [998]
❍ 10 till 14 år [2]
❍ 15 till 19 år [3]
❍ 20 till 24 år [4]
❍ 25 till 29 år [5]
❍ 30 till 34 år [6]
❍ 35 till 39 år [7]
❍ 40 år eller äldre [8]

Om PT20b är "ja", visa PT20d

[pts2_20d]

PT20d. Hur många gånger har detta hänt?

❍ 2 [1]

❍ 3 [2]

❍ 4 [3]

❍ 5 eller fler [4]

Om PT är "haft ett barn med en livshotande sjukdom eller skada", visa PT21b

[pts2_21b]

PT21b. Du nämnde att du haft ett barn med en livshotande sjukdom eller skada. Har detta hänt dig flera gånger?

❍ Ja [1] ❍ Vet ej / vill ej svara [998]

❍ Nej [2]

[pts3_21c]

PT21c. Hur gammal var du när detta hände {, för första gången}?

❍ 9 år eller yngre [1] ❍ Vet ej / vill ej svara [998]
❍ 10 till 14 år [2]
❍ 15 till 19 år [3]
❍ 20 till 24 år [4]
❍ 25 till 29 år [5]
❍ 30 till 34 år [6]
❍ 35 till 39 år [7]
❍ 40 år eller äldre [8]

Om PT21b är "ja", visa PT21d

[pts2_21d]

PT21d. Hur många gånger har detta hänt?

❍ 2 [1]

❍ 3 [2]

❍ 4 [3]

❍ 5 eller fler [4]

Om PT är "som barn blivit vittne till allvarliga slagsmål hemma", visa PT22b

[pts2_22b]

PT22b. Du nämnde att du som barn blivit vittne till allvarliga slagsmål hemma. Har detta hänt dig flera gånger?

❍ Ja [1] ❍ Vet ej / vill ej svara [998]

❍ Nej [2]

[pts3_22c]

PT22c. Hur gammal var du när detta hände {, för första gången}?

❍ 9 år eller yngre [1]   ❍ Vet ej / vill ej svara [998]
❍ 10 till 14 år [2]
❍ 15 till 19 år [3]
❍ 20 till 24 år [4]
❍ 25 till 29 år [5]
❍ 30 till 34 år [6]
❍ 35 till 39 år [7]
❍ 40 år eller äldre [8]

Om PT22b är "ja", visa PT22d

[pts2_22d]

PT22d. Hur många gånger har detta hänt?

❍ 2 [1]

❍ 3 [2]

❍ 4 [3]

❍ 5 eller fler [4]

Om PT är "haft en närstående som haft en extremt traumatisk upplevelse", visa PT22.1b

[pts2_22_1b]

PT22.1b. Du nämnde att du haft en närstående som haft en extremt traumatisk upplevelse. Har detta hänt dig flera gånger?

❍ Ja [1]   ❍ Vet ej / vill ej svara [998]

❍ Nej [2]

[pts3_22_1c]

PT22.1c. Hur gammal var du när detta hände {, för första gången}?

❍ 9 år eller yngre [1]   ❍ Vet ej / vill ej svara [998]
❍ 10 till 14 år [2]
❍ 15 till 19 år [3]
❍ 20 till 24 år [4]
❍ 25 till 29 år [5]
❍ 30 till 34 år [6]
❍ 35 till 39 år [7]
❍ 40 år eller äldre [8]

Om PT22.1b är "ja", visa PT22.1d

[pts2_22_1d]

PT22.1d. Hur många gånger har detta hänt?

❍ 2 [1]

❍ 3 [2]

❍ 4 [3]

❍ 5 eller fler [4]

Om PT är "sett någon bli svårt skadad, dödad eller oväntat sett en död kropp", visa PT23b

[pts2_23b]

PT23b. Du nämnde att du sett någon bli svårt skadad, dödad eller att du oväntat sett en död kropp. Har detta hänt dig flera gånger?

❍ Ja [1]   ❍ Vet ej / vill ej svara [998]

❍ Nej [2]

[pts3_23c]

PT23c. Hur gammal var du när detta hände {, för första gången}?

❍ 9 år eller yngre [1]   ❍ Vet ej / vill ej svara [998]

❍ 10 till 14 år [2]
❍ 15 till 19 år [3]
❍ 20 till 24 år [4]
❍ 25 till 29 år [5]
❍ 30 till 34 år [6]
❍ 35 till 39 år [7]
❍ 40 år eller äldre [8]

Om PT23b är "ja", visa PT23d

[pts2_23d]

PT23d. Hur många gånger har detta hänt?

❍ 2 [1]

❍ 3 [2]

❍ 4 [3]

❍ 5 eller fler [4]

❖ **C. OM NÅGRA FRÅGOR I SPANNET PT4 TILL PT23 ÄR "JA", VISA NEDANSTÅENDE FRÅGOR**

[pts_62]

PT62. Låt oss sammanfatta. Du har varit med om olika typer av händelser, {dvs., exempelvis}: {nyckelfraser från PT4 till PT23}.

Efter {en sådan händelse, sådana händelser}, har människor ibland problem med t.ex. hemska minnen eller drömmar, att känna en känslomässig distans till andra människor, problem med sömnen och koncentrationen, och att känna sig nervös eller lättskrämd.

Hade du något av dessa problem efter någon av de traumatiska händelserna som du har gått igenom?

❍ Ja [1] ❍ Vet ej / vill ej svara [998]

❍ Nej [2]

❖ **D. LÄMNA OM PT62 ÄR "NEJ".**

Om PT62 är "ja", visa PT62.2

[pts_62_2]

PT62.2. Har du någonsin i ditt liv talat med en läkare eller annan professionell person om (problemet/något av dessa problem)? (Med en professionell person menas psykolog, medicinsk eller andlig rådgivare, örtmedicinare, akupunktör eller annan vårdgivare.)

❍ Ja [1] ❍ Vet ej / vill ej svara [998]

❍ Nej [2]

Om PT62.2 är "ja", visa PT62.2a

[pts2_62_2a]

PT62.2a Hur gammal var du första gången du talade med en professionell person om problemet eller något av dessa problem?

❍ 9 år eller yngre [1] ❍ Vet ej / vill ej svara [998]
❍ 10 till 14 år [2]
❍ 15 till 19 år [3]
❍ 20 till 24 år [4]
❍ 25 till 29 år [5]
❍ 30 till 34 år [6]
❍ 35 till 39 år [7]
❍ 40 år eller äldre [8]

[pts_66_1]

PT66.1 Du nämnde att du har haft följande trauman {, t.ex.}: {nyckelfraser från PT4 till PT23}.

[pts_67]

PT67. Vid tiden för de trauma(n) som är angivna ovan:

	Ja [1]	Nej [2]	Vet ej / vill ej svara [998]
PT67a1. Var du skräckslagen eller mycket rädd? [a1]	☐	☐	☐
PT67a. Kände du dig hjälplös? [a]	☐	☐	☐
PT67b. Kände du dig chockad eller förskräckt? [b]	☐	☐	☐
PT67c. Kände du dig förlamad? [c]	☐	☐	☐

[pts_68_86]

	Ja [1]	Nej [2]	Vet ej / vill ej svara [998]
PT68. Under veckorna, månaderna, eller åren efter den traumatiska händelsen (händelserna), tidigare eller för närvarande, försöker/försökte du att inte tänka på vad som hänt? [68]	☐	☐	☐
PT69. Höll du dig undan från platser, människor eller aktiviteter som påminde dig om den traumatiska händelsen (händelserna)? [69]	☐	☐	☐
PT70. Var det någonsin så att du inte kunde komma ihåg några viktiga delar av vad som hände? [70]	☐	☐	☐
PT71. Förlorade du intresset för att göra saker som du tyckte om tidigare? [71]	☐	☐	☐
PT72. Kände du dig känslomässigt distanserad eller avskärmad från andra människor? [72]	☐	☐	☐
PT73. Hade du svårt att känna normala känslor som kärlek, lycka, eller värme inför andra människor? [73]	☐	☐	☐
PT74. Kände du att du inte hade någon anledning att planera för framtiden därför att du trodde att den skulle bli förkortad? [74]	☐	☐	☐
PT86. Hade du någonsin upprepade, oönskade minnen eller inkräktande tankar på den traumatiska händelsen	☐	☐	☐

	Ja [1]	Nej [2]	Vet ej / vill ej svara [998]
(händelserna) – d.v.s. du påmindes om den även när du inte ville det? [86]			

[pts_87_106]

	Ja [1]	Nej [2]	Vet ej / vill ej svara [998]
PT87. Hade du någonsin upprepade, obehagliga drömmar om den traumatiska händelsen (händelserna)? [87]	☐	☐	☐
PT88. Hade du s.k. “flashbacks” – d.v.s. att du plötsligt agerade eller kände som om den traumatiska händelsen (händelserna) hände igen? [88]	☐	☐	☐
PT89. Blev du mycket olycklig när du påmindes om den traumatiska händelsen (händelserna)? [89]	☐	☐	☐
PT90. När du påmindes om den traumatiska händelsen (händelserna), hände det att du hade fysiska reaktioner såsom att svettas, att hjärtat rusade eller att du skakade? [90]	☐	☐	☐
PT102. Sedan tiden för den traumatiska händelsen (händelserna) har du problem med att somna eller fortsätta sova? [102]	☐	☐	☐
PT103. Har du varit mer irriterad eller haft kortare stubin än du brukar? [103]	☐	☐	☐
PT104. Har du haft mer problem med att koncentrera dig eller att hålla tankarna på vad du gör? [104]	☐	☐	☐
PT105. Har du varit mer alert eller vaksam, även när det inte fanns någon verklig anledning? [105]	☐	☐	☐
PT106. Har du varit nervös eller lättskrämd av vanliga ljud? [106]	☐	☐	☐

[pts_114]

PT114. Hur olycklig kände du dig över dessa reaktioner?

❍ Inte alls [1] ❍ Mycket [4]

❍ Litet [2] ❍ Väldigt mycket [5]

❍ Något [3] ❍ Vet ej / vill ej svara [998]

[pts_115]

PT115. I vilken utsträckning störde eller hindrade problemet eller problemen dig i ditt normala dagliga liv?

❍ Inte alls [1] ❍ Mycket [4]

❍ Litet [2] ❍ Väldigt mycket [5]

❍ Något [3] ❍ Vet ej / vill ej svara [998]

- **A OCH C. VILLKOREN AVSLUTAS**

Social fobi

- **A. OM VILLKORET A I SCREENINGMODULEN FÖR SOCIAL FOBI ÄR SANT, VISA NEDANSTÅENDE FRÅGOR**

[soi_so1]

SO1.Tidigare har du nämnt att du har en stark rädsla för vissa sociala situationer eller när du ska prestera något. Har du någonsin haft en stark rädsla för någon av följande situationer:

	Ja [1]	Nej [2]	Vet ej / vill ej svara [998]
SO1a. Att träffa nya människor? [1]	☐	☐	☐
SO1b. Att tala med människor i auktoritär ställning? [2]	☐	☐	☐
SO1c. Att tala i ett möte eller i en klass? [3]	☐	☐	☐
SO1d. Att gå på fest eller annan social sammankomst? [4]	☐	☐	☐
SO1e. Agera, uppträda eller hålla tal inför publik? [5]	☐	☐	☐
SO1f. Att göra ett viktigt prov eller gå på arbetsintervju, även om du är väl förberedd? [6]	☐	☐	☐
SO1g. Att arbeta medan någon tittar på? [7]	☐	☐	☐
SO1h. Att gå in i ett rum när andra redan är närvarande? [8]	☐	☐	☐

[soi_so1a]

SO1*. Har det någonsin funnits en period i ditt liv då du kände dig blyg, rädd eller obekväm i följande situationer:

	Ja [1]	Nej [2]	Vet ej / vill ej svara [998]
SO1i. Att tala med människor som du inte känner så väl? [1]	☐	☐	☐
SO1j. Att uttrycka en avvikande ståndpunkt för människor som du inte känner så väl? [2]	☐	☐	☐
SO1k. Att skriva, äta eller dricka när någon tittar på? [3]	☐	☐	☐
SO1l. Att kissa på offentlig toalett eller att använda någon annans toalett? [4]	☐	☐	☐
SO1m. Att gå på dejt? [5]	☐	☐	☐
SO1n. Någon annan social situation eller där du förväntas prestera något eller där du kunde hamna i centrum för uppmärksamheten eller där något pinsamt skulle kunna hända? [6]	☐	☐	☐

❖ **B. LÄMNA OM MINDRE ÄN FYRA FRÅGOR I SO1 ÄR "JA"**

[soi2_3i1]

SO3. Du nämnde att du har en rädsla för {nyckelfraser i O1}. Hur gammal var du första gången du upplevde rädsla för denna eller någon av dessa situationer?

❍ 9 år eller yngre [1]   ❍ Vet ej / vill ej svara [998]
❍ 10 till 14 år [2]
❍ 15 till 19 år [3]
❍ 20 till 24 år [4]
❍ 25 till 29 år [5]
❍ 30 till 34 år [6]
❍ 35 till 39 år [7]
❍ 40 år eller äldre [8]

Om SO3 är "vet ej / vill ej svara", visa SO3b

[soi_3b1]

SO3b. Ungerfär i vilken ålder var du?

❍ Före skolstarten [1]

❍ Före tonåren [2]

❍ Inte före tonåren [3]

❍ Vet ej / vill ej svara [998]

[soi_4]

SO4. Tycker du att rädslan någonsin var överdriven eller orimlig, eller mycket starkare än den borde ha varit?

❍ Ja [1]   ❍ Vet ej / vill ej svara [998]

❍ Nej [2]

❖ **C. AVSLUTA MODUL OM SO4 ÄR "NEJ"**

[soi_5]

SO5. Har det någonsin funnits en period då du nästan alltid blev mycket olycklig eller orolig när du stod inför någon sådan social situation eller när du förväntas prestera något?

❍ Ja [1]   ❍ Vet ej / vill ej svara [998]

❍ Nej [2]

[soi_6]

S06. Har du undvikit situationen eller dessa situationer närhelst du kunde på grund av din rädsla?

❍ Ja [1]   ❍ Vet ej / vill ej svara [998]

❍ Nej [2]

[soi_16]

SO16. I vilken utsträckning har din rädsla (eller ditt undvikande) någonsin hindrat dig i ditt arbete, sociala liv eller dina personliga relationer?

❍ Inte alls [5] ❍ Mycket [2]

❍ Litet [4] ❍ Extremt mycket [1]

❍ Något [3] ❍ Vet ej / vill ej svara [998]

Om SO16 är "något", "mycket" eller "extremt mycket", visa SO16.1

[soi_16a]

SO16a. Hur ofta under den perioden kunde du inte utföra dina dagliga aktiviteter eller ta hand om dig själv på grund av din rädsla (eller undvikande)?

❍ Ofta [1] ❍ Aldrig [4]

❍ Ibland [2] ❍ Vet ej / vill ej svara [998]

❍ Inte så ofta [3]

[soi2_18a]

SO18a. Hur gammal var du senaste gången du antingen hade en stark rädsla eller undvek situationen eller någon av dessa situationer?

❍ 9 år eller yngre [1] ❍ Vet ej / vill ej svara [998]
❍ 10 till 14 år [2]
❍ 15 till 19 år [3]
❍ 20 till 24 år [4]
❍ 25 till 29 år [5]
❍ 30 till 34 år [6]
❍ 35 till 39 år [7]
❍ 40 år eller äldre [8]

[soi_so25]

SO25. Har du någonsin i ditt liv talat med en läkare eller annan professionell person om din rädsla för (eller ditt undvikande av) { situationen, dessa

situationer}? (Med annan professionell person menas psykolog, medicinsk eller andlig rådgivare, örtmedicinare, akupunktör eller annan vårdpersonal.)

❍ Ja [1] ❍ Vet ej / vill ej svara [998]

❍ Nej [2]

Om SO25 är "ja", visa SO25a

[soi2_so25a]

SO25a. Hur gammal var du första gången (du talade med en professionell person om din rädsla)?

❍ 9 år eller yngre [1] ❍ Vet ej / vill ej svara [998]
❍ 10 till 14 år [2]
❍ 15 till 19 år [3]
❍ 20 till 24 år [4]
❍ 25 till 29 år [5]
❍ 30 till 34 år [6]
❍ 35 till 39 år [7]
❍ 40 år eller äldre [8]

❖ **A. VILLKORET AVSLUTAS**

Barn och förälder

Kompilerad enkät

ENKÄTEN BESVARAS DELS AV FÖRÄLDRAR OM SINA BARN SOM ÄR FRÅN 0 ÅR SAMT AV BARN FRÅN 11 ÅR

Innehåll

Innehåll 1

Sociodemografi 5

Hushållets sammansättning 5

Livsstil 7

Hygien 7

Solning 9

Matvanor barn 0-17 år 11

Intro 11

Amning 11

Livsmedel 14

Maträtter 20

Övriga frågor 26

Kosttillskott 33

Fysisk aktivitet 35

Allmän aktivitet 35

Transport till skola / dagis / sysselsättning 37

Fritidsaktiviteter ... 39

Skolidrott ... 44

Sportaktiviteter ... 45

Sexualvanor ... 52

Mobil och trådlöst ... 53

Husdjur ... 54

Egenvård ... 57

Receptfria mediciner ... 57

Alternativmedicinska produkter, behandlingar, träningstekniker ... 57

Pubertet ... 58

Kvinnans hälsa ... 60

Tobak, alkohol och droganvändning ... 61

Rökvanor ... 61

Snus ... 63

Vänner ... 64

Alkohol ... 65

Droganvändning ... 67

Hälsohistoria ... 68

Tandhälsa ... 68

Infektioner ... 69

Sjukdomar ... 70

Hälsokontroll ... 75

Vaccination ... 77

Sömn ... 78

Napp ... 78

Tupplur ... 79

Sovtid ... 80

Sömnproblem 83
Huvudvärk 87
Hörsel 88
Astma och allergi 89
Astma 89
Allergi 90
Eksem 91
Skador 94
Mental hälsa 95
Temperament 95
Småbarnsbeteende 104
Föräldraskap 109
Familjeliv 111
Skreeningfrågor 116
Styrkor och svårigheter (SDQ) 116
Social förmåga (SAS) 123
Separationsångest [a] 125
Rädsla i sociala situationer [c] 127
Generaliserad ångest [g] 128
Depression [h] 130
Uppmärksamhet och aktivitet [j] 133
Besvärlighet och problematiskt uppförande [k] 135
Bantning, vikt och kroppsform (P) 136
Tics (Q) 137
Styrkor [n] 139
Uppföljningsfrågor 140
Utveckling (R) 140

Separation [a] 152
Sociala situationer [c]...158
Oro [g] 165
Depression [h]..170
Uppmärksamhet och aktivitet [j] 179
Uppförande [k] 184
Bantning, vikt och kroppsform (P) 193
Tics (Q) 204

Sociodemografi

Hushållets sammansättning

[soc_livefamily]

C.SC10. Bor {ditt barn, du} vanligen i en enda familj?

❍ Ja [1]   ❍ Nej, {han/hon, jag} alternerar mellan {sina, mina} föräldrar [2]
❍ Vet ej / vill ej svara [998]

[soc_livewith]

C.SC20. {Tänk på de två familjerna där {ditt barn, du} bor den största delen av tiden,}. Hur många personer bor {ditt barn, du} tillsammans med {(totalt),}?

Antal vuxna	Antal minderåriga
❍ 1 [1]<br>❍ 2 [2]<br>❍ 3[3]<br>❍ 4 [4]	❍ 1 [1]<br>❍ 2 [2]<br>❍ 3 [3]<br>❍ 4 [4]<br>❍ 5 [5]<br>❍ 6 [6]<br>❍ 7 [7]

Om ålder >= 4, visa C.SC30, C.SC40 och C.SC50

[soc_pmoney]

C.SC30. Får {ditt barn, du} fickpengar?

Ja
❍ Veckopeng [wee]
❍ Månadspeng [mon]
❍
❍ Nej [5]
❍ Vet ej / vill ej svara [998]

Om inte "nej", visa C.SC40 och C.SC50

[soc_pmoney_amount]

C.SC40. Hur mycket får {ditt barn, du} i fickpengar varje gång?

❍ (kr) [kr]____________ [kr_other] ❍ Vet ej / vill ej svara [998]

[soc_money_spend]

C.SC50. Hur brukar {ditt barn, du} använda {sina, dina} fickpengar?

❍ {Mitt barn, Jag} spenderar alla {sina, mina} pengar som {han/hon, jag} vill [1]

❍ {Jag, Mina föräldrar} kräver att {mitt barn, jag} använder en del av pengarna på vissa saker [2]

❍ Vet ej / vill ej svara [998]

Om ålder >= 11, visa C.SC60

[soc_extrajob]

C.SC60. Tjänar {ditt barn, du} extra pengar regelbundet {, utöver vecko- eller månadspengen}?

Ja

❑ Hjälper till hemma [hom]

❑ Har ett extrajobb (t.ex. på skollov eller helger) [ext]

❑ På annat sätt [oth]

❍ Nej [no]

❍ Vet ej / vill ej svara [998]

Livsstil

Hygien

- **A. OM ÅLDER ÄR 1-10 VISA NEDANSTÅENDE FRÅGOR**

[was_hand]

C.QL40. Hur ofta brukar {du tvätta händerna på ditt barn, ditt barn tvätta sina händer}?

❍ Mindre än 1 gång om dagen [1]
❍ 1 till 2 gånger om dagen [2]
❍ 3 till 4 gånger om dagen [3]
❍ 5 gånger om dagen eller oftare [4]
❍ Vet ej / vill ej svara [998]

[was_hand_soap]

C.QL50. Hur ofta använder {du tvål när du tvättar händerna på ditt barn, ditt barn tvål när han/hon tvättar händerna}?

❍ Aldrig eller nästan aldrig [1]
❍ Hälften av gångerna [2]
❍ Alltid [3]
❍ Vet ej / vill ej svara [998]

- **A. VILLKORET AVSLUTAS**
- **B. OM ÅLDER ÄR 0-10 VISA NEDANSTÅENDE FRÅGOR**

[was_shower]

C.QL60. Hur ofta brukar {du duscha eller bada ditt barn, ditt barn duscha eller bada}?

❍ Dagligen [1]
❍ 4 till 6 gånger i veckan [2]
❍ 2 till 3 gånger i veckan [3]
❍ 1 gång i veckan eller mindre ofta [4]
❍ Vet ej / vill ej svara [998]

[was_shower_soap]

C.QL70. Hur ofta använder {du tvål när du duschar eller badar ditt barn, ditt barn tvål när han/hon duschar eller badar}?

❍ Aldrig eller nästan aldrig [1]
❍ Alltid [3]

❍ Hälften av gångerna [2] ❍ Vet ej / vill ej svara [998]

❖ **B. VILLKORET AVSLUTAS**

Solning

- ❖ **A. OM ÅLDER >= 1, VISA QL730 TILL QL760**

[sun_skin]

QL730. Vilket av följande alternativ passar in på {ditt barn, dig} när {han/hon, du} vistas ute i solen?

❍ {Han/hon, du} bränner {mig, dig} lätt och blir aldrig brun [1]
❍ {Han/hon, du} blir lätt bränd men blir brun till slut [2]
❍ {Han/hon, du} blir bara bränd i starkt solljus och blir alltid brun [3]
❍ {Han/hon, du} blir aldrig bränd men alltid brun [4]
❍ {Han/hon, du} har lite mörkare hy – som människor från Medelhavsområdet [5]
❍ {Han/hon, du} är svart – av afrikanskt ursprung [6]
❍ Vet ej / vill ej svara [998]

[sun_bathe]

QL740. Hur mycket brukar {ditt barn, du} vistas i solen mitt på dagen jämfört med andra i samma ålder?

❍ Mer än andra [1]
❍ Lika mycket som andra [2]
❍ Mindre än andra [3]
❍ Vet ej / vill ej svara [998]

[sun_burned]

QL745. Har {ditt barn bränt sig, du bränt dig} i solen så att huden blev röd under det senaste året?

Ja
❍ 1 gång [1]
❍ 2 gånger [2]
❍ 3 till 5 gånger [3]
❍ Mer än 5 gånger [4]
❍ Nej [no]
❍ Vet ej / vill ej svara [998]

[sun_resort]

QL750. Hur många veckor vistades {ditt barn, du} på solsemester under det senaste året?

❍ Mindre än 1 vecka eller inte alls [1]
❍ 1 till 2 veckor [2]
❍ 3 till 4 veckor [3]
❍ 5 till 6 veckor [4]
❍ 7 veckor eller mer [5]
❍ Vet ej / vill ej svara [998]

[sun_protect]

QL760. Brukar du regelbundet skydda {ditt barn, dig} mot solen?

Ja
❑ Med solskyddskräm [lot]
❑ Med klädesplagg [clo]
❑ {Mitt barn, Du} håller {sig, dig} i skuggan eller inomhus [sha]
❑
❍ Nej [no]
❍ Vet ej / vill ej svara [998]

- **A. VILLKORET AVSLUTAS**

Livsstil

Matvanor barn 0-17 år

Intro

<u>ålder < 1:</u> Vi kommer att fråga dig om vad ditt barn ätit och druckit under de senaste månaderna. Om det inte finns ett alternativ som passar precis in på ditt barn, välj det som ligger närmast.

<u>ålder 1-5:</u> Vi kommer att fråga dig om vad ditt barn ätit och druckit under de senaste månaderna. Frågorna avser all mat och dryck som ditt barn intar under dygnet, i hemmet, på dagis och på andra platser.

<u>ålder >= 6:</u> Vi kommer att fråga dig om vad {ditt barn, du} ätit och druckit under de senaste månaderna. Frågorna avser all mat och dryck som {ditt barn, du} intar under dygnet, i hemmet, i skolan och på andra platser.

Amning

- **Z1. OM ENKÄTEN BESVARAS FÖR 0-12 MÅNADERS BABY VISA NEDAN FRÅGOR**

[c_die_nurse]

DI10. Ammas ditt barn för närvarande?

❍ Ja [1]
❍ Nej, men ammades förut [2]
❍ Nej, har aldrig ammats [3]
❍ Vet ej / vill ej svara [998]

Om DI10 är ”ja”, visa DI20

[c_die_nurse_full]

DI20. Helammas ditt barn för närvarande (utan tillägg av annan mat eller dryck)?

❍ Ja [1] ❍ Vet ej / vill ej svara [998]
❍ Nej [2]

Om DI10 är ”nej, men ammades förut”, visa DI30 och DI40

[c_die2_nurse_tot]

DI30. Hur länge ammades ditt barn sammanlagt (helt eller delvis)?

❍ Mindre än 6 månader [1] ❍ Vet ej / vill ej svara [998]
❍ 6 - 8 månader [2]
❍ 9 – 12 månader [3]

Om DI20 är ”nej” visa DI40

[c_die_nurse_only]

DI40. Hur länge fick ditt barn enbart bröstmjölk {, (utan tillägg av annan mat eller dryck)}?

❍ 0 - 2 månader [1] ❍ Mer än 6 månader [4]
❍ 3 - 4 månader [2] ❍ Vet ej / vill ej svara [998]
❍ 5 - 6 månader [3]

- **A. LÄMNA MODULEN OM DI20 BESVARAS ”JA”**
- **Z1. VILLKORET AVSLUTAS**
- **Z2. OM ENKÄTEN BESVARAS AV 1-2 ÅRINGAR VISA NEDAN FRÅGOR**

[c_die_nurse]

DI10. Ammas ditt barn för närvarande?

❍ Ja [1]
❍ Nej, har aldrig ammats [3]
❍ Nej, men ammades förut [2]
❍ Vet ej / vill ej svara [998]

Om DI10 är "nej, men ammades förut", visa DI30

[c_die_nurse_tot]

DI30. Hur länge ammades ditt barn sammanlagt (helt eller delvis)?

❍ Mindre än 6 månader [1]
❍ 6 – 8 månader [2]
❍ 9 - 12 månader [3]
❍ 13 – 18 månader [4]
❍ Mer än 18 månader [5]
❍ Vet ej / vill ej svara [998]

Om DI10 är "ja" eller "nej, men ammades förut", visa DI40

[c_die_nurse_only]

DI40. Hur länge fick ditt barn enbart bröstmjölk {, (utan tillägg av annan mat eller dryck)}?

❍ 0 - 2 månader [1]
❍ 3 - 4 månader [2]
❍ 5 - 6 månader [3]
❍ Mer än 6 månader [4]
❍ Vet ej / vill ej svara [998]

❖ **Z2. VILLKORET AVSLUTAS**

❖ **B. OM ENKÄT BESVARAS AV FÖRÄLDER ELLER AV BARN >= 15 ÅR, VISA NEDAN FRÅGOR**

[c_die_food1]

DI50. För de livsmedel {ditt barn, du} äter eller dricker minst en gång i månaden, välj i rullistan hur ofta {han/hon, du} brukar äta eller dricka dessa.

	Antal gånger [frq]
{< 6 år: Välling} [grue]	❍ 2 gånger per dag eller mer [1]<br>❍ 1 gång per dag [2]<br>❍ 3-6 gånger per vecka [3]<br>❍ 1-2 gånger per vecka [4]<br>❍ 1-3 gånger per månad [5]
Gröt [pori]	❍ 2 gånger per dag eller mer [1]<br>❍ 1 gång per dag [2]<br>❍ 3-6 gånger per vecka [3]<br>❍ 1-2 gånger per vecka [4]<br>❍ 1-3 gånger per månad [5]
{ålder >= 6: Mellanmålsgröt (t.ex. risifrutti eller mannafrutti)} [risi]	❍ 2 gånger per dag eller mer [1]<br>❍ 1 gång per dag [2]<br>❍ 3-6 gånger per vecka [3]<br>❍ 1-2 gånger per vecka [4]<br>❍ 1-3 gånger per månad [5]
Fil eller yoghurt [yogh]	❍ 2 gånger per dag eller mer [1]<br>❍ 1 gång per dag [2]<br>❍ 3-6 gånger per vecka [3]<br>❍ 1-2 gånger per vecka [4]<br>❍ 1-3 gånger per månad [5]
{>= 1 år: Flingor eller müsli } [musl]	❍ 2 gånger per dag eller mer [1]<br>❍ 1 gång per dag [2]<br>❍ 3-6 gånger per vecka [3]<br>❍ 1-2 gånger per vecka [4]<br>❍ 1-3 gånger per månad [5]
{>= 6 år: Chokladdryck (t.ex. varm choklad eller O'boy)} [oboy]	❍ 2 gånger per dag eller mer [1]<br>❍ 1 gång per dag [2]<br>❍ 3-6 gånger per vecka [3]<br>❍ 1-2 gånger per vecka [4]<br>❍ 1-3 gånger per månad [5]
Mjölk {1-5 år:, inkl. mjölkersättning} {>= 6 år:, i glas eller tallrik} [milk]	❍ 2 gånger per dag eller mer [1]<br>❍ 1 gång per dag [2]<br>❍ 3-6 gånger per vecka [3]

	Antal gånger [frq]
	❍ 1-2 gånger per vecka [4]<br>❍ 1-3 gånger per månad [5]
{< 1 år: Modersmjölksersättning eller mjölkersättning (t.ex. NAN, Babysemp eller Nutramigen)} [milr]	❍ 2 gånger per dag eller mer [1]<br>❍ 1 gång per dag [2]<br>❍ 3-6 gånger per vecka [3]<br>❍ 1-2 gånger per vecka [4]<br>❍ 1-3 gånger per månad [5]
Juice {< 1 år: eller barndryck} [juic]	❍ 2 gånger per dag eller mer [1]<br>❍ 1 gång per dag [2]<br>❍ 3-6 gånger per vecka [3]<br>❍ 1-2 gånger per vecka [4]<br>❍ 1-3 gånger per månad [5]
>= 6 år: Te [tea]	❍ 2 gånger per dag eller mer [1]<br>❍ 1 gång per dag [2]<br>❍ 3-6 gånger per vecka [3]<br>❍ 1-2 gånger per vecka [4]<br>❍ 1-3 gånger per månad [5]
>= 15 år: Kaffe [coff]	❍ 2 gånger per dag eller mer [1]<br>❍ 1 gång per dag [2]<br>❍ 3-6 gånger per vecka [3]<br>❍ 1-2 gånger per vecka [4]<br>❍ 1-3 gånger per månad [5]
{< 3 år: Vatten, 1 dl per gång; >= 3 år: Vatten (från kran eller på flaska), 1 glas per gång} [wate]	❍ 2 gånger per dag eller mer [1]<br>❍ 1 gång per dag [2]<br>❍ 3-6 gånger per vecka [3]<br>❍ 1-2 gånger per vecka [4]<br>❍ 1-3 gånger per månad [5]

- **B1. OM ÅLDER < 3 OCH DI50 BESVARAS ”VÄLLING”, ”GRÖT” ELLER ”FIL…” ELLER ”FLINGOR…” 1-2 GÅNGER PER VECKA ELLER MER VISA NEDAN FRÅGOR**
- **B2. OM ÅLDER < 6 OCH DI50 BESVARAS ”GRÖT” ELLER OM ÅLDER >= 3 OCH DI50 BESVARATS ”FIL…” ELLER ”FLINGOR…” 1-2 GÅNGER PER VECKA ELLER MER VISA NEDAN FRÅGOR**

Du nämnde att ditt barn {äter, dricker} {nyckelfraser från DI50}.

Om DI50 är ”välling”, visa DI51

[c_die2_food1_grue_kind]

DI51. Vilka av följande vällingsorter dricker ditt barn oftast? (Markera alla aktuella)

❑ Mild fullkornsvälling [mil]
❑ Fullkornsvälling [ful]
❑ Majsvälling eller glutenfri välling [cor]
❑ Annan välling [oth]
❍ Vet ej / vill inte svara [998]

Om DI50 är ”gröt”, visa DI52

[c_die_food1_pori_kind]

DI52. Vilka av följande grötsorter äter ditt barn oftast? (Markera alla aktuella)

❑ Havregrynsgröt [oat]
❑ Barngröt pulverbaserad (t.ex. fullkornsgröt eller fruktgröt) [chi]
❑ Mannagrynsgröt eller risgrynsgröt [man]
❑ Mellanmålsgröt (t.ex. risifrutti) [risi]
❑ Annan gröt [oth]
❍ Vet ej / vill inte svara [998]

Om DI50 är ”fil...”, visa DI54

[c_die2_food1_yogh_kind]

DI54. Vilken av följande fil- eller yoghurtsorter äter {ditt barn, du} oftast?

❍ Naturell [nat]
❍ Frukt-, bär- eller vaniljsmak [fru]
❍ Vet ej / vill inte svara [998]

Om DI50 är ”flingor...”, visa DI55

[c_die_food1_musl_kind]

DI55. Vilka av följande flingor eller müslisorter äter {ditt barn, du} oftast? (Markera alla aktuella)

❑ Cornflakes eller Special K [corn]
❑ Müsli eller fullkornsflingor [musl]
❑ Havrefras/kuddar eller cheerios [oat]
❑ Coco-pops, Kalaspuffar eller sötade flingor [coco]
❑ Andra flingor [oth]
❍ Vet ej / vill inte svara [998]

- ❖ **B1 OCH B2. VILLKOREN AVSLUTAS**
- ❖ **B3. OM KAFFE ELLER TE I OVAN DI50 BESVARATS 1-2 GÅNGER PER VECKA ELLER OFTARE VISA NEDANSTÅENDE FRÅGOR**

Du nämnde att du dricker {nyckelfraser för te och kaffe från DI50}.

Om kaffe, visa DI56

[c_die_food1_coff_acc]

DI56. Vad brukar du ha i ditt kaffe? (Markera alla aktuella)

❑ Mjölk (t.ex. i cappuccino eller latte) [milk]
❑ Socker [suga]
❑ Sötningsmedel [swee]
❍ Inget av dessa [996]
❍ Vet ej / vill ej svara [998]

Om te, visa DI57 och DI58

[c_die_food1_tea_kind]

DI57. Vilka följande tesorter brukar du oftast dricka? (Markera alla aktuella)

❑ Svart te [blac]
❑ Grönt te [gree]
❑ Rött te [red]
❑ Örtte [herb]
❑ Annan tesort [oth]
❍ Vet ej / vill ej svara [998]

[c_die_food1_tea_acc]

DI58. Vad brukar du ha i ditt te? (Markera alla aktuella)

❑ Mjölk [milk]
❑ Socker eller honung [suga]
❑ Sötningsmedel [swee]
❍ Inget av dessa [996]
❍ Vet ej / vill ej svara [998]

❖ **B OCH B3. VILLKOREN AVSLUTAS**

❖ **C1. OM ENKÄT BESVARAS AV FÖRÄLDER ELLER AV BARN >= 15 ÅR, VISA NEDAN FRÅGOR**

[c_die_food2]

DI60. För de livsmedel {ditt barn, du} äter minst en gång i månaden, välj i rullistan hur ofta {han/hon, du} brukar äta dessa.

	Antal gånger [frq]
{<u><1 år:</u> Fruktpuré eller dessert på burk; <u><2 år:</u> Fruktpuré (barnmatsburk eller bägare)} [pure]	❍ 2 gånger per dag eller mer [1]<br>❍ 1 gång per dag [2]<br>❍ 3-6 gånger per vecka [3]<br>❍ 1-2 gånger per vecka [4]<br>❍ 1-3 gånger per månad [5]
{<u>1-14 år:</u> Fruktsoppa, fruktkräm (t.ex. nyponsoppa eller blåbärssoppa)} [soup]	❍ 2 gånger per dag eller mer [1]<br>❍ 1 gång per dag [2]<br>❍ 3-6 gånger per vecka [3]<br>❍ 1-2 gånger per vecka [4]<br>❍ 1-3 gånger per månad [5]
{<u>1-5 år:</u> Sylt eller äppelmos} [jam]	❍ 2 gånger per dag eller mer [1]<br>❍ 1 gång per dag [2]<br>❍ 3-6 gånger per vecka [3]<br>❍ 1-2 gånger per vecka [4]<br>❍ 1-3 gånger per månad [5]
{<u>< 1 år:</u> Frukt, bär eller hemlagad puré, <u>>= 1 år:</u> Frukt eller bär} [frui]	❍ 2 gånger per dag eller mer [1]<br>❍ 1 gång per dag [2]<br>❍ 3-6 gånger per vecka [3]<br>❍ 1-2 gånger per vecka [4]<br>❍ 1-3 gånger per månad [5]
Vitt bröd (t.ex. formfranska, polarbröd eller limpa) [brew]	❍ 2 gånger per dag eller mer [1]<br>❍ 1 gång per dag [2]<br>❍ 3-6 gånger per vecka [3]<br>❍ 1-2 gånger per vecka [4]<br>❍ 1-3 gånger per månad [5]
Grovt bröd (t.ex. fullkornsbröd eller knäckebröd) [bred]	❍ 2 gånger per dag eller mer [1]<br>❍ 1 gång per dag [2]<br>❍ 3-6 gånger per vecka [3]<br>❍ 1-2 gånger per vecka [4]<br>❍ 1-3 gånger per månad [5]
{<u>< 1 år:</u> Smörgåsrån eller majsbågar; <u>>= 1 år:</u> Skorpor, riskakor eller digestive} [bisc]	❍ 2 gånger per dag eller mer [1]<br>❍ 1 gång per dag [2]<br>❍ 3-6 gånger per vecka [3]<br>❍ 1-2 gånger per vecka [4]<br>❍ 1-3 gånger per månad [5]

	Antal gånger [frq]
Smör eller margarin på smörgås [butt]	❍ 2 gånger per dag eller mer [1]<br>❍ 1 gång per dag [2]<br>❍ 3-6 gånger per vecka [3]<br>❍ 1-2 gånger per vecka [4]<br>❍ 1-3 gånger per månad [5]
Pålägg [spre]	❍ 2 gånger per dag eller mer [1]<br>❍ 1 gång per dag [2]<br>❍ 3-6 gånger per vecka [3]<br>❍ 1-2 gånger per vecka [4]<br>❍ 1-3 gånger per månad [5]
Ägg [egg]	❍ 2 gånger per dag eller mer [1]<br>❍ 1 gång per dag [2]<br>❍ 3-6 gånger per vecka [3]<br>❍ 1-2 gånger per vecka [4]<br>❍ 1-3 gånger per månad [5]

- **C2. OM ”SMÖR…” ELLER ”PÅLÄGG” ELLER ”FRUKT…” I OVAN DI60 BESVARATS 1-2 GÅNGER PER VECKA ELLER MER VISA NEDANSTÅENDE FRÅGOR**

Du nämnde att {ditt barn, du} äter {nyckelfraser för frukt... samt smör... och pålägg från DI60}.

Om ”frukt…”, visa DI61

[c_die_food2_frui_kind]

DI61. Vilka av följande frukter och bär äter {ditt barn, du} oftast{<u><3år:</u> (hel, i bitar, eller i hemlagad puré)}? (Markera alla aktuella)

❑ Banan [ban]
❑ Äpple eller päron [app]
❑ Citrus (t.ex. apelsin eller clementin) [cit]
❑ Bär (t.ex. blåbär, hallon eller jordgubbar) [ber]
❑ Kiwi [kiw]
❑ Vindruvor [gra]
❑ Annan frukt [oth]
❍ Vet ej / vill ej svara [998]

Om "smör…", visa DI62

[c_die_food2_butt_kind]

DI62. Vilka av följande slags smör och margarin äter {ditt barn, du} oftast? (Markera alla aktuella)

❑ Bregott [bre]
❑ Smörgåsmargarin (t.ex. Lätta, Lätt och Lagom eller Becel) [marl]
❑ Smör [butt]
❑ Annat smörgåsfett [oth]
❍ Vet ej / vill ej svara [998]

Om "pålägg", visa DI63

[c_die_food2_spre_kind]

DI64. Vilka av följande pålägg äter {ditt barn, du} oftast? (Markera alla aktuella)

❑ Ost [che]
❑ Messmör [whey]
❑ Leverpastej [live]
❑ Köttpålägg (t.ex. skinka eller salami) [meat]
❑ Kaviar [kavi]
❑ Marmelad [marm]
❑ Gurka [cucu]
❑ Tomat eller paprika [toma]
❑ Annat pålägg [oth]
❍ Vet ej / vill ej svara [998]

❖ **C1 OCH C2. VILLKOREN AVSLUTAS**

Maträtter

❖ **D. OM ENKÄT BESVARAS AV FÖRÄLDRAR ELLER AV BARN >= 15 ÅR, VISA NEDAN FRÅGOR**

ålder < 1: Vi frågar nu om lunch och middag både i hemmet och på andra platser. Frågorna gäller både lagad mat och barnmat på burk.

ålder 1-5: Vi frågar nu om lunch och middag både i hemmet, på dagis och på andra platser. Frågorna gäller både lagad mat och barnmat på burk.

ålder >= 6: Vi frågar nu om lunch och middag både i hemmet, i skolan och på andra platser.

[c_die_meal1]

DI70. För de maträtter {ditt barn, du} äter minst en gång i månaden, välj i rullistan hur ofta {han/hon, du} brukar äta dessa.

	Antal gånger [frq]
{ålder >= 1: Hamburgare med bröd eller Tex-mex (t.ex. tacos); ålder>=15: Hamburgare med bröd, kebab med bröd eller Tex-mex (t.ex. tacos)} [hamb]	❍ 7 gånger per vecka eller mer [1]<br>❍ 3-6 gånger per vecka [2]<br>❍ 1-2 gånger per vecka [3]<br>❍ 1-3 gånger per månad [4]
Köttfärsrätter {ålder < 1: (t.ex. köttfärssås eller köttbullar); ålder >= 1: (t.ex. köttfärssås, köttbullar eller pannbiff)} [minc]	❍ 7 gånger per vecka eller mer [1]<br>❍ 3-6 gånger per vecka [2]<br>❍ 1-2 gånger per vecka [3]<br>❍ 1-3 gånger per månad [4]
{ålder >= 1: Lasagne, moussaka eller tortellini med kött} [lasa]	❍ 7 gånger per vecka eller mer [1]<br>❍ 3-6 gånger per vecka [2]<br>❍ 1-2 gånger per vecka [3]<br>❍ 1-3 gånger per månad [4]
Kötträtter {ålder < 1: (t.ex. gryta); ålder >= 1: (t.ex. stek, gryta eller wok)} [meat]	❍ 7 gånger per vecka eller mer [1]<br>❍ 3-6 gånger per vecka [2]<br>❍ 1-2 gånger per vecka [3]<br>❍ 1-3 gånger per månad [4]
Korvrätter {ålder >= 1: (t.ex. stekt, kokt, gratinerad eller i gryta)} [sauc]	❍ 7 gånger per vecka eller mer [1]<br>❍ 3-6 gånger per vecka [2]<br>❍ 1-2 gånger per vecka [3]<br>❍ 1-3 gånger per månad [4]
Blodpudding [blac]	❍ 7 gånger per vecka eller mer [1]<br>❍ 3-6 gånger per vecka [2]<br>❍ 1-2 gånger per vecka [3]<br>❍ 1-3 gånger per månad [4]
Kyckling eller kalkon {ålder >= 1: (t.ex. grillad, bits, wok eller i gryta)} [chic]	❍ 7 gånger per vecka eller mer [1]<br>❍ 3-6 gånger per vecka [2]<br>❍ 1-2 gånger per vecka [3]<br>❍ 1-3 gånger per månad [4]
Fisk eller skaldjur [fish]	❍ 7 gånger per vecka eller mer [1]<br>❍ 3-6 gånger per vecka [2]<br>❍ 1-2 gånger per vecka [3]<br>❍ 1-3 gånger per månad [4]
Vegetariska rätter (t.ex. linsgryta, bönbiffar, sojakorv eller quorn) [vego]	❍ 7 gånger per vecka eller mer [1]<br>❍ 3-6 gånger per vecka [2]<br>❍ 1-2 gånger per vecka [3]

	Antal gånger [frq]
	❍ 1-3 gånger per månad [4]

❖ **D1. OM ”FISK…” I OVAN DI70 BESVARATS 1-2 GÅNGER PER VECKA ELLER MER ELLER OM ”VEGETARISKA RÄTTER…” BESVARATS 3-6 GÅNGER PER VECKA ELLER MER VISA NEDANSTÅENDE FRÅGA**

Om ”fisk…”, visa DI171

[c_die2_meal1_fish_kind]

DI71. Du nämnde att {ditt barn, du} äter fisk eller skaldjur. Vilka av följande äter {ditt barn, du} oftast? (Markera alla aktuella)

❑ Fiskpinnar eller fiskbullar [stic]
❑ Torsk, sej eller annan vit fisk (ej fiskpinnar och fiskbullar) [whit]
❑ Lax {ålder >= 1: sushi}, makrill eller sill [salm]
❑ Tonfisk [funa]
❑ Skaldjur (t.ex. räkor eller musslor) [shel]
❑ Annan fisk [oth]
❍ Vet ej / vill ej svara [998]

Om ”vegetariska rätter…” , visa DI72

[c_die2_meal1_vego_kind]

DI72. Vilka av följande vegetariska rätter äter du vanligtvis? (Markera alla aktuella)

❑ Bön-, lins- eller kikärtsrätter (t.ex. linsgryta, bönbiff eller falafel) [been]
❑ Sojakött, sojakorv eller tofu [soya]
❑ Quornfärs eller quorngryta [quor]
❑ Rotfruktsrätter (t.ex. rödbetor, palsternacka eller kålrot) [root]
❍ Vet ej / vill ej svara [998]

❖ **D1. VILLKORET AVSLUTAS**

❖ **E. OM BESVARAS AV FÖRÄLDER ELLER BESVARAS AV BARN >= 15 ÅR, VISA NEDAN FRÅGOR**

[c_die_meal2]

DI80. För de maträtter och livsmedel {ditt barn, du} äter minst en gång i månaden, välj i rullistan hur ofta {ditt barn, du} brukar äta dessa.

	Antal gånger [frq]
ålder >= 1: Pizza, panpizza, pirog eller paj [pizz]	❍ 7 gånger per vecka eller mer [1]<br>❍ 3-6 gånger per vecka [2]<br>❍ 1-2 gånger per vecka [3]<br>❍ 1-3 gånger per månad [4]
Pannkakor, plättar, våfflor eller ugnspannkaka [pann]	❍ 7 gånger per vecka eller mer [1]<br>❍ 3-6 gånger per vecka [2]<br>❍ 1-2 gånger per vecka [3]<br>❍ 1-3 gånger per månad [4]
ålder >= 1: Soppa (t.ex. tomatsoppa, ärtsoppa eller gulaschsoppa) [soup]	❍ 7 gånger per vecka eller mer [1]<br>❍ 3-6 gånger per vecka [2]<br>❍ 1-2 gånger per vecka [3]<br>❍ 1-3 gånger per månad [4]
Grönsaker {ålder < 1: (råa, tillagade eller puré); ålder >= 1: (råa eller tillagade)} [vege]	❍ 7 gånger per vecka eller mer [1]<br>❍ 3-6 gånger per vecka [2]<br>❍ 1-2 gånger per vecka [3]<br>❍ 1-3 gånger per månad [4]
Potatis (kokt eller mos) [pota]	❍ 7 gånger per vecka eller mer [1]<br>❍ 3-6 gånger per vecka [2]<br>❍ 1-2 gånger per vecka [3]<br>❍ 1-3 gånger per månad [4]
ålder >= 1: Pommes frites, potatisbullar, stekt potatis, klyftpotatis, potatisgratäng eller pytt i panna [pomm]	❍ 7 gånger per vecka eller mer [1]<br>❍ 3-6 gånger per vecka [2]<br>❍ 1-2 gånger per vecka [3]<br>❍ 1-3 gånger per månad [4]
Ris [rice]	❍ 7 gånger per vecka eller mer [1]<br>❍ 3-6 gånger per vecka [2]<br>❍ 1-2 gånger per vecka [3]<br>❍ 1-3 gånger per månad [4]
Pasta { ålder >= 1: eller nudlar} [past]	❍ 7 gånger per vecka eller mer [1]<br>❍ 3-6 gånger per vecka [2]<br>❍ 1-2 gånger per vecka [3]<br>❍ 1-3 gånger per månad [4]
ålder >= 1: Bulgur, cous cous eller quinoa [bulg]	❍ 7 gånger per vecka eller mer [1]<br>❍ 3-6 gånger per vecka [2]<br>❍ 1-2 gånger per vecka [3]<br>❍ 1-3 gånger per månad [4]
ålder >= 1: Tillbehör (t.ex. ketchup eller sås) [sauc]	❍ 7 gånger per vecka eller mer [1]<br>❍ 3-6 gånger per vecka [2]<br>❍ 1-2 gånger per vecka [3]<br>❍ 1-3 gånger per månad [4]

- **E. VILLKORET AVSLUTAS**
- **F. OM ”GRÖNSAKER” I DI80 BESVARATS 1-2 GÅNGER PER VECKA ELLER OFTARE ELLER OM ”TILLBEHÖR…” I DI80 BESVARATS ”1-2 GÅNGER PER VECKA” ELLER MER VISA NEDAN FRÅGOR**

Du nämnde att ditt barn {äter grönsaker, har tillbehör i maten}.

Om ”grönsaker…” besvarats ”7 gånger per vecka eller mer”, visa DI101

[c_die_meal2_vege_frq]

DI101. Hur många gånger per dag äter {ditt barn, du} grönsaker?

❍ 1 gång [1___]
❍ 2 gånger [2___]
❍ 3 gånger eller mer [3___]
❍ Vet ej / vill ej svara [998]

[c_die2_meal2_vege_kind]

DI102. Vilka av följande grönsaker äter {ditt barn, du} oftast? (Markera alla aktuella)

❑ Tomat eller paprika [toma]
❑ Gurka, salladsblad eller zucchini [cucu]
❑ Morötter [carr]
❑ Lök [onio]
❑ Majs [corn]
❑ Avocado [avoc]
❑ Blomkål eller vitkål (t.ex. pizzasallad) [cabb]
❑ Broccoli [broc]
❑ Spenat [spin]
❑ Gröna ärtor [pees]
❑ Bönor, linser eller kikärter [been]
❑ Palsternacka, kålrot eller rotselleri [pars]
❑ Inlagda grönsaker [cons]
❑ Annan grönsak [oth]
❍ Vet ej / vill ej svara [998]

Om ”tillbehör…”, visa DI103

[c_die_meal2_sauc_kind]

DI103. Vilka av följande tillbehör har {ditt barn, du} oftast i maten? (Markera alla aktuella)

❑ Ketchup, tomatsås eller tomatsalsa [toma]
❍ Vet ej / vill ej svara [998]

❑ Vinägrett (olja och vinäger) [vina]
❑ Gräddsås, crème fraiche eller gräddfilssås [crem]
❑ Bearnaiessås eller hamburgersås [bear]
❑ Annan sås [oth]

❖ **D OCH F. VILLKOREN AVSLUTAS**

❖ **G1. OM ÅLDER < 3 VISA NEDAN FRÅGOR**

[c_die_can]

DI110. Hur stor del av maträtterna som ditt barn äter till lunch och middag är barnmat på burk?

❍ Nästan alla rätter [1]
❍ Hälften av rätterna [2]
❍ En fjärdedel av rätterna [3]
❍ Enstaka rätter [4]
❍ Inga rätter alls [5]
❍ Vet ej / vill ej svara [998]

Om ålder < 1

[c_die_port_infant]

DI111. Hur mycket mat äter ditt barn vid varje måltid (lagad mat eller barnmat på burk)?

1 burk motsvarar 200 g eller 2 dl mat.

❍ Mer än en burk [1]
❍ En burk [2]
❍ En halv burk [3]
❍ Mindre än en halv burk [4]
❍ Vet ej / vill ej svara [998]

❖ **G1. VILLKORET AVSLUTAS**

❖ **G2. OM ÅLDER MELLAN 1 OCH 11 OCH BESVARAS AV FÖRÄLDER ELLER OM ENKÄTEN BESVARAS AV BARN VISA NEDAN FRÅGOR**

[c_die_port_pota]

DI112. Hur stor portion äter {ditt barn, du} vanligtvis till lunch och middag av följande? (Markera portionsstorlekar för de livsmedel som gäller dig)

	[1]	[2]	[3]	[4]	[5]
Ris, potatis, pasta m.m.	☐	☐	☐	☐	☐

[c_die_port_prot]

	[1]	[2]	[3]	[4]	[5]
Kött, fisk eller vegetariskt alternativ.	☐	☐	☐	☐	☐

[c_die_port_vege]

	[1]	[2]	[3]	[4]	[5]
Grönsaker (råa eller tillagade).	☐	☐	☐	☐	☐

❖ **G2. VILLKORET AVSLUTAS**

❖ **H. OM ÅLDER >= 1**

[c_die2_meals]

DI120. Hur ofta brukar {ditt barn, du} äta eller dricka något vid följande måltider?

	Ange hur ofta [frq]
Frukost [brea]	❍ Varje dag [1]<br>❍ Flera gånger per vecka [2]<br>❍ Någon gång per vecka [3]<br>❍ Mer sällan eller aldrig [4]
Lunch [lunc]	❍ Varje dag [1]<br>❍ Flera gånger per vecka [2]<br>❍ Någon gång per vecka [3]<br>❍ Mer sällan eller aldrig [4]
Middag [dinn]	❍ Varje dag [1]<br>❍ Flera gånger per vecka [2]<br>❍ Någon gång per vecka [3]<br>❍ Mer sällan eller aldrig [4]

[c_die_meals_coff_frq]

DI125. Hur ofta brukar {ditt barn, du} fika eller äta mellanmål{, eller ha fruktpaus}?

❍ 4 gånger per dag eller mer [1]
❍ 3 gånger per dag [2]
❍ 1 - 2 gånger per dag [3]
❍ Några gånger per vecka [4]
❍ Mer sällan eller aldrig [5]
❍ Vet ej / vill ej svara [998]

Om ålder >= 6, visa DI125

[c_die_restaurang]

DI127. Hur ofta äter {ditt barn, du} från salladsbuffén i skolan eller på restaurang?

❍ Varje dag [1]
❍ Flera gånger per vecka [2]
❍ Någon gång per vecka [3]
❍ Mer sällan eller aldrig [4]
❍ Vet ej / vill ej svara [998]

[c_die_fastfood]

DI130. Hur ofta äter {ditt barn, du} på hamburgerrestaurang, pizzeria eller gatukök?

❍ Varje dag [1]
❍ Flera gånger per vecka [2]
❍ Någon gång per vecka [3]
❍ Mer sällan eller aldrig [4]
❍ Vet ej / vill ej svara [998]

[c_die_snac]

DI140. För de snacks och sötsaker {ditt barn, du} äter eller dricker minst en gång i månaden, välj i rullistan hur ofta {han/hon, du} brukar äta eller dricka dessa.

	Antal ganger [frq]
{<u>ålder < 6:</u> Läsk eller saft; <u>ålder >= 6:</u> Läsk, saft eller energidryck} [soda]	❍ 7 gånger per vecka eller mer [1]<br>❍ 3-6 gånger per vecka [2]<br>❍ 1-2 gånger per vecka [3]<br>❍ 1-3 gånger per månad [4]
{<u>ålder >= 15:</u> Proteindryck} [prot]	❍ 7 gånger per vecka eller mer [1]<br>❍ 3-6 gånger per vecka [2]<br>❍ 1-2 gånger per vecka [3]<br>❍ 1-3 gånger per månad [4]
{<u>ålder > 1:</u> Bullar, kex eller kakor; <u>ålder >= 15:</u>	❍ 7 gånger per vecka eller mer [1]

	Antal ganger [frq]
kex eller småkakor} [cook]	❍ 3-6 gånger per vecka [2]<br>❍ 1-2 gånger per vecka [3]<br>❍ 1-3 gånger per månad [4]
{ålder >= 15: Bulle, muffin eller mjuk kaka} [muff]	❍ 7 gånger per vecka eller mer [1]<br>❍ 3-6 gånger per vecka [2]<br>❍ 1-2 gånger per vecka [3]<br>❍ 1-3 gånger per månad [4]
{ålder > 1: Glass; ålder >= 15: Glass, sorbet eller parfait} [icec]	❍ 7 gånger per vecka eller mer [1]<br>❍ 3-6 gånger per vecka [2]<br>❍ 1-2 gånger per vecka [3]<br>❍ 1-3 gånger per månad [4]
{ålder > 1: Snacks (t.ex. chips, popcorn eller ostbågar); ålder >= 15: Chips eller ostbågar} [snac]	❍ 7 gånger per vecka eller mer [1]<br>❍ 3-6 gånger per vecka [2]<br>❍ 1-2 gånger per vecka [3]<br>❍ 1-3 gånger per månad [4]
{ålder > 1: Nötter; ålder >= 15: Nötter, mandlar eller frön} [nuts]	❍ 7 gånger per vecka eller mer [1]<br>❍ 3-6 gånger per vecka [2]<br>❍ 1-2 gånger per vecka [3]<br>❍ 1-3 gånger per månad [4]
{ålder > 1: Choklad, godis eller naturgodis; ålder >= 15: Godis (ej choklad)} [cand]	❍ 7 gånger per vecka eller mer [1]<br>❍ 3-6 gånger per vecka [2]<br>❍ 1-2 gånger per vecka [3]<br>❍ 1-3 gånger per månad [4]
{ålder >= 15: Choklad} [choc]	❍ 7 gånger per vecka eller mer [1]<br>❍ 3-6 gånger per vecka [2]<br>❍ 1-2 gånger per vecka [3]<br>❍ 1-3 gånger per månad [4]
{ålder < 6: Russin; ålder >= 6: Torkad frukt; ålder >= 15: Torkad frukt eller naturgodis} [rais]	❍ 7 gånger per vecka eller mer [1]<br>❍ 3-6 gånger per vecka [2]<br>❍ 1-2 gånger per vecka [3]<br>❍ 1-3 gånger per månad [4]

- **I1. OM ÅLDER >= 3 VISA NEDAN FRÅGOR**
- **I2. OM ”LÄSK…” ELLER ”GLASS” VISA FRÅGA DI141**

Du nämnde att {ditt barn, du} {äter, dricker} {nyckelfraser för läsk och glass i DI40}.

Om “läsk och saft”, visa DI141

[c_die2_snac_soda_kind]

DI141. Vilken slags läsk eller saft dricker {ditt barn, du} oftast? (Markera alla aktuella)

❑ Light-läsk eller light-saft (med sötningsmedel) [ligh] ❍ Vet ej / vill ej svara [998]
❑ Läsk eller saft (med socker) [suga]
❑ {ålder >= 6: Energidryck (t.ex. Redbull)} [ener]

Om "glass", visa DI142

[c_die_snac_icec_kind]

DI142. Hur mycket glass brukar {ditt barn, du} äta vid varje tillfälle?

1 glasskula motsvarar 50 g eller cirka 1 dl.

❍ Mindre än 1 kula [1] ❍ Vet ej / vill ej svara [998]
❍ 1 kula [2]
❍ 2 kulor [3]
❍ 3 kulor eller mer [4]

- **I2. VILLKOREN AVSLUTAS**
- **J. OM "SNACKS", "CHOKLAD…", "CHOKLAD" ELLER "GODIS…" VISA FRÅGA DI143**

Du nämnde att {ditt barn, du} äter {nyckelfraser för snacks och choklad i DI140}.

Om "snacks", visa DI143

[c_die_snac_snac_kind]

DI143. Hur mycket chips {ålder < 15: , popcorn} eller ostbågar brukar {ditt barn, du} äta vid varje tillfälle?

1 vanlig chipspåse väger 200 g.

❍ {ålder < 15: Enstaka chips, popcorn eller ostbågar, ålder >= 15: Enstaka chips eller ostbågar} [1] ❍ Vet ej / vill ej svara [998]
❍ 25 g [2]
❍ 50 g [3]
❍ 100 g {ålder < 6: eller mer} [4]

❍ {ålder >= 6: 200 g eller mer} [5]

Om "choklad..." eller ”godis...", visa DI144

[c_die2_snac_cand_kind]

DI144. Hur mycket {om ”choklad...": choklad, godis eller naturgodis; om ”godis...": godis} brukar {ditt barn, du} äta vid varje tillfälle?

{om ”choklad...": om ålder < 6: En chokladkaka motsvarar 100 g.; om ålder >= 6: En stor chokladkaka motsvarar 200 g.}

En godispåse, t.ex. Bilar eller Gott&Blandat, motsvarar 150g. 1 tablettask motsvarar 25 g.

❍ Enstaka godisbitar {om ”choklad...": eller chokladrutor} [1]
❍ 50 g [2]
❍ 100 g {ålder < 6: eller mer} [3]
❍ {ålder >= 6: 200 g eller mer} [4]
❍ Vet ej / vill ej svara [998]

Om "choklad", visa DI145

[c_die_snac_choc_kind]

DI145. Hur mycket choklad brukar du äta vid varje tillfälle?

En stor chokladkaka motsvarar 200 g.

❍ Enstaka chokladrutor [1]
❍ 50 g [2]
❍ 100 g [3]
❍ 200 g eller mer [4]
❍ Vet ej / vill ej svara [998]

- **I, H OCH J. VILLKOREN AVSLUTAS**
- **K. OM ENKÄT BESVARAS AV FÖRÄLDER ELLER AV BARN >= 15 ÅR VISA NEDAN FRÅGOR**

[c_die_prob]

DI150. Äter {ditt barn, du} produkter med probiotika, dvs. "goda bakterier" som finns tillsatta i exempelvis fruktdryck som Proviva, fil som Verum hälsofil och A-fil {ålder < 3: och probiotikaberikad gröt eller välling}?

❍ Ja [1] ❍ Vet ej / vill ej svara [998]
❍ Nej [2]

Om DI18 är "ja", visa DI51

[c_die_prob_frq]

DI151. Hur ofta äter {ditt barn, du} produkter med probiotika?

❍ Varje dag [1] ❍ Mer sällan eller aldrig [4]
❍ Flera gånger per vecka [2] ❍ Vet ej / vill ej svara [998]
❍ Någon gång per vecka [3]

- **L. OM ÅLDER < 3 VISA NEDAN FRÅGOR**

DI160. Hur gammal var ditt barn när han/hon fick börja smaka på följande livsmedel?

[c_die_taste_egg]

DI161. Ägg.

❍ (Månader gammal) [mnt] _____ [mnt_other] ❍ Vet ej / vill ej svara [998]
❍ Har inte ännu fått smaka [none]

[c_die_taste_fis]

DI162. Fisk.

❍ (Månader gammal) [mnt] _____ [mnt_other] ❍ Vet ej / vill ej svara [998]
❍ Har inte ännu fått smaka [none]

[c_die_taste_gra]

DI163. Vete, havre, råg, korn. (Finns i kex, skorpa, gröt, välling m.m.)

❍ (Månader gammal) [mnt] _____ [mnt_other] ❍ Vet ej / vill ej svara [998]
❍ Har inte ännu fått smaka [none]

❖ **K OCH L. VILLKOREN AVSLUTAS**

Kosttillskott

❖ **M. OM ÅLDER >= 1 OCH ENKÄT BESVARAS AV FÖRÄLDER ELLER AV BARN >= 15 ÅR VISA NEDAN FRÅGOR**

[c_die_suppl_use]

DI164. Brukar {ditt barn, du} äta vitaminer, mineraler eller andra tillskott?

❍ Ja, regelbundet [1]
❍ Ja, ibland [2]
❍ Nej [3]
❍ Vet ej / vill ej svara [998]

Om DI164 är ”Ja,…”, visa DI165

[c_die_suppl_vit]

DI165. För de kosttillskott {ditt barn, du} ätit under de senaste månaderna, välj i rullistan hur ofta {ditt barn, du} brukar äta dessa.

	Under de senaste månaderna [frq]
Multivitaminer eller mineraler [mult]	❍ Varje dag [1]<br>❍ Några gånger i veckan [2]<br>❍ Några gånger i månaden [3]<br>❍ I perioder [4]<br>❍ Mer sällan eller aldrig [5]
A-vitamin [vita]	❍ Varje dag [1]<br>❍ Några gånger i veckan [2]<br>❍ Några gånger i månaden [3]<br>❍ I perioder [4]<br>❍ Mer sällan eller aldrig [5]

B-vitaminer [vitb]	❍ Varje dag [1]<br>❍ Några gånger i veckan [2]<br>❍ Några gånger i månaden [3]<br>❍ I perioder [4]<br>❍ Mer sällan eller aldrig [5]
C-vitamin [vitc]	❍ Varje dag [1]<br>❍ Några gånger i veckan [2]<br>❍ Några gånger i månaden [3]<br>❍ I perioder [4]<br>❍ Mer sällan eller aldrig [5]
D-vitamin [vitd]	❍ Varje dag [1]<br>❍ Några gånger i veckan [2]<br>❍ Några gånger i månaden [3]<br>❍ I perioder [4]<br>❍ Mer sällan eller aldrig [5]
E-vitamin [vite]	❍ Varje dag [1]<br>❍ Några gånger i veckan [2]<br>❍ Några gånger i månaden [3]<br>❍ I perioder [4]<br>❍ Mer sällan eller aldrig [5]
Folsyra [foli]	❍ Varje dag [1]<br>❍ Några gånger i veckan [2]<br>❍ Några gånger i månaden [3]<br>❍ I perioder [4]<br>❍ Mer sällan eller aldrig [5]
Järn [iron]	❍ Varje dag [1]<br>❍ Några gånger i veckan [2]<br>❍ Några gånger i månaden [3]<br>❍ I perioder [4]<br>❍ Mer sällan eller aldrig [5]
Kalcium [calc]	❍ Varje dag [1]<br>❍ Några gånger i veckan [2]<br>❍ Några gånger i månaden [3]<br>❍ I perioder [4]<br>❍ Mer sällan eller aldrig [5]

❖ **M. VILLKORET AVSLUTAS**

Fysisk aktivitet

Följande frågor avser {ditt barns, din} fysiska aktivitet under de senaste månaderna.

Allmän aktivitet

[phy_act]

PA10. Hur mycket brukar {ditt barn, du} röra på {sig, dig}? {Mitt barn, Jag} rör {sig, mig}:

❍ Ganska lite [1]
❍ En del men aldrig så att {hon/han, jag} blir andfådd och svettig [2]
❍ Så att {hon/han, jag} blir svettig och andfådd någon gång i veckan [3]
❍ Så att {hon/han, jag} blir svettig och andfådd flera gånger i veckan [4]
❍ Så att {hon/han, jag} blir svettig och andfådd varje dag eller nästan varje dag [5]

❍ Vet ej / vill ej svara [998]

❖ **Z. OM ÅLDER < 15 VISA NEDAN FRÅGOR**

Om ålder < 11 och enkät besvaras av förälder, visa PA20
Om ålder >= 11 och enkät besvaras av barn, visa PA20

[phy_act_dur]

PA20. Hur mycket brukar {ditt barn, du} vara ute varje dag{; inklusive lek ute på dagis eller hos dagbarnvårdare; inklusive på rasterna i skolan; på rasterna och på fritiden}?

Måndag till Fredag [weekday]	Lördag och Söndag [weekend]
❍ Mindre än 1 timme per dag [1]	❍ Mindre än 1 timme per dag [1]
❍ 1 upp till 2 timmar per dag [2]	❍ 1 upp till 2 timmar per dag [2]

Måndag till Fredag [weekday]	Lördag och Söndag [weekend]
❍ 2 upp till 4 timmar per dag [3]<br>❍ 4 upp till 6 timmar per dag [4]<br>❍ 6 timmar eller mer per dag [5]	❍ 2 upp till 4 timmar per dag [3]<br>❍ 4 upp till 6 timmar per dag [4]<br>6 timmar eller mer per dag [5]

Om ålder < 11, visa PA30.1

[phy_act_intens]

PA30.1. Hur mycket rör ditt barn på sig när hon/han är ute?

❍ Är oftast stillsamt {, t.ex. sitter i sandlådan} [1]
❍ Rör sig oftast måttligt [2]
❍ Rör sig oftast mycket {, t.ex. springer, hoppar, cyklar, klättrar eller spelar boll} [3]

❍ Vet ej / vill ej svara [998]

Om ålder < 11 och besvaras av förälder, visa PA30.2
Om ålder >= 11 och besvaras av barn, visa PA30.2

[phy_act_intens]

PA30.2. Hur mycket rör {ditt barn på sig, du på dig} när {hon/han, du} är ute?

❍ Lite [1]
❍ Medel [2]
❍ Mycket [3]

❍ Vet ej / vill ej svara [998]

- **Z. VILLKORET AVSLUTAS**
- **U1. OM ÅLDER < 11 ELLER OM ÅLDER MELLAN 11 OCH 14 OCH BESVARAS AV BARN VISA NEDAN FRÅGOR**

Transport till skola / dagis / sysselsättning

[phy_tran_type]

PA40. Hur tar {sig ditt barn, du dig} oftast till och från {dagis eller dagbarnvårdare, skolan}? (Markera alla aktuella)

- ❑ Åker bil [car]
- ❑ Åker buss eller tåg [bus]
- ❑ {< 6 år: Får skjuts i vagn eller på cykel} [lif]
- ❑ Går eller cyklar själv [wal]
- ❑ {>= 15 år: Åker moped; >= 16: Åker moped, skoter eller motorcykel} [mot]
- ❑ På annat sätt [oth]

❍ Inte aktuellt för {mitt barn, mig} [996]
❍ Vet ej / vill ej svara [998]

Om PA40 är "går eller cyklar själv", visa PA50

[phy_tran]

PA50. Hur ofta går eller cyklar {ditt barn, du} till och från skolan?

Dagar per vecka [frq]	Tid enkel väg till {dagis, skola} per dag [dur]
❍ 1 [1]<br>❍ 2 [2]<br>❍ 3 [3]<br>❍ 4 [4]<br>❍ 5 [5]	❍ 0 - 9 minuter [1]<br>❍ 10 - 19 minuter [2]<br>❍ 20 - 29 minuter [3]<br>❍ 30 minuter eller mer [4]

- ❖ **U1. VILLKORET AVSLUTAS**
- ❖ **U2. OM ÅLDER >= 15 VISA NEDAN FRÅGOR**

[phy3_occtran]

Hur tar du dig vanligtvis till och från skola, jobb eller annan daglig sysselsättning?

❑ Promenerar [wal]
❑ Åker buss, tåg, tunnelbana eller båt

❑ Cyklar [bic]
❑ Åker motorcykel, moped eller skoter [mc]
❑ Åker bil eller taxi [car]
[bus]
❑ På annat sätt [oth]
❍ Gäller inte mig [996]
❍ Vet ej / vill ej svara [998]

❖ U3. FÖR VARJE SVAR MARKERAT I OVAN FRÅGA, VISA MOTSVARANDE NEDAN FRÅGA

[phy_tran_type]

Hur ofta tar du dig till din dagliga sysselsättning på följande sätt och hur lång tid tar det?

	Antal dagar i veckan [frq]	Tid enkel väg per dag [dur]
Promenerar [wal]	❍ 1 [1]<br>❍ 2 [2]<br>❍ 3 [3]<br>❍ 4 [4]<br>❍ 5 [5]<br>❍ 6 [6]<br>❍ 7 [7]	❍ Mindre än 15 minuter [1]<br>❍ 15 - 29 minuter [2]<br>❍ 30 - 44 minuter [3]<br>❍ 45 - 59 minuter [4]<br>❍ 1 - 2 timmar [5]<br>❍ Mer än 2 timmar [6]
Cyklar [bic]	❍ 1 [1]<br>❍ 2 [2]<br>❍ 3 [3]<br>❍ 4 [4]<br>❍ 5 [5]<br>❍ 6 [6]<br>❍ 7 [7]	❍ Mindre än 15 minuter [1]<br>❍ 15 - 29 minuter [2]<br>❍ 30 - 44 minuter [3]<br>❍ 45 - 59 minuter [4]<br>❍ 1 - 2 timmar [5]<br>❍ Mer än 2 timmar [6]
Åker motorcykel, moped eller skoter [mc]	❍ 1 [1]<br>❍ 2 [2]<br>❍ 3 [3]<br>❍ 4 [4]<br>❍ 5 [5]<br>❍ 6 [6]<br>❍ 7 [7]	❍ Mindre än 15 minuter [1]<br>❍ 15 - 29 minuter [2]<br>❍ 30 - 44 minuter [3]<br>❍ 45 - 59 minuter [4]<br>❍ 1 - 2 timmar [5]<br>❍ Mer än 2 timmar [6]
Åker bil eller taxi [car]	❍ 1 [1]<br>❍ 2 [2]<br>❍ 3 [3]<br>❍ 4 [4]<br>❍ 5 [5]<br>❍ 6 [6]<br>❍ 7 [7]	❍ Mindre än 15 minuter [1]<br>❍ 15 - 29 minuter [2]<br>❍ 30 - 44 minuter [3]<br>❍ 45 - 59 minuter [4]<br>❍ 1 - 2 timmar [5]<br>❍ Mer än 2 timmar [6]
Åker buss, tåg, tunnelbana eller båt [bus]	❍ 1 [1]<br>❍ 2 [2]<br>❍ 3 [3]<br>❍ 4 [4]	❍ Mindre än 15 minuter [1]<br>❍ 15 - 29 minuter [2]<br>❍ 30 - 44 minuter [3]<br>❍ 45 - 59 minuter [4]

	Antal dagar i veckan [frq]	Tid enkel väg per dag [dur]
	❍ 5 [5]<br>❍ 6 [6]<br>❍ 7 [7]	❍ 1 - 2 timmar [5]<br>❍ Mer än 2 timmar [6]
På det andra sättet [oth]	❍ 1 [1]<br>❍ 2 [2]<br>❍ 3 [3]<br>❍ 4 [4]<br>❍ 5 [5]<br>❍ 6 [6]<br>❍ 7 [7]	❍ Mindre än 15 minuter [1]<br>❍ 15 - 29 minuter [2]<br>❍ 30 - 44 minuter [3]<br>❍ 45 - 59 minuter [4]<br>❍ 1 - 2 timmar [5]<br>❍ Mer än 2 timmar [6]

❖ **U2 OCH U3. VILLKOREN AVSLUTAS**

Fritidsaktiviteter

❖ **A. OM ÅLDER < 11 ELLER (ÅLDER < 15 OCH ENKÄT BESVARAS AV BARNEN) VISA NEDAN FRÅGOR**

[phy_leisure_tv_1]

PA90. Hur mycket brukar {ditt barn, du} vanligtvis titta på TV eller DVD/video?

Måndag till Fredag [weekday]	Lördag och Söndag [weekend]
❍ Sällan eller inte alls [1]<br>❍ Mindre än 1 timme per dag [2]<br>❍ 1 upp till 2 timmar per dag [3]<br>❍ 2 upp till 4 timmar per dag [4]<br>❍ 4 upp till 6 timmar per dag [5]<br>❍ 6 timmar eller mer per dag [6]	❍ Sällan eller inte alls [1]<br>❍ Mindre än 1 timme per dag [2]<br>❍ 1 upp till 2 timmar per dag [3]<br>❍ 2 upp till 4 timmar per dag [4]<br>❍ 4 upp till 6 timmar per dag [5]<br>❍ 6 timmar eller mer per dag [6]

[phy_leisure_com_1]

PA100. Hur mycket brukar {ditt barn, du} vanligtvis spela TV-spel eller sitta vid dator per dag{, t.ex. för att surfa, chatta eller spela dataspel}?

Måndag till Fredag [weekday]	Lördag och Söndag [weekend]
❍ Sällan eller inte alls [1]	❍ Sällan eller inte alls [1]
❍ Mindre än 1 timme per dag [2]	❍ Mindre än 1 timme per dag [2]
❍ 1 upp till 2 timmar per dag [3]	❍ 1 upp till 2 timmar per dag [3]
❍ 2 upp till 4 timmar per dag [4]	❍ 2 upp till 4 timmar per dag [4]
❍ 4 upp till 6 timmar per dag [5]	❍ 4 upp till 6 timmar per dag [5]
❍ 6 timmar eller mer per dag [6]	❍ 6 timmar eller mer per dag [6]

- **A. VILLKORET AVSLUTAS**
- **B. OM ÅLDER < 6 VISA NEDAN FRÅGOR**

[phy_leisure_sports]

PA110. Utöver ditt barn regelbundet någon fysiskt aktiv fritidsaktivitet, t.ex. gymnastik, dans eller simning?

❍ Ja [1]
❍ Nej [2]
❍ Vet ej / vill ej svara [998]

Om PA110 är "ja", visa PA120

[phy2_leisure_sports_frq]

PA120. Hur ofta utövar hon/han sina fritidsaktiviteter?

❍ 3 gånger per vecka eller mer [5]
❍ 2 gånger per vecka [4]
❍ 1 gång per vecka [3]
❍ 1 – 3 gånger per månad [2]
❍ Vet ej / vill ej svara [998]

- **B. VILLKORET AVSLUTAS**
- **C. OM ÅLDER >= 15 VISA NEDAN FRÅGOR**

[phy3_leiact]

Vilka aktiviteter ägnar du dig åt minst en gång i veckan under din fritid?

❑ Sitter och tittar på TV, DVD m.m. [tv]
❑ Sitter vid datorn, läser e-brev, spelar datorspel, playstation, Xbox m.m. [com]
❑ Sitter och läser, skriver, syr m.m. [rea]
❑ Spelar musikinstrument eller fysiskt aktiva dator- och TV-spel (t.ex. Wii) [phy]
❑ Hushållsarbetar, städar, tvättar, tar hand om barn, trädgårdsarbetar m.m. [hom]
❑ Handlar eller uträttar ärenden [sho]
❑ Arbetar extra mot betalning [wor]
❑ Går ut och dansar (t.ex. disco eller dansband) [dan]
❑ Promenerar (ej som transport till daglig sysselsättning), stavgång, rastar hunden [wal]
❑ Cyklar (ej som transport till daglig sysselsättning) [bic]
❍ Inget av dessa [996]
❍ Vet ej / vill ej svara [998]

❖ **D. FÖR VARJE SVAR SOM MARKERATS I OVAN FRÅGA, VISA MOTSVARANDE I NEDAN FRÅGA**

[phy3_leiact1_type]

Hur ofta ägnar du dig åt följande fritidsaktiviteter och hur lång tid gör du det per dag?

	Antal dagar i veckan [frq]	Total tid per dag [dur]
Sitter och tittar på TV, DVD m.m. [tv]	❍ 1 [1]<br>❍ 2 [2]<br>❍ 3 [3]<br>❍ 4 [4]<br>❍ 5 [5]<br>❍ 6 [6]<br>❍ 7 [7]	❍ Mindre än 30 minuter [1]<br>❍ 30 - 59 minuter [2]<br>❍ 1 - 2 timmar [3]<br>❍ 3 - 4 timmar [4]<br>❍ 5 - 8 timmar [5]<br>Mer än 8 timmar [6]
Sitter vid datorn, läser e-brev, spelar datorspel, playstation, Xbox m.m. [com]	❍ 1 [1]<br>❍ 2 [2]<br>❍ 3 [3]<br>❍ 4 [4]<br>❍ 5 [5]<br>❍ 6 [6]<br>❍ 7 [7]	❍ Mindre än 30 minuter [1]<br>❍ 30 - 59 minuter [2]<br>❍ 1 - 2 timmar [3]<br>❍ 3 - 4 timmar [4]

	Antal dagar i veckan [frq]	Total tid per dag [dur]
		❍ 5 - 8 timmar [5]<br><br>Mer än 8 timmar [6]
Sitter och läser, skriver, syr m.m. [rea]	❍ 1 [1]<br>❍ 2 [2]<br>❍ 3 [3]<br>❍ 4 [4]<br>❍ 5 [5]<br>❍ 6 [6]<br>❍ 7 [7]	❍ Mindre än 30 minuter [1]<br><br>❍ 30 - 59 minuter [2]<br><br>❍ 1 - 2 timmar [3]<br><br>❍ 3 - 4 timmar [4]<br><br>❍ 5 - 8 timmar [5]<br><br>Mer än 8 timmar [6]
Spelar musikinstrument eller spelar fysiskt aktiva dator- och TV-spel (t.ex. Wii) [phy]	❍ 1 [1]<br>❍ 2 [2]<br>❍ 3 [3]<br>❍ 4 [4]<br>❍ 5 [5]<br>❍ 6 [6]<br>❍ 7 [7]	❍ Mindre än 30 minuter [1]<br><br>❍ 30 - 59 minuter [2]<br><br>❍ 1 - 2 timmar [3]<br><br>❍ 3 - 4 timmar [4]<br><br>❍ 5 - 8 timmar [5]<br><br>Mer än 8 timmar [6]
Hushållsarbetar, städar, tvättar, tar hand om barn, trädgårdsarbetar m.m. [hom]	❍ 1 [1]<br>❍ 2 [2]<br>❍ 3 [3]<br>❍ 4 [4]<br>❍ 5 [5]<br>❍ 6 [6]<br>❍ 7 [7]	❍ Mindre än 30 minuter [1]<br><br>❍ 30 - 59 minuter [2]<br><br>❍ 1 - 2 timmar [3]<br><br>❍ 3 - 4 timmar [4]<br><br>❍ 5 - 8 timmar [5]<br><br>Mer än 8 timmar [6]
Handlar eller uträttar ärenden [sho]	❍ 1 [1]<br>❍ 2 [2]<br>❍ 3 [3]<br>❍ 4 [4]<br>❍ 5 [5]<br>❍ 6 [6]<br>❍ 7 [7]	❍ Mindre än 30 minuter [1]<br><br>❍ 30 - 59 minuter [2]<br><br>❍ 1 - 2 timmar [3]<br><br>❍ 3 - 4 timmar [4]<br><br>❍ 5 - 8 timmar [5]

	Antal dagar i veckan [frq]	Total tid per dag [dur]
		Mer än 8 timmar [6]
Arbetar extra mot betalning [wor]	❍ 1 [1]<br>❍ 2 [2]<br>❍ 3 [3]<br>❍ 4 [4]<br>❍ 5 [5]<br>❍ 6 [6]<br>❍ 7 [7]	❍ Mindre än 30 minuter [1]<br>❍ 30 - 59 minuter [2]<br>❍ 1 - 2 timmar [3]<br>❍ 3 - 4 timmar [4]<br>❍ 5 - 8 timmar [5]<br>Mer än 8 timmar [6]

[phy3_leiact2_type]

	Antal dagar i veckan	Total tid per dag
Går ut och dansar (t.ex. disco eller dansband) [dan]	❍ 1 [1]<br>❍ 2 [2]<br>❍ 3 [3]<br>❍ 4 [4]<br>❍ 5 [5]<br>❍ 6 [6]<br>❍ 7 [7]	❍ Mindre än 30 minuter [1]<br>❍ 30 - 59 minuter [2]<br>❍ 1 - 2 timmar [3]<br>❍ 3 - 4 timmar [4]<br>❍ 5 - 8 timmar [5]<br>Mer än 8 timmar [6]
Promenerar (ej som transport till daglig sysselsättning), stavgång, rastar hunden [wal]	❍ 1 [1]<br>❍ 2 [2]<br>❍ 3 [3]<br>❍ 4 [4]<br>❍ 5 [5]<br>❍ 6 [6]<br>❍ 7 [7]	❍ Mindre än 30 minuter [1]<br>❍ 30 - 59 minuter [2]<br>❍ 1 - 2 timmar [3]<br>❍ 3 - 4 timmar [4]<br>❍ 5 - 8 timmar [5]<br>Mer än 8 timmar [6]
Cyklar (ej som transport till daglig sysselsättning) [bic]	❍ 1 [1]<br>❍ 2 [2]<br>❍ 3 [3]	❍ Mindre än 30

	Antal dagar i veckan	Total tid per dag
	❍ 4 [4]<br>❍ 5 [5]<br>❍ 6 [6]<br>❍ 7 [7]	minuter [1]<br>❍ 30 - 59 minuter [2]<br>❍ 1 - 2 timmar [3]<br>❍ 3 - 4 timmar [4]<br>❍ 5 - 8 timmar [5]<br>Mer än 8 timmar [6]

❖ **D. VILLKORET AVSLUTAS**

Om ”arbetar extra mot betalning”, visa nedan fråga

[phy3_leiact_work_intens_1]

Du nämnde att du har avlönat extraarbete. Vilken aktivitetsnivå har du vanligtvis på ditt arbete?

Sitter mest [1]	[2]	Står och går mest [3]	[4]	Tungt kroppsarbete [5]
☐	☐	☐	☐	☐

❖ **C. VILLKORET AVSLUTAS**

Skolidrott

❖ **E. OM ÅLDER >= 6 VISA NEDAN FRÅGOR. BESVARAS ENBART AV BARN FRÅN 11 ÅR**

[phy2_school_sports]

PA70. Hur många lektioner idrott {har ditt barn, är du med på} i skolan?

❍ Mindre än 1 lektion per vecka [1]
❍ 1 lektion per vecka [2]
❍ 2 lektioner per vecka [3]
❍ 3 lektioner per vecka [4]
❍ 4 lektioner per vecka [5]
❍ 5 lektioner eller fler per vecka [6]
❍ {ålder >= 15: Inte aktuellt för mig} [996]
❍ Vet ej / vill ej svara [998]

Om PA70 är "1 lektion per vecka" eller mer, visa PA80

[phy2_school_sports_intens]

PA80. Hur {aktivt är ditt barn, aktiv är du} på idrottslektionerna i skolan?

❍ Lite [1]
❍ Medel [2]
❍ Mycket [3]
❍ Vet ej / vill ej svara [998]

❖ **E. VILLKORET AVSLUTAS**

Sportaktiviteter

❖ **F. OM ÅLDER >= 6 VISA NEDAN FRÅGOR**

[phy2_sport]

PA150. Tränar eller sportar {ditt barn, du} regelbundet {, t.ex. gymnasik, dans eller fotboll}?

❍ Ja [1] ❍ Vet ej / vill ej svara [998]
❍ Nej [2]

- **G. OM PA150 ÄR "JA" OCH BESVARAS AV FÖRÄLDER ELLER AV BARN >= 15 ÅR, VISA NEDAN FRÅGOR**

[phy3_sports]

Vilka träningsformer eller sporter ägnar {sig ditt barn, du dig} vanligtvis åt? Om inte just {ditt barns, din} aktivitet finns med i listan, välj en liknande aktivitet.

❑ Gympapass eller aerobics [aer]
❑ Styrketräning {ålder >= 15: med vikter} [gym]
❑ Jogging, löpning eller orienteering [jog]
❑ Friidrott (t.ex. höjdhopp), längdhopp eller tresteg [ath]
❑ {ålder >= 11: Spinning eller cykling i tuff terräng, Cykling i tuff terräng} [spi]
❑ Simning [swi]
❑ Bollspel i lag (t.ex. fotboll, basket, volleyboll eller innebandy) [bal]
❑ Golf [gol]
❑ {ålder >= 18: Danskurs eller tävlingsdans, ålder >= 15: Dans (t.ex. pardans, balett, jazz eller street), ålder < 15: Dans (t.ex. balett, jazz, street)} [dan]
❑ Ridning [hor]
❑ Skridskoåkning, hockey eller bandy [hoc]
❑ Skidåkning utför eller längd [ski]
❑ Kampsport (t.ex. judo eller karate) [mar]
❑ Boxning eller brottning [box]
❑ Tennis, badminton eller bordtennis [ten]
❑ Squash [squ]
❑ Segling, surfing, kanot eller rodd [sai]
❑ Motorsport (t.ex. motorcross) [mot]
❑ Klättring [cli]
❑ Yoga, pilates eller Tai chi [yog]
❑ Annan träningsform eller sport (ange vilken) [oth]___________ [oth_other]
❑
❍ Ingen av dessa [996]
❍ Vet ej / vill ej svara [998]

- **H. FÖR VARJE SVAR PÅ OVAN FRÅGA, VISA MOTSVARANDE I NEDAN FRÅGA**

[phy3_sport_type]

Hur ofta ägnar {sig ditt barn, du dig} åt följande sporter och hur lång tid gör {ditt barn, du} det per gång?

	Antal gånger [frq]	Tid per gång [dur]
Gympapass eller aerobics [aer]	❍ 1 - 3 gånger per månad [1]<br>❍ 1 gång per vecka [2]<br>❍ 2 - 3 gånger per vecka [3]<br>❍ 4 - 5 gånger per vecka [4]<br>❍ 6 - 7 gånger per vecka [5]	❍ Mindre än 30 minuter [1]<br>❍ 30 - 59 minuter [2]<br>❍ 60 - 119 minuter [3]<br>❍ 2 - 4 timmar [4]<br>❍ Mer än 4 timmar [5]
Styrketräning {ålder >=15: med vikter} [gym]	❍ 1 - 3 gånger per månad [1]<br>❍ 1 gång per vecka [2]<br>❍ 2 - 3 gånger per vecka [3]<br>❍ 4 - 5 gånger per vecka [4]<br>❍ 6 - 7 gånger per vecka [5]	❍ Mindre än 30 minuter [1]<br>❍ 30 - 59 minuter [2]<br>❍ 60 - 119 minuter [3]<br>❍ 2 - 4 timmar [4]<br>❍ Mer än 4 timmar [5]
Jogging, löpning eller orientering [jog]	❍ 1 - 3 gånger per månad [1]<br>❍ 1 gång per vecka [2]<br>❍ 2 - 3 gånger per vecka [3]<br>❍ 4 - 5 gånger per vecka [4]<br>❍ 6 - 7 gånger per vecka [5]	❍ Mindre än 30 minuter [1]<br>❍ 30 - 59 minuter [2]<br>❍ 60 - 119 minuter [3]<br>❍ 2 - 4 timmar [4]<br>❍ Mer än 4 timmar [5]
Friidrott (t.ex. höjdhopp), längdhopp eller tresteg [ath]	❍ 1 - 3 gånger per månad [1]<br>❍ 1 gång per vecka [2]<br>❍ 2 - 3 gånger per vecka [3]<br>❍ 4 - 5 gånger per vecka [4]<br>❍ 6 - 7 gånger per vecka [5]	❍ Mindre än 30 minuter [1]<br>❍ 30 - 59 minuter [2]<br>❍ 60 - 119 minuter [3]<br>❍ 2 - 4 timmar [4]<br>❍ Mer än 4 timmar [5]
{ålder >= 11: Spinning eller cykling i tuff terräng, ålder < 11: Cykling i tuff terräng} [spi]	❍ 1 - 3 gånger per månad [1]<br>❍ 1 gång per vecka [2]<br>❍ 2 - 3 gånger per vecka [3]<br>❍ 4 - 5 gånger per vecka [4]<br>❍ 6 - 7 gånger per vecka [5]	❍ Mindre än 30 minuter [1]<br>❍ 30 - 59 minuter [2]<br>❍ 60 - 119 minuter [3]<br>❍ 2 - 4 timmar [4]<br>❍ Mer än 4 timmar [5]
Simning [swi]	❍ 1 - 3 gånger per månad [1]<br>❍ 1 gång per vecka [2]<br>❍ 2 - 3 gånger per vecka [3]	❍ Mindre än 30 minuter [1]<br>❍ 30 - 59 minuter [2]<br>❍ 60 - 119 minuter [3]<br>❍ 2 - 4 timmar [4]

	Antal gånger [frq]	Tid per gång [dur]
	❍ 4 - 5 gånger per vecka [4]<br>❍ 6 - 7 gånger per vecka [5]	❍ Mer än 4 timmar [5]
Bollspel i lag (t.ex. fotboll, basket, volleyboll eller innebandy) [bal]	❍ 1 - 3 gånger per månad [1]<br>❍ 1 gång per vecka [2]<br>❍ 2 - 3 gånger per vecka [3]<br>❍ 4 - 5 gånger per vecka [4]<br>❍ 6 - 7 gånger per vecka [5]	❍ Mindre än 30 minuter [1]<br>❍ 30 - 59 minuter [2]<br>❍ 60 - 119 minuter [3]<br>❍ 2 - 4 timmar [4]<br>❍ Mer än 4 timmar [5]
Golf [gol]	❍ 1 - 3 gånger per månad [1]<br>❍ 1 gång per vecka [2]<br>❍ 2 - 3 gånger per vecka [3]<br>❍ 4 - 5 gånger per vecka [4]<br>❍ 6 - 7 gånger per vecka [5]	❍ Mindre än 30 minuter [1]<br>❍ 30 - 59 minuter [2]<br>❍ 60 - 119 minuter [3]<br>❍ 2 - 4 timmar [4]<br>❍ Mer än 4 timmar [5]
{ålder >= 18: Danskurs eller tävlingsdans, ålder >= 15: Dans (t.ex. pardans, balett, jazz eller street), ålder < 15: Dans (t.ex. balett, jazz, street)} [dan]	❍ 1 - 3 gånger per månad [1]<br>❍ 1 gång per vecka [2]<br>❍ 2 - 3 gånger per vecka [3]<br>❍ 4 - 5 gånger per vecka [4]<br>❍ 6 - 7 gånger per vecka [5]	❍ Mindre än 30 minuter [1]<br>❍ 30 - 59 minuter [2]<br>❍ 60 - 119 minuter [3]<br>❍ 2 - 4 timmar [4]<br>❍ Mer än 4 timmar [5]
Ridning [hor]	❍ 1 - 3 gånger per månad [1]<br>❍ 1 gång per vecka [2]<br>❍ 2 - 3 gånger per vecka [3]<br>❍ 4 - 5 gånger per vecka [4]<br>❍ 6 - 7 gånger per vecka [5]	❍ Mindre än 30 minuter [1]<br>❍ 30 - 59 minuter [2]<br>❍ 60 - 119 minuter [3]<br>❍ 2 - 4 timmar [4]<br>❍ Mer än 4 timmar [5]
Skridskoåkning, hockey eller bandy [hoc]	❍ 1 - 3 gånger per månad [1]<br>❍ 1 gång per vecka [2]<br>❍ 2 - 3 gånger per vecka [3]<br>❍ 4 - 5 gånger per vecka [4]<br>❍ 6 - 7 gånger per vecka [5]	❍ Mindre än 30 minuter [1]<br>❍ 30 - 59 minuter [2]<br>❍ 60 - 119 minuter [3]<br>❍ 2 - 4 timmar [4]<br>❍ Mer än 4 timmar [5]

	Antal gånger [frq]	Tid per gång [dur]
Skidåkning utför eller längd [ski]	❍ 1 - 3 gånger per månad [1]<br>❍ 1 gång per vecka [2]<br>❍ 2 - 3 gånger per vecka [3]<br>❍ 4 - 5 gånger per vecka [4]<br>❍ 6 - 7 gånger per vecka [5]	❍ Mindre än 30 minuter [1]<br>❍ 30 - 59 minuter [2]<br>❍ 60 - 119 minuter [3]<br>❍ 2 - 4 timmar [4]<br>❍ Mer än 4 timmar [5]
Kampsport (t.ex. judo eller karate) [mar]	❍ 1 - 3 gånger per månad [1]<br>❍ 1 gång per vecka [2]<br>❍ 2 - 3 gånger per vecka [3]<br>❍ 4 - 5 gånger per vecka [4]<br>❍ 6 - 7 gånger per vecka [5]	❍ Mindre än 30 minuter [1]<br>❍ 30 - 59 minuter [2]<br>❍ 60 - 119 minuter [3]<br>❍ 2 - 4 timmar [4]<br>❍ Mer än 4 timmar [5]
Boxning eller brottning [box]	❍ 1 - 3 gånger per månad [1]<br>❍ 1 gång per vecka [2]<br>❍ 2 - 3 gånger per vecka [3]<br>❍ 4 - 5 gånger per vecka [4]<br>❍ 6 - 7 gånger per vecka [5]	❍ Mindre än 30 minuter [1]<br>❍ 30 - 59 minuter [2]<br>❍ 60 - 119 minuter [3]<br>❍ 2 - 4 timmar [4]<br>❍ Mer än 4 timmar [5]
Tennis, badminton eller bordtennis [ten]	❍ 1 - 3 gånger per månad [1]<br>❍ 1 gång per vecka [2]<br>❍ 2 - 3 gånger per vecka [3]<br>❍ 4 - 5 gånger per vecka [4]<br>❍ 6 - 7 gånger per vecka [5]	❍ Mindre än 30 minuter [1]<br>❍ 30 - 59 minuter [2]<br>❍ 60 - 119 minuter [3]<br>❍ 2 - 4 timmar [4]<br>❍ Mer än 4 timmar [5]
Squash [squ]	❍ 1 - 3 gånger per månad [1]<br>❍ 1 gång per vecka [2]<br>❍ 2 - 3 gånger per vecka [3]<br>❍ 4 - 5 gånger per vecka [4]<br>❍ 6 - 7 gånger per vecka [5]	❍ Mindre än 30 minuter [1]<br>❍ 30 - 59 minuter [2]<br>❍ 60 - 119 minuter [3]<br>❍ 2 - 4 timmar [4]<br>❍ Mer än 4 timmar [5]
Segling, surfing, kanot eller rodd [sai]	❍ 1 - 3 gånger per månad [1]<br>❍ 1 gång per vecka [2]<br>❍ 2 - 3 gånger per vecka [3]	❍ Mindre än 30 minuter [1]<br>❍ 30 - 59 minuter [2]<br>❍ 60 - 119 minuter [3]<br>❍ 2 - 4 timmar [4]

	Antal gånger [frq]	Tid per gång [dur]
	❍ 4 - 5 gånger per vecka [4]<br>❍ 6 - 7 gånger per vecka [5]	❍ Mer än 4 timmar [5]
Motorsport (t.ex. motorcross) [mot]	❍ 1 - 3 gånger per månad [1]<br>❍ 1 gång per vecka [2]<br>❍ 2 - 3 gånger per vecka [3]<br>❍ 4 - 5 gånger per vecka [4]<br>❍ 6 - 7 gånger per vecka [5]	❍ Mindre än 30 minuter [1]<br>❍ 30 - 59 minuter [2]<br>❍ 60 - 119 minuter [3]<br>❍ 2 - 4 timmar [4]<br>❍ Mer än 4 timmar [5]
Klättring [cli]	❍ 1 - 3 gånger per månad [1]<br>❍ 1 gång per vecka [2]<br>❍ 2 - 3 gånger per vecka [3]<br>❍ 4 - 5 gånger per vecka [4]<br>❍ 6 - 7 gånger per vecka [5]	❍ Mindre än 30 minuter [1]<br>❍ 30 - 59 minuter [2]<br>❍ 60 - 119 minuter [3]<br>❍ 2 - 4 timmar [4]<br>❍ Mer än 4 timmar [5]
Yoga, pilates eller Tai chi [yog]	❍ 1 - 3 gånger per månad [1]<br>❍ 1 gång per vecka [2]<br>❍ 2 - 3 gånger per vecka [3]<br>❍ 4 - 5 gånger per vecka [4]<br>❍ 6 - 7 gånger per vecka [5]	❍ Mindre än 30 minuter [1]<br>❍ 30 - 59 minuter [2]<br>❍ 60 - 119 minuter [3]<br>❍ 2 - 4 timmar [4]<br>❍ Mer än 4 timmar [5]
Din andra träningsform eller sport (**systemet visar angiven sport**) [oth]	❍ 1 - 3 gånger per månad [1]<br>❍ 1 gång per vecka [2]<br>❍ 2 - 3 gånger per vecka [3]<br>❍ 4 - 5 gånger per vecka [4]<br>❍ 6 - 7 gånger per vecka [5]	❍ Mindre än 30 minuter [1]<br>❍ 30 - 59 minuter [2]<br>❍ 60 - 119 minuter [3]<br>❍ 2 - 4 timmar [4]<br>❍ Mer än 4 timmar [5]

- **H. VILLKORET AVSLUTAS**

- **I. OM EN ELLER FRÅGOR INOM VILLKORET C BESVARATS ”2 – 3 gånger per vecka” ELLER OFTARE VISA NEDAN FRÅGA**

[phy3_prof]

Tävlar {ditt barn, du} regelbundet inom någon sport?

❍ Ja [1]   ❍ Vet ej / vill ej svara [998]
❍ Nej [2]

❖ **F, G OCH I. VILLKOREN AVSLUTAS**

Sexualvanor

- **F. OM ÅLDER >= 15, ANVÄND MODULEN SEXUELLT BETEENDE FRÅN TEMAT DAGLIGA AKTIVITETER FÖR VUXNA**

Mobil och trådlöst

- **G. OM ÅLDER MELLAN 5 OCH 10, VISA NEDANSTÅENDE FRÅGOR**

[wir_mobown]

C.QL200. Har {ditt barn, du} mobiltelefon?

❍ Ja [1] ❍ Nej [2]
❍ Vet ej / vill ej svara [998]

Om "ja", visa C.QL210 och C.QL220

[wir_mobuse]

C.QL210. Hur använder {ditt barn sin, du din} mobiltelefon?

❑ {Mitt barn, jag} ringer och tar emot samtal [1]
❑ {Mitt barn, jag} spelar eller laddar musik [2]
❑ {Mitt barn, jag} spelar spel [3]
❑ {Mitt barn, jag} använder den för internet [4]
❑ {Mitt barn, jag} använder den för SMS / MMS [5]
❍ Vet ej / vill ej svara [998]

[wir_mobon]

C.QL220. Hur länge brukar mobiltelefonen vara på varje dag?

❍ 1 timme eller mindre [1]
❍ 2 till 3 timmar [2]
❍ 4 timmar eller mer [3]
❍ Vet ej / vill ej svara [998]

- **H. OM ÅLDER >= 11, ANVÄND MODULEN TRÅDLÖS UTRUSTNING I DAGLIGA AKTIVITETER FÖR VUXNA**

Husdjur

[pet_home]

C.IA220. Finns det några husdjur i {ditt barns, ditt} hem?

Ja
❑ Katt [cat]
❑ Hund [dog]
❑ Gnagare [rod]
❑ Fåglar [bir]
❑ Fiskar [fis]
❑ Annat (ange) [oth] ____________[oth_other]
❑
❍ Nej, inga husdjur [no]
❍ Vet ej / vill ej svara [998]

[pet_contact]

C.IA230. Har {ditt barn, du} regelbunden kontakt med något {, annat} djur?

❍ Ja [1]
❍ Nej [2]
❍ Vet ej / vill ej svara [998]

Om C.IA230 är "ja", visa C.IA240

[pet_contact_type]

C.IA240. Hur ofta har {ditt barn, du} kontakt med djuret eller djuren?

	Varje dag [1]	En gång i veckan [2]	En gång i månaden [3]	Aldrig [4]	Vet ej / vill ej svara [998]
Katt [cat]	☐	☐	☐	☐	☐
Hund [dog]	☐	☐	☐	☐	☐
Gnagare [rod]	☐	☐	☐	☐	☐
Häst [hor]	☐	☐	☐	☐	☐
Djur i en ladugård [cow]	☐	☐	☐	☐	☐
Annat djur [oth]	☐	☐	☐	☐	☐

Om C.IA240 är "annat", visa C.IA250

[pet_contact_other]

C.IA250. Vilket annat djur?

❖ **AK. OM C.IA220 ÄR "INGA HUSDJUR" I C.IA220, VISA NEDANSTÅENDE FRÅGOR**

[pet_getrid]

C.IA270. Har familjen vid något tillfälle gjort sig av med ett husdjur?

❍ Ja [1] ❍ Vet ej / vill ej svara [998]
❍ Nej [2]

Om C.IA270 är ”nej”, visa C.IA280

[pet_avoid]

C.IA280. Har familjen undvikit att skaffa husdjur?

❍ Ja [1] ❍ Vet ej / vill ej svara [998]
❍ Nej [2]

Om C.IA270 eller C.IA280 är ”ja”, visa C.IA285

[pet_why]

C.IA285. Varför {gjorde ni er av med husdjuret, har ni undvikit att skaffa husdjur}?

❍ På grund av allergi eller astma [1] ❍ Vet ej / vill ej svara [998]
❍ På grund av oro för att få allergi eller astma [2]
❍ Av annat skäl [3]

Om C.IA285 är ”på grund av...”, visa C.IA287

[pet_who]

C.IA287. {Vilka i familjen hade allergi eller astma, För vilka i familjen gällde oron}? (Markera alla aktuella)

❑ Barn [chi]
❑ Förälder [par]
❑ Annan person [oth]
❍ Vet ej / vill ej svara [998]

- **AK. VILLKORET AVSLUTAS**

Egenvård

Receptfria mediciner

- **R. BARN ANVÄNDER EN ANPASSAD VERSION AV SEKTIONEN FÖR RECEPTFRIA MEDICINER I TEMAT EGENVÅRD FÖR VUXNA**

Se vuxentemat. (Tilltal anpassat till barn och förälder)

- **R. VILLKORET AVSLUTAS**

Alternativmedicinska produkter, behandlingar, träningstekniker

- **S. BARN ANVÄNDER EN ANPASSAD VERSION AV SEKTIONEN FÖR PRODUKTER I TEMAT EGENVÅRD FÖR VUXNA. OM ÅLDER >= 15, ANVÄND ÄVEN SEKTIONERNA BEHANDLINGAR OCH TEKNIKER**

Se vuxenmodulen (Tilltal anpassat till barn och förälder)

Pubertet

❖ **I. OM ÅLDER >= 10, VISA NEDANSTÅENDE FRÅGOR (FÖRÄLDRAR SVARAR ENDAST VID 10-ÅRS ÅLDER)**

Följande frågor handlar om den utveckling och förändring som händer i kroppen under puberteten. Dessa förändringar brukar normalt ske hos unga människor i olika åldrar. Om du inte förstår en fråga eller om du inte kan svaret så kan du markera "Vet ej / vill ej svara".

[pub_height]

C.QL230. Skulle du säga att spurten i ditt {,barns} växande…

❍ har inte ännu börjat [1]
❍ har enbart börjat [2]
❍ har definitivt kommit igång [3]
❍ är avslutad [4]
❍ Vet ej / vill ej svara [998]

[pub_bodyhair]

C.QL240. Skulle du säga att {ditt barns, din} kroppsbehåring (armhåle- och könsbehåring)...

❍ har inte ännu börjat växa [1]
❍ har nätt och jämt börjat växa [2]
❍ har definitivt kommit igång [3]
❍ är färdigutväxt [4]
❍ Vet ej / vill ej svara [998]

[pub_skinchange]

C.QL245. Har du lagt märke till {att ditt barn fått,} hudförändringar, speciellt finnar?

❍ Nej [1]
❍ Ja, nätt och jämnt [2]
❍ Ja, definitivt [3]
❍ Hudförändringarna har upphört [4]
❍ Vet ej / vill ej svara [998]

Om kön är "man", visa C.QL260 och C.QL270

[pub_voice]

C.QL260. Har du lagt märke till att {ditt barns, din} röst blivit grövre?

❍ Nej [1]
❍ Ja, nätt och jämnt [2]
❍ Ja, definitivt [3]
❍ Röstförändringarna har upphört [4]

❍ Vet ej / vill ej svara [998]

[pub_facehair]

C.QL270. Har {ditt barn, du} börjat få skägg?

❍ Skäggväxten har ännu inte börjat [1]
❍ Skäggväxten har knappast börjat [2]
❍ Skäggväxten har definitivt börjat [3]
❍ Skäggväxten är nu regelbunden [4]
❍ Vet ej / vill ej svara [998]

Om kön är "kvinna", visa C.QL280, C.QL290 och C.QL300

[pub_breast]

C.QL280. Har du lagt märke till att {ditt barns, dina} bröst börjat växa?

❍ Nej [1]
❍ Ja, nätt och jämnt [2]
❍ Ja, definitivt [3]
❍ Brösttillväxten verkar vara avslutad [4]
❍ Vet ej / vill ej svara [998]

[pub_mens]

C.QL290. Har {ditt barn, du} börjat få menstruation?

❍ Ja [1]
❍ Nej [2]
❍ Vet ej / vill ej svara [998]

[pub_mens_age]

C.QL300. Hur många år och månader var {ditt barn, du} vid den första menstruationen?

❍ (År) [yrs]________ [yrs_other]
❍ (Månader) [mnt]______ [mnt_other]
❍ Vet ej / vill ej svara [998]

❖ **I. VILLKORET AVSLUTAS**

Kvinnans hälsa

- **J. OM ÅLDER >= 15, ANVÄND TEMAT KVINNANS HÄLSA FÖR VUXNA**

Tobak, alkohol och droganvändning

Rökvanor

- ❖ **K. OM ÅLDER ÄR FRÅN 0 TILL 10 VISA NEDAN FRÅGOR**

[smo_others]

SM170. Röker någon i ditt barns hushåll?

❍ Ja [1] ❍ Vet ej / vill ej svara [998]
❍ Nej [2]

Om "ja", visa SM180

[smo_others_amount]

SM180. Hur många cigaretter röks en vanlig dag i hushållet?

❍ (Antal) [nbr]____________ [nbr_other] ❍ Vet ej / vill ej svara [998]

[smo_place]

SM190. Var brukar {ni, man} röka?

❍ Utomhus för det mesta [1] ❍ Inomhus för det mesta [3]
❍ Ungefär lika mycket utomhus som inomhus [2] ❍ Vet ej / vill ej svara [998]

- ❖ **K. VILLKORET AVSLUTAS**
- ❖ **L. OM ÅLDER ÄR >= 15 OCH BESVARAS AV BARN, ANVÄND ÄVEN MODULEN OM RÖKNING I LEVNADSVANOR FÖR VUXNA**

Se vuxenmodulen

Snus

- **OM ÅLDER >= 15 OCH BESVARAS AV BARN, ANVÄND SNUSMODULEN I TEMAT LEVNADSVANOR FÖR VUXNA**

Se vuxenmodulen

Vänner

- **OM ÅLDER >= 15 OCH BESVARAS AV BARN, ANVÄND MODULEN FÖR VÄNNER I TEMAT LEVNADSVANOR FÖR VUXNA**

Se vuxenmodulen

Alkohol

❖ **M. OM ÅLDER ÄR FRÅN 11 TILL 14 OCH BESVARAS AV BARN, VISA NEDANSTÅENDE FRÅGOR**

[alc_sip]

C.AL10. Har du någonsin smakat alkohol?

❍ Ja [1] ❍ Vet ej / vill ej svara [998]
❍ Nej [2]

❖ **N. LÄMNA OM "JA" I C.AL10**

[alc_fulldrink_child]

C.AL20. Har du någonsin druckit en hel alkoholhaltig dryck?

❍ Ja [1] ❍ Vet ej / vill ej svara [998]
❍ Nej [2]

❖ **O. LÄMNA OM "JA" I C.AL20**

[alc_parent]

C.AL30. Har du någonsin druckit alkohol utan att dina föräldrar varit närvarande?

❍ Ja [1] ❍ Vet ej / vill ej svara [998]
❍ Nej [2]

[alc_24hdrinks]

C.AL40. Hur många alkoholhaltiga drinkar har du druckit som mest under en 24-timmarsperiod?

❍ (Antal) [nbr]____________ [nbr_other] ❍ Vet ej / vill ej svara [998]

[alc_intox]

C.AL50. Har du någonsin varit full eller berusad?

❍ Ja [1] ❍ Vet ej / vill ej svara [998]
❍ Nej [2]

Om "ja", visa C.AL60

[alc_intox_age]

C.AL60. Hur gammal var du första gången du var full eller berusad?

❍ (Ålder) [yrs]____________ [yrs_other] ❍ Vet ej / vill ej svara [998]

Följande två frågor handlar om ditt drickande under de senaste 12 månaderna.

[alc_12mnts_frq]

C.AL70. Hur ofta tar du en alkoholhaltig drink?

❍ Aldrig [1]
❍ En gång i månaden eller mindre [2]
❍ 2 till 3 gånger i månaden [3]
❍ 1 gång i veckan [4]
❍ 2 till 3 gånger i veckan [5]
❍ 4 eller fler gånger i veckan [6]
❍ Vet ej / vill ej svara [998]

[alc_12mnts_24hfrq]

C.AL80. Hur många alkoholhaltiga drinkar dricker du en typisk dag då du dricker?

❍ 1 till 2 [1] ❍ 5 till 6 [3] ❍ 10 eller fler [5]
❍ 3 till 4 [2] ❍ 7 till 9 [4] ❍ Vet ej / vill ej svara [998]

- **M. VILLKORET AVSLUTAS**
- **P. OM ÅLDER >= 15 ANVÄND ALKOHOLMODULEN I TEMAT LEVNADSVANOR FÖR VUXNA**

Se vuxenmodulen

Droganvändning

- **Q. OM ÅLDER ÄR >= 15 ANVÄND DROGMODULEN I TEMAT TOBAK, ALKOHOL OCH DROGANVÄNDNING FÖR VUXNA**

Se vuxenmodulen

Hälsohistoria

Tandhälsa

- **T. OM ÅLDER >= 5, ANVÄND TANDHÄLSOMODULEN I TEMAT MEDICINSK HISTORIA FÖR VUXNA**

Se vuxenmodulen. (Tilltal anpassat till barn och förälder)

Infektioner

- **A. FÖR ÅLDER >= 4 ANVÄND INFEKTIONSMODULEN I TEMAT MEDICINSK HISTORIA FÖR VUXNA**

Se vuxenmodulen

Sjukdomar

Om ålder < 5, visa C.DS10

[dis_1c]

C.DS10. {Har ditt barn eller har han/hon haft någon av följande sjukdomar, Har ditt barn haft någon av följande sjukdomar under det första levnadsåret}?

Ja
❑ Förkylning [cold]
❑ Öroninflammation [ear]
❑ Ögoninflammation (conjunctivitis) [conj]
❑ Halsfluss [tons]
❑ Falsk krupp [pseu]
❑ Astma [asth]
❑ Lunginflammation [pneu]
❑ Urinvägsinfektion eller njurbäckeninflammation [urin]
❑ Springmask (enterobiasis) [pinw]
❑ Eksem [ecze]
❑ Nässelutslag [hive]
❑ Allergi eller intolerans mot födoämne [food]
❑ Feberkramper [feve]
❑ Nedsatt immunförsvar [immu]
❑
❍ Nej, ingen av dessa [996]
❍ Vet ej / vill ej svara [998]

Om ålder > 1, visa C.DS15

[dis_2c]

C.DS20. Vilka av följande sjukdomar har ditt barn eller han/hon haft {under de senaste 12 månaderna, efter det första levnadsåret}?

❑ Förkylning [cold]
❑ Öroninflammation [ear]
❑ Ögoninflammation (conjunctivitis) [conj]
❑ Halsfluss [tons]
❑ {ålder < 10: Falsk krupp} [pseu]
❑ Astma [asth]
❑ Lunginflammation [pneu]
❑ Urinvägsinfektion eller njurbäckeninflammation [urin]
❑ Springmask (enterobiasis) [pinw]
❑ Eksem [ecze]
❑ Nässelutslag [hive]
❑ Allergi eller intolerans mot födoämne [food]
❑ {ålder < 10: Feberkramper} [feve]
❑ Nedsatt immunförsvar [immu]
❑ { >= 7: Migrän} [migr]
❑ Annan sjukdom (ange)[oth]
___[oth_other]
❑
❍ Ingen av dessa [996]
❍ Vet ej / vill ej svara [998]

❖ **A1. OM ENKÄT BESVARAS AV BARN, VISA NEDAN FRÅGOR**

Om ålder < 5, visa C.DS20

[dis_antibio_1styear]

C.DS22. Har ditt barn behandlats med antibiotika för förkylning{, under första levnadsåret}?

Ja

❍ En gång [1] ❍ Nej [no]
❍ 2 till 4 gånger [2] ❍ Vet ej / vill ej svara [998]
❍ 5 eller fler gånger [3]

Om ålder mellan 1 och 4, visa C.DS25

[dis_antibio_lastyears]

C.DS25. Har ditt barn behandlats med antibiotika för förkylning efter hans/hennes första levnadsår?

Ja

❍ En gång [1] ❍ Nej [no]
❍ 2 till 4 gånger [2] ❍ Vet ej / vill ej svara [998]
❍ 5 eller fler gånger [3]

Om ålder >= 1, visa C.DS27

[dis_antibio_lastyear]

C.DS27. Har ditt barn behandlats med antibiotika för förkylning under det senate året?

Ja

❍ En gång [1] ❍ Nej [no]
❍ 2 till 4 gånger [2] ❍ Vet ej / vill ej svara [998]
❍ 5 eller fler gånger [3]

Om ålder >= 5, visa C.DS30

[dis_breastfeed]

C.DS30. Hur länge fick ditt barn bröstmjölk (helt eller delvis)?

❍ Upp till 6 månader [1]
❍ 6 till 11 månader [2]
❍ 1 till 2 år [3]
❍ Mer än 2 år [4]
❍ Mitt barn fick inte bröstmjölk [5]
❍ Vet ej / vill ej svara [998]

[dis_expillness_1]

C.DS40. Hur uppskattar du ditt barns hälsa jämfört med andra barn i samma ålder?

	1	2	3	4	5	
Oftare sjuk	☐	☐	☐	☐	☐	Oftare frisk

- **A1. VILLKORET AVSLUTAS**
- **A2. OM ENKÄT BESVARAS AV FÖRÄLDEER, VISA NEDAN FRÅGOR**

Om ålder >= 10, visa C.DS16

[dis_2c_dysl]

C.DS46. Uppfattar du att ditt barn har läs- och skrivsvårigheter (dyslexi)?

❍ Ja [1]
❍ Nej [2]
❍ Vet ej / vill ej svara [998]

Om C.DS46 är ”ja”, visa C.DS47

[dis_2c_dysl_diag]

C.DS47. Har en logoped ställt diagnosen dyslexi?

❍ Ja [1]
❍ Nej [2]
❍ Vet ej / vill ej svara [998]

Om ålder >= 3, visa C.DS48

[dis_2c_dysp]

C.DS48. Uppfattar du att ditt barn har svårighet att tala, hitta ord eller att förstå tal?

❍ Ja [1] ❍ Vet ej / vill ej svara [998]
❍ Nej [2]

Om C.DS48 "ja", visa C.DS50 till C.DS53

[dis_2c_dysph]

C.DS50. Hur skulle du beskriva ditt barns språkliga färdigheter, i jämförelse med jämnåriga, att på sitt modersmål:

	Mycket svårt [1]	Ganska svårt [2]	Ungefär medel [3]	Ganska lätt [4]	Mycket lätt [5]
Uttala ord rätt (t.ex. så att spade inte uttalas pade, att ros inte uttalas jos eller att kam inte uttalas tam) [pre]	☐	☐	☐	☐	☐
Upprepa ord eller fraser såsom andra uttalat dem [rep]	☐	☐	☐	☐	☐
Hitta ord eller kunna namnge saker [nam]	☐	☐	☐	☐	☐
Formulera grammatiskt korrekta meningar [gra]	☐	☐	☐	☐	☐
Förstå vad andra sager [com]	☐	☐	☐	☐	☐
Lära sig nya ord [lea]	☐	☐	☐	☐	☐
Uttrycka sig med ett flytande tal [flu]	☐	☐	☐	☐	☐
{ålder >= 10: Uttrycka sig i skrift} [wri]	☐	☐	☐	☐	☐

[dis_2c_sylla]

C.DS51. Använde ditt barn stavelsejoller (da-da-da, ba-ba) före 1 års ålder?

❍ Ja [1] ❍ Vet ej / vill ej svara [998]
❍ Nej [2]

[dis_2c_slur]

C.DS52. Är ditt barns tal sluddrigt, otydligt eller svagt?

❍ Ja [1] ❍ Vet ej / vill ej svara [998]
❍ Nej [2]

[dis_2c_therapy]

C.DS53. Har ditt barn fått talterapi eller någon annan behandling för talsvårigheter?

❍ Ja [1] ❍ Vet ej / vill ej svara [998]
❍ Nej [2]

❖ **A2. VILLKORET AVSLUTAS**

Hälsokontroll

- **A. OM ÅLDER < 1 VISA NEDAN FRÅGOR**

[con_weight]

ED40. Hur mycket vägde du vid tidpunkten för förlossningen?

❍ (kg) [kg]____________ [kg_other] ❍ Vet ej / vill ej svara [998]

[con_checkup]

MH100. Hur många gånger har du varit på barnavårdscentralen med ditt barn?

❍ Aldrig [5] ❍ 6 till 10 gånger [2]
❍ 1 till 2 gånger [4] ❍ Mer än 10 gånger [1]
❍ 3 till 5 gånger [3] ❍ Vet ej / vill ej svara [998]

[con_card]

MH110. Vi vill nu ställa några frågor om ditt barns längd, vikt och huvudomfång. Denna information finns tillgänglig på ditt barns hälsokort. Har du kortet tillgängligt?

❍ Ja [1] ❍ Vet ej / vill ej svara [998]
❍ Nej [2]

Om MH110 är "ja" och för varje kontrolltillfälle, visa MH120 och MH130.

[con_card1_date]

MH120. Vilket datum var du på {den n:te}-kontrollen?

[con_card1]

MH130. Ange ditt barns längd, vikt och huvudomfång vid {den n:te} kontrollen.

Längd (cm) [length]	Vikt (gram) [weight]	Huvudomfång (cm) [head]
_______	_______	_______

- **A. VILLKORET AVSLUTAS**

Vaccination

[vac_program]

IM10. Har ditt barn fått vaccinationer enligt det svenska vaccinationsprogrammet?

❍ Ja, helt [1] ❍ Nej [3]
❍ Ja, delvis [2] ❍ Vet ej / vill ej svara [998]

Om IM10 är "ja, delvis", visa IM20

[vac_partly]

IM20. Vilka av följande har ditt barn fått vaccin mot? (Markera alla aktuella)

❑ Difteri, stelkramp, kikhosta [1]
❑ Polio [2]
❑ Hib (haemophilus influenzae typ b) [3]
❑ MPR – mässlingen, påssjuka, röda hund [4]
❍ Inget av dessa [996]
❍ Vet ej / vill ej svara [998]

Om IM10 är "nej", visa IM25

[vac_notprogram]

IM25. Varför fick ditt barn inte vaccinationerna i det svenska vaccinationsprogrammet?

❍ Mitt barn bodde utomlands och fick vaccin där [1]
❍ Jag tror att vaccinet är skadligt [2]
❍ Annat skäl [3]
❍ Vet ej / vill ej svara [998]

[vac_vacc]

IM30. Har ditt barn vaccinerats för något av följande? (Markera alla aktuella)

Ja
❑ Rotavirus [rot]
❑ Vattkoppor [var]
❑ TBE, Borrelia [tbe]
❑ Hepatit B [hep]
❑ Pneumokocker [pne]
❑ HPV (humant papillomvirus) [hpv]
❑ BCG - tuberkulos (Bacillus Calmette-Guérin) [bcg]
❑
❍ Nej [none]
❍ Vet ej / vill ej svara [998]

Sömn

❖ **Z1. OM ÅLDER < 6 VISA NEDANSTÅENDE FRÅGOR**

Napp

[sle_pacifier]

C.SP10. Använder ditt barn eller har han/hon använt något av följande?

Ja
❑ Napp [pac]
❑ Suga på tumme eller finger [suc]
❑ Kramdjur eller snuttetrasa [cud]

❑
❍ Nej [no]
❍ Vet ej / vill ej svara [998]

Om C.SP10 är "napp", "suger..." eller "kramdjur...", visa C.SP15

[sle_pacifier_now]

C.SP15. Använder ditt barn napp, suger på tumme eller finger, eller har kramdjur eller snuttetrasa nu?

❍ Ja [1]
❍ Nej [2]
❍ Vet ej / vill ej svara [998]

Om C.SP15 är "ja", visa C.SP20

[sle_pacifier_frq]

C.SP20. Vid vilka tillfällen {använder, använde} ditt barn {napp, suger på tumme eller finger, eller har kramdjur eller snuttetrasa, napp, sög på tumme eller finger, eller hade kramdjur eller snuttetrasa}?

❍ Alltid [all]
❑ På natten [nig]
❑ Som tröst [com]
❍ Vet ej / vill ej svara [998]

Om C.SP15 är "nej", visa C.SP30

[sle_pacifier_quit]

C.SP30. Hur gammal var ditt barn när han/hon slutade med napp, att suga på tummen eller fingret, eller att ha kramdjur eller snuttetrasa?

❍ (År gammal) [yrs]________ [yrs_other] ❍ Vet ej / vill ej svara [998]
❍ (Månader gammal) [mnt]____________ [mnt_other]

❖ **Z1. VILLKORET AVSLUTAS**

Tupplur

❖ **AA1. OM ÅLDER ÄR FRÅN 1 TILL 10 VISA NEDAN FRÅGOR**

[sle_nap]

C.SP35. Brukar ditt barn sova en stund under dagen?

❑ Ja, på vardagarna [wkd]
❑ Ja, på helgerna [wke]
❍ Nej [no]
❍ Vet ej / vill ej svara [998]

Om C.SP35 är "ja, på vardagarna" eller "ja, på helgerna", visa C.SP37 och C.SP40

[sle_nap_frq]

C.SP37. Hur många tupplurar per dag brukar ditt barn ta på en {vardag, helgdag}?

❍ 1 gång [1]
❍ 2 gånger [2]
❍ 3 gånger [3]
❍ 4 gånger eller fler [4]
❍ Vet ej / vill ej svara [998]

[sle_nap_quit]

C.SP40. Hur mycket sömn får ditt barn totalt under tupplurarna på en {vardag, helgdag}?

❍ Mindre än 30 minuter [1]
❍ 30 till 59 minuter [2]
❍ 1 till 2 timmar [3]
❍ Mer än 2 timmar [4]
❍ Vet ej / vill ej svara [998]

❖ **AA1. VILLKORET AVSLUTAS**

Sovtid

❖ **AA2. OM ÅLDER < 15 VISA NEDAN FRÅGOR**

[sle_sleeptime]

C.SP45. Hur många timmar sover ditt barn varje natt?

❍ Mindre än 8 timmar [1]
❍ 8 till 10 timmar [2]
❍ 11 till 12 timmar [3]
❍ 13 till 14 timmar [4]
❍ Mer än 14 timmar [5]
❍ Vet ej / vill ej svara [998]

[sle_asleep]

C.SP50. Hur lång tid tar det vanligtvis för ditt barn att somna på kvällen?

❍ Mindre än 5 minuter [1]
❍ 5 till 14 minuter [2]
❍ 15 till 29 minuter [3]
❍ 30 till 59 minuter [4]
❍ 60 minuter eller mer [5]
❍ Vet ej / vill ej svara [998]

Om ålder < 2, visa C.SP55

[sle_position]

C.SP55. Hur lägger du vanligtvis ditt barn i sängen?

❍ På rygg [1] ❍ Vet ej / vill ej svara [998]

❍ På sidan [2]

❍ På mage [3]

Om ålder >= 1, visa C.SP60 till C.SP90

[sle_weekday_rise]

C.SP60. Hur dags brukar ditt barn {vakna, stiga upp} på en {vardag, skoldag}?

Klockan	och (minut)
❍ 5 eller tidigare [1]	❍ 00 [1]
❍ 6 [2]	❍ 15 [2]
❍ 7 [3]	❍ 30 [3]
❍ 8 [4]	❍ 45 [4]
❍ 9 [5]	
❍ 10 [6]	
❍ 11 eller senare [7]	

[sle_weekday_bed]

C.SP70. Hur dags brukar ditt barn gå och lägga sig (lyset släcks) kvällen före en {vardag, skoldag}?

Klockan	och (minut)
❍ 19 eller tidigare [1]	❍ 00 [1]
❍ 20 [2]	❍ 15 [2]
❍ 21 [3]	❍ 30 [3]
❍ 22 [4]	❍ 45 [4]
❍ 23 [5]	

❍ 24 [6]

❍ 1 eller senare [7]

[sle_dayoff_rise]

C.SP80. Hur dags brukar ditt barn stiga upp på "lediga dagar"?

Klockan	och (minut)
❍ 6 eller tidigare [1]	❍ 00 [1]
❍ 7 [2]	❍ 15 [2]
❍ 8 [3]	❍ 30 [3]
❍ 9 [4]	❍ 45 [4]
❍ 10 [5]	
❍ 11 [6]	
❍ 12 eller senare [7]	

[sle_dayoff_bed]

C.SP90. Hur dags brukar ditt barn gå och lägga {sig, dig} (lyset släcks) kvällen före "lediga dagar”?

Klockan	och (minut)
❍ 20 eller tidigare [1]	❍ 00 [1]
❍ 21 [2]	❍ 15 [2]
❍ 22 [3]	❍ 30 [3]
❍ 23 [4]	❍ 45 [4]
❍ 24 [5]	
❍ 1 [6]	
❍ 2 eller senare [7]	

❖ **AA2. VILLKORET AVSLUTAS**

Sömnproblem

❖ **AA3. OM ENKÄTEN BESVARAS AV "FÖRÄLDER" OCH ÅLDER >= 1 VISA NEDAN FRÅGOR**

[sle_wakeup]

C.SP100. Under veckodagar, brukar ditt barn vakna själv på morgonen?

❍ Ja [1]
❍ Nej, jag väcker honom/henne [2]
❍ Vet ej / vill ej svara [998]

Om C.SP100 är "nej,...", visa C.SP105

[sle_wakeup_problem]

C.SP105. Brukar det vara svårt för ditt barn att vakna?

❍ Ja [1] ❍ Vet ej / vill ej svara [998]
❍ Nej [2]

❖ **AA3. VILLKORET AVSLUTAS**

[sle_wakeup_night]

C.SP110. Brukar ditt barn vakna på natten?

❍ Sällan eller aldrig [3]
❍ 1 till 2 gånger varje natt [2]
❍ 3 gånger eller mer varje natt [1]
❍ Vet ej / vill ej svara [998]

Om C.SP110 är oftare än "sällan eller aldrig", visa C.SP120

[sle_fallasleep]

C.SP120. Hur lång tid tar det för ditt barn att somna om?

❍ Mindre än 15 minuter [1]
❍ 15 till 30 minuter [2]
❍ Mer än 30 minuter [3]
❍ Vet ej / vill ej svara [998]

Om ålder < 4, visa C.SP130

[sle_samebed]

C.SP130. Sover ditt barn minst halva natten i samma säng som mamman eller pappan?

❍ Ja, ofta [1] ❍ Aldrig [3]
❍ Ja, ibland [2] ❍ Vet ej / vill ej svara [998]

❖ **AA4. OM ENKÄTEN BESVARAS AV "FÖRÄLDER" VISA NEDAN FRÅGOR**

[sle_snore]

C.SP140. Snarkar ditt barn för närvarande?

❍ Ja [1] ❍ Vet ej / vill ej svara [998]
❍ Nej [2]

Om C.SP140 är "ja", visa C.SP150 och C.SP160

[sle_snore_frq]

C.SP150. Hur ofta snarkar ditt barn?

❍ Sällan eller aldrig [4] ❍ Vet ej / vill ej svara [998]
❍ 1 till 3 gånger i månaden [3]
❍ 1 till 3 gånger i veckan [2]
❍ 4 gånger eller mer i veckan [1]

[sle_sore_loud]

C.SP160. Hur högljutt snarkar ditt barn?

❍ Ganska tyst [1] ❍ Mycket högt [3]

❍ Måttligt [2]   ❍ Vet ej / vill ej svara [998]

Om C.SP150 är “4 gånger eller mer i veckan”, visa C.SP165

[sle_snore_onset]

C.SP165. Hur gammal var ditt barn när du först lade märke till att han/hon snarkade?

❍ Ynge än 6 månader [1]   ❍ Vet ej / vill ej svara [998]
❍ 6 till 12 månader [2]
❍ 1 till 2 år [3]
❍ 3 till 4 år [4]
❍ 5 år eller äldre [5]

- ❖ **AA4. VILLKORET AVSLUTAS**

[sle_breathe]

C.SP170. Brukar ditt barn kippa efter andan eller sluta andas i sömnen?

❍ Aldrig eller sällan [4]   ❍ Vet ej / vill ej svara [998]
❍ 1 till 3 gånger i månaden [3]
❍ 1 till 3 gånger i veckan [2]
❍ 4 gånger eller mer i veckan [1]

Om ålder >= 4, visa C.SP180

[sle_awake_day]

C.SP180. Har ditt barn problem att hålla sig vaken under dagen?

❍ Aldrig eller sällan [4]   ❍ Vet ej / vill ej svara [998]
❍ 1 till 3 gånger i månaden [3]
❍ 1 till 3 gånger i veckan [2]
❍ 4 gånger eller mer i veckan [1]

- ❖ **AA3. VILLKORET AVSLUTAS**
- ❖ **AA5. OM ÅLDER >= 15, AKTIVERA SÖMNMODULEN I TEMAT MEDICINSK HISTORIA FÖR VUXNA**

Huvudvärk

- **AB. OM ÅLDER >= 11, ANVÄND HUVUDVÄRKSMODULEN I TEMAT MEDICINSK HISTORIA FÖR VUXNA**

Se vuxenmodulen

Hörsel

- **AC. OM ÅLDER >= 11, ANVÄND HÖRSELMODULEN I TEMAT MEDICINSK HISTORIA FÖR VUXNA**

Se vuxenmodulen

Astma och allergi

Astma

- **AD. OM ÅLDER >= 0,5 ANVÄND ASTMAMODULEN I TEMAT INFLAMMATION OCH ALLERGI FÖR VUXNA**

Se vuxenmodulen

Allergi

- **AI. OM ÅLDER >= 1, ANVÄND ALLERGIMODULEN I TEMAT INFLAMMATION OCH ALLERGI FÖR VUXNA**

Se vuxenmodulen

Eksem

- **AI. OM ÅLDER >= 1 VISA NEDANSTÅENDE FRÅGOR**

[ecz_itch]

C.IA290. Har {ditt barn, du} någonsin haft kliande utslag som återkom flera gånger under minst 6 månader?

❍ Ja [1] ❍ Vet ej / vill ej svara [998]
❍ Nej [2]

Om C.IA290 är "ja", visa C.IA300 och C.IA310

[ecz_itchplace]

C.IA300. Har det kliande utslaget någonsin förekommit i armbågsvecken, i knävecken, på framsidan av anklarna, under skinkorna eller runt hals, öron eller ögon?

❍ Ja [1] ❍ Vet ej / vill ej svara [998]
❍ Nej [2]

[ecz_12mnt_itch]

C.IA310. Har {ditt barn, du} haft kliande utslag under de senaste 12 månaderna?

❍ Ja [1] ❍ Vet ej / vill ej svara [998]
❍ Nej [2]

- **AM. OM C.IA290 ÄR "JA", VISA NEDANSTÅENDE FRÅGOR**

Om enkäten besvaras av "förälder", visa C.IA320

[ecz_12mnt_itchage]

C.IA320. Hur gammal var {ditt barn, du} när {han/hon, du} hade kliande utslag första gången?

❍ Yngre än 1 år [1] ❍ 5 år eller äldre [4]
❍ 1 till 2 år [2] ❍ Vet ej / vill ej svara [998]
❍ 3 till 4 år [3]

[ecz_12mnt_itchclear]

C.IA330. Har utslaget gått bort helt någon gång under de senaste 12 månaderna?

❍ Ja [1] ❍ Vet ej / vill ej svara [998]
❍ Nej [2]

[ecz_12mnt_itchawke]

C.IA340. Under de senaste 12 månaderna, hur ofta i genomsnitt, har {ditt barn, du} varit vaken på natten på grund av kliande utslag?

❍ Aldrig [1] ❍ En eller fler nätter i veckan [3]
❍ Mindre än en natt i veckan [2] ❍ Vet ej / vill ej svara [998]

❖ **AM. VILLKORET AVSLUTAS**

[ecz_dryskin]

C.IA380. Har {ditt barn, du} torr hud?

❍ Ja [1] ❍ Vet ej / vill ej svara [998]
❍ Nej [2]

[ecz_urt]

C.IA390. Har {ditt barn, du} någonsin haft nässelutslag (urtikaria)?

❍ Ja [1] ❍ Vet ej / vill ej svara [998]
❍ Nej [2]

Om C.IA390 besvarats "ja", visa C.IA400

[ecz_urt_diag]

C.IA400. Har en läkare ställt diagnosen urtikaria?

❍ Ja [1] ❍ Vet ej / vill ej svara [998]
❍ Nej [2]

Om C.IA400 besvarats "ja", visa C.IA401

[ecz_urt_diag_age]

C.IA401. Vid vilken ålder fick {ditt barn, du} diagnosen urtikaria?

❍ (År gammal) [yrs]_____ [yrs_other]   ❍ Vet ej / vill ej svara [998]

- ❖ **AI OCH AL. VILLKOREN AVSLUTAS**

Skador

Vuxenmodulen används från 0 år. Barnen svarar från 14 år

Se vuxenmodulen. (Frågor addresseras till barn och föräldrar)

Mental hälsa

Temperament

❖ **AN. OM ÅLDER ÄR FRÅN 3 TILL 12 MÅNADER, VISA NEDANSTÅENDE FRÅGOR**

[ibq_1]

IB10. Vänligen markera hur ofta din baby ägnat sig åt nedan beskrivna beteenden under förra veckan.

Kolumnen "Gäller ej" används om du inte sett ditt barn i den beskrivna situationen under senaste veckan.

"Aldrig" används om du sett barnet i den beskrivna situationen under senaste veckan, utan att barnet någon gång ägnade sig åt det beskrivna beteendet.

	Aldrig [7]	Mycket sällan [6]	Mindre än halva tiden [5]	Ungefär halva tiden [4]	Mer än halva tiden [3]	Nästan alltid [2]	Alltid [1]	Gäller ej [994]
1. Under på- och avklädning under den senaste veckan, hur ofta slingrade sig barnet och/eller försökte rulla iväg? [1]	❍	❍	❍	❍	❍	❍	❍	❍
2. När barnet kastades omkring på ett lekfullt sätt, hur ofta skrattade barnet? [2]	❍	❍	❍	❍	❍	❍	❍	❍
3. När barnet var trött, hur ofta visade han/hon att han/hon var	❍	❍	❍	❍	❍	❍	❍	❍

	Aldrig [7]	Mycket sällan [6]	Mindre än halva tiden [5]	Ungefär halva tiden [4]	Mer än halva tiden [3]	Nästan alltid [2]	Alltid [1]	Gäller ej [994]
ledsen? [3]								
4. När barnet presenterades för en okänd vuxen, hur ofta klamrade han/hon sig fast vid en förälder? [4]	❍	❍	❍	❍	❍	❍	❍	❍
5. Hur ofta under förra veckan tyckte barnet om att man läste högt för honom/henne? [5]	❍	❍	❍	❍	❍	❍	❍	❍

[ibq_2]

	Aldrig [7]	Mycket sällan [6]	Mindre än halva tiden [5]	Ungefär halva tiden [4]	Mer än halva tiden [3]	Nästan alltid [2]	Alltid [1]	Gäller ej [994]
6. Hur ofta under förra veckan, lekte barnet med en leksak eller annan sak i 5-10 minuter? [6]	❍	❍	❍	❍	❍	❍	❍	❍
7. Hur ofta under veckan, rörde sig barnet fort mot nya saker? [7]	❍	❍	❍	❍	❍	❍	❍	❍
8. När barnet sattes i	❍	❍	❍	❍	❍	❍	❍	❍

	Aldrig [7]	Mycket sällan [6]	Mindre än halva tiden [5]	Ungefär halva tiden [4]	Mer än halva tiden [3]	Nästan alltid [2]	Alltid [1]	Gäller ej [994]
badvattnet, hur ofta skrattade han/hon? [8]								
9. När det var dags att lägga sig eller att sova en stund och ditt barn inte ville, hur ofta jämrade han/hon sig eller snyftade? [9]	❍	❍	❍	❍	❍	❍	❍	❍
10. Efter att ha sovit, hur ofta grät barnet om ingen kom inom några minuter? [10]	❍	❍	❍	❍	❍	❍	❍	❍

[ibq_3]

	Aldrig [7]	Mycket sällan [6]	Mindre än halva tiden [5]	Ungefär halva tiden [4]	Mer än halva tiden [3]	Nästan alltid [2]	Alltid [1]	Gäller ej [994]
11. Under den senaste veckan, när du matade barnet i ditt knä, hur ofta verkade han/hon ivrig att komma iväg så fort som han/hon hade blivit matad? [1]	❍	❍	❍	❍	❍	❍	❍	❍

	Aldrig [7]	Mycket sällan [6]	Mindre än halva tiden [5]	Ungefär halva tiden [4]	Mer än halva tiden [3]	Nästan alltid [2]	Alltid [1]	Gäller ej [994]
12. När du sjöng eller pratade med din baby, hur ofta lugnade han/hon ner sig omedelbart? [12]	❍	❍	❍	❍	❍	❍	❍	❍
13. När barnet lades på rygg, hur ofta slingrade han/hon sig och vände kroppen? [13]	❍	❍	❍	❍	❍	❍	❍	❍
14. Vid en tittut-lek, hur ofta skrattade barnet? [14]	❍	❍	❍	❍	❍	❍	❍	❍
15. Hur ofta tittade barnet upp från sin lek när telefonen ringde? [15]	❍	❍	❍	❍	❍	❍	❍	❍

[ibq_4]

	Aldrig [7]	Mycket sällan [6]	Mindre än halva tiden [5]	Ungefär halva tiden [4]	Mer än halva tiden [3]	Nästan alltid [2]	Alltid [1]	Gäller ej [994]
16. Hur ofta verkade barnet argt (grät och gnällde) när du lämnade honom/henne i	❍	❍	❍	❍	❍	❍	❍	❍

	Aldrig [7]	Mycket sällan [6]	Mindre än halva tiden [5]	Ungefär halva tiden [4]	Mer än halva tiden [3]	Nästan alltid [2]	Alltid [1]	Gäller ej [994]
spjälsängen? [16]								
17. Hur ofta under den senaste veckan kom barnet att spritta till vid en plötslig ändring i kroppsläge (t.ex. när han/hon flyttades plötsligt)? [17]	❍	❍	❍	❍	❍	❍	❍	❍
18. Hur ofta under den senaste veckan tyckte barnet om att höra ljudet av ord, som i barnvisor? [18]	❍	❍	❍	❍	❍	❍	❍	❍
19. Hur ofta under den senaste veckan tittade barnet på bilder i böcker och/eller tidningar i 5 minuter i sträck eller längre? [19]	❍	❍	❍	❍	❍	❍	❍	❍

[ibq_5]

	Aldrig [7]	Mycket sällan [6]	Mindre än halva tiden [5]	Ungefär halva tiden [4]	Mer än halva tiden [3]	Nästan alltid [2]	Alltid [1]	Gäller ej [994]
20. Vid besök på ett nytt ställe, hur ofta blev ditt barn	❍	❍	❍	❍	❍	❍	❍	❍

	Aldrig [7]	Mycket sällan [6]	Mindre än halva tiden [5]	Ungefär halva tiden [4]	Mer än halva tiden [3]	Nästan alltid [2]	Alltid [1]	Gäller ej [994]
begeistrad över att utforska en ny miljö? [20]								
21. Hur ofta under den senaste veckan, log eller skrattade barnet när man gav honom/henne en leksak? [21]	❍	❍	❍	❍	❍	❍	❍	❍
22. Vid slutet av en spännande dag, hur ofta blev ditt barn gråtfärdigt? [22]	❍	❍	❍	❍	❍	❍	❍	❍
23. Hur ofta under den senaste veckan protesterade barnet när han/hon blev fastspänd eller placerad i ett begränsat utrymme (t.ex. i en barnstol, lekhage, barnbilstol)? [23]	❍	❍	❍	❍	❍	❍	❍	❍
24. När man höll om barnet, under den senaste veckan, verkade han/hon tycka om det? [24]	❍	❍	❍	❍	❍	❍	❍	❍
25. När man visade barnet något att titta på, hur ofta lugnade han/hon ned sig	❍	❍	❍	❍	❍	❍	❍	❍

	Aldrig [7]	Mycket sällan [6]	Mindre än halva tiden [5]	Ungefär halva tiden [4]	Mer än halva tiden [3]	Nästan alltid [2]	Alltid [1]	Gäller ej [994]
omedelbart? [25]								
26. Vid hårtvätt, hur ofta skrek barnet? [26]	❍	❍	❍	❍	❍	❍	❍	❍
27. Hur ofta lade din baby märke till ljudet av ett flygplan som passerade ovanför? [27]	❍	❍	❍	❍	❍	❍	❍	❍
28. När barnet presenterades för en okänd vuxen, hur ofta vägrade han/hon att gå till den okända personen? [28]	❍	❍	❍	❍	❍	❍	❍	❍

[ibq_6]

	Aldrig [7]	Mycket sällan [6]	Mindre än halva tiden [5]	Ungefär halva tiden [4]	Mer än halva tiden [3]	Nästan alltid [2]	Alltid [1]	Gäller ej [994]
29. När du var upptagen med en annan aktivitet, och ditt barn inte kunde få din uppmärksamhet, hur ofta grät han/hon? [29]	❍	❍	❍	❍	❍	❍	❍	❍

	Aldrig [7]	Mycket sällan [6]	Mindre än halva tiden [5]	Ungefär halva tiden [4]	Mer än halva tiden [3]	Nästan alltid [2]	Alltid [1]	Gäller ej [994]
30. Hur ofta under den senaste veckan, tyckte barnet om mjuka, rytmiska aktiviteter som att gunga eller svaja? [30]	❍	❍	❍	❍	❍	❍	❍	❍
31. Hur ofta under den senaste veckan stirrade babyn på en mobil eller på sängskyddet eller en bild i 5 minuter eller mer? [31]	❍	❍	❍	❍	❍	❍	❍	❍
32. När barnet ville ha något, hur ofta blev han/hon ledsen när han/hon inte kunde få vad han/hon ville ha? [32]	❍	❍	❍	❍	❍	❍	❍	❍
33. I närvaro av flera okända vuxna, hur ofta klamrade barnet sig fast vid en förälder? [33]	❍	❍	❍	❍	❍	❍	❍	❍
34. När han/hon blev vyssjad eller kramad verkade ditt barn tycka om det? [34]	❍	❍	❍	❍	❍	❍	❍	❍
35. När du klappade eller mjukt strök barnet över kroppen, hur ofta	❍	❍	❍	❍	❍	❍	❍	❍

	Aldrig [7]	Mycket sällan [6]	Mindre än halva tiden [5]	Ungefär halva tiden [4]	Mer än halva tiden [3]	Nästan alltid [2]	Alltid [1]	Gäller ej [994]
lugnade han/hon ned sig omedelbart? [35]								
36. Hur ofta lät ditt barn som om han/hon pratade när ni åkte bil? [36]	❍	❍	❍	❍	❍	❍	❍	❍
37. När ditt barn placerats i en barnstol eller barnbilstol, hur ofta slingrade han/hon sig och vred på kroppen? [37]	❍	❍	❍	❍	❍	❍	❍	❍

❖ **AN. VILLKORET AVSLUTAS.**

Småbarnsbeteende

❖ **AO. OM ÅLDER ÄR FRÅN 1 TILL 3 ÅR, VISA NEDANSTÅENDE FRÅGOR**

[bit_time_child]

BI10. En typisk vecka, hur mycket tid tillbringar ditt barn med andra småbarn (förutom syskon)?

❍ (timmar per vecka) [hrs] ____________ [hrs_other] ❍ Vet ej / vill ej svara [998]
❍ Ingen kontakt med andra barn [no]

[bit_childcare]

BI20. Använder du någon slags barnomsorg för ditt barn?

❍ Nej [2] ❍ Vet ej / vill ej svara [998]
❍ Ja [1]

Om "ja", visa nedan fråga

[bit_childcare_time]

BI30. Hur många timmars barnomsorg använder du en typisk vecka?

❍ (timmar per vecka) [hrs] ____________ [hrs_other] ❍ Vet ej / vill ej svara [998]

[bit_a1_10]

BI40. Många påståenden nedan beskriver normala känslor och beteenden hos småbarn, men en del påståenden beskriver känslor och beteenden som kan vara ett problem. Gör ditt bästa för att svara på varje påstående om ditt barn.

	Sällan [3]	Ibland [2]	Ofta [1]	Gäller ej [994]
1. Visar glädje när han/hon lyckas (t.ex. klappar för sig själv) [1]	☐	☐	☐	☐
2. Gör illa sig så ofta att du inte kan ta ögonen från honom/henne [2]	☐	☐	☐	☐

	Sällan [3]	Ibland [2]	Ofta [1]	Gäller ej [994]
3. Verkar nervös, spänd eller rädd [3]	☐	☐	☐	☐
4. Är rastlös och kan inte sitta still [4]	☐	☐	☐	☐
5. Följer regler [5]	☐	☐	☐	☐
6. Vaknar på natten och behöver hjälp för att somna om [6]	☐	☐	☐	☐
7. Gråter eller har raseriutbrott tills han/hon är utmattad [7]	☐	☐	☐	☐
8. Är rädd för vissa platser, djur eller saker [8]	☐	☐	☐	☐
9. Har mindre roligt än andra barn [9]	☐	☐	☐	☐
10. Tittar efter dig (eller den andra föräldern) när han/hon är ledsen [10]	☐	☐	☐	☐

[bit_a11_20]

	Sällan [3]	Ibland [2]	Ofta [1]	Gäller ej [994]
11. Gråter eller klänger sig fast vid dig när du försöker gå [1]	☐	☐	☐	☐
12. Oroar sig mycket eller är mycket allvarlig [2]	☐	☐	☐	☐
13. Tittar rakt på dig när du säger hans/hennes namn [3]	☐	☐	☐	☐
14. Reagerar inte när han/hon gör illa sig [4]	☐	☐	☐	☐
15. Är tillgiven med nära och kära [5]	☐	☐	☐	☐
16. Tar inte i vissa saker på grund av hur de känns [6]	☐	☐	☐	☐
17. Har svårt att somna eller att fortsätta sova [7]	☐	☐	☐	☐
18. Springer iväg på allmänna platser [8]	☐	☐	☐	☐

	Sällan [3]	Ibland [2]	Ofta [1]	Gäller ej [994]
19. Leker bra tillsammans med andra barn (förutom syskon) [9]	☐	☐	☐	☐
20. Kan hålla uppmärksamheten länge (förutom när han/hon ser på TV) [10]	☐	☐	☐	☐

[bit_a21_30]

	Sällan [3]	Ibland [2]	Ofta [1]	Gäller ej [994]
21. Har svårt att anpassa sig vid förändringar [1]	☐	☐	☐	☐
22. Försöker att hjälpa när någon gör illa sig (t.ex. ger en leksak) [2]	☐	☐	☐	☐
23. Blir ofta mycket upprörd [3]	☐	☐	☐	☐
24. Sätter mat i halsen eller får kväljningar [4]	☐	☐	☐	☐
25. Härmar lekfulla ljud när du ber honom/henne [5]	☐	☐	☐	☐
26. Vägrar att äta [6]	☐	☐	☐	☐
27. Slår, knuffar, sparkar eller biter barn (förutom syskon) [7]	☐	☐	☐	☐
28. Är destruktiv. Har sönder eller förstör saker med flit [8]	☐	☐	☐	☐
29. Pekar för att visa dig något långt borta [9]	☐	☐	☐	☐
30. Slår, biter eller sparkar dig (eller den andra föräldern) [10]	☐	☐	☐	☐

[bit_a31_34]

	Sällan [3]	Ibland [2]	Ofta [1]	Gäller ej [994]
31. Kramar eller matar dockor eller mjukisdjur	☐	☐	☐	☐

	Sällan [3]	Ibland [2]	Ofta [1]	Gäller ej [994]
[1]				
32. Verkar mycket olycklig, ledsen, deprimerad, eller tillbakadragen [2]	☐	☐	☐	☐
33. Försöker skada dig (eller den andra föräldern) med flit [3]	☐	☐	☐	☐
34. När han/hon är ledsen, blir han/hon väldigt stilla, stelnar eller rör sig inte [4]	☐	☐	☐	☐

[bit_a35_42]

BI50. Följande påståenden beskriver känslor och beteenden som kan vara problem för små barn. En del beskrivningar kan vara lite svåra att förstå, speciellt om du inte sett beteendet hos ditt barn. Gör ditt bästa för att svara på alla påståendena. Vänligen klicka på det svar som bäst beskriver ditt barns beteende under den senaste månaden.

	Sällan [3]	Ibland [2]	Ofta [1]	Gäller ej [994]
35. Lägger/ställer saker i en speciell ordning om och om igen och blir ledsen om han/hon blir avbruten [1]	☐	☐	☐	☐
36. Upprepar samma agerande eller fras om och om igen utan att ha någon glädje av det [2]	☐	☐	☐	☐
37. Upprepar samma rörelse om och om igen (som att gunga eller snurra) [3]	☐	☐	☐	☐
38. Blir okontaktbar. Är helt omedveten om vad som händer runt om honom/henne [4]	☐	☐	☐	☐
39. Tar inte ögonkontakt [5]	☐	☐	☐	☐
40. Undviker fysisk kontakt [6]	☐	☐	☐	☐
41. Gör sig själv illa med flit (t.ex. slår i huvudet) [7]	☐	☐	☐	☐
42. Äter eller dricker sådant som inte är ätbart, som papper eller färg [8]	☐	☐	☐	☐

❖ **AO. VILLKORET AVSLUTAS.**

Föräldraskap

❖ **AP. OM ENKÄTEN BESVARAS AV FÖRÄLDER VISA NEDANSTÅENDE FRÅGOR**

[apq_short]

AP10. Följande är ett antal påståenden om din familj. Ange hur ofta följande vanligtvis sker i ditt hem.

	Aldrig [5]	Nästan aldrig [4]	Ibland [3]	Ofta [2]	Alltid [1]
Du låter barnet veta när han/hon gjort någonting bra [1]	☐	☐	☐	☐	☐
Du hotar att bestraffa ditt barn men faktiskt låter bli att straffa honom/henne [2]	☐	☐	☐	☐	☐
Ditt barn lämnar inte en lapp om vart han/hon tar vägen [3]	☐	☐	☐	☐	☐
Ditt barn pratar sig ur att bli bestraffad efter att han/hon gjort något fel [4]	☐	☐	☐	☐	☐
Ditt barn är ute på kvällarna efter den tid som han/hon skulle vara hemma [5]	☐	☐	☐	☐	☐
Du uppmuntrar ditt barn när han/hon gjort något bra [6]	☐	☐	☐	☐	☐
Du hyllar ditt barn när han/hon gör något bra [7]	☐	☐	☐	☐	☐
Ditt barn är ute med vänner som du inte känner [8]	☐	☐	☐	☐	☐
Du låter ditt barn komma undan bestraffning lindrigt (t.ex. lättar på restriktioner tidigare än du sagt) [9]	☐	☐	☐	☐	☐

- **AP. VILLKORET AVSLUTAS**

Familjeliv

❖ **AQ. OM ENKÄTEN BESVARAS AV FÖRÄLDER ELLER AV BARN FRÅN 14 VISA NEDANSTÅENDE FRÅGOR**

[fes_1_10]

FE10. Följande är ett antal påståenden om familjer. Du bestämmer hur väl de stämmer för de flesta medlemmar i din familj. Kom ihåg att vi vill veta hur du uppfattar din familj, så tänk inte på hur andra medlemmar uppfattar dig.

	Stämmer inte alls [5]	Stämmer inte så bra [4]	Stämmer varken eller [3]	Stämmer ganska bra [2]	Stämmer precis [1]
I vår familj hjälper och stöttar vi verkligen varandra [1]	☐	☐	☐	☐	☐
Vi bråkar mycket i vår familj [2]	☐	☐	☐	☐	☐
Vi tycker att det är viktigt att vara bäst [3]	☐	☐	☐	☐	☐
Vi diskuterar politik och sociala problem [4]	☐	☐	☐	☐	☐
Vi är nästan aldrig arga inför varandra [5]	☐	☐	☐	☐	☐
Det är viktigt att komma någonstans i livet [6]	☐	☐	☐	☐	☐
Vi går sällan på föredrag, teater	☐	☐	☐	☐	☐

	Stämmer inte alls [5]	Stämmer inte så bra [4]	Stämmer varken eller [3]	Stämmer ganska bra [2]	Stämmer precis [1]
eller konsert [7]					
Vänner kommer ofta hem till oss på middag eller besök [8]	☐	☐	☐	☐	☐
Vi är i stort sett mycket prydliga och ordentliga [9]	☐	☐	☐	☐	☐
Vi har inte många regler att följa i vår familj [10]	☐	☐	☐	☐	☐

[fes_11_20]

	Stämmer inte alls [5]	Stämmer inte så bra [4]	Stämmer varken eller [3]	Stämmer ganska bra [2]	Stämmer precis [1]
Det är svårt att rensa luften utan att göra någon upprörd [1]	☐	☐	☐	☐	☐
Det finns ingen i vår familj som utövar någon sport [2]	☐	☐	☐	☐	☐
Det finns en känsla av sammanhållning i vår familj [3]	☐	☐	☐	☐	☐
Vi talar med varandra om våra personliga problem [4]	☐	☐	☐	☐	☐
Det är sällsynt att någon i familjen tappar humöret [5]	☐	☐	☐	☐	☐
Vi är inte särskilt	☐	☐	☐	☐	☐

	Stämmer inte alls [5]	Stämmer inte så bra [4]	Stämmer varken eller [3]	Stämmer ganska bra [2]	Stämmer precis [1]
intresserade av kultur [6]					
Vi går ofta på bio, sportevenemang, etc. [7]	☐	☐	☐	☐	☐
För oss är det viktigt att passa tider [8]	☐	☐	☐	☐	☐
Allt görs enligt en bestämd rutin hemma [9]	☐	☐	☐	☐	☐
Vi kritiserar ofta varandra inom familjen [10]	☐	☐	☐	☐	☐

[fes_21_30]

	Stämmer inte alls [5]	Stämmer inte så bra [4]	Stämmer varken eller [3]	Stämmer ganska bra [2]	Stämmer precis [1]
Vi strävar alltid efter att göra saker lite bättre nästa gång [1]	☐	☐	☐	☐	☐
Vi har sällan intellektuella diskussioner [2]	☐	☐	☐	☐	☐
Att följa regler är viktigt i vår familj [3]	☐	☐	☐	☐	☐
Det finns gott om tid och uppmärksamhet för var och en i familjen [4]	☐	☐	☐	☐	☐
Det är som regel någon som blir upprörd när familjen får kritik utifrån	☐	☐	☐	☐	☐

	Stämmer inte alls [5]	Stämmer inte så bra [4]	Stämmer varken eller [3]	Stämmer ganska bra [2]	Stämmer precis [1]
[5]					
Medlemmar i vår familj oroar sig knappast över sådant som befordran i jobbet, betyg etc. [6]	☐	☐	☐	☐	☐
Vi är inte särskilt engagerade i fritidsaktiviteter [7]	☐	☐	☐	☐	☐
Var och en i familjen ser till att deras rum är städade och fina [8]	☐	☐	☐	☐	☐
Vi har mycket liten gruppanda i vår familj [9]	☐	☐	☐	☐	☐
Det talas helt öppet om pengar och ekonomi vår familj [10]	☐	☐	☐	☐	☐

[fes_31_40]

	Stämmer inte alls [5]	Stämmer inte så bra [4]	Stämmer varken eller [3]	Stämmer ganska bra [2]	Stämmer precis [1]
Vi strävar inte så hårt efter framgång i vår familj [1]	☐	☐	☐	☐	☐
Var och ens skyldigheter är klart bestämda i vår familj [2]	☐	☐	☐	☐	☐
Vi kommer verkligen bra överens med varandra [3]	☐	☐	☐	☐	☐

	Stämmer inte alls [5]	Stämmer inte så bra [4]	Stämmer varken eller [3]	Stämmer ganska bra [2]	Stämmer precis [1]
Vi försöker ofta bräcka varandra inom familjen [4]	☐	☐	☐	☐	☐
Medlemmarna i familjen går ofta ut och roar sig [5]	☐	☐	☐	☐	☐
Vi måste strikt följa de regler som finns i vår familj [6]	☐	☐	☐	☐	☐
Det uppstår ofta spontana diskussioner hos oss [7]	☐	☐	☐	☐	☐
Vi tycker mycket om musik, konst och litteratur [8]	☐	☐	☐	☐	☐
Disken blir nästan alltid avklarad direkt efter måltiden [9]	☐	☐	☐	☐	☐
I vår familj kan man inte göra mycket utan att bli upptäckt [10]	☐	☐	☐	☐	☐

❖ **AQ. VILLKORET AVSLUTAS**

Utveckling och välbefinnande

Skreeningfrågor

❖ **OM BARN >= 4 ÅR VISA NEDANSTÅENDE FRÅGOR**

Styrkor och svårigheter (SDQ)

[daw_sdq1]

SDQ1. Det är värdefullt om du besvarar alla frågor, även om du inte är helt säker eller tycker att frågan verkar konstig.

förälder: Frågorna gäller det beteende [Namn] har haft de senaste 6 månaderna.

barn: Frågorna gäller hur du har haft det de senaste 6 månaderna.

	Stämmer inte [1]	Stämmer delvis [2]	Stämmer helt [3]
förälder: Omtänksam, tar hänsyn till andra människors känslor<br><br>barn: Jag försöker vara vänlig mot andra. Jag bryr mig om deras känslor [1]	☐	☐	☐
förälder: Rastlös, överaktiv, kan inte vara stilla länge<br><br>barn: Jag är rastlös. Jag kan inte vara stilla länge [2]	☐	☐	☐
förälder: Klagar ofta över huvudvärk, ont i magen eller illamående<br><br>barn: Jag har ofta huvudvärk, ont i magen eller illamående [3]	☐	☐	☐
förälder: Delar gärna med sig till andra barn (t.ex. godis, leksaker, pennor)<br><br>barn: Jag delar ofta med mig till andra (t.ex. godis, spel, pennor) [4]	☐	☐	☐
förälder: Har ofta raseriutbrott eller häftigt humör	☐	☐	☐

	Stämmer inte [1]	Stämmer delvis [2]	Stämmer helt [3]
barn: Jag blir mycket arg och tappar ofta humöret [5]			
förälder: Ganska ensam, leker eller håller sig ofta för sig själv<br><br>barn: Jag är ofta för mig själv. Jag gör oftast saker ensam [6]	☐	☐	☐
förälder: Som regel lydig, följer vanligtvis vuxnas uppmaningar<br><br>barn: Jag gör oftast som jag blir tillsagd [7]	☐	☐	☐
förälder: Oroar sig över mycket, verkar ofta bekymrad<br><br>barn: Jag oroar mig mycket [8]	☐	☐	☐
förälder: Hjälpsam om någon är ledsen, upprörd eller känner sig dålig<br><br>barn: Jag är hjälpsam om någon är ledsen, upprörd eller känner sig dålig [9]	☐	☐	☐
förälder: Svårt att sitta stilla, rör och vrider jämt på sig<br><br>barn: Jag har svårt att sitta stilla, jag vill jämt röra och vrida på mig [10]	☐	☐	☐

[daw_sdq2]

SDQ2.

	Stämmer inte [1]	Stämmer delvis [2]	Stämmer helt [3]
förälder: Har minst en god vän (kamrat)<br><br>barn: Jag har en eller flera kompisar [11]	☐	☐	☐
förälder: Slåss/bråkar ofta med andra barn eller mobbar dem<br><br>barn: Jag slåss eller bråkar mycket. Jag kan tvinga andra att göra som jag vill [12]	☐	☐	☐
förälder: Ofta ledsen, nedstämd eller tårögd<br><br>barn: Jag är ofta ledsen, nedstämd eller gråtfärdig [13]	☐	☐	☐
förälder: Vanligtvis omtyckt av andra barn	☐	☐	☐

	Stämmer inte [1]	Stämmer delvis [2]	Stämmer helt [3]
barn: Jämnåriga verkar gilla mig för det mesta [14]			
förälder: Lättstörd, tappar lätt koncentrationen<br><br>barn: Jag har svårt att koncentrera mig, jag är lättstörd [15]	☐	☐	☐
förälder: Nervös eller klängig i nya situationer, blir lätt otrygg<br><br>barn: Jag blir nervös i nya situationer. Jag blir lätt osäker [16]	☐	☐	☐
förälder: Snäll mot yngre barn<br><br>barn: Jag är snäll mot yngre barn [17]	☐	☐	☐
förälder: Ljuger eller fuskar ofta<br><br>barn: Jag blir ofta anklagad för att ljuga eller fuska [18]	☐	☐	☐
förälder: Blir retad eller mobbad av andra barn<br><br>barn: Andra barn eller ungdomar retar eller mobbar mig [19]	☐	☐	☐
förälder: Ställer ofta upp och hjälper andra (föräldrar, {förskollärare, lärare}, andra barn)<br><br>barn: Jag ställer ofta upp och hjälper andra (föräldrar, {förskollärare, lärare}, andra barn) [20]	☐	☐	☐

[daw_sdq3]

SDQ3.

	Stämmer inte [1]	Stämmer delvis [2]	Stämmer helt [3]
förälder: Tänker sig för innan han/hon gör olika saker<br><br>barn: Jag tänker mig för innan jäg gör olika	☐	☐	☐

	Stämmer inte [1]	Stämmer delvis [2]	Stämmer helt [3]
saker [21]			
förälder: Stjäl hemma, i {förskolan, skolan} eller på andra ställen<br>barn: Jag tar saker som inte tillhör mig, t.ex. från {förskolan, skolan} eller andra ställen [22]	☐	☐	☐
förälder: Kommer bättre överens med vuxna än med andra barn<br><br>barn: Jag kommer bättre överens med vuxna än med jämnåriga [23]	☐	☐	☐
förälder: Rädd för mycket, är lättskrämd<br><br>barn: Jag är rädd för mycket, jag är lättskrämd [24]	☐	☐	☐
förälder: Fullföljer uppgifter, bra koncentrationsförmåga<br><br>barn: Jag kan koncentrera mig, göra klart det jag arbetar med [25]	☐	☐	☐

[daw_sdq4]

SDQ4.

förälder: Sammantaget, tycker du att [Namn] har svårigheter på ett eller flera av följande områden: med känslor, koncentration, beteende eller med att komma överens och umgås med andra människor?

barn: Tycker du att du har svårigheter på något av följande: dina känslor, din koncentration, ditt beteende eller med att komma överens och umgås med andra människor?

❍ Nej [1]
❍ Ja, små svårigheter [2]
❍ Ja, klara svårigheter [3]
❍ Ja, allvarliga svårigheter [4]
❍ Vet ej / vill ej svara [998]

❖ **A. OM SDQ4 ÄR "JA, SMÅ…", "JA, KLARA…" ELLER "JA, ALLVARLIGA…", VISA NEDANSTÅENDE FRÅGOR**

[daw_sdq5]

SDQ5.

Hur länge har svårigheterna funnits?

❍ Mindre än 1 månad [1]
❍ 1 till 5 månader [2]
❍ 6 tillo 12 månader [3]
❍ Mer än 1 år [4]
❍ Vet ej / vill ej svara [998]

[daw_sdq6]

SDQ6.

förälder: Oroas eller lider [Namn] av sina svårigheter?

barn: Besväras eller oroas du av svårigheterna?

❍ Inte alls [1]
❍ Bara lite [2]
❍ Ganska mycket [3]
❍ Väldigt mycket [4]
❍ Vet ej / vill ej svara [998]

[daw_sdq7]

SDQ7.

förälder: Stör de svårigheter som [Namn] har hans/hennes vardagsliv inom följande områden?

barn: Stör svårigheterna ditt vardagsliv inom följande områden?

	Inte alls [1]	Bara lite [2]	Ganska mycket [3]	Väldigt mycket [4]
Hemma / i familjen [1]	☐	☐	☐	☐
Med kamrater [2]	☐	☐	☐	☐

	Inte alls [1]	Bara lite [2]	Ganska mycket [3]	Väldigt mycket [4]
{Göromåli förskolan, I skolarbetet} [3]	☐	☐	☐	☐
Vid fritidsaktiviteter [4]	☐	☐	☐	☐

[daw_sdq8]

SDQ8.

förälder: Blir svårigheterna en belastning för dig eller för familjen som helhet?

barn: Tror du att svårigheterna blir jobbiga för människor omkring dig (familj, vänner, lärare etc.)?

❍ Inte alls [1]
❍ Bara lite [2]
❍ Ganska mycket [3]
❍ Väldigt mycket [4]
❍ Vet ej / vill ej svara [998]

- **A. VILLKORET AVSLUTAS**

- **B. BERÄKNING AV FÖLJANDE SKALOR**

daw_sdq_emotscore

daw_sdq_condscore

daw_sdq_hypescore

daw_sdq_peerscore

daw_sdq_prosscore

daw_sdq_totscore

daw_sdq_impascore

daw_sdq_peerminuspros_score

❖ **B. VILLKORET AVSLUTAS**

Social förmåga (SAS)

❖ **A. OM BESVARAD AV "PARENT", VISA NEDANSTÅENDE FRÅGOR**

[daw_sa1_7]

SAS1-7. Hur är [Namn] jämfört med andra i samma ålder i följande situationer:

	Mycket sämre än medel [1]	Lite sämre än medel [2]	Ungefär medel [3]	Lite bättre än medel [4]	Mycket bättre än medel [5]	Vet ej / vill ej svara [998]
Klarar att skoja med andra, kan till exempel acceptera att andra är lättsamt retfulla och reagerar då på ett passande sätt. [1]	☐	☐	☐	☐	☐	☐
Lätt att prata med även om samtalsämnet inte särskilt intresserar honom/henne. [2]	☐	☐	☐	☐	☐	☐
Kan göra kompromisser och vara flexibel. [3]	☐	☐	☐	☐	☐	☐
Kan säga eller göra det rätta för att ta udden av spända eller genanta situationer [4]	☐	☐	☐	☐	☐	☐
Tar det med fattning när han/hon inte vinner eller får som han/hon vill. Bra förlorare. [5]	☐	☐	☐	☐	☐	☐
Andra människor känner sig avspända och hemmastadda ihop med honom/henne. [6]	☐	☐	☐	☐	☐	☐
Kan läsa mellan raderna vad andra verkligen tänker och känner. [7]	☐	☐	☐	☐	☐	☐

[daw_sa8_10]

SAS8-10. Hur är [Namn] jämfört med andra i samma ålder i följande situationer:

	Mycket sämre än medel [1]	Lite sämre än medel [2]	Ungefär medel [3]	Lite bättre än medel [4]	Mycket bättre än medel [5]	Vet ej / vill ej svara [998]
När han/hon gjort något fel så kan han/hon be om ursäkt, och reda upp situationen så att fortsatt konflikt eller ovänskap undviks. [8]	☐	☐	☐	☐	☐	☐
Kan ta ledningen utan att andra känner sig kommenderade. [9]	☐	☐	☐	☐	☐	☐
Medveten om vad som är passande och opassande i sociala situationer. [10]	☐	☐	☐	☐	☐	☐

Beräkning av skalan daw_sas_score

- ❖ **A. VILLKORET AVSLUTAS**

Separationsångest (a)

[daw_a1]

förälder: De flesta barn är särskilt fästa vid några få betydelsefulla vuxna som de söker trygghet och tröst hos, och som de vänder sig till när de är ledsna eller har gjort sig illa.

barn: De flesta ungdomar är särskilt fästa vid några få betydelsefulla vuxna som de söker trygghet och tröst hos, och som de vänder sig till när de är upprörda eller har gjort sig illa.

A1.

förälder: Är [Namn] särskilt fäst vid någon av följande vuxna? (Markera alla aktuella)

barn: Är du särskilt fäst vid någon av följande vuxna? (Markera alla aktuella)

❑ förälder: Mor (biologisk eller adoptiv) barn: Din mor (biologisk eller adoptiv) [mot]
❑ förälder: Far (biologisk eller adoptiv) barn: Din far (biologisk eller adoptiv) [fat]
❑ Annan modersfigur (styvmor, fostermor, pappas partner) [stm]
❑ Annan fadersfigur (styvfar, fosterfar, mammas partner) [stf]
❑ En eller flera mor/farföräldrar [gra]
❑ En eller flera andra vuxna släktingar (t.ex. moster/faster, morbror/farbror, vuxen bror eller syster) [rel]
❑ Dagmamma, barnflicka eller au pair [chi]
❑ En eller flera {förskollärare, lärare} [tea]
❑ förälder: En eller flera andra vuxna som han/hon inte är släkt med (t.ex. en vän till familjen eller granne) barn: En eller flera andra vuxna som du inte är släkt med (t.ex. en vän till familjen eller granne) [adu]
❍ Inte särskilt fäst vid någon vuxen [none]
❍ Vet ej / vill ej svara [998]

Om A1 är "inte särskilt…", visa A1a

[daw_a1a]

A1a.

förälder: Är [Namn] särskilt fäst vid följande barn eller unga personer? (Markera alla aktuella)

barn: Är du särskilt fäst vid följande barn eller unga personer? (Markera alla aktuella)

❑ En eller flera bröder, systrar eller andra unga släktingar [sib]
❑ En eller flera vänner [fri]
❍ Inte särskilt fäst vid någon [none]
❍ Vet ej / vill ej svara [998]

Om A1a inte är "inte särskilt...", visa A2

[daw_a2]

A2.

förälder: Du har just berättat för oss vem [Namn] är särskilt fäst vid. Från och med nu kommer vi att hänvisa till dessa personer som hans/hennes"anknytningspersoner".

I nästa fråga vill vi veta hur mycket [Namn] bekymrar sig för att inte få vara tillsammans med sina "anknytningspersoner". De flesta barn har av och till sådan oro. Det vi vill veta är hur [Namn] är i jämförelse med andra i hans/hennes ålder. Vi är intresserade av hur han/hon är vanligtvis – alltså inte under en enstaka "dålig dag".

Sammantaget för de senaste 4 veckorna, har han/hon varit påtagligt orolig för att inte få vara tillsammans med sina anknytningspersoner?

barn: Du har just berättat för oss vem du är särskilt fäst vid. Från och med nu kommer vi att hänvisa till dessa personer som dina "anknytningspersoner".

I nästa fråga vill vi veta hur mycket du bekymrar dig för att inte kunna vara tillsammans med dina "anknytningspersoner". De flesta ungdomar har av och till sådan oro. Det vi vill veta är hur du är i jämförelse med andra i din ålder. Vi är intresserade av hur du vanligtvis är – alltså inte under en enstaka "dålig dag".

Sammantaget för de senaste 4 veckorna, har du varit påtagligt orolig för att inte kunna vara tillsammans med dina anknytningspersoner?

❍ Nej [2]
❍ Ja [1]
❍ Vet ej / vill ej svara [998]

Beräkning av s1_c_mm_positive_a

Rädsla i sociala situationer (c)

[daw_c1]

C1.

<u>förälder:</u> Sammantaget, skulle du säga att [Namn] är särskilt rädd för eller undviker sociala situationer med många människor inblandade, att möta nya människor eller att behöva göra något när andra ser på?

<u>barn:</u> Sammantaget, skulle du säga att du är särskilt rädd för eller undviker sociala situationer med många människor inblandade, att möta nya människor eller att behöva göra något när andra ser på?

❍ Nej [2]
❍ Ja [1]
❍ Vet ej / vill ej svara [998]

Beräkning av s1_c_mm_positive_c

Generaliserad ångest (g)

[daw_g2]

G2.

förälder: Händer det att [Namn] oroar sig?

barn: Händer det att du oroar dig?

❍ Nej [2]
❍ Ja [1]
❍ Vet ej / vill ej svara [998]

Om G2 är "ja", visa g2a

[daw_g2a]

G2A.

förälder: En del barn/ungdomar oroar sig enbart för enstaka saker. Deras bekymmer kan ha samband med någon specifik rädsla, tvångstankar eller separationsångest. Andra barn/ungdomar oroar sig för många olika sidor av sin tillvaro. Dessa barn/ungdomar kan också ha specifika rädslor, tvångstankar eller separationsångest, men de oroar sig alltså dessutom för mycket annat.

Är [Namn] på det hela taget en som oroar sig?

barn: En del ungdomar oroar sig bara för enstaka saker. Deras bekymmer hör ibland ihop med någon specifik rädsla, tvångstankar eller separationsångest. Andra ungdomar oroar sig för många sidor av sin tillvaro. Dessa ungdomar kan också ha specifika rädslor, tvångstankar eller separationsångest, men de oroar sig alltså dessutom för mycket annat.

Är du på det hela taget en som oroar sig?

❍ förälder: Nej, han/hon har endast några få specifika bekymmer barn: Nej, jag har bara några få specifika bekymmer [2]
❍
❍ förälder: Ja, han/hon oroar sig vanligtvis för något barn: Ja, jag oroar mig vanligtvis för något [1]
❍ Vet ej / vill ej svara [998]

Beräkning av visningsvillkor för G3

[daw_g3]

G3.

förälder: Har [Namn] under de 6 senaste månaderna haft så mycket oro för så många saker, att detta verkligen har upprört honom/henne eller så att det har påverkat hans/hennes liv?

barn: Har du under de 6 senaste månaderna haft så mycket oro för så många saker, att detta verkligen har upprört dig eller påverkat ditt liv?

❍ Nej [1]
❍ Kanske [2]
❍ Definitivt [3]
❍ Vet ej / vill ej svara [998]

Beräkning av s1_c_mm_positive_g

Depression (h)

[daw_h1]

H1.

Förälder: Har det under de senaste 4 veckorna funnits stunder då [Namn] varit mycket ledsen, olycklig, nedstämd eller tårögd?

barn: Har det under de senaste 4 veckorna funnits stunder då du varit mycket ledsen, olycklig, nedstämd eller gråtfärdig?

❍ Nej [2]
❍ Ja [1]
❍ Vet ej / vill ej svara [998]

[daw_h7]

H7.

förälder: Har det under de senaste 4 veckorna funnits stunder när [Namn] har varit vresig eller irriterad på ett sätt som inte liknar honom/henne?

barn: Har det under de senaste 4 veckorna funnits stunder när du har varit vresig eller irriterad på ett sätt som inte är likt dig?

❍ Nej [2]
❍ Ja [1]
❍ Vet ej / vill ej svara [998]

[daw_h13]

H13.

förälder: Har det under de senaste 4 veckorna funnits stunder när [Namn] förlorade intresset och glädjen för allt eller nästan allt, som han/hon normalt tycker om att göra?

barn: Har det under de senaste 4 veckorna funnits stunder när du tappade intresset för allt eller nästan allt, som du normalt tycker om att göra?

❍ Nej [2]
❍ Ja [1]
❍ Vet ej / vill ej svara [998]

- ❖ **A. OM H1 ÄR "NEJ" OCH H7 ÄR "NEJ" OCH H13 ÄR "NEJ", VISA NEDANSTÅENDE FRÅGOR**

[daw_h22]

H22.

<u>förälder:</u> Har han/hon under de senaste 4 veckorna pratat om att avsiktligt skada sig själv?

<u>barn:</u> Har du under de senaste 4 veckorna tänkt på att avsiktligt skada dig själv?

❍ Nej [2]
❍ Ja [1]
❍ Vet ej / vill ej svara [998]

[daw_h23]

H23.

<u>förälder:</u> Har han/hon under de senaste 4 veckorna försökt skada sig själv?

<u>barn:</u> Har du under de senaste 4 veckorna försökt skada dig själv?

❍ Nej [2]
❍ Ja [1]
❍ Vet ej / vill ej svara [998]

[daw_h24]

H24.

<u>förälder:</u> Har han/hon någonsin talat om att skada sig själv?

<u>barn:</u> Har du någonsin, alltså under hela ditt liv, försökt skada dig själv?

❍ Nej [2]
❍ Ja [1]
❍ Vet ej / vill ej svara [998]

Beräkning av daw_m1_h_del

- ❖ **VILLKORET AVSLUTAS**

Beräkning av s1_c_mm_positive_h

Uppmärksamhet och aktivitet (j)

Om enkäten besvaras av "förälder", visa J1a

[daw_j1a]

J1. Tycker du att [Namn] har tydliga problem med överaktivitet och dålig koncentration för sin ålder?

❍ Nej [2]
❍ Ja [1]
❍ Vet ej / vill ej svara [998]

Om enkäten besvaras av "barn", visa J1b

[daw_j1b]

J1-3.

	Nej eller inte tillämpligt [1]	Lite [2]	Mycket [3]	Vet ej / vill ej svara [998]
Klagar dina lärare på att du har problem med överaktivitet eller dålig koncentration? [1]	☐	☐	☐	☐
Klagar din familj på att du har problem med överaktivitet eller dålig koncentration? [2]	☐	☐	☐	☐
Och vad tycker du? Har du problem med överaktivitet eller dålig koncentration? [3]	☐	☐	☐	☐

Beräkning av s1_c_mm_positive_j

Besvärlighet och problematiskt uppförande (k)

Om enkäten besvaras av "förälder", visa K1a

[daw_k1a]

K1. Hur har det varit med [Namn] och hans/hennes uppförande under de senaste 6 månaderna jämfört med andra barn i hans/hennes ålder?

❍ Mindre besvärligt och problematiskt än genomsnittet [1]
❍ Som genomsnittet [2]
❍ Mer besvärligt och problematiskt än genomsnittet [3]
❍ Vet ej / vill ej svara [998]

Om enkäten besvaras av "barn", visa K1b

[daw_k1b]

K1-3.

	Nej eller inte tillämpligt [1]	Lite [2]	Mycket [3]	Vet ej / vill ej svara [998]
Klagar dina lärare på att du är jobbig och besvärlig? [1]	☐	☐	☐	☐
Klagar din familj på att du är jobbig och besvärlig? [2]	☐	☐	☐	☐
Och vad tycker du? Är du jobbig och besvärlig? [3]	☐	☐	☐	☐

Beräkning av s1_c_mm_positive_k

Bantning, vikt och kroppsform (p)

[daw_p1]

P1.

	Nej [2]	Ja [1]	Vet ej / vill ej svara [998]
a) förälder: Har [Namn] någonsin tyckt att han/hon är tjock även om andra tyckt att han/hon är *mycket* smal?<br><br>barn: Har du någonsin tyckt att du är tjock även om andra sagt att du är *mycket* smal? [a]	☐	☐	☐
b) förälder: Skulle [Namn] skämmas om andra visste hur mycket han/hon äter?<br><br>barn: Skulle du skämmas om andra visste hur mycket du äter? [b]	☐	☐	☐
c) förälder: Har [Namn] någonsin själv framkallat kräkningar (spytt)?<br>barn: Har du någonsin med avsikt framkallat kräkningar (spytt)? [c]	☐	☐	☐
d) förälder: Påverkas hans/hennes liv av oro kring ätandet (Vad? Var? Hur mycket?)<br><br>barn: Påverkas ditt liv av oro kring att äta (Vad? Var? Hur mycket?) [d]	☐	☐	☐
e) förälder: Om [Namn] äter för mycket, anklagar han/hon sig själv mycket för det?<br><br>barn: Om du äter för mycket, anklagar du dig själv mycket för det? [e]	☐	☐	☐

Beräkning av daw_p1_cond och s1_c_mm_positive_p

Tics (q)

❖ **A. NÄR ENKÄT BESVARAS AV "FÖRÄLDER", VISA NEDANSTÅENDE FRÅGOR**

[daw_q1]

Q1. Har [Namn] under de senaste 12 månaderna haft tics i form av rörelser som han/hon inte verkade kunna kontrollera t.ex. överdrivna blinkningar, grimaser, rörelser kring näsan, huvudnickningar?

❍ Nej [2]
❍ Ja [1]
❍ Vet ej / vill ej svara [998]

[daw_q2]

Q2. Har [Namn] under de senaste 12 månaderna haft tics i form av ljud som han/hon inte verkade kunna kontrollera t.ex. överdrivna sniffningar, hostningar eller harklingar?

❍ Nej [2]
❍ Ja [1]
❍ Vet ej / vill ej svara [998]

❖ **A. VILLKORET AVSLUTAS**

Beräkning av s1_c_mm_positive_q

Vänskap (fr)

[daw_fr1]

Fr1. Hur är det för [Namn] att få kamrater?
❍ Har det svårare än medel [1]
❍ Medel [2]

❍ Lättare än medel [3]
❍ Vet ej / vill ej svara [998]

[daw_fr2]

Fr2. Hur är det för [Namn] att behålla kamrater?
❍ Har det svårare än medel [1]
❍ Medel [2]
❍ Lättare än medel [3]
❍ Vet ej / vill ej svara [998]

[daw_fr3]

Fr3. Hur är det för [Namn] att behålla kamrater?
❍ Ingen [1]
❍ En [2]
❍ 2 till 4 [3]
❍ 5 till 9 [4]
❍ 10 eller fler [5]
❍ Vet ej / vill ej svara [998]

Om Fr3 är ”en” eller fler, visa fr4 till fr6

[daw_fr4]

Fr4. Har [Namn] intressen som {han, hon} delar med sina kamrater?
❍ Nej [1]
❍ Lite [2]
❍ Mycket [3]
❍ Vet ej / vill ej svara [998]

[daw_fr5]

Fr5. Gör [Namn] och {hans, hennes} kamrater aktiviteter tillsammans, t.ex.sportar, spelar dataspel eller shoppar tillsammans?
❍ Nej [1]
❍ Lite [2]
❍ Mycket [3]
❍ Vet ej / vill ej svara [998]

[daw_fr6]

Fr6. Om [Namn] skulle vara mycket stressad, eller om {han, hon} skulle ha ett hemligt bekymmer, tror du att {han, hon} då skulle tala med en kamrat om hur {han, hon} har det?
❍ Nej [1]
❍ Kanske [2]
❍ Definitivt [3]
❍ Vet ej / vill ej svara [998]

{borttagen från v1.0.2}Styrkor (n)

[daw_n1]

N1.

<u>förälder:</u> Passar följande beskrivning in på [Namn]?

<u>barn:</u> Passar följande beskrivning in på dig?

	Nej [1]	Lite [2]	Mycket [3]	Vet ej / vill ej svara [998]
a) Generös [a]	☐	☐	☐	☐
b) Livlig [b]	☐	☐	☐	☐
c) Villig att lära [c]	☐	☐	☐	☐
d) Tillgiven [d]	☐	☐	☐	☐
e) Pålitlig och ansvarsfull [e]	☐	☐	☐	☐
f) Lätt att ha att göra med [f]	☐	☐	☐	☐
g) Rolig, känsla för det humoristiska [g]	☐	☐	☐	☐
h) Mångsidiga intressen [h]	☐	☐	☐	☐
i) Omhändertagande, vänlig [i]	☐	☐	☐	☐
j) Kommer igen snabbt efter motgångar [j]	☐	☐	☐	☐

Uppföljningsfrågor

- **OM BARN >= 4 ÅR VISA NEDANSTÅENDE FRÅGOR**

Utveckling (r)

[daw_r1]

R1. Hur är det med [Namn] och hans/hennes {göromål i förskolan, skolarbete} och förmåga att tänka ut saker och ting - är han/hon ungefär som genomsnittet för sin ålder eller är han/hon före eller efter?

❍ Före [1]
❍ Medel [2]
❍ Efter [3]
❍ Vet ej / vill ej svara [998]

Om R1 is "efter" , visa R2

[daw_r2]

R2. För närvarande, på ungefär vilken åldersnivå befinner sig han/hon {i sina göromål i förskolan, i sitt skolarbete} och när det gäller förmågan att tänka ut saker och ting?

❍ Vet ej
❍ Under 12 månaders nivå [0]
❍ 1 år [1]
❍ 2 år [2]
❍ 3 år [3]
❍ 4 år [4]
❍ 5 år [5]
❍ 6 år [6]
❍ 7 år [7]
❍ 8 år [8]
❍ 9 år [9]
❍ 10 år [10]
❍ 11 år [11]
❍ 12 år [12]
❍ 13 år [13]
❍ 14 år [14]

❍ 15 år [15]
❍ 16 år [16]
❍ 17 år [17]
❍ Vet ej / vill ej svara [998]

[daw_r3]

R3. Är [Namn] när det gäller förmågan att använda språket – att säga vad han/hon menar, och att förstå andra - ungefär genomsnittlig för sin ålder, före eller efter?

❍ Före [1]
❍ Medel [2]
❍ Efter [3]
❍ Vet ej / vill ej svara [998]

Om R3 är "efter" , visa R4 och R5

[daw_r4]

R4. För närvarande, på ungefär vilken åldersnivå befinner han/hon sig språkligt när det gäller att uttrycka sig och att förstå?

❍ Vet ej
❍ Under 12 månaders nivå [0]
❍ 1 år [1]
❍ 2 år [2]
❍ 3 år [3]
❍ 4 år [4]
❍ 5 år [5]
❍ 6 år [6]
❍ 7 år [7]
❍ 8 år [8]
❍ 9 år [9]
❍ 10 år [10]
❍ 11 år [11]
❍ 12 år [12]
❍ 13 år [13]
❍ 14 år [14]
❍ 15 år [15]
❍ 16 år [16]
❍ 17 år [17]
❍ Vet ej / vill ej svara [998]

[daw_r5]

R5. Kan han/hon komma runt sina språkliga problem genom att använda andra kommunikationssätt, t.ex. gester, tecken, ansiktsuttryck eller genom att visa med handlingar?

❍ Nej [1]
❍ Lite [2]
❍ Mycket [3]
❍ Vet ej / vill ej svara [998]

[daw_r6]

R6. Fanns det under hans/hennes 3 första levnadsår något som allvarligt oroade dig eller någon annan när det gäller:

	Nej [2]	Ja [1]	Vet ej / vill ej svara [998]
a) hur tal och språk utvecklades hos [Namn] [a]	☐	☐	☐
b) hans/hennes kontakt med andra [b]	☐	☐	☐
c) utvecklingen av förmågan att leka fantasi- eller låtsaslekar [c]	☐	☐	☐
d) ovanliga ritualer eller ovanliga vanor som var *mycket* svåra att avbryta [d]	☐	☐	☐
e) hans/hennes förmåga att lära sig nya saker, eller att göra nya saker - som att lägga pussel eller att hjälpa till vid påklädningen [e]	☐	☐	☐

Beräkning av daw_r6_cond

Om R6 condition är "1", visa R7

[daw_r7]

R7. Det som gjorde dig eller någon annan allvarligt oroad har det nu löst sig helt och hållet?

❍ Det har löst sig helt [1]
❍ En del problem finns kvar [2]
❍ Vet ej / vill ej svara [998]

❖ **A. OM SAS OCH SDQ VILLKOREN ÄR UPPFYLLDA VISA NEDANSTÅENDE FRÅGOR**

Om R6a är ”ja”, visa R8 och R9

[daw_r8]

R8. Kunde han/hon använda några riktiga ord frånsett ”mamma” eller ”pappa” före två års ålder? *(barnspråk som t.ex. ”tata” för ”kaka” räknas som riktiga ord. Andra ord för mamma eller pappa räknas inte in här)*

❍ Nej [2]
❍ Ja [1]
❍ Vet ej / vill ej svara [998]

[daw_r9]

R9. Efter att först ha använt enstaka ord brukar barn sätta ihop dessa till fraser eller korta meningar som ”gå park se pippi” eller ”mamma ge kaka”.

Satte [Namn] ihop ord till fraser eller korta meningar före 3 års ålder?
(Räkna inte in talesätt eller stående uttryck som t.ex. ”tack så mycket” eller ”god natt” som barnet använder som ett ord)

❍ Nej [2]
❍ Ja [1]
❍ Vet ej / vill ej svara [998]

[daw_r10]

R10. Småbarn kommunicerar ofta med gester som att vinka adjö, peka på saker, ”slängkyssar” eller genom att sätta fingret framför munnen och säga Sschh!(tyst!)

När han/hon var liten, brukade [Namn] använda den sortens gester lika mycket som andra barn i samma ålder?

❍ Ungefär lika mycket [1]
❍ Lite mindre [2]
❍ Mycket mindre [3]
❍ Vet ej / vill ej svara [998]

[daw_r11]

R11. Vissa barn tycker om att leka lekar som ”titt ut”, ”rida rida ranka”, ”baka-kaka” eller andra småbarnslekar.

Brukade [Namn] tycka om sådana lekar?

❍ Nej [1]

❍ Lite [2]
❍ Mycket [3]
❍ Vet ej / vill ej svara [998]

[daw_r12]

R12. Små barn försöker ofta dela nöjen eller sådant som intresserar dem med någon annan, och de försöker ofta visa vad de lärt sig. De kan till exempel visa genom att peka på det som roar eller intresserar dem.

Som liten (före cirka 3 års ålder), ville [Namn] dela det som roade och intresserade honom/henne med andra, och ville han/hon visa det han/hon hade lärt sig för andra?

❍ Nej [1]
❍ Lite [2]
❍ Mycket [3]
❍ Vet ej / vill ej svara [998]

[daw_r13]

R13. Vissa barn ägnar mycket av den tid de leker till att upprepa samma handling om och om igen, till exempel snurra på hjulen på en leksaksbil, öppna och stänga kranar eller trycka av och på en strömbrytare eller öppna och stänga en dörr.

Har det någonsin varit så med [Namn]?

❍ Nej [1]
❍ Lite [2]
❍ Mycket [3]
❍ Vet ej / vill ej svara [998]

[daw_r14]

R14. Barn är ibland på ett oväntat sätt fascinerade av aspekter eller delar av leksaker eller andra saker. De kan till exempel hellre än att leka med en leksak ägna sig åt att lukta på den, eller känna med ett finger på ytan av leksaken, eller hålla leksaken mot ansiktet för att kunna känna om den vibrerar.

Har det någonsin varit så med [Namn]?

❍ Nej [1]
❍ Lite [2]
❍ Mycket [3]
❍ Vet ej / vill ej svara [998]

[daw_r15]

R15. Låtsaslekar är viktiga för vissa barn. Det kan gälla roll-lekar med andra barn - som polis och tjuv eller mamma, pappa, barn. Till och med när de är ensamma kan barn hitta på historier som de gestaltar med dockor, action-gubbar eller rena fantasisaker.

<u>ålder < 11:</u> Som förskolebarn, och även på senare tid, har [Namn] lekt låtsaslekar?

<u>ålder >= 11:</u> Om man tänker på när [Namn] var yngre (låt säga mellan 5 och 10), brukade han/hon då ägna sig åt låtsaslekar?

❍ Nej [1]
❍ Lite [2]
❍ Mycket [3]
❍ Vet ej / vill ej svara [998]

[daw_r16]

R16.

<u>ålder < 11:</u> För närvarande, kan [Namn] anpassa sin lek till dem han/hon leker med? Till exempel, inte vara för brysk när han/hon leker med yngre barn, och att inte försöka bestämma när han/hon leker med äldre barn.

<u>ålder >= 11:</u> När han/hon var yngre (låt säga omkring 10), kunde [Namn] anpassa sin lek till dem han/hon lekte med? Till exempel, inte vara för brysk när han/hon lekte med yngre barn, och att inte försöka bestämma när han/hon lekte med äldre barn.

❍ Nej [1]
❍ Lite [2]
❍ Mycket [3]
❍ Vet ej / vill ej svara [998]

[daw_r17]

R17. När han/hon är tillsammans med andra barn/ungdomar, har han/hon problem med turtagning, att dela eller samarbeta?

❍ Nej [1]
❍ Lite [2]
❍ Mycket [3]
❍ Vet ej / vill ej svara [998]

[daw_r18]

R18. En del barn/ungdomar tycker om att ägna mycket tid åt att samla på saker, eller har stort nöje av att gå in för bara ett ämne, som sport, bilar eller en viss pop-grupp. Man kan säga att dessa barn/ungdomar är ”fixerade” vid sitt intresse, men det rör sig inte om en obehaglig fixering, utan om något som de gillar, och vanligen tycker om att

tala om.

Har [Namn] haft någon långvarig fixering av denna typ?

❍ Nej [1]
❍ Lite [2]
❍ Mycket [3]
❍ Vet ej / vill ej svara [998]

[daw_r19_26]

R19-26. Fixeringar kan gälla vanliga eller ovanliga områden. Till exempel är det ganska vanligt att en 8-åring är fixerad vid dinosaurier, men det är ovanligt för en 8-åring att vara fixerad vid antika stolar, streckkoder eller gatlyktor.

	Nej [1]	Lite [2]	Mycket [3]	Vet ej / vill ej svara [998]
Gällde fixeringen hos [Namn] ett ovanligt område? [r19]	☐	☐	☐	☐
Dominerar/dominerade fixeringen hans/hennes liv? [r20]	☐	☐	☐	☐
Dominerar/dominerade den hans/hennes samtal med andra? [r21]	☐	☐	☐	☐
Förhindrar/förhindrade den honom/henne från att göra andra viktiga saker, till exempel att leka, göra skolarbete eller vara ute? [r22]	☐	☐	☐	☐
Kan [Namn] inleda samtal med andra? [r24]	☐	☐	☐	☐
Om andra människor börjar samtala med honom/henne, kan [Namn] då bidra till att hålla liv i samtalet? [r25]	☐	☐	☐	☐
Är [Namn] genuint intresserad av att prata med andra människor för att få höra om deras erfarenheter och intressen - även om dessa intressen skiljer sig från hans/hennes egna intressen? [r26]	☐	☐	☐	☐

[daw_r27]

R27. En del barn/ungdomar har svårt att anpassa sitt sätt att tala så att det passar i olika sociala sammanhang. De kan till exempel tala alltför slangartat med en {förskollärare, lärare}, eller alltför formellt eller stelt med jämnåriga.

Förändrar [Namn] sitt sätt att tala beroende på om det är en formell (till exempel då han/hon möter en ny {förskollärare, lärare}) eller informell (med jämnåriga han/hon känner) situation?

❍ Nej [1]
❍ Lite [2]

❍ Mycket [3]
❍ Vet ej / vill ej svara [998]

[daw_r28]

R28. Somliga barns/ungdomars känslor är relativt lätta att avläsa om man iakttar deras ansiktsutryck, tonläge och kroppsspråk. Andra unga är svårare att avläsa, särskilt om man inte känner dem mycket väl.

Har de flesta svårt att avläsa känslorna hos [Namn] genom att iaktta hans/hennes ansiktsutryck, tonläge eller kroppsspråk?

❍ Nej [1]
❍ Lite [2]
❍ Mycket [3]
❍ Vet ej / vill ej svara [998]

[daw_r29]

R29. Omvänt, barn/ungdomar har olika förmågor att avläsa andras känslor. Vissa barn/ungdomar är bra på att upptäcka små ledtrådar i kroppsspråk, ansiktsuttryck eller tonläge på rösten hos andra. De vet till exempel genast när deras mamma börjar bli lite arg, eller när ett annat barn/ungdom känner sig lite generad. För andra barn/ungdomar är det mycket svårare att upptäcka dessa ledtrådar.

Har [Namn] svårt att upptäcka de ledtrådar som kan finnas i andras ansiktsuttryck, kroppsspråk och tonläge?

❍ Nej [1]
❍ Lite [2]
❍ Mycket [3]
❍ Vet ej / vill ej svara [998]

[daw_r30]

R30. När vi talar ansikte mot ansikte med någon är ögonkontakt mycket viktig. Vi känner oss vanligen besvärade eller som om något skulle vara fel, om den andre ger för lite ögonkontakt, eller för mycket, eller ger ögonkontakt vid fel tillfällen.

Har [Namn] någonsin haft en period när han/hon har givit för lite ögonkontakt, för mycket eller vid fel tillfällen?

❍ Nej [1]
❍ Kanske [2]
❍ Definitivt [3]
❍ Vet ej / vill ej svara [998]

[daw_r31]

R31. Många små barn går igenom en period när de upprepar de ord någon just sagt till dem. Om du till exempel har sagt, ”vi ska gå hem om 5 minuter” så kan de mekaniskt säga efter ”vi ska gå hem om 5 minuter”. Eller de kanske ”ekar” det sista ordet ”minuter”.

Har [Namn] någonsin ”ekat” eller mekaniskt sagt efter på detta sätt?

❍ Nej [1]
❍ Lite [2]
❍ Mycket [3]
❍ Vet ej / vill ej svara [998]

[daw_r32]

R32. Vissa barn/ungdomar frågar samma sak om och om igen. Till exempel, ”När ska vi gå till parken?” eller ”vad får vi till middag?”, eller ”ska vi åka och bada i helgen?”. De fortsätter att upprepa dessa frågor fastän de redan fått svar på frågan många gånger. Frågorna de upprepar är kanske inte exakt samma från en vecka till en annan.

Har [Namn] någonsin haft en tendens att upprepa frågor på detta sätt?

❍ Nej [1]
❍ Lite [2]
❍ Mycket [3]
❍ Vet ej / vill ej svara [998]

[daw_r33]

R33. Ett annat sätt som barn/ungdomar kan upprepa sig på är genom att använda samma tomma fraser om och om igen. De kan till exempel börja varje mening med ”Om du vill veta min åsikt…” eller ”Logiskt sett….”. Det kanske kan hända att frasen ibland är lämplig, men den används mycket mera än vad som egentligen behövs.

Har [Namn] använt mycket av sådana ganska tomma fraser?

❍ Nej [1]
❍ Lite [2]
❍ Mycket [3]
❍ Vet ej / vill ej svara [998]

[daw_r34]

R34. Somliga barn/ungdomar trivs med rutiner och vill att saker och ting ska vara desamma varje dag. De vill till exempel ha samma mat från samma tallrik medan de sitter på samma stol varenda dag. Eller så kan det finnas mycket fasta rutiner för på-

och avklädning.

Har [Namn] någonsin haft uttalade eller ovanliga rutiner som han/hon hållit fast vid därför att han/hon trivdes med dem?

❍ Nej [1]
❍ Lite [2]
❍ Mycket [3]
❍ Vet ej / vill ej svara [998]

[daw_r36]

R36. Somliga barn har lätt för att bli upprörda av små förändringar av deras rutiner. De kan till exempel bli mycket upprörda av att behöva gå en annan väg till skolan, av att behöva bada/duscha vid en annorlunda tidpunkt, eller om man möblerar om hemma.

Har [Namn] någonsin haft lätt för att bli upprörd om rutiner förändras?

❍ Nej [1]
❍ Lite [2]
❍ Mycket [3]
❍ Vet ej / vill ej svara [998]

If age >= 6, show R37

[daw_r37]

R37. Somliga barn i förskoleåldern har en period med viftningar och flaxande rörelser upp och ner med händer och armar. Vissa barn fortsätter med detta i åratal.

Har [Namn] haft en tendens att vifta med armarna sedan 4 års ålder?

❍ Nej [1]
❍ Lite [2]
❍ Mycket [3]
❍ Vet ej / vill ej svara [998]

[daw_r38]

R38. Du har besvarat många frågor om utvecklingen hos [Namn]—med särskild inriktning på språk, lekbeteende, rutiner, och hans/hennes förmåga att samspela med andra människor.

Är du för närvarande bekymrad över något av dessa områden i utvecklingen hos [Namn]?

❍ Nej [1]

❍ Lite [2]
❍ Mycket [3]
❍ Vet ej / vill ej svara [998]

❖ **B. OM R38 ÄR ”LITE” ELLER ”MYCKET” VISA NEDANSTÅENDE FRÅGOR**

[daw_r39]

R39. Sett till de senaste 12 månaderna, har svårigheter på något av de områden som vi frågat om lett till att han/hon har blivit upprörd eller orolig?

❍ Inte alls [1]
❍ Lite [2]
❍ Måttligt [3]
❍ Mycket[4]
❍ Vet ej / vill ej svara [998]

[daw_r40]

R40. Har svårigheter med språk, rutiner, lek, eller social förmåga påverkat…

	Inte alls [1]	Lite [2]	Måttligt [3]	Mycket [4]	Vet ej / vill ej svara [998]
a) hur bra han/hon kommer överens med dig och resten av familjen? [1]	☐	☐	☐	☐	☐
b) kamratrelationer - att få och behålla vänner? [2]	☐	☐	☐	☐	☐
c) inlärning eller {göromål i förskolan, skolarbete}? [3]	☐	☐	☐	☐	☐
d) lek, hobbies, sport eller andra fritidsaktiviteter? [4]	☐	☐	☐	☐	☐

[daw_r41]

R41. Har dessa bekymmer varit en belastning för dig eller för familjen som helhet?

❍ Inte alls [1]
❍ Lite [2]
❍ Måttligt [3]
❍ Mycket [4]
❍ Vet ej / vill ej svara [998]

[daw_r42]

R42. Somliga barns utveckling har varit ovanlig alltsedan födelsen. Sett i backspegeln inser deras föräldrar att utvecklingen aldrig var helt normal. Så är det dock inte alltid. Ibland är föräldrarna säkra på att utvecklingen var helt normal en tid och att det sedan inträffade en plötslig förändring.

Vilket av detta gäller för [Namn]?

❍ Det fanns alltid en avvikelse [1]
❍ Det inträffade en plötslig förändring [2]
❍ Vet ej / vill ej svara [998]

Om R42 är ”det inträffade...”, visa R43

[daw_r43]

R43. Hur gammal var [Namn] när denna förändring inträffade?

❍ Under 12 månaders nivå [0]
❍ 1 år gammal [1]
❍ 2 år gammal [2]
❍ 3 år gammal [3]
❍ 4 år gammal [4]
❍ 5 år gammal [5]
❍ 6 år gammal [6]
❍ 7 år gammal [7]
❍ 8 år gammal [8]
❍ 9 år gammal [9]
❍ 10 år gammal [10]
❍ 11 år gammal [11]
❍ 12 år gammal [12]
❍ 13 år gammal [13]
❍ 14 år gammal [14]
❍ 15 år gammal [15]
❍ 16 år gammal [16]
❍ 17 år gammal [17]
❍ Vet ej / vill ej svara [998]

❖ **A OCH B. VILLKORET AVSLUTAS**

Separation (a)

❖ **A. OM A2 OCH SDQ VILLKOREN ÄR UPPFYLLDA VISA NEDANSTÅENDE FRÅGOR**

[daw_a3]

A3.

förälder: Under de senaste 4 veckorna, och i jämförelse med andra barn/ungdomar i samma ålder…

barn: Under de senaste 4 veckorna och i jämförelse med andra ungdomar i samma ålder…

	Inte mer än andra (eller inte aktuellt) [1]	Lite mer än andra [2]	Mycket mer än andra [3]	Vet ej / vill ej svara [998]
a) förälder: har han/hon oroat sig för att anknytningspersonerna ska råka ut för något otrevligt eller för att förlora dem?<br><br>barn: har du oroat dig för att anknytningspersonerna ska råka ut för något otrevligt eller för att förlora dem? [a]	☐	☐	☐	☐
b) förälder: har han/hon haft orimlig oro för att bli åtskild från sina anknytningspersoner, t.ex. genom att bli kidnappad, inlagd på sjukhus eller dödad?<br><br>barn: har du haft orimlig oro för att bli åtskild från dina anknytningspersoner, t.ex. genom att bli kidnappad, inlagd på sjukhus eller dödad? [b]	☐	☐	☐	☐
c) förälder:<br>har han/hon vägrat gå till {förskolan, skolan} p.g.a. rädsla för att något hemskt kan hända anknytningspersonerna under tiden han/hon är	☐	☐	☐	☐

	Inte mer än andra (eller inte aktuellt) [1]	Lite mer än andra [2]	Mycket mer än andra [3]	Vet ej / vill ej svara [998]
i {förskolan, skolan}? *(Inkluderar inte motvilja att gå till {förskolan, skolan} av andra skäl, som t.ex. rädsla för att bli mobbad)*<br><br>barn: har du vägrat gå till {förskolan, skolan} p.g.a. rädsla för att något hemskt kan hända anknytningspersonerna under tiden du är i {förskolan, skolan}? *(Inkluderar inte motvilja att gå till {förskolan, skolan} av andra skäl, som t.ex. rädsla för att bli mobbad eller inför examina)* [c]				
d)<br>förälder: har han/hon varit rädd för att sova ensam?<br><br>barn: har du varit rädd för att sova ensam? [d]	☐	☐	☐	☐
e)<br>förälder: har han/hon kommit ut från sitt sovrum på kvällen/natten för att se efter anknytningspersonerna eller för att sova i närheten av dem?<br><br>barn: har du kommit ut från sitt sovrum på kvällen/natten för att se efter anknytningspersonerna eller för att sova i närheten av dem? [e]	☐	☐	☐	☐
f)<br>förälder: har han/hon varit rädd för att sova borta från hemmet?<br><br>barn: har du varit rädd för att sova borta från hemmet? [f]	☐	☐	☐	☐
g)<br>förälder ålder < 11: har han/hon varit rädd för att vara ensam i ett rum även om anknytningspersonerna är i närheten?<br><br>förälder ålder >= 11: har han/hon varit rädd för att vara ensam hemma om anknytningspersonerna skulle gå ut en stund?<br><br>barn: har du varit rädd för att vara ensam hemma om anknytningspersonerna skulle gå ut en stund? [g]	☐	☐	☐	☐
i)<br>förälder: har han/hon haft återkommande				

	Inte mer än andra (eller inte aktuellt) [1]	Lite mer än andra [2]	Mycket mer än andra [3]	Vet ej / vill ej svara [998]
mardrömmar eller obehagliga drömmar om att inte få vara tillsammans med anknytningspersonerna?<br><br>barn: har du haft återkommande mardrömmar eller obehagliga drömmar om att bli separerad från anknytningspersonerna? [i]				
j)<br>förälder: har han/hon haft huvudvärk, magont eller känt sig illamående när han/hon varit tvungen att lämna anknytningspersonerna, eller om han/hon visste att detta skulle ske?<br><br>barn: har du haft huvudvärk, magont eller känt dig illamående när du varit tvungen att lämna anknytningspersonernaeller om du visste att det skulle ske? [j]	☐	☐	☐	☐
k)<br>förälder: har han/hon reagerat med ängslan, gråt, raserianfall, klängighet eller förtvivlan när han/hon varit åtskild från anknytningspersonerna eller vid tanken på att vara åtskild från dem?<br><br>barn: har du blivit ängslig, gråtit, blivit mycket arg eller förtvivlad när du varit åtskild från anknytningspersonerna eller vid tanken på att vara åtskild från dem? [k]	☐	☐	☐	☐

❖ **B. OM NÅGON FRÅGA I A3 ÄR ”MYCKET MER ÄN ANDRA” VISA NEDANSTÅENDE FRÅGOR**

[daw_a4]

A4.

förälder: Har [Namn] haft denna oro att inte få vara tillsammans med sina anknytningspersoner under åtminstone 4 veckor?

barn: Har dina bekymmer för att inte kunna vara tillsammans med dina”anknytningspersoner” funnits under åtminstone 4 veckor?

❍ Nej [2]
❍ Ja [1]
❍ Vet ej / vill ej svara [998]

Om A4 villkoren är uppfyllda, visa A5 till A8

[daw_a5]

A5.

förälder: Hur gammal var han/hon när han/hon började bekymra sig för separation?

barn: Hur gammal var du när du började bekymra dig för inte kunna vara tillsammans med dina ”anknytningspersoner”?

❍ Sedan födelsen [0]
❍ 1 år gammal [1]
❍ 2 år gammal [2]
❍ 3 år gammal [3]
❍ 4 år gammal [4]
❍ 5 år gammal [5]
❍ 6 år gammal [6]
❍ 7 år gammal [7]
❍ 8 år gammal [8]
❍ 9 år gammal [9]
❍ 10 år gammal [10]
❍ 11 år gammal [11]
❍ 12 år gammal [12]
❍ 13 år gammal [13]
❍ 14 år gammal [14]
❍ 15 år gammal [15]
❍ 16 år gammal [16]
❍ 17 år gammal [17]
❍ Vet ej / vill ej svara [998]

[daw_a6]

A6.

förälder: Hur mycket har dessa bekymmer upprört eller oroat honom/henne?

barn: Hur mycket har dessa bekymmer upprört eller oroat dig?

❍ Inte alls [1]

❍ Lite [2]
❍ Måttligt [3]
❍ Mycket [4]
❍ Vet ej / vill ej svara [998]

❖ C. OM A4 VILLKOREN ÄR UPPFYLLDA VISA NEDANSTÅENDE FRÅGOR

[daw_a7]

A7. Har bekymren påverkat…

	Inte Alls [1]	Lite [2]	Måttligt [3]	Mycket [4]	Vet ej / vill ej svara [998]
a) förälder: hur bra han/hon kommer överens med dig och resten av familjen? barn: hur bra du kommer överens med resten av familjen? [a]	☐	☐	☐	☐	☐
b) kamratrelationer - att få och behålla vänner? [b]	☐	☐	☐	☐	☐
c) inlärning eller {göromål i förskolan, skolarbete}? [c]	☐	☐	☐	☐	☐
d) lek, hobbies, sport eller andra fritidsaktiviteter? [d]	☐	☐	☐	☐	☐

[daw_a8]

A8.

förälder: Har dessa bekymmer varit en belastning för dig eller för familjen som helhet?

barn: Har dessa bekymmer blivit jobbiga för dem du har omkring dig (familj, vänner, lärare etc.)?

❍ Inte alls [1]
❍ Lite [2]
❍ Måttligt [3]
❍ Mycket [4]
❍ Vet ej / vill ej svara [998]

- **A, B OCH C. VILLKOREN AVSLUTAS**

Sociala situationer (c)

[daw_c2]

C2.

förälder: Vi är intresserade av om [Namn] är särskilt rädd för sociala sammanhang. Detta om man jämför med andra barn/ungdomar i samma ålder, och om man bortser från vanlig blyghet eller hur han/hon kan vara en tillfälligt dålig dag.

Har [Namn] varit särskilt rädd för någon av de följande sociala situationerna under de senaste 4 veckorna?

barn: Vi är intresserade av om du är särskilt rädd i sociala sammanhang. Detta i jämförelse med jämnåriga, och om man bortser från vanlig blyghet eller hur du kan vara en tillfälligt dålig dag.

Har du varit särskilt rädd för någon av de följande sociala situationerna under de senaste 4 veckorna?

	Nej [1]	Lite [2]	Mycket [3]	Vet ej / vill ej svara [998]
a) Att möta nya människor? [a]	☐	☐	☐	☐
b) Att möta många människor, t.ex. på en fest? [b]	☐	☐	☐	☐
c) Att äta tillsammans med andra? [c]	☐	☐	☐	☐
d) Att prata inför klassen? [d]	☐	☐	☐	☐
e) Att läsa högt när andra hör på? [e]	☐	☐	☐	☐
f) Att skriva när andra ser på? [f]	☐	☐	☐	☐

❖ **A. OM C2 VILLKOREN ÄR UPPFYLLDA VISA NEDANSTÅENDE FRÅGOR**

[daw_c3]

C3.

förälder: De flesta barn är fästa vid några få betydelsefulla vuxna och är tryggare i närheten av dessa. Vissa barn är rädda för sociala situationer endast om dessa inte finns i närheten.

Andra barn är rädda för sociala situationer även om de är tillsammans med sina betydelsefulla vuxna.

Vad gäller för [Namn]?

barn: De flesta ungdomar är fästa vid några få vuxna som betyder mycket för dem och de är tryggare i närheten av dessa. Vissa ungdomar är bara rädda i sociala situationer när de inte har sina betydelsefulla vuxna i närheten.

Andra ungdomar är rädda i sociala situationer även om de är tillsammans med en av dessa betydelsefulla vuxna.

Vad gäller för dig?

❍ Oftast bra i sociala situationer så länge betydelsefulla vuxna finns i närheten [1]
❍ Tydliga sociala rädslor även om betydelsefulla vuxna finns i närheten [2]
❍ Vet ej / vill ej svara [998]

[daw_c4]

C4.

förälder: Är [Namn] bara rädd i situationer med vuxna eller är han/hon också rädd i situationer när många barn är inblandade eller för att träffa nya barn?

barn: Är du bara rädd i situationer med vuxna eller är du också rädd i situationer när många ungdomar är inblandade eller rädd för att träffa nya ungdomar?

❍ Bara med vuxna [1]

❍ förälder: Bara med barn
barn: Bara med ungdomar [2]

❍ förälder: Både vuxna och barn
barn: Både med vuxna och ungdomar [3]
❍ Vet ej / vill ej svara [998]

[daw_c5]

C5.

förälder: När han/hon inte befinner sig i dessa sociala sammanhang, fungerar [Namn] bra tillsammans med de barn och vuxna han/hon känner väl?

barn: När du inte befinner dig i dessa sociala sammanhang, fungerar det bra tillsammans med de ungdomar och vuxna du känner bäst?

❍ Nej [2]
❍ Ja [1]

❍ Vet ej / vill ej svara [998]

[daw_c6]

C6.

förälder: Tror du att han/hon inte tycker om vissa sociala sammanhang för att han/hon är rädd för att uppträda på ett sätt som skulle vara pinsamt eller avslöjande för honom/henne?

barn: Tror du att du inte tycker om sociala situationer för att du är rädd för att uppträda på ett sätt som skulle vara pinsamt eller att du skulle ”göra bort dig”?

❍ Nej [1]
❍ Kanske [2]
❍ Definitivt [3]
❍ Vet ej / vill ej svara [998]

Om daw_c2_cond2 är positivt, visa C7

[daw_c7]

C7.

förälder: Har hans/hennes ovilja mot sociala situationer samband med specifika problem när det gäller att tala, läsa eller skriva?

barn: Har din ovilja mot sociala sammanhang samband med särskilda problem när det gäller att tala, läsa eller skriva?

❍ Nej [1]
❍ Kanske [2]
❍ Definitivt [3]
❍ Vet ej / vill ej svara [998]

[daw_c8]

C8.

förälder: Hur länge har han/hon haft denna rädsla för sociala situationer?

barn: Hur länge har du haft denna rädsla i sociala situationer?

❍ Mindre än 1 månad? [1]
❍ 1 till 5 månader [2]
❍ 6 månader eller mer [3]
❍ Vet ej / vill ej svara [998]

[daw_c9]

C9.

förälder: Hur gammal var han/hon när denna rädsla för sociala situationer började?

barn: Hur gammal var du när denna rädsla för sociala situationer började?

❍ Sedan födelsen [0]
❍ 1 år gammal [1]
❍ 2 år gammal [2]
❍ 3 år gammal [3]
❍ 4 år gammal [4]
❍ 5 år gammal [5]
❍ 6 år gammal [6]
❍ 7 år gammal [7]
❍ 8 år gammal [8]
❍ 9 år gammal [9]
❍ 10 år gammal [10]
❍ 11 år gammal [11]
❍ 12 år gammal [12]
❍ 13 år gammal [13]
❍ 14 år gammal [14]
❍ 15 år gammal [15]
❍ 16 år gammal [16]
❍ 17 år gammal [17]
❍ Vet ej / vill ej svara [998]

Om besvarad av "barn", visa C10a

[daw_c10a]

C10a.

När du hamnar i en social situation som du är rädd för, brukar du…

	Nej [2]	Ja [1]	Vet ej / vill ej svara [998]

	Nej [2]	Ja [1]	Vet ej / vill ej svara [998]
a) rodna (bli röd) eller skaka (darra)? [a]	☐	☐	☐
b) bli rädd att du ska bli illamående (kräkas)? [b]	☐	☐	☐
c) behöva rusa i väg till toaletten eller bli rädd för att inte hinna dit i tid?	☐	☐	☐

[daw_c11a]

C11a.

<u>förälder:</u> När [Namn] är i en social situation som han/hon är rädd för eller tror att han/hon kommer att hamna i en sådan situation, blir han/hon då ångestfylld eller upprörd?

<u>barn:</u> När du är i en social situation du är rädd för eller tror att du kommer att hamna i en sådan situation, blir du då ångestfylld eller upprörd?

❍ Nej [1]
❍ Lite [2]
❍ Mycket [3]
❍ Vet ej / vill ej svara [998]

Om C11a är "mycket", visa C12a

[daw_c12a]

C12a.

<u>förälder:</u> Hur ofta leder hans/hennes rädsla i sociala situationer till att han/hon blir upprörd på detta sätt?

<u>barn:</u> Hur ofta leder din rädsla i sociala situationer till att du blir upprörd på detta sätt?

❍ Av och till [1]
❍ De flesta veckor [2]
❍ De flesta dagar [3]
❍ Flera gånger per dag [4]
❍ Vet ej / vill ej svara [998]

[daw_c13a]

C13a.

<u>förälder:</u> Leder hans/hennes rädsla till att [Namn] undviker sociala sammanhang?

<u>barn:</u> Leder din rädsla till att du undviker sociala situationer?

❍ Nej [1]
❍ Lite [2]
❍ Mycket [3]
❍ Vet ej / vill ej svara [998]

Om C13a är "mycket", visa C14a

[daw_c14a]

C14a.

<u>förälder:</u> Påverkar detta undvikande hans/hennes vardagsliv?

<u>barn:</u> Påverkas ditt vardagsliv av att du undviker sociala situationer?

❍ Nej [1]
❍ Lite [2]
❍ Mycket [3]
❍ Vet ej / vill ej svara [998]

[daw_c15a]

C15a.

<u>förälder:</u> Tycker han/hon själv att denna rädsla för sociala sammanhang är överdriven eller orimlig?

<u>barn:</u> Tycker du själv att din rädsla i sociala situationer är överdriven eller orimlig?

❍ Nej [1]
❍ Kanske [2]
❍ Definitivt [3]
❍ Vet ej / vill ej svara [998]

Beräkna daw_m1_c

[daw_c16a]

C16a.

<u>förälder:</u> Är han/hon upprörd eller ledsen över att ha denna rädsla?

<u>barn:</u> Är du upprörd eller ledsen över att ha denna rädsla?

❍ Nej [1]
❍ Kanske [2]
❍ Definitivt [3]
❍ Vet ej / vill ej svara [998]

[daw_c17a]

C17a.

<u>förälder:</u> Har den rädsla som [Namn] har för sociala situationer blivit en belastning för dig eller för familjen som helhet?

<u>barn:</u> Har din rädsla för sociala situationer blivit jobbig för dem omkring dig (familj, vänner, {förskollärare, lärare} etc.)?

❍ Inte alls [1]
❍ Lite [2]
❍ Måttligt [3]
❍ Mycket[4]
❍ Vet ej / vill ej svara [998]

- **A. VILLKORET AVSLUTAS**

Oro (g)

[daw_g4]

G4.

<u>förälder:</u> Under de 6 senaste månaderna och i jämförelse med andra barn/ungdomar i samma ålder, har [Namn] oroat sig för …

<u>barn:</u> Under de 6 sista månaderna och i jämförelse med andra ungdomar i samma ålder, har du oroat dig för …

	Inte mer än andra [1]	Lite mer än andra [2]	Mycket mer än andra [3]	Vet ej / vill ej svara [998]
a) Tidigare uppförande: Gjorde jag något fel? Har jag gjort någon ledsen? Har de förlåtit mig? [1]	☐	☐	☐	☐
b) <u>ålder < 6:</u> Lärande i förskolan<br><br><u>ålder >= 6:</u> Skolarbete, läxor eller examina [2]	☐	☐	☐	☐
c) Katastrofer: inbrott, rån, brand, bomber etc. [3]	☐	☐	☐	☐
<u>d)</u><br><u>förälder:</u> Hans/hennes egen hälsa<br><br><u>barn:</u> Din egen hälsa [4]	☐	☐	☐	☐
e) Otäcka saker som kan hända andra: familjen, vänner, husdjur, världen (t.ex. krig) [5]	☐	☐	☐	☐
f) Framtiden: t.ex. {byte av förskola, skolbyte}, flytta till annan bostad, skaffa ett arbete, skaffa pojk-/flickvän [6]	☐	☐	☐	☐
g) Skaffa {sig, dig}och behålla vänner [7]	☐	☐	☐	☐
h) Döden och att dö [8]	☐	☐	☐	☐
i) Bli mobbad eller retad [9]	☐	☐	☐	☐
j)<br><u>förälder:</u> Eget utseende eller vikt<br><br><u>barn:</u> Eget utseende eller vikt [10]	☐	☐	☐	☐

Beräkna daw_g4_cond

- ❖ **A. OM TVÅ ELLER FLER FRÅGOR I G4 BESVARATS ”MYCKET MER ÄN ANDRA” VISA NEDANSTÅENDE FRÅGOR**

[daw_g6]

G6.

förälder: Under de 6 senaste månaderna har han/hon haft flera dagar med överdriven oro än utan?

barn: Har du under de 6 senaste månaderna haft flera dagar med mycket oro än utan?

❍ Nej [2]
❍ Ja [1]
❍ Vet ej / vill ej svara [998]

[daw_g7]

G7.

förälder: Är det svårt för honom/henne att få kontroll över oron?

barn: Är det svårt för dig att få kontroll över oron?

❍ Nej [2]
❍ Ja [1]
❍ Vet ej / vill ej svara [998]

- ❖ **B. OM G6 ÄR ”JA” ELLER G7 ÄR ”JA” VISA NEDANSTÅENDE FRÅGOR**

[daw_g8]

G8. För de senaste 6 månaderna:

	Nej [2]	Ja, men inte de flesta av dagarna [1]	Ja, det händer mer än hälften av dagarna [3]	Vet ej / vill ej svara [998]
förälder: Leder oron till att han/hon känner sig rastlös, spänd eller inte kan slappna av?<br><br>barn: Leder oron till att du känner dig rastlös, spänd eller inte kan slappna av? [a]	☐	☐	☐	☐
förälder: Leder oron till att han/hon lättare känner sig trött eller utsliten?<br><br>barn: Leder oron till att du lättare känner dig trött eller utsliten? [b]	☐	☐	☐	☐
förälder: Leder oron till svårigheter med koncentrationen eller ”hjärnsläpp” (att inte komma på det man egentligen vet)?<br><br>barn: Leder oron till svårigheter med koncentrationen eller plötsliga minnessvårigheter? c)	☐	☐	☐	☐
d)<br>förälder: Leder oron till irritabilitet?<br><br>barn: Leder oron till att du blir irriterad?<br><br>[d]	☐	☐	☐	☐
förälder: Leder oron till muskelspänningar?<br><br>barn: Leder oron till att du känner dig spänd i hela kroppen? [e]	☐	☐	☐	☐
förälder: Stör oron hans/hennes sömn, t.ex. så att han/hon har svårt att sova eller somna eller att han/hon har orolig och otillfredsställande sömn?<br><br>barn: Stör oron din sömn, t.ex. så att du har svårt att sova eller somna eller att du har orolig och otillfredsställande sömn? [f]	☐	☐	☐	☐

[daw_g9]

G9.

<u>förälder:</u> Hur upprörd eller plågad är [Namn] till följd av alla hans/hennes olika oroskänslor?

<u>barn:</u> Hur upprörd eller plågad är du till följd av alla dina olika oroskänslor?

❍ Inte alls [1]
❍ Lite [2]
❍ Måttlig [3]
❍ Mycket [4]
❍ Vet ej / vill ej svara [998]

[daw_g10]

G10.

<u>förälder:</u> Har hans/hennes oro påverkat…

<u>barn:</u> Har din oro påverkat…

	Inte alls [1]	Lite [2]	Måttligt [3]	Mycket [4]	Vet ej / vill ej svara [998]
a) <u>förälder:</u> hur bra han/hon kommer överens med dig och resten av familjen?<br><br><u>barn:</u> hur bra du kommer överens med resten av familjen? [a]	☐	☐	☐	☐	☐
b) kamratrelationer - att få och behålla vänner? [b]	☐	☐	☐	☐	☐
c) inlärning eller {göromål i förskolan, skolarbete}? [c]	☐	☐	☐	☐	☐
d) lek, hobbies, sport eller andra fritidsaktiviteter? [d]	☐	☐	☐	☐	☐

[daw_g11]

G11.

<u>förälder:</u> Har denna oro blivit en belastning för dig eller för familjen i helhet?

barn: Har denna oro blivit jobbig för dem du har omkring dig (familj, vänner, lärare etc.)?

❍ Inte alls [1]
❍ Lite [2]
❍ Måttligt [3]
❍ Mycket [4]
❍ Vet ej / vill ej svara [998]

❖ **A OCH B. VILLKORET AVSLUTAS**

Om H2 är "ja", visa H2 till H5

[daw_h2]

H2.

förälder: Du nämnde tidigare att det under de senaste 4 veckorna funnits stunder då [Namn] varit mycket ledsen, olycklig, nedstämd eller tårögd.

Har [Namn] under de senaste 4 veckorna haft en period när han/hon varit mycket nedstämd nästan varje dag?

barn: Du nämnde tidigare att det under de senaste 4 veckorna funnits stunder då du varit mycket ledsen, olycklig, nedstämd eller gråtfärdig.

Har du under de senaste 4 veckorna haft en period när du varit mycket nedstämd nästan varje dag?

❍ Nej [2]
❍ Ja [1]
❍ Vet ej / vill ej svara [998]

[daw_h3]

H3.

förälder: Under den tid [Namn] har varit ledsen, har han/hon varit mycket nedstämd större delen av dagen? (dvs. fler timmar med nedstämdhet än utan)

barn: Under den tid du har varit nedstämd, har du då varit mycket nedstämd under större delen av dagen? (dvs. fler timmar med nedstämdhet än utan)

❍ Nej [2]
❍ Ja [1]
❍ Vet ej / vill ej svara [998]

[daw_h4]

H4.

förälder: Kunde [Namn] muntras upp när han/hon var nedstämd?

barn: Kunde du muntras upp när du var nedstämd?

❍ Lätt [1]
❍ Svårt / bara kortvarigt [2]
❍ Inte alls [3]
❍ Vet ej / vill ej svara [998]

[daw_h5]

H5.

Förälder: Har han/hon under de senaste 4 veckorna varit mycket nedstämd under en period som varat:

barn: Har du under de senaste 4 veckorna varit mycket nedstämd under en period som pågått:

❍ Mindre än 2 veckor [1]
❍ 2 veckor eller mer [2]
❍ Vet ej / vill ej svara [998]

Beräkna daw_m1_h_dep

Om H7 är "ja", visa H8 till H11

[daw_h8]

H8.

förälder: Du nämnde tidigare att det under de senaste 4 veckorna funnits stunder när [Namn] har varit vresig eller irriterad på ett sätt som inte liknar honom/henne.

Har [Namn] under de senaste 4 veckorna haft en period när han/hon varit mycket vresig och irriterad nästan varje dag?

barn: Du nämnde tidigare att det under de senaste 4 veckorna funnits stunder när du har varit vresig eller irriterad på ett sätt som inte är likt dig.

Har du under de senaste 4 veckorna haft en period när du varit mycket vresig och irriterad nästan varje dag?

❍ Nej [2]
❍ Ja [1]
❍ Vet ej / vill ej svara [998]

[daw_h9]

H9.

<u>förälder:</u> Under den period [Namn] har varit vresig och irriterad, har han/hon varit så under större delen av dagen? (dvs. fler timmar med vresighet än utan)

<u>barn:</u> Under den period du har varit vresig och irriterad, har du varit så under större delen av dagen? (dvs. fler timmar med vresighet än utan)

❍ Nej [2]
❍ Ja [1]
❍ Vet ej / vill ej svara [998]

[daw_h10]

H10.

<u>förälder:</u> Har vissa aktiviteter, t.ex. besök av vänner eller annat kunnat minska irritabiliteten hos [Namn]?

<u>barn:</u> Har vissa aktiviteter, t.ex. besök av vänner eller annat kunnat minska din irritabilitet?

❍ Lätt [1]
❍ Svårt/ bara kortvarigt [2]
❍ Inte alls [3]
❍ Vet ej / vill ej svara [998]

[daw_h11]

H11.

<u>förälder:</u> Har han/hon under de senaste 4 veckorna varit mycket irritabel under en period som varat:

<u>barn:</u> Har du under de senaste 4 veckorna varit mycket irritabel under en period som varat:

❍ Mindre än 2 veckor [1]
❍ 2 veckor eller mer [2]
❍ Vet ej / vill ej svara [998]

Beräkna daw_m1_h_irr

Om H13 är "ja", visa H14 till H17

[daw_h14]

H14.

förälder: Du nämnde tidigare att det under de senaste 4 veckorna funnits stunder när [Namn] förlorade intresset och glädjen för allt eller nästan allt, som han/hon normalt tycker om att göra.

Har det de senaste 4 veckorna funnits en period när hans/hennes minskade intresse och glädje visade sig nästan varje dag?

barn: Du nämnde tidigare att det under de senaste 4 veckorna funnits stunder när du förlorade intresset och glädjen för allt eller nästan allt, som du normalt tycker om att göra.

Har du känt sådant minskat intresse nästan varje dag under de senaste 4 veckorna?

❍ Nej [2]
❍ Ja [1]
❍ Vet ej / vill ej svara [998]

[daw_h15]

H15.

förälder: Under de dagar [Namn] förlorade intresset och glädjen för saker och ting, var han/hon så under större delen av dagen? (dvs. fler timmar med problemet än utan)

barn: Under den period då du tappade intresset för saker och ting, var det så under större delen av dagarna? (dvs. fler timmar med problem än utan)

❍ Nej [2]
❍ Ja [1]
❍ Vet ej / vill ej svara [998]

[daw_h16]

H16.

förälder: Har [Namn] under de senaste 4 veckorna haft sådant minskat intresse under:

barn: Har du under de senaste 4 veckorna haft sådant minskat intresse under:

❍ Mindre än 2 veckor [1]
❍ 2 veckor eller mer [2]
❍ Vet ej / vill ej svara [998]

Om depression eller irritabilitet, visa H17

[daw_h17]

H17.

förälder: Har detta minskade intresse och glädje förekommit under samma period som han/hon för det mesta var mycket nedstämd eller irriterad?

barn: Har du haft sådant minskat intresse samtidigt som du var mycket nedstämd eller irriterad?

❍ Nej [2]
❍ Ja [1]
❍ Vet ej / vill ej svara [998]

Beräkna daw_m1_h_los

- **A. OM DEPRESSION, IRRITABILITET ELLER FÖRLORAT INTRESSET VISA NEDANSTÅENDE FRÅGOR**

[daw_h18a_g]

H18.

förälder: Under den period som [Namn] var nedstämd, irriterad och hade minskat intresse och glädje…

barn: Under den period som du var nedstämd, irriterad och hade minskat intresse för att göra saker…

	Nej [2]	Ja [1]	Vet ej / vill ej svara [998]
a) förälder: saknade han/hon energi och verkade trött hela tiden?<br><br>barn: saknade du energi och kände dig trött hela tiden? [a]	☐	☐	☐
b) förälder: åt han/hon mycket mer eller mycket mindre än normalt?<br><br>barn: åt du mycket mer eller mycket mindre än normalt? [b]	☐	☐	☐
c) förälder: ökade eller minskade han/hon i vikt?<br><br>barn: minskade eller ökade du i vikt? [c]	☐	☐	☐
d) förälder: var det svårt för honom/henne att somna in och sova?<br><br>barn: var det svårt för dig att somna eller att fortsätta sova under natten? [d]	☐	☐	☐
e) förälder: var det svårt för honom/henne att somna in och sova?<br><br>barn: sov du för mycket? [e]	☐	☐	☐
f) förälder: var han/hon uppvarvad eller rastlös mycket av tiden?<br><br>barn: var du uppvarvad eller rastlös mest hela tiden? [f]	☐	☐	☐
g) förälder: kände han/hon sig värdelös och onödigt skuldtyngd mycket av tiden?<br><br>barn: kände du dig värdelös och orimligt skuldtyngd mest hela tiden? [g]	☐	☐	☐

[daw_h18h_k]

H18.

förälder: Under den period som [Namn] var nedstämd, irriterad och hade minskat intresse och glädje…

barn: Under den period som du var nedstämd, irriterad och hade minskat intresse för att göra saker…

	Nej [2]	Ja [1]	Vet ej / vill ej svara [998]
h) förälder: tyckte han/hon det var ovanligt svårt att koncentrera sig och att tänka klart?<br><br>barn: hade du ovanligt svårt att koncentrera dig och att tänka klart? [h]	☐	☐	☐
i) förälder: tänkte han/hon mycket på döden?<br><br>barn: tänkte du mycket på döden? [i]	☐	☐	☐
j) förälder: pratade han/hon om att göra sig själv illa eller ta sitt eget liv?<br><br>barn: tänkte du på att göra dig själv illa eller att begå självmord? [j]	☐	☐	☐
k) förälder: försökte han/hon skada sig själv eller ta sitt eget liv?<br><br>barn: försökte du skada dig själv eller begå självmord?<br>[k]	☐	☐	☐

[daw_h18l]

H18L.

förälder: Har han/hon någonsin, alltså under hela sitt liv, försökt skada sig själv eller ta sitt liv?

barn: Har du någonsin, alltså under hela ditt liv, försökt skada dig själv eller begå självmord?

❍ Nej [2]
❍ Ja [1]
❍ Vet ej / vill ej svara [998]

[daw_h19]

H19.

förälder: Hur mycket har nedstämdheten, irritabiliteten eller det minskade intresset hos [Namn] upprört eller plågat honom/henne?

barn: Hur mycket har du plågats av din nedstämdhet, irritabilitet eller det minskade intresset?

❍ Inte alls [1]
❍ Lite [2]
❍ Måttligt [3]
❍ Mycket [4]
❍ Vet ej / vill ej svara [998]

[daw_h20]

H20.

förälder: Har hans/hennes nedstämdhet, irritabilitet eller minskade intresse påverkat…

barn: Har din nedstämdhet, irritabilitet eller minskade intresse och glädje påverkat...

	Inte alls [1]	Lite [2]	Måttligt [3]	Mycket [4]	Vet ej / vill ej svara [998]
a) förälder: hur bra han/hon kommer överens med dig och resten av familjen?<br><br>barn: hur bra du kommer överens med resten av familjen? [1]	☐	☐	☐	☐	☐
b) kamratrelationer - att få och behålla vänner? [2]	☐	☐	☐	☐	☐
c) inlärning eller {göromål i förskolan, skolarbete}? [3]	☐	☐	☐	☐	☐
d) lek, hobbies, sport eller andra fritidsaktiviteter? [4]	☐	☐	☐	☐	☐

[daw_h21]

H21.

förälder: Har hans/hennes nedstämdhet, irritabilitet och minskade intresse blivit en belastning för dig eller för familjen som helhet?

barn: Har din nedstämdhet, irritabilitet och minskade intresse blivit jobbigt för dem du har omkring dig (familj, vänner, {förskollärare, lärare} etc.)?

❍ Inte alls [1]
❍ Lite [2]
❍ Måttligt [3]
❍ Mycket [4]
❍ Vet ej / vill ej svara [998]

❖ **A. VILLKORET AVSLUTAS**

Uppmärksamhet och aktivitet (j)

❖ A. OM SDQ VILLKOREN ÄR UPPFYLLDA VISA NEDANSTÅENDE FRÅGOR

[daw_j2]

J2. Nästan alla barn/ungdomar är någon gång överaktiva och okoncentrerade, och vi önskar veta hur [Namn] är i jämförelse med andra barn/ungdomar i hans/hennes ålder. Vi är intresserade av hur han/hon oftast är – alltså inte under en enstaka ”dålig dag”.

Vi vill nu gå igenom några fler detaljerade frågor om hur [Namn] har varit under de senaste 6 månaderna. Vi börjar med att fråga om hur aktiv han/hon har varit.

Under de senaste 6 månaderna och jämfört med andra barn/ungdomar i hans/hennes ålder…

	Inte mer än andra [1]	Lite mer än andra [2]	Mycket mer än andra [3]	Vet ej / vill ej svara [998]
a) skruvar, vrider och rör han/hon sig ofta oroligt? [1]	☐	☐	☐	☐
b) har han/hon svårt att sitta ner en längre stund? [2]	☐	☐	☐	☐
c) springer han/hon omkring eller klänger och klättrar när han/hon inte borde? [3]	☐	☐	☐	☐
d) är det svårt för honom/henne att leka eller ta del i andra fritidsaktiviteter utan att föra oväsen? [4]	☐	☐	☐	☐
e) när han/hon härjar och springer omkring, är det svårt för honom/henne att lugna ner sig när någon ber honom/henne om det? [5]	☐	☐	☐	☐

[daw_j3]

J3. Under de 6 senaste månaderna och jämfört med andra barn/ungdomar i samma ålder…

	Inte mer än andra [1]	Lite mer än andra [2]	Mycket mer än andra [3]	Vet ej / vill ej svara [998]
a) brukar han/hon slänga ur sig ett svar innan han/hon lyssnat färdigt på frågan? [1]	☐	☐	☐	☐
b) har han/hon svårt att vänta på sin tur? [2]	☐	☐	☐	☐
c) brukar han/hon ofta avbryta andra människors samtal eller lek? [3]	☐	☐	☐	☐
d) brukar han/hon ofta fortsätta prata även om han/hon blivit ombedd att sluta eller om ingen lyssnar? [4]	☐	☐	☐	☐

[daw_j4]

J4. Under de 6 senaste månaderna och i jämförelse med andra barn/ungdomar i samma ålder…

	Inte mer än andra [1]	Lite mer än andra [2]	Mycket mer än andra [3]	Vet ej / vill ej svara [998]
a) gör [Namn] ofta slarvfel eller har han/hon problem att behålla uppmärksamheten på det han/hon gör? [1]	☐	☐	☐	☐
b) verkar det som om han/hon ofta tappar intresset för det han/hon håller på med? [2]	☐	☐	☐	☐
c) brukar han/hon inte lyssna på vad folk säger till honom/henne? [3]	☐	☐	☐	☐
d) fullföljer han/hon ofta inte sina uppgifter ordentligt? [4]	☐	☐	☐	☐
e) har han/hon ofta svårt att organisera sina uppgifter och aktiviteter? [5]	☐	☐	☐	☐
f) brukar han/hon ofta försöka undvika att göra saker där han/hon måste tänka efter{, t.ex. som läxor}? [6]	☐	☐	☐	☐
g) brukar han/hon ofta tappa bort saker han/hon behöver i {förskolan, skolan} eller för att leka? [7]	☐	☐	☐	☐
h) blir han/hon lätt distraherad? [8]	☐	☐	☐	☐
i) är han/hon ofta glömsk? [9]	☐	☐	☐	☐

Beräkna daw_j2_4_cond

[daw_j5]

J5. Har {förskollärare, lärare} till [Namn] under de 6 senaste månaderna klagat på att han/hon har problem med…

	Nej [1]	Lite [2]	Mycket [3]	Vet ej / vill ej svara [998]
a) att sitta stilla, rastlöshet eller överaktivitet? [1]	☐	☐	☐	☐
b) dålig koncentration eller att han/hon lätt blir distraherad? [2]	☐	☐	☐	☐
c) att han/hon handlar utan att tänka sig för, ofta avbryter och stör andra eller inte väntar på sin tur? [3]	☐	☐	☐	☐

Om daw_j2_4_cond är positiv, visa J6 to J10

[daw_j6]

J6. Har svårigheterna med aktivitet eller koncentration hos [Namn] varat minst 6 månader?

❍ Nej [2]
❍ Ja [1]
❍ Vet ej / vill ej svara [998]

[daw_j7]

J7. Hur gammal var han/hon när svårigheterna med aktivitet eller koncentration började?

❍ Sedan födelsen [0]
❍ 1 år gammal [1]
❍ 2 år gammal [2]
❍ 3 år gammal [3]
❍ 4 år gammal [4]
❍ 5 år gammal [5]
❍ 6 år gammal [6]

❍ 7 år gammal [7]
❍ 8 år gammal [8]
❍ 9 år gammal [9]
❍ 10 år gammal [10]
❍ 11 år gammal [11]
❍ 12 år gammal [12]
❍ 13 år gammal [13]
❍ 14 år gammal [14]
❍ 15 år gammal [15]
❍ 16 år gammal [16]
❍ 17 år gammal [17]
❍ Vet ej / vill ej svara [998]

❖ **B. OM VILLKOREN FÖR J2 TILL J4 ÄR UPPFYLLDA VISA NEDANSTÅENDE FRÅGOR**

[daw_j8]

J8. Har svårigheterna med aktivitet eller koncentration hos [Namn] upprört eller plågat honom/henne själv?

❍ Inte alls [1]
❍ Lite [2]
❍ Måttligt [3]
❍ Mycket [4]
❍ Vet ej / vill ej svara [998]

[daw_j9]

J9. Har svårigheterna med aktivitet eller koncentration hos [Namn] påverkat…

	Inte alls [1]	Lite [2]	Måttligt [3]	Mycket [4]	Vet ej / vill ej svara [998]
a) hur bra han/hon kommer överens med dig och resten av familjen? [1]	☐	☐	☐	☐	☐
b) kamratrelationer – att få och behålla vänner? [2]	☐	☐	☐	☐	☐
c) inlärning eller {göromål i förskolan, skolarbete}? [3]	☐	☐	☐	☐	☐
d) lek, hobbies, sport eller andra fritidsaktiviteter? [4]	☐	☐	☐	☐	☐

[daw_j10]

J10. Har dessa svårigheter med aktivitet eller koncentration blivit en belastning för dig eller för resten av familjen?

❍ Inte alls [1]
❍ Lite [2]
❍ Måttligt [3]
❍ Mycket [4]
❍ Vet ej / vill ej svara [998]

❖ **A OCH B. VILLKORET AVSLUTAS**

❖ **A. OM ENKÄTEN BESVARAS AV "FÖRÄLDER", VISA NEDANSTÅENDE FRÅGOR**

[daw_k2a]

K2a. En del barn/ungdomar är besvärliga eller retsamma med bara en viss person, kanske med dig eller en bror eller syster. Andra barn/ungdomar är besvärliga med flera vuxna eller barn. Följande frågor gäller hur [Namn] är i allmänhet, alltså inte bara med en viss person.

Under de 6 senaste månaderna och i jämförelse med andra barn/ungdomar i samma ålder har han/hon ofta…

	Inte mer än andra [1]	Lite mer än andra [2]	Mycket mer än andra [3]	Vet ej / vill ej svara [998]
haft raseriutbrott? [a]	☐	☐	☐	☐
grälat med vuxna? [b]	☐	☐	☐	☐
struntat i regler eller vägrat göra som han/hon blivit tillsagd? [c]	☐	☐	☐	☐
verkat vilja förarga andra med flit? [d]	☐	☐	☐	☐
skyllt på andra för egna misstag eller dåligt uppförande? [e]	☐	☐	☐	☐
varit snarstucken eller stingslig? [f]	☐	☐	☐	☐
varit arg och harmsen? [g]	☐	☐	☐	☐
varit elak? [h]	☐	☐	☐	☐
försökt ge igen på andra? [i]	☐	☐	☐	☐

Beräkna daw_k2a_cond

❖ **B. OM VILLKOREN FÖR K2a ÄR UPPFYLLDA VISA NEDANSTÅENDE FRÅGOR**

[daw_k3a]

K3a. Har hans/hennes {förskollärare, lärare} under de senaste 6 månaderna klagat på att [Namn] har sådant besvärligt eller störande uppförande {i förskolan, under skoltid}?

❍ Nej [1]
❍ Lite [2]
❍ Mycket [3]
❍ Vet ej / vill ej svara [998]

[daw_k4a]

K4. Har [Namn] haft detta besvärliga uppförande i minst 6 månader?

❍ Nej [2]
❍ Ja [1]
❍ Vet ej / vill ej svara [998]

[daw_k5a]

K5. Hur gammal var [Namn] när detta besvärliga uppförande började?

❍ Sedan födelsen [0]
❍ 1 år gammal [1]
❍ 2 år gammal [2]
❍ 3 år gammal [3]
❍ 4 år gammal [4]
❍ 5 år gammal [5]
❍ 6 år gammal [6]
❍ 7 år gammal [7]
❍ 8 år gammal [8]
❍ 9 år gammal [9]
❍ 10 år gammal [10]
❍ 11 år gammal [11]
❍ 12 år gammal [12]
❍ 13 år gammal [13]
❍ 14 år gammal [14]
❍ 15 år gammal [15]
❍ 16 år gammal [16]
❍ 17 år gammal [17]
❍ Vet ej / vill ej svara [998]

[daw_k6a]

K6a. Har detta besvärliga uppförande påverkat…

	Inte alls [1]	Lite [2]	Måttligt [3]	Mycket [4]	Vet ej / vill ej svara [998]
a) hur bra han/hon kommer överens med dig och resten av familjen? [a]	☐	☐	☐	☐	☐
b) kamratrelationer – att få och behålla vänner? [b]	☐	☐	☐	☐	☐
c) inlärning eller {göromål i förskolan, skolarbete}? [c]	☐	☐	☐	☐	☐
d) lek, hobbies, sport eller andra fritidsaktiviteter? [d]	☐	☐	☐	☐	☐

[daw_k7a]

K7a. Har hans/hennes besvärliga uppförande blivit en belastning för dig eller för familjen som helhet?

❍ Inte alls [1]
❍ Lite [2]
❍ Måttligt [3]
❍ Mycket [4]
❍ Vet ej / vill ej svara [998]

❖ **A OCH B. VILLKOREN AVSLUTAS**

[daw_k8aa]

K8aa.

förälder: Vi tänker nu fråga om beteenden som ibland försätter barn/ungdomar i svårigheter. Det inkluderar farligt, aggressivt eller antisocialt beteende.

Såvitt du vet, och sett till de senaste 12 månaderna…

barn: Den här delen av intervjun handlar om uppförande som ibland leder till problem med föräldrar, lärare eller andra vuxna.

Vi ska nu fråga om saker som du eventuellt har gjort under de senaste 12 månaderna.

	Nej eller inte tillämpligt [1]	Kanske [2]	Stämmer för de senaste 6 månaderna [3]	Stämde för 7 till 12 månader sedan men inte efter det [4]

	Nej eller inte tillämpligt [1]	Kanske [2]	Stämmer för de senaste 6 månaderna [3]	Stämde för 7 till 12 månader sedan men inte efter det [4]
a) förälder: har han/hon ofta ljugit för att få saker, eller favörer, eller för att slippa göra saker han/hon förväntats göra?<br><br>barn: har du ofta ljugit för att få saker eller förmåner från andra eller för att slippa göra saker som du borde? [a]	☐	☐	☐	☐
b) förälder: har han/hon ofta startat slagsmål? *(Med andra än syskon)*<br><br>barn: har du ofta börjat slåss (*Med andra än dina syskon*) [b]	☐	☐	☐	☐
c) förälder: har han/hon ofta mobbat eller hotat andra?<br><br>barn: har du ofta mobbat eller hotat folk? [c]	☐	☐	☐	☐
d) förälder: har han/hon ofta varit ute tills det har blivit mörkt, mycket senare än tillåtet?<br><br>barn: har du ofta stannat ute mycket senare än du fick tillåtetelse till? [d]	☐	☐	☐	☐
e) förälder: har han/hon stulit något i hemmet, från andras hem eller från affärer eller {förskolan, skolan}? *(Inkluderar inte småsnatterier, som att ha stulit sin brors penna eller stulit mat från frysen)*<br><br>barn: har du stulit saker hemma eller hemma hos andra eller i affärer eller {förskolan, skolan}? (*Det innefattar inte att ta småsaker t ex att ta sin brors penna eller mat från kylen*) [e]	☐	☐	☐	☐
f) förälder: har han/hon rymt hemifrån mer än en gång eller stannat borta hela natten utan lov?<br><br>barn: har du rymt hemifrån mer än	☐	☐	☐	☐

	Nej eller inte tillämpligt [1]	Kanske [2]	Stämmer för de senaste 6 månaderna [3]	Stämde för 7 till 12 månader sedan men inte efter det [4]
någon enstaka gång eller någonsin varit borta en hel natt utan tillåtelse? [f]				
g) förälder: har han/hon ofta skolkat från skolan?<br><br>barn: har du ofta skolkat från skolan? [g]	☐	☐	☐	☐

Beräkna daw_k8aa_cond1

Beräkna daw_k8aa_cond2

❖ **C. OM VILLKOREN FÖR K8aa ÄR UPPFYLLDA VISA NEDANSTÅENDE FRÅGOR**

[daw_k9a]

K9a.

förälder: Började han/hon skolka innan han/hon hade fyllt 13 år?

barn: Började du skolka före 13 års ålder?

❍ Nej [2]
❍ Ja [1]
❍ Vet ej / vill ej svara [998]

❖ **C. VILLKORET AVSLUTAS**

❖ **D. OM PROBLEMATISKT UPPFÖRANDE VISA NEDANSTÅENDE FRÅGOR**

[daw_k10a]

K10a.

förälder: Vi vill nu fråga dig om beteenden som är mindre vanliga, men som kan vara mycket allvarliga. Vi behöver få ställa samtliga frågor till alla, även om det inte passar in på er.

Så vitt du vet har något av följande hänt minst en gång de senaste 12 månaderna?

barn: Vi kommer nu att fråga om några fler beteenden som ibland ger folk problem. Vi måste ställa samtliga frågor till alla även om de inte passar in på just dig.

Även om det bara hänt en gång, har något av följande hänt under de senaste 12 månaderna…

	Nej [1]	Stämmer för de senaste 6 månaderna [2]	Stämde för 7 till 12 månader sedan men inte efter det [3]
a) förälder: Har han/hon använt vapen eller någonting som allvarligt kan skada någon? *(t.ex. slagträ, sten, trasig flaska, kniv, pistol)*<br><br>barn: Har du använt ett vapen eller något annat i syfte att skada en annan människa? *(t.ex. slagträ, tegelsten, trasig flaska, kniv, pistol*) [a]	☐	☐	☐
b) förälder: Har han/hon faktiskt skadat någon eller varit fysiskt grym mot någon?<br><br>barn: Har du riktigt skadat eller varit fysiskt grym mot någon? *(t.ex. bundit, skurit eller bränt någon)* [b]	☐	☐	☐
c) förälder: Har han/hon varit riktigt elak mot djur och fåglar?<br><br>barn: Har du medvetet varit riktigt grym mot djur och fåglar? [c]	☐	☐	☐
d) förälder: Har han/hon avsiktligt startat en brand? *(Endast om avsikten var att orsaka allvarlig skada. Frågan handlar alltså inte om att t.ex. tända en lägereld eller att tända enstaka tändstickor eller pappersbitar)*<br><br>barn: Har du avsiktligt tänt eld på något? (*Detta bara om du menade att åstadkomma skada. Frågan handlar inte om att tända en vanlig eld eller bränna tändstickor eller pappersbitar*) [d]	☐	☐	☐
e) förälder: Har han/hon avsiktligt förstört	☐	☐	☐

	Nej [1]	Stämmer för de senaste 6 månaderna [2]	Stämde för 7 till 12 månader sedan men inte efter det [3]
någon annans egendom? *(Frågan handlar inte om att starta en brand, eller om mindre handlingar, som att förstöra sin systers teckning. Frågan gäller beteenden som att krossa bilfönster eller vandalism i {förskolan, skolan}.)*<br><br>barn: Har du avsiktligt förstört någon annans egendom? (*Det gäller inte att anlägga eld eller obetydliga småsaker t.ex. att förstöra systerns teckningar. Det innefattar att slå sönder fönsterrutor eller vandalism i {förskolan, skolan})* [e]			
f) förälder: Har han/hon varit inblandad i stöld på gatan, t.ex. väskryckning eller rån?<br><br>barn: Har du någon gång varit inblandad i att stjäla, t.ex. väskryckning eller rån? [f]	☐	☐	☐
g) förälder: Har han/hon försökt tvinga någon till sexuell handling mot dennes vilja?<br><br>barn: Har du försökt tvinga någon till en sexuell handling mot hans/hennes vilja? [g]		☐	☐
h) förälder: Har han/hon brutit sig in i hus, byggnader eller bilar?<br><br>barn: Har du gjort inbrott i ett hus, någon annan byggnad eller en bil? [h]	☐	☐	☐

Om enkät besvaras av "förälder", visa K11a

[daw_k11a]

K11a.

Har {förskollärare, lärare} klagat över att [Namn] haft ett sådant problematisktuppförande under de senaste 6 månaderna?

❍ Nej [2]
❍ Ja [1]
❍ Vet ej / vill ej svara [998]

[daw_k11b]

K11b.

förälder: Har [Namn] haft detta problematiska uppförande i minst 6 månader?

barn: Du har berättat om det du gör som leder till att du får problem. Har du gjort så här i minst 6 månader?

❍ Nej [2]
❍ Ja [1]
❍ Vet ej / vill ej svara [998]

[daw_k11c]

K11c.

förälder: Har [Namn] någon gång haft problem med polisen?

barn: Har du åkt dit för polisen någon gång?

❍ Nej [2]
❍ Ja [1]
❍ Vet ej / vill ej svara [998]

Beräkna daw_k11c_cond

❖ E. OM PROBLEMATISKT UPPFÖRANDE VISA NEDANSTÅENDE FRÅGOR

[daw_k12a]

K12a.

förälder: Har det problematiska uppförandet hos [Namn] påverkat…

barn: Har ditt problematiska uppförande påverkat…

	Inte alls [1]	Lite [2]	Måttligt [3]	Mycket [4]	Vet ej / vill ej svara [998]
a) <u>förälder:</u> hur bra han/hon kommer överens med dig och resten av familjen?<br><br><u>barn:</u> hur bra du kommer överens med resten av familjen? [a]	☐	☐	☐	☐	☐
b) kamratrelationer - att få och behålla vänner? [b]	☐	☐	☐	☐	☐
c) inlärning eller {göromål i förskolan, skolarbete}? [c]	☐	☐	☐	☐	☐
d) lek, hobbies, sport eller andra fritidsaktiviteter? [d]	☐	☐	☐	☐	☐

[daw_k13a]

K13a.

<u>förälder:</u> Har hans/hennes problematiska uppförande blivit en belastning för dig eller familjen som helhet?

<u>barn:</u> Har ditt problematiska uppförande blivit jobbigt för dem du har omkring dig (familj, vänner, {förskollärare, lärare} etc.)?

❍ Inte alls [1]
❍ Lite [2]
❍ Måttligt [3]
❍ Mycket [4]
❍ Vet ej / vill ej svara [998]

❖ **D OCH E. VILLKORET AVSLUTAS**

Bantning, vikt och kroppsform (p)

Om ålder >= 7, visa denna modul

- **A. OM VILLKORET FÖR P1 ÄR UPPFYLLT VISA NEDANSTÅENDE FRÅGOR**

Visa uttryck relaterat till graviditet för flickor i ålder >= 15

[daw_p2]

förälder: Hur lång är [Namn]? (ungefär)<br>barn: Hur lång är du? (ungefär) (cm) [a]	_______
förälder: Hur mycket väger [Namn] för närvarande? (ungefär)<br>barn: Hur mycket väger du för närvarande? (ungefär) (kg) [b]	_______
förälder: Vad har han/hon vägt som minst under de senaste 12 månaderna?<br>barn: Vad har du vägt som minst under de senaste 12 månaderna? (kg) [c]	_______
förälder: Vad har han/hon vägt som mest under sitt liv? (kg)<br>*(ej vikt under ev. graviditet)*<br>barn: Vad har du vägt som mest i ditt liv? *(ej vikt under ev. graviditet)* (kg) [d]	_______

[daw_p3a]

P3a.

förälder: För närvarande, skulle du beskriva [Namn] som mycket mager, mager, medel, rund eller tjock?

barn: Hur skulle andra, som vänner och familj, beskriva dig nu - som mycket mager, mager, medel, rund eller tjock?

❍ Mycket mager[1]
❍ Mager [2]
❍ Medel [3]
❍ Rund [4]
❍ Tjock [5]
❍ Vet ej / vill ej svara [998]

Om P3a är "mycket mager" eller "mager", visa P4

[daw_p4a]

P4.

förälder: Om du jämför hur han/hon är i år med hur han/hon varit under tidigare år, skulle du då säga att han/hon:

barn: När man jämför med hur det är i år med hur du varit förr, skulle andra människor säga att du:

❍ Varit ännu magrare tidigare år [1]
❍ Alltid lika mager [2]
❍ Lite magrare i år än tidigare år [3]
❍ Mycket magrare i år än tidigare år [4]
❍ Vet ej / vill ej svara [998]

[daw_p5a]

P5a.

förälder: För närvarande, skulle han/hon beskriva sig som mycket mager, mager, medel, rund eller tjock?

barn: För närvarande, skulle du beskriva dig själv som mycket mager, mager, medel, rund eller tjock?

❍ Mycket mager [1]
❍ Mager [2]
❍ Medel [3]
❍ Rund [4]
❍ Tjock [5]

❍ Vet ej / vill ej svara [998]

[daw_p6]

P6.

förälder: Har andra människor - som familjen, en vän, en läkare - varit allvarligt oroad över att hans/hennes vikt har varit skadlig för hans/hennes kroppsliga hälsa?

barn: Har andra människor - som din familj, en vän eller läkare - varit allvarligt oroade för att din vikt kan vara ett problem för din kroppsliga hälsa?

❍ Nej [2]
❍ Ja [1]
❍ Vet ej / vill ej svara [998]

[daw_p7]

P7.

förälder: Vad tycker [Namn]? Tror han/hon att vikten har varit ett problem för hans/hennes kroppsliga hälsa?

barn: Vad tycker du? Tror du att din vikt har varit ett problem för din hälsa?

❍ Nej [2]
❍ Ja [1]
❍ Vet ej / vill ej svara [998]

Beräkna daw_m1_p_thi

[daw_p8]

P8.

förälder: Är [Namn] rädd för att gå upp i vikt eller bli tjock?

barn: Är du rädd att öka i vikt eller att bli tjock?

❍ Nej [1]
❍ Lite [2]
❍ Mycket [3]

❍ Vet ej / vill ej svara [998]

Om P8 är "mycket", visa P9

[daw_p9]

P9.

förälder: Blir [Namn] skräckslagen vid tanken på att gå upp i vikt eller bli tjock?

barn: Blir du skräckslagen vid tanken på att gå upp i vikt eller bli tjock?

❍ Nej [2]
❍ Ja [1]
❍ Vet ej / vill ej svara [998]

[daw_p10]

P10.

förälder: Om en läkare säger att [Namn] av hälsoskäl måste öka två kg i vikt, skulle han/hon tycka det vore lätt, svårt eller omöjligt att gå med på? *(Om [Namn] har en fysisk sjukdom som gör det svårt för honom/henne att öka i vikt är frågan om han/hon vill försöka, inte om han/hon kan lyckas)*

barn: Om en läkare säger att du av hälsoskäl måste öka två kg i vikt, skulle du tycka det vore lätt, svårt eller omöjligt att gå med på? *(Om du har en fysisk sjukdom som hindrar att du ökar i vikt är frågan om du vill försöka, inte om du kan lyckas)*

❍ Lätt [1]
❍ Svårt [2]
❍ Omöjligt [3]
❍ Vet ej / vill ej svara [998]

[daw_p11]

P11.

förälder: Undviker [Namn] mat som han/hon tror gör honom/henne tjock?

barn: Försöker du undvika mat som du blir tjock av?

❍ Nej [1]
❍ Lite [2]
❍ Mycket [3]
❍ Vet ej / vill ej svara [998]

Om P11 är "mycket", visa P12

[daw_p12]

P12.

förälder: Hur ofta lyckas [Namn] med detta? (t.ex. undvika fet mat)

barn: Hur ofta lyckas du med detta?

❍ Aldrig [1]
❍ Ibland [2]
❍ Oftast [3]
❍ Alltid [4]
❍ Vet ej / vill ej svara [998]

[daw_p13]

P13.

förälder: Tillbringar [Namn] mycket tid med att tänka på mat?

barn: Tillbringar du mycket tid med att tänka på mat?

❍ Nej [2]
❍ Ja [1]
❍ Vet ej / vill ej svara [998]

[daw_p14]

P14.

förälder: Ibland säger folk att de känner en sådan längtan efter mat, och att denna längtan är svår att motstå, det liknar det en missbrukare känner för droger eller alkohol.

Passar det in på [Namn]?

barn: Ibland säger folk att de känner en sådan längtan efter mat, och att denna längtan är svår att motstå, det liknar det en missbrukare känner för droger eller alkohol.

Passar det in på dig?

❍ Nej [1]
❍ Lite [2]
❍ Mycket [3]
❍ Vet ej / vill ej svara [998]

[daw_p15]

P15.

förälder: Ibland förlorar folk kontrollen över vad de äter, och då äter de mycket stora mängder mat på kort tid. Till exempel kan de öppna kylskåpet och äta allt vad de kan hitta, äter och äter tills de blir fysiskt illamående. Detta händer vanligen när de är ensamma.

Händer det för [Namn]?

barn: Ibland förlorar folk kontrollen över vad de äter, och då äter de mycket stora mängder mat på kort tid. Till exempel kan de öppna kylskåpet och äta allt vad de kan hitta, de äter och äter tills de blir fysiskt illamående. Detta händer vanligen när de är ensamma.

Passar det in på dig?

❍ Nej [2]
❍ Ja [1]
❍ Vet ej / vill ej svara [998]

Beräkna daw_m1_p_los

❖ **B. OM VILLKORET FÖR P15 ÄR UPPFYLLT VISA NEDANSTÅENDE FRÅGOR**

[daw_p16]

P16.

förälder: Under de senaste tre månaderna, hur ofta har det hänt i genomsnitt?

barn: Hur ofta har detta i genomsnitt hänt under de senaste tre månaderna?

❍ Har aldrig hänt [1]
❍ Då och då [2]
❍ Cirka en gång i veckan [3]
❍ Två gånger i veckan eller mer [4]
❍ Vet ej / vill ej svara [998]

[daw_p17]

P17.

förälder: När detta händer, har [Namn] då en känsla av att förlora kontrollen över sitt ätande?

barn: När det händer har du då en känsla av att förlora kontrollen över ditt ätande?

❍ Nej [2]
❍ Ja [1]
❍ Vet ej / vill ej svara [998]

❖ **B. VILLKORET AVSLUTAS**

[daw_p18]

P18.

förälder: De senaste tre månaderna, har [Namn] gjort något av nedanstående för att undvika att öka i vikt:

barn: Har du under de senaste tre månaderna gjort något av nedanstående för att undvika att öka i vikt:

	Nej [1]	Försöker men får inte [2]	Lite [3]	Mycket [4]	Vet ej / vill ej svara [998]
Äter mindre vid måltider [1]	☐	☐	☐	☐	☐
Hoppar över måltider [2]	☐	☐	☐	☐	☐
Är utan mat långa tider, t ex hela eller nästan hela dagen [3]	☐	☐	☐	☐	☐
förälder: Gömmer eller kastar mat som givits	☐	☐	☐	☐	☐

	Nej [1]	Försöker men får inte [2]	Lite [3]	Mycket [4]	Vet ej / vill ej svara [998]
barn: Gömmer eller kastar bort mat som du fått [4]					
Tränar mer [5]	☐	☐	☐	☐	☐
förälder: Framkallar kräkning (kastar upp)<br><br>barn: Framkallar kräkning (kastar upp) [6]	☐	☐	☐	☐	☐
Tar piller för att gå ned i vikt [7]	☐	☐	☐	☐	☐
Gör andra saker *(t.ex. är diabetiker, men tar inte insulin)* [8]	☐	☐	☐	☐	☐

Beräkna daw_p18_cond

❖ **C. OM VILLKORET FÖR P18 ÄR UPPFYLLT VISA NEDANSTÅENDE FRÅGOR**

[daw_p19]

P19.

förälder: Du berättade tidigare för oss om de gånger då [Namn] förlorar kontrollen och äter för mycket. Brukar han/hon efter sådana tillfällen ha olika vanor/rutiner för att undvika att gå upp i vikt (äta mindre, träna, kräkas, ta piller eller medicin)?

barn: Du berättade tidigare för oss om de gånger du förlorar kontrollen och äter för mycket. När det har hänt, brukar du då vanligtvis göra något för att förhindra att du ökar i vikt? (äta mindre, träna, kräkas, ta piller eller medicin)

❍ Nej [2]
❍ Ja [1]
❍ Vet ej / vill ej svara [998]

❖ **C. VILLKORET AVSLUTAS**

- **D. OM FRÅGAN BESVARATS AV EN KVINNA (FLICKA ELLER FÖRÄLDER TILL FLICKA**

[daw_p20]

P20.

förälder: Har hon haft menstruation de senaste tre månaderna?

barn: Har du haft mens de senaste tre månaderna?

❍ Nej [2]
❍ Ja [1]
❍ Vet ej / vill ej svara [998]

[daw_p21]

P21.

förälder: Har hon någonsin haft mens?

barn: Har du någonsin haft mens?

❍ Nej [2]
❍ Ja [1]
❍ Vet ej / vill ej svara [998]

Om P21 är "ja", visa P22

[daw_p22]

P22.

förälder: Tar hon några hormontabletter eller injektioner? (inklusive p-piller)

barn: Tar du några hormontabletter eller injektioner? (inklusive p-piller)

❍ Nej [2]
❍ Ja [1]
❍ Vet ej / vill ej svara [998]

- ❖ **D. VILLKORET AVSLUTAS**
- ❖ **E. OM daw_m1_p_thi, daw_m1_p_foc, daw_m1_p_los eller daw_m1_p_avo är positivt, VISA NEDANSTÅENDE FRÅGOR**

[daw_p26]

P26.

<u>förälder:</u> Du har berättat om hur [Namn] äter och om hans/hennes oro för sin vikt och kroppsform. Hur upprörd eller ledsen gör dessa saker honom/henne?

<u>barn:</u> Du har berättat om ditt sätt att äta och att du oroar dig för din vikt och kroppsform. Är du upprörd eller ledsen över detta?

❍ Inte alls [1]
❍ Lite [2]
❍ Måttligt [3]
❍ Mycket [4]
❍ Vet ej / vill ej svara [998]

[daw_p27]

P27.

<u>förälder:</u> Hur mycket har hans/hennes sätt att äta eller att oroa sig för vikt och kroppsform påverkat:

<u>barn:</u> Har ditt sätt att äta och att du oroar dig för din vikt och kroppsform påverkat:

	Inte alls [1]	Lite [2]	Måttligt [3]	Mycket [4]	Vet ej / vill ej svara [998]
a) <u>förälder:</u> hur bra han/hon kommer överens med resten av familjen?<br><br><u>barn:</u> hur bra du kommer överens med resten av familjen? [a]	☐	☐	☐	☐	☐
b) kamratrelationer - att få och behålla vänner? [b]	☐	☐	☐	☐	☐
c) inlärning eller {göromål i förskolan, skolarbete}? [c]	☐	☐	☐	☐	☐

	Inte alls [1]	Lite [2]	Måttligt [3]	Mycket [4]	Vet ej / vill ej svara [998]
d) lek, hobbies, sport eller andra fritidsaktiviteter? [d]	☐	☐	☐	☐	☐

[daw_p28]

P28.

<u>förälder:</u> Har hans/hennes sätt att äta och oron för vikt och kroppsform blivit en belastning för dig och familjen som helhet?

<u>barn:</u> Har ditt sätt att äta och att du oroar dig för din vikt och kroppsform blivit jobbigt för dem omkring dig (familj, vänner, {förskollärare, lärare} etc.)?

❍ Inte alls [1]
❍ Lite [2]
❍ Måttligt [3]
❍ Mycket [4]
❍ Vet ej / vill ej svara [998]

❖ **A OCH E. VILLKORET AVSLUTAS**

[daw_q3a_j]

Q3. Med motoriska tics avser läkare upprepade rörelser som kommer plötsligt och snabbt, som följer ungefär samma mönster varje gång de uppträder, och som kommer fastän personen egentligen inte vill.

Har [Namn] någon gång under hela sitt liv haft motoriska tics som inbegriper några av följande typer av upprepade rörelser?

	Nej [2]	Ja [1]	Vet ej / vill ej svara [998]
Överdrivna blinkningar [a]	☐	☐	☐
Höjning av ögonbrynen [b]	☐	☐	☐
Kisar eller vindar med ögonen [c]	☐	☐	☐
Ögonrullningar uppåt, neråt eller i sidled [d]	☐	☐	☐
Ryckningar kring näsan [e]	☐	☐	☐
Vidgningar av näsborrarna [f]	☐	☐	☐
Plutande med munnen (som för att ge en kyss) [g]	☐	☐	☐
Gapningar, öppnar munnen och håller den vidöppen [h]	☐	☐	☐
Nickar med huvudet [i]	☐	☐	☐
Förvrider ansiktet [j]	☐	☐	☐

[daw_q3k_n]

Q3. Har [Namn] någon gång under hela sitt liv haft motoriska tics som inbegriper några av följande typer av upprepade rörelser?

	Nej [2]	Ja [1]	Vet ej / vill ej svara [998]
Rörelse som leder till att hakan berör skuldran [k]	☐	☐	☐
Sträcker nacken [l]	☐	☐	☐
Rycker på axlarna [m]	☐	☐	☐
Ryckig rörelse i arm eller ben [n]	☐	☐	☐

Beräkna daw_q3_cond

- **A. OM VILLKORET FÖR Q3 ÄR UPPFYLLT VISA NEDANSTÅENDE FRÅGOR**

[daw_q4]

Q4. Ibland har rörelser som ser ut som tics andra orsaker. Exempelvis så kisar vissa barn med ögonen på grund av att de behöver glasögon eller på grund av att de behöver byta till starkare glasögon. På liknande sätt kan en del barn ha problem med näsa och ögon under pollensäsong.

Tror du att vissa eller alla av rörelserna för [Namn] kan ha haft andra orsaker?

❍ Nej [2]
❍ Ja [1]
❍ Vet ej / vill ej svara [998]

❖ **A. VILLKORET AVSLUTAS**

[daw_q6]

Q6. Vi ska nu ändra inriktning från motoriska till vokala tics. Vokala tics är ljud som kommer från mun, näsa eller hals. De är plötsliga och snabba, följer i stort samma mönster varje gång, och uppträder fastän personen egentligen inte vill.
Har [Namn] någon gång under hela sitt liv haft vokala tics som inbegriper några av följande typer av upprepade ljud?

	Nej [2]	Ja [1]	Vet ej / vill ej svara [998]
Harklingar [1]	☐	☐	☐
Överdrivet sniffande [2]	☐	☐	☐
Vanemässiga hostningar [3]	☐	☐	☐
Sväljningar [4]	☐	☐	☐
Gälla tjut [5]	☐	☐	☐
Utstötande av småljud, ex ”ah”, ”eh”, ”ii” [6]	☐	☐	☐
Sugande ljud [7]	☐	☐	☐
	☐	☐	☐
Ett ord som upprepas utan att det kan kopplas till sammanhanget [9]	☐	☐	☐
Svordomar utan avsikt att förarga och utan att vara förargad [10]	☐	☐	☐
	☐	☐	☐

Beräkna daw_q6_cond

❖ **B. OM VILLKORET FÖR Q6 ÄR UPPFYLLT VISA NEDANSTÅENDE FRÅGOR**

[daw_q7]

Q7. Ibland har ljud som ter sig som tics en annan förklaring. Exempelvis så harklar vissa barn sig när de är nervösa eller hostar på grund av att de har en halsirritation som beror på förkylning eller allergi.

Tror du att vissa eller alla ljud som [Namn] har kan ha andra förklaringar?

❍ Nej [2]
❍ Ja [1]
❍ Vet ej / vill ej svara [998]

❖ **B. VILLKORET AVSLUTAS**

❖ **C. OM daw_m1_q_mot ELLER daw_m1_q_voc ÄR POSITIVT, VISA NEDANSTÅENDE FRÅGOR**

[daw_q13]

Q13. Hur gammal var han/hon när ticsen kom första gången?

❍ Sedan födelsen [0]
❍ 1 år gammal [1]
❍ 2 år gammal [2]
❍ 3 år gammal [3]
❍ 4 år gammal [4]
❍ 5 år gammal [5]
❍ 6 år gammal [6]
❍ 7 år gammal [7]
❍ 8 år gammal [8]
❍ 9 år gammal [9]
❍ 10 år gammal [10]
❍ 11 år gammal [11]
❍ 12 år gammal [12]
❍ 13 år gammal [13]
❍ 14 år gammal [14]
❍ 15 år gammal [15]
❍ 16 år gammal [16]
❍ 17 år gammal [17]
❍ Vet ej / vill ej svara [998]

[daw_q14]

Q14. Vi ska nu fråga om dåliga veckor med mycket tics. Med det menar vi en vecka när ticsen kommer flera gånger om dagen, antingen varje dag eller de flesta dagar i den veckan. Har [Namn] haft någon sådan dålig vecka under det senaste året?

(Bara för att påminna, vi menar alltså åtminstone en vecka när han/hon hade mycket tics varje dag i veckan eller de flesta dagar av veckan.)

❍ Nej [2]
❍ Ja [1]
❍ Vet ej / vill ej svara [998]

[daw_q15]

Q15. När började [Namn] ha dåliga veckor med mycket tics?

❍ Mindre än en månad sedan [1]
❍ 1 till 12 månader sedan [2]
❍ Mer än ett år sedan [3]
❍ Vet ej / vill ej svara [998]

- **D. OM Q15 ÄR ”1 TILL 11 MÅNADER SEDAN” ELLER MER SÄLLAN VISA NEDAN FRÅGOR**

[daw_q16]

Q16. Ungefär hur många av veckorna det senaste året har varit dåliga veckor med mycket tics?

❍ Klart mindre än hälften [1]
❍ Ungefär hälften [2]
❍ Klart fler än hälften [3]
❍ Alla eller nästan alla [4]
❍ Vet ej / vill ej svara [998]

[daw_q17]

Q17. Under det senaste året, har [Namn] haft en period på minst 4 veckor i rad som var dåliga veckor när det gäller tics?

❍ Nej [2]
❍ Ja [1]
❍ Vet ej / vill ej svara [998]

Om q17 är "ja", visa q18

[daw_q18]

Q18. Har de senaste 4 veckorna varit dåliga veckor när det gäller tics?

❍ Nej [2]
❍ Ja [1]
❍ Vet ej / vill ej svara [998]

[daw_q19]

Q19. Vissa barn/ungdomar har tics vecka in och vecka ut - fastän mönstret och antalet tics inte nödvändigtvis är lika varje vecka. Andra barn har veckor eller månader när ticsen försvinner helt. Har [Namn] under det senaste året haft någon period utan tics som varat i veckor eller månader?

❍ Nej [2]
❍ Ja [1]
❍ Vet ej / vill ej svara [998]

Om q19 är "ja", visa q20

[daw_q20]

Q20. Hur lång var den längsta perioden utan tics det senaste året?

❍ Mindre än 3 månader [1]
❍ 3 månader [2]
❍ Mer än 3 månader [3]
❍ Vet ej / vill ej svara [998]

❖ **D. CONDITION STOP**

[daw_q21]

Q21. Hur oroad eller plågad har [Namn] blivit av alla sina tics?

❍ Inte alls [1]
❍ Lite [2]
❍ Måttligt [3]
❍ Mycket [4]

❍ Vet ej / vill ej svara [998]

[daw_q22]

Q22. Har ticsen påverkat...

	Inte alls [1]	Lite [2]	Måttligt [3]	Mycket [4]	Vet ej / vill ej svara [998]
hur bra han/hon kommer överens med resten av familjen? [1]	☐	☐	☐	☐	☐
kamratrelationer - att få och behålla vänner? [2]	☐	☐	☐	☐	☐
inlärning eller {göromål i förskolan, skolarbete}? [3]	☐	☐	☐	☐	☐
lek, hobbies, sport eller andra fritidsaktiviteter? [4]	☐	☐	☐	☐	☐

[daw_q23]

Q23. Har ticsen varit en belastning för dig eller för familjen som helhet?

❍ Inte alls [1]
❍ Lite [2]
❍ Måttligt [3]
❍ Mycket [4]
❍ Vet ej / vill ej svara [998]

- **C. VILLKORET AVSLUTAS**

- **OM ÅLDER >= 15 ANVÄND MODULERNA PERSONLIGHET, SKREENING OCH UPPFÖLJNINGSFRÅGOR I TEMAT MENTAL HÄLSA FÖR VUXNA**
